# A visible-light activated secondary phosphine oxide ligand enabling Pd-catalyzed radical cross-couplings

**DOI:** 10.1038/s41467-022-31613-9

**Published:** 2022-07-13

**Authors:** Takahito Kuribara, Masaya Nakajima, Tetsuhiro Nemoto

**Affiliations:** Graduate School of Pharmaceutical Sciences, Chiba University, 1-8-1, Inohana, Chuo-ku, Chiba, 260-8675 Japan

**Keywords:** Synthetic chemistry methodology, Photocatalysis, Photocatalysis

## Abstract

Although transition metal-catalyzed reactions have evolved with ligand development, ligand design for palladium-catalyzed photoreactions remains less explored. Here, we report a secondary phosphine oxide ligand bearing a visible-light sensitization moiety and apply it to Pd-catalyzed radical cross-coupling reactions. The tautomeric phosphinous acid coordinates to palladium in situ, allowing for pseudo-intramolecular single-electron transfer between the ligand and palladium. Molecular design of the metal complexes aided by time-dependent density functional theory calculations enables the involvement of allyl radicals from π-allyl palladium(II) complexes, and alkyl and aryl radicals from the corresponding halides and palladium(0) complex. This complex enables radical cross-couplings by ligand-to-Pd(II) and Pd(0)-to-ligand single-electron transfer under visible-light irradiation.

## Abstract (web summary)

Ligand design is key for improving the performance in light-enabled catalytic processes. Here, the authors report the synthesis of a visible-light–activated secondary phosphine oxide ligand and apply it to Pd-catalyzed radical cross-coupling reactions.

## Introduction

Pd-catalyzed cross-coupling is an important C–C bond formation methodology for the synthesis of pharmaceuticals, bioactive molecules, agrochemicals, and other functional molecules^1–3^. Transition metal catalysts have evolved along with ligand development. Ligand tuning expands the reactivity of transition metals, increases turnover number, and allows for reactions under mild conditions; therefore, the design and synthesis of electronically and sterically controlled ligands have created major breakthroughs in palladium chemistry^4^.

**Fig. 1:**
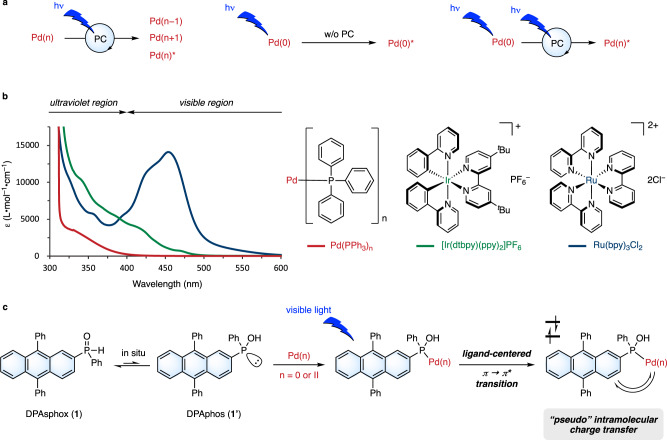
Pd-catalyzed photoreactions. **a** Classification of photo-induced Pd-catalyzed reactions. **b** Absorption coefficient of Pd(0) complex is smaller than Ir and Ru photoredox complexes in visible region. UV-Vis spectra were measured in *N*,*N*-dimethylacetamide (DMA). **c** Concept of visible-light–activated secondary phosphine oxide ligand (DPAsphox (**1**)). PC photocatalyst, dtbpy 4,4'-di-tert-butyl-2,2'-bipyridyl, ppy 2-phenylpyridine, bpy 2,2'-bipyridine.

Owing to the development of LED light and visible-light photocatalysts, transition metal-catalyzed photoreactions have recently attracted the increased attention of many chemists. The Pd-catalyzed photoreactions can be classified into three modes (Fig. 1a), as follows: (1) those in which the external photocatalyst is the sole light-absorbing species to proceed along three pathways: oxidative single electron transfer (SET), reductive SET, and energy transfer^5–7^; (2) those in which a Pd catalyst absorbs light^8–10^; and (3) those in which the external photocatalyst and Pd catalyst absorb light and the energy transfers from the photocatalyst to the Pd-containing intermediate^11^ (Fig. 1a). The generation of such a highly active palladium species enables versatile transformations. Moreover, although UV-light irradiation causes nonselective excitation of almost all materials, visible-light irradiation can selectively excite photocatalysts or Pd complexes. Thus, reports of Pd-catalyzed photoreactions have increased in recent years. Although the reaction mechanisms of photoreactions are quite different from those of thermal reactions, phosphine ligands, which are well-studied and designed for thermal reactions, have been applied for photoreactions. The development of ligands that orient photoreactions is limited^12^. In addition, the absorption coefficient of the d → p transition^13,14^ of the Pd(0) complex in the visible-light region is much smaller than that of other transition metals such as Ru and Ir photoredox catalysts (Fig. 1b). Hence, we expected that the development of ligands that absorb visible light will enhance the applications of transition metals.

To develop a ligand for photoreactions, we planned to synthesize a phosphine ligand with a photosensitive moiety. We first attempted to synthesize a tertiary phosphine bearing 9,10-diphenylanthracene (DPA), but it was unstable and thus easily oxidized under air (Supplementary Fig. 25). Therefore, we use a secondary phosphine oxide (DPAsphox (**1**)) as a pre-ligand (Fig. 1c), which acts as a ligand through the phosphorus atom after in situ tautomerization to phosphinous acids (DPAphos (**1**'))^15,16^. This ligand design enables the efficient absorption of visible light and pseudo-intramolecular electron or energy transfer to the metal center. Moreover, unlike photoredox catalysts, this metal complex enables a ligand-centered π → π* transition on DPAphos because the DPA moiety does not directly coordinate to palladium. Thus, we hypothesized that the excited DPAphos would generate Pd(I) by one-electron oxidation of Pd(0) via metal-to-ligand charge transfer (MLCT) or one-electron reduction of Pd(II) via ligand-to-metal charge transfer (LMCT) to promote further various radical reactions. The yielded DPA(•–) or (•+) exhibits opposite reactivities, indicating that different oxidation states of Pd(0 or II) can produce quite different reactivities.

Herein, we report the design and synthesis of DPAsphox and describe two different Pd-catalyzed radical cross-coupling reactions using Pd(0) or Pd(II) under visible-light irradiation.

## Results

### Computational studies of Pd complexes

**Fig. 2:**
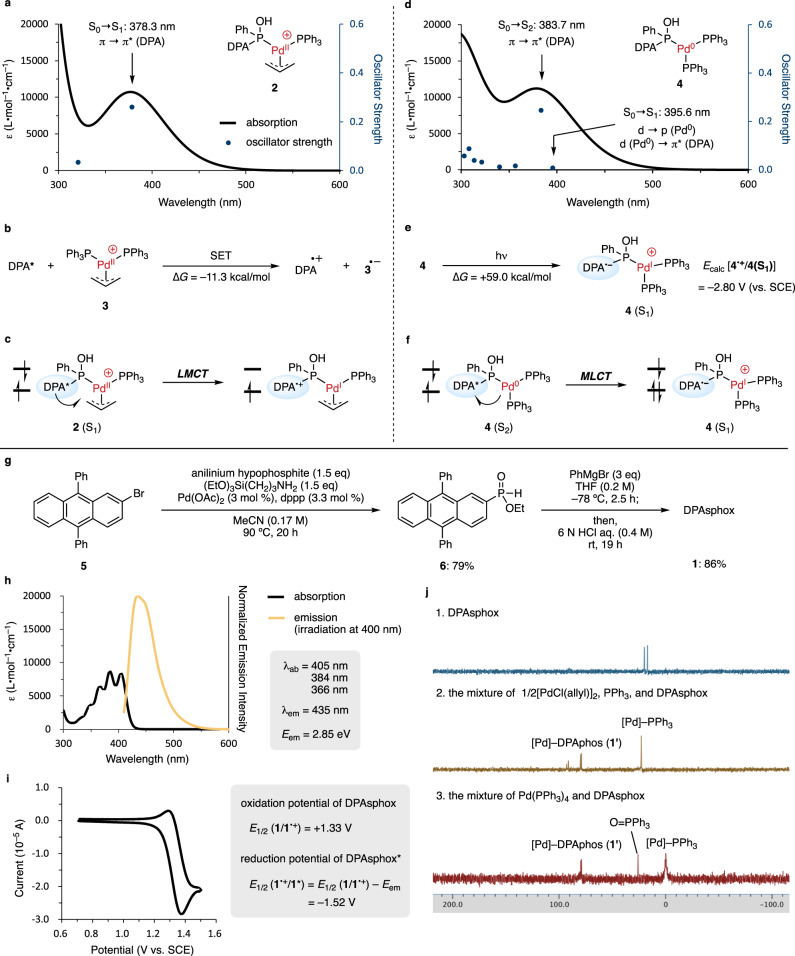
Calculated excited states of Pd-DPAphos complexes and synthesis of DPAsphox (1). **a** UV-Vis spectrum of Pd(II) complex (**2**) calculated with ωB97X-D/SDD, 6-311 + G(d,p)//MN15/SDD, 6-31 G(d) in DMF (PCM). **b** Intermolecular SET from DPA* to Pd(II) complex (**3**) is a thermodynamically favored pathway. **c** LMCT after π → π* transition on complex **2** produces Pd(I) and DPA^•+^. **d** UV-Vis spectrum of Pd(0) complex (**4**) calculated with ωB97X-D/SDD, 6-311 + G(d,p)//MN15/SDD, 6-31 G(d) in DMA (PCM). **e** Calculation of the first singlet excited state of **4**. **f** MLCT after π → π* transition on complex **4** produces Pd(I) and DPA^•−^. **g** Synthesis of DPAsphox. **h** UV-Vis spectra of DPAsphox in DMF. **i** Cyclic voltammogram and redox potential of DPAsphox. **j** ^31^P NMR studies of the mixture of Pd(0 or II) complex and DPAsphox. DPA 9,10-diphenylanthracene, SET single electron transfer, LMCT ligand-to-metal charge transfer, MLCT metal-to-ligand charge transfer, dppp 1,3-Bis(diphenylphosphino)propane, THF tetrahydrofuran, SCE saturated calomel electrode.

We first calculated the absorption wavelength of the Pd(II)-DPAphos complex using time-dependent density functional theory (TD-DFT) calculations (Fig. 2a). The S_0_ → S_1_ absorption of Pd(II) complex (**2**) was calculated as the ligand-centered π → π* transition on DPA, and its wavelength expanded up to 500 nm, indicating that visible-light irradiation enables selective excitation of the DPA moiety. We concluded that this excited DPA moiety in the Pd(II) complex enabled intramolecular one-electron reduction of Pd(II) to afford Pd(I) and DPA^•+^ via LMCT. Thus, we next individually compared the reduction potential of DPA and Pd(II). The reduction potential of DPA* (*E*_1/2_ [DPA^•+^/DPA*] = –1.68 V vs. SCE) (Supplementary Information, Section 1–7) was lower than that of allyl Pd(II) complex **3** (*E*_1/2_ [**3/3**^**•−**^] = –1.35 V vs. SCE)^17^ and the one-electron reduction of **3** afforded 11.3 kcal/mol stable states (Fig. 2b), indicating that a rapid LMCT in the excited state of **2** would proceed to afford Pd(I) and DPA^•+^ (Fig. 2c).

We next calculated the absorption wavelength of Pd(0) complex (**4**); the ligand-centered π → π* transition on DPA was S_0_ → S_2_ at 383.7 nm, and the metal-centered d → p transition and d → π* MLCT were S_0_ → S_1_ at 395.6 nm (Fig. 2d). Although these metal-centered and MLCTs (S_0_ → S_1_) showed longer wavelengths than the ligand-centered transitions (S_0_ → S_2_), the oscillator strength of the S_0_ → S_1_ transition was 32 times smaller than that of the S_0_ → S_2_ transition, suggesting that the ligand-centered S_0_ → S_2_ transition favorably proceeds by irradiation under near 400 nm light. Furthermore, in the calculated S_1_ state, the singlet biradicals were located on the singly occupied molecular orbital (SOMO) of the π* orbital of the DPA moiety and on the SOMO–1 of the d orbital of palladium (Fig. 2e), meaning that the radiationless transition from S_2_ afforded the S_1_ state via MLCT (Fig. 2f). This MLCT pathway is supported by the oxidation potential of DPA in the excited state (*E*_1/2_ [DPA^•–^/DPA*] = +0.93 V vs. SCE) (Supplementary Information, Section 1–7), which is higher than that of Pd(PPh_3_)_4_ (*E*_1/2_ [Pd(0)/Pd(I)] = –0.03 V vs. SCE)^18^. Therefore, the most favorable ligand-centered excitation (S_0_ → S_2_) facilitates the formation of **4**(S_1_) via MLCT (S_2_ → S_1_), and its high reduction potential (*E*_calc_ [**4**^•+^/**4**(S_1_)] = –2.80 V vs. SCE) may cause a further radical reactions such as one-electron reduction of alkyl and aryl halides to generate the carbon-centered radicals.

### Synthesis and experimental analysis of DPAsphox

DPAsphox **1** was synthesized, and the stability, spectroscopic and electrochemical properties, and coordination ability to palladium were evaluated. Pd-catalyzed C–P cross-coupling and esterification of 2-bromo-9,10-diphenylanthracene (**5**) with anilinium hypophosphite afforded ethyl phosphinate **6** in 79% yield^19^, and the subsequent nucleophilic substitution with phenyl magnesium bromide produced DPAsphox in 86% yield (Fig. 2g). The stability of DPAsphox was tested, and the purity was maintained for at least 1 week under refrigeration (Supplementary Table 7). Absorption and emission spectra of the synthesized DPAsphox revealed S_0_ → S_1_ absorption at 405 nm and S_1_ → S_0_ emission at 435 nm (2.85 eV) (Fig. 2h). In addition, cyclic voltammetry showed that the oxidation potential of DPAsphox was +1.33 V (vs. SCE) (Fig. 2i). Thus, the reduction potential of excited DPAsphox was estimated to be −1.52 V (vs. SCE) by Rehm-Weller formalism^20^. The coordination of DPAsphox to Pd(II) and Pd(0) complexes was next investigated by ^31^P NMR (Fig. 2j). Stirring DPAsphox with [PdCl(allyl)]_2_ and PPh_3_ in DMF-*d*_*7*_ for 1 hour led to the disappearance of the doublet peak at 18.5 ppm of DPAsphox, and the emergence of new peaks between 79.1 and 92.8 ppm, suggesting that DPAphos (**1'**) derived from DPAsphox coordinates to palladium^15^. In addition, stirring with Pd(PPh_3_)_4_ also caused a peak shift between 79.1 and 79.8 ppm. Therefore, as we expected, DPAsphox can be a stable pre-ligand for DPAphos to become a Pd-DPAphos complex.

**Fig. 3:**
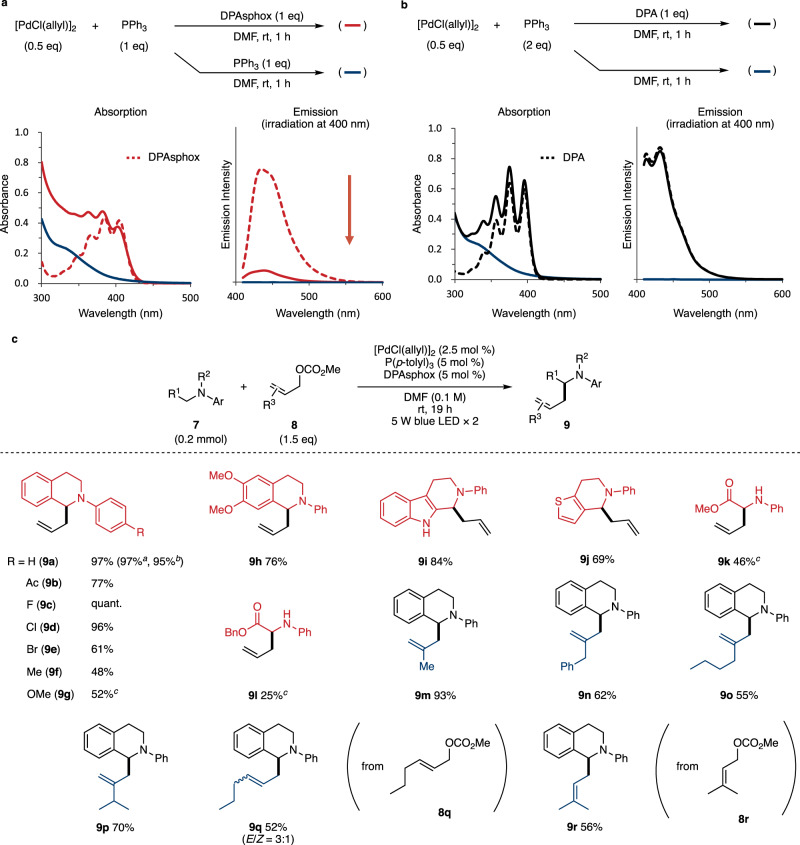
Pd-catalyzed α-allylation of amines. **a** UV-Vis analysis with DPAsphox in DMF (0.05 mM). **b** UV-Vis analysis with DPA in DMF (0.05 mM). **c** Substrate scope. ^a^1.0 mmol scale. ^b^Allyl alcohol was used instead of **8a**. ^c^2,4,6-collidine (1 equiv.) was added. DMF *N*,*N*-dimethylformamide, DPA 9,10-diphenylanthracene, *p*-tolyl *p*-methylphenyl.

### Radical cross-couplings using a Pd(II) complex

We performed Pd-catalyzed radical cross-coupling reactions under visible-light irradiation to demonstrate the utility of DPAsphox. We first verified the reactivity of the allyl-Pd(II)-DPAphos complex (Table 1 and Fig. 3). Using *N*-phenyl-1,2,3,4-tetrahydroisoquinoline (**7a**) and allyl methyl carbonate (**8a**), *C*-allylated product **9a** was obtained in 75% yield under purple-light irradiation (400 nm) (Table 1, entry 1)^21^. Irradiation with blue LED light (450 nm) showed the similar performance to give the product (**9a**) in 76 % yield (entry 2). Control experiments in entries 3 and 4 indicated that Pd catalyst and visible-light irradiation are essential for the allylation reaction. When using ligands such as PPh_3_, DPEphos, and Xantphos, which cannot absorb visible light by themselves, no allylated compound (**9a**) was obtained (entries 5 and 6). The reaction with DPA instead of DPAsphox also afforded **9a** in 25% yield (entry 7), suggesting that the pseudo-intramolecular electron transfer of DPAphos improved the yield in comparison with intermolecular SET with DPA.

**Table 1:** Establishment of the reaction condition.


Entry	Ligand and additive	Yield (%)^a^ of 9a
1	PPh_3_ (5 mol %), DPAsphox (5 mol %)	75
2^b^	PPh_3_ (5 mol %), DPAsphox (5 mol %)	76
3^c^	PPh_3_ (5 mol %), DPAsphox (5 mol %)	trace
4^d^	PPh_3_ (5 mol %), DPAsphox (5 mol %)	n.d.
5	PPh_3_ (10 mol %)	trace
6	DPEphos or Xantphos (5 mol %)	n.d.
7	PPh_3_ (10 mol %), DPA (5 mol %)	25

DMF *N*,*N*-dimethylformamide, n.d. not detected, DPEphos Bis[(2-diphenylphosphino)phenyl] ether, Xantphos 4,5-Bis(diphenylphosphino)-9,9-dimethylxanthene, DPA 9,10-diphenylanthracene.

^a^Yields were determined by ^1^H NMR analysis using triphenylmethane as an internal standard.

^b^Blue LED were used instead of purple LED.

^c^The reaction was performed without [PdCl(allyl)]_2_.

^d^The reaction was performed under light-shielding condition.

Thus, we next carried out a fluorescence quenching study of DPAphos and DPA to investigate the efficiency of the SET from DPAphos to Pd(II) (Fig. 3a, b). The absorption spectrum of the mixture of DPAsphox, [PdCl(allyl)]_2_, and PPh_3_ (solid red line) showed the same intensity as DPAsphox (dotted red line) at around 400 nm, meaning that the DPA moiety of the DPAphos absorbs the light (Fig. 3a). In contrast, the emission intensity was strongly suppressed by the coordination of DPAphos to palladium, clearly suggesting that quenching proceeded via LMCT from excited DPAphos to Pd(II). On the other hand, no quenching was observed when we performed the same spectroscopic study using DPA instead of DPAsphox (Fig. 3b). The results of these quenching studies suggest that coordination of DPAphos to Pd(II) enables highly efficient LMCT by pseudo-intramolecular electron transfer.

We therefore investigated the substrate scope of Pd-catalyzed α-allylation of amines (Fig. 3c). Amine **7a** and 1.5 equivalents of allyl methyl carbonate **8a** yielded the product **9a** in 97% yield under 5 W blue LED lights. The desired **9a** was also obtained with 1.0 mmol of **7a** in the same yield. Furthermore, the reaction proceeded using allyl alcohol instead of **8a** to provide **9a** in 95% yield. Substituents at the para position of aniline were examined (**9b-g**): acetyl (77%), fluoride (quant.), chloride (96%), bromide (61%), methyl (48%), and methoxy (52%). A bromo group was applicable for this allylation in the presence of a Pd catalyst (**9e**). Electron-donating groups, however, decreased the yields (**9** **f, g**), and the product **9** **g** with a methoxy group was obtained in 52% yield when 2,4,6-collidine was added as a Brønsted base to facilitate deprotonation at the α-position of amine^22^. 6,7-Dimethoxy tetrahydroisoquinoline derivative (**9** **h**) and indole- and thiophene-fused piperidine derivatives (**9i, j**) were obtained in 76%, 84%, 69% yields, respectively. The *C*-allylation of amino acids also proceeded to give **9k** (46%) and **9** **l** (25%). Next, we investigated the substrate scope of allyl methyl carbonate (**9m-r**). Allyl methyl carbonate with β-methyl, benzyl, *n*-butyl, and isopropyl groups yielded the product in 93%, 62%, 55%, and 70%, respectively. In addition, using γ-substituted allyl methyl carbonate **8q** and **8r**, allylic alkylation proceeded in a linear-selective manner to afford **9q** (52%) and **9r** (56%).

### Radical cross-couplings using a Pd(0) complex

**Fig. 4:**
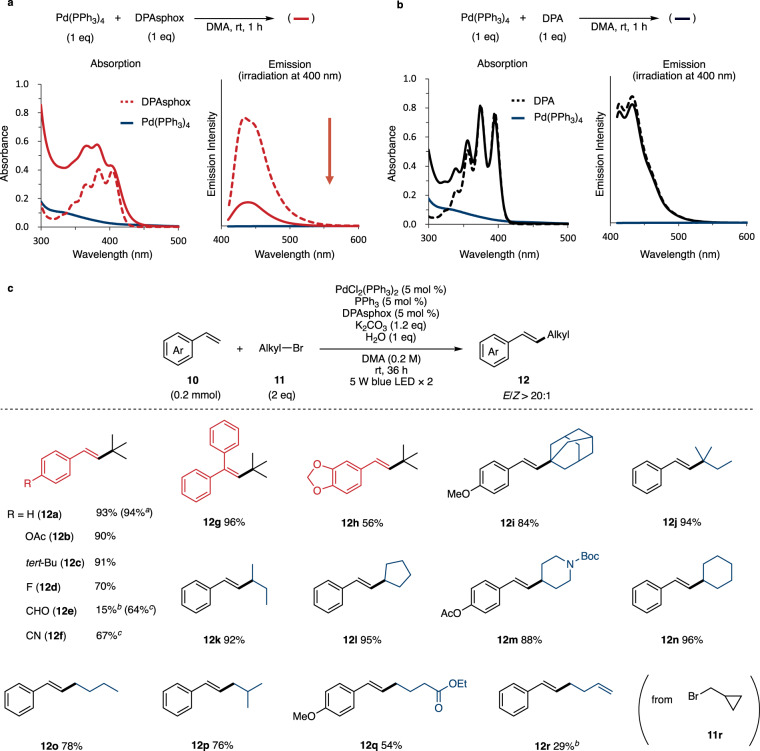
Pd-catalyzed Heck reaction of unactivated alkyl bromide. **a** UV-Vis analysis with DPAsphox in DMA (0.05 mM). **b** UV-Vis analysis with DPA in DMA (0.05 mM). **c** Substrate scope. ^a^1.0 mmol scale. ^b^Yield determined by ^1^H NMR analysis. ^c^P(OPh)_3_ (5 mol %) was added. DMA *N*,*N*-dimethylacetamide, DPA 9,10-diphenylanthracene.

The reactivity and spectroscopic properties of the Pd(0) complex were next evaluated under blue-light irradiation (Table 2 and Fig. 4). Control experiments of the Heck reaction were performed with styrene (**10a**) and unactivated tertiary alkyl bromide (**11a**) (Table 2)^23–26^. The addition of 5 mol % of Pd(PPh_3_)_4_ and DPAsphox provided β-alkylated styrene (**12a**) in 79% yield under 5 W blue LED lights (entry 1). In situ generation of Pd(0) complex from 5 mol % of Pd(PPh_3_)_2_Cl_2_, PPh_3_, and DPAsphox improved the yield of **12a** in 93% (entry 2). Under conditions without DPAsphox (entry 3) and with DPA instead of DPAsphox (entry 4), **12a** was produced in low yield, indicating that DPAsphox plays an important role as a visible-light–activated ligand. Shang and Fu reported this kind of photo-induced Heck reaction, in which they achieved a high yield and broad substrate generality using Xantphos;^25^ the reaction required intense light, however, such as a 36 W blue LED light, because Xantphos does not absorb visible light. Thus, under our 5 W blue LED condition, the photoreaction with Xantphos gave the product in 57% yield (entry 5).

**Table 2:** Control experiments.


Entry	Pd catalyst	Ligand and additive	Yield (%)^a^ of 12a
1	Pd(PPh_3_)_4_	DPAsphox (5 mol %)	79
2	PdCl_2_(PPh_3_)_2_	PPh_3_ (5 mol %), DPAsphox (5 mol %)	93
3	PdCl_2_(PPh_3_)_2_	PPh_3_ (5 mol %)	19
4	PdCl_2_(PPh_3_)_2_	PPh_3_ (5 mol %), DPA (5 mol %)	31
5	PdCl_2_(PPh_3_)_2_	Xantphos (5 mol %)	57

^a^Isolated yields were shown. DMA *N*,*N*-dimethylacetamide, DPA 9,10-diphenylanthracene, Xantphos 4,5-Bis(diphenylphosphino)-9,9-dimethylxanthene.

Thus, we next carried out a UV-Vis spectroscopic and fluorescence quenching study using Pd(0) and DPAsphox to clarify the interaction between them (Fig. 4a). The absorption intensity of the mixture of Pd(PPh_3_)_4_ and DPAsphox was stronger (solid red line) than that of Pd(PPh_3_)_4_ alone (blue line) because of the π → π* transition of DPAphos (dotted red line). The emission of DPAphos (dotted red line) was clearly suppressed in the presence of Pd(PPh_3_)_4_ (solid red line), indicating that pseudo-intramolecular quenching occurred in the Pd(0)-DPAphos complex. Additionally, *tert*-BuBr (**11a**) did not affect the absorption and emission spectra (Supplementary Figs. 37, 38), suggesting that no EDA complex formed between Pd(0) and **11a**. Furthermore, the mixture of Pd(PPh_3_)_4_ and DPA (solid black line) showed almost no change in the absorption and emission spectra compared with DPA alone (dotted black line) (Fig. 4b) similar to the Pd(II) complex. Together, these results support the high efficiency of our visible-light–activated ligand.

The substrate scope of the photo-Heck reaction was investigated (Fig. 4c). The β-alkylated styrene **12a** was also obtained in 94% yield with 1.0 mmol of styrene. The *para*-substituted styrenes were examined next, and styrenes with acetoxy (90%), *tert*-butyl (91%), and fluoro groups (70%) afforded the corresponding alkylated styrenes (**12b-d**). The yield was decreased by electron-withdrawing groups at the para-position^26^, but the addition of triphenyl phosphite improved the yield (**12e, f**). This effect was also observed using Xantphos as a ligand (Supplementary Information, Section 1–15). 1,1-Diphenylethene (**10** **g**) and a piperonal derivative (**10** **h**) were applicable to this photo-Heck reaction. Other tertiary alkyl bromides, such as 1-adamantyl and 2-methylbutyl bromides, gave the products in 84% (**12i**) and 94% (**12j**) yields. Secondary butyl (92%) and cyclic alkyl groups (88%-96%) were also introduced at the β-position of styrene (**12k-n**). In addition, primary alkyl bromides afforded the products with high *E*/*Z* selectivity (>20:1) (**12o-q**), and the radical clock experiment using (bromomethyl)cyclopropane yielded the ring-opened product **12r**, indicating that this reaction involves a radical process.

**Fig. 5:**
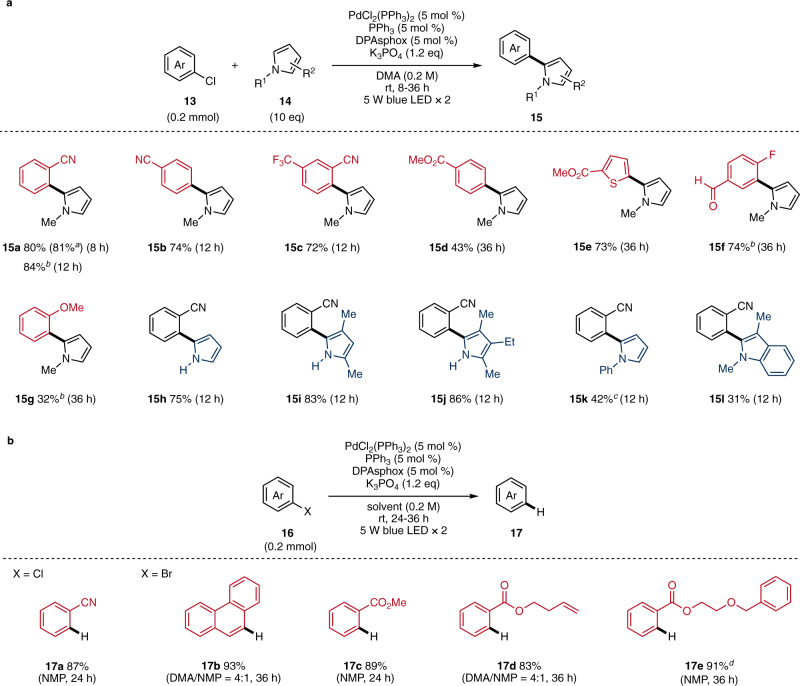
Pd-catalyzed biaryl synthesis and dehalogenative hydrogenation. **a** Substrate scope of biaryl synthesis. **b** Substrate scope of dehalogenative hydrogenation. ^a^1.0 mmol scale. ^b^Aryl bromide was used as a substrate. ^c^*N*-phenylpyrrole (5 equiv.) was used. ^d^Solvent (0.1 M) was used. DMA *N*,*N*-dimethylacetamide, NMP *N*-Methyl-2-pyrrolidone.

We next applied this photo-ligand-Pd(0) system to a one-electron reduction of aryl bromide and chloride, examples for which are quite limited in Pd-catalyzed photoreactions^27^. Cross-coupling of aryl halide and pyrroles was performed with PdCl_2_(PPh_3_)_2_, PPh_3_, and DPAsphox under blue-light irradiation (Fig. 5a). Biarene **15a** was obtained from 2-chlorobenzonitrile and *N*-methyl pyrrole in 80% (from 0.2 mmol of **13a**) and 81% yield (from 1.0 mmol of **13a**). **15a** was also obtained from 2-bromobenzonitrile in 84% yield. Biaryl compounds (**15b-e**) were also afforded by 4-chlorobenzonitrile (74%), 2-chloro-5-trifluoromethyl benzonitrile (72%), methyl 4-chlorobenzoate (43%), and methyl 5-chlorothiophene-2-carboxylate (73%). This reaction was also applicable for coupling using aryl bromide; 3-bromo-4-fluorobenzaldehyde (74%) and 2-bromoanisole (32%) gave the corresponding products (**15** **f, g**). The substrate scope of radical acceptors was examined next, and various pyrroles were applicable: *N*-H pyrrole (**15** **h**, 75%), 2,4-dimethyl pyrrole (**15i**, 83%), 3-ethyl-2,4-dimethyl pyrrole (**15j**, 86%), *N*-Ph-pyrrole (**15k**, 42%), and 1,3-dimethylindole (**15** **l**, 31%).

Dehalogenative hydrogenations of aryl chloride and bromide were demonstrated (Fig. 5b). The hydrogenation of 2-chlorobenzonitrile proceeded in 87% yield using *N*-methylpyrrolidone (NMP) as a solvent and a hydrogen donor (**17a**)^28^. 9-Bromophenanthrene and methyl 2-bromobenzoate yielded the corresponding arenes in 93% and 89% yields (**17b, c**). In addition, our reaction system selectively hydrogenated halide on arenes in the presence of terminal olefin and benzyl ether in the substrates (**17d, e**).

### Reaction mechanisms of Pd-catalyzed radical cross-couplings

**Fig. 6:**
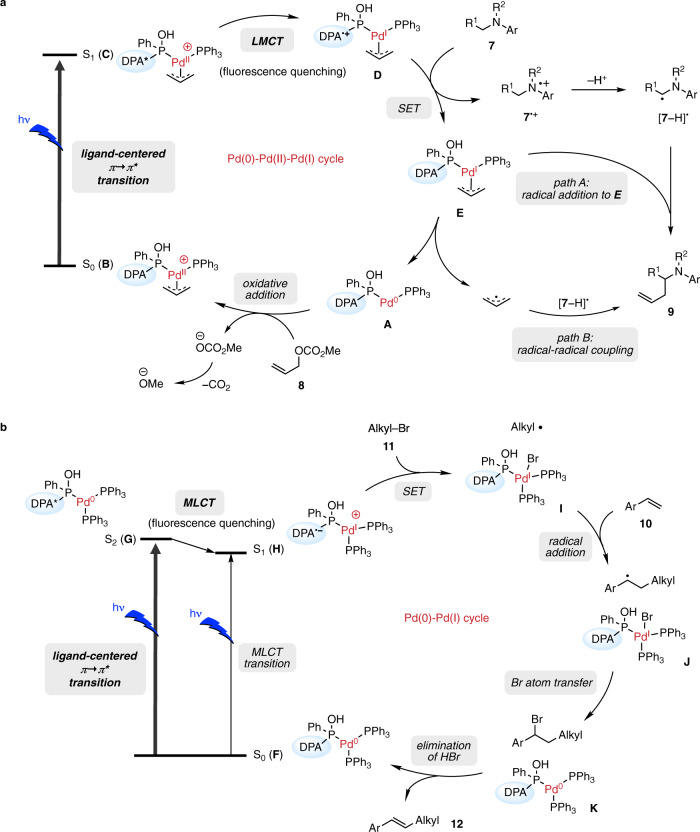
Proposed reaction mechanisms of Pd-catalyzed radical cross-couplings. **a** Pd-catalyzed α-allylation of amines. **b** Pd-catalyzed photo-Heck reaction. DPA 9,10-diphenylanthracene, LMCT ligand-to-metal charge transfer, SET single electron transfer, MLCT metal-to-ligand charge transfer.

Finally, the reaction mechanisms of α-allylation of amines with Pd(II) (Fig. 3) and a photo-Heck reaction with Pd(0) (Fig. 4) are considered and compared in Fig. 6. The process involving light is the ligand-centered excitation for both reactions, namely the π → π* transition of the DPA moiety, but the subsequent quenching path differs between them: a one-electron reduction of Pd(II) (LMCT) and a one-electron oxidation of Pd(0) (MLCT).

In the allylation reaction, oxidative addition of allyl methyl carbonate **8** to Pd(0) complex **A** forms redox-active π-allyl Pd(II) complex **B** at room temperature^29^. Although the direct reduction pathway of **8** by excited Pd(0) complex **A** cannot be excluded, the formation of π-allyl Pd(II) complex **B** is plausible because no ligands other than DPAsphox functioned (Table 1) and allyl alcohol was also applicable for this allylation reaction (Fig. 3c)^30^. The eliminated methyl carbonate generates methoxide anions through decarboxylation and acts as a base. The highly efficient pseudo-intramolecular one-electron reduction (LMCT) from the S_1_ state (**C**) generated the biradical state **D**; fluorescence quenching supported this process (Fig. 3b). The following one-electron oxidation of amine (**7**) by DPA^•+^ afforded the corresponding **7**^•+^. Then, the free radical [**7** − H]^•^ generated from **7**^•+^ by deprotonation reacted with the allyl-Pd(I) complex (**E**) (path A) or free allyl radical (path B) to give the product **9** and complex **A**^21^.

On the other hand, in the reaction mechanisms of the Pd-catalyzed photo-Heck reaction, Pd(I) cation species (**H**) would be generated as the S_1_ state by pseudo-intramolecular one-electron oxidation of Pd(0) from an excited DPA moiety via an S_0_ → S_2_ → S_1_ transition. Although the S_0_ → S_1_ transition directly produces cationic Pd(I) (**H**), the oscillator strength of the transition was 32 times smaller than that of the S_0_ → S_2_ transition (Fig. 2d), indicating that the S_0_ → S_2_ → S_1_ transition would be the primary excitation mechanism of this complex. Because the DPA^•−^ moiety of **H** has a high reduction potential, alkyl bromide **11** (*E*_1/2_ [**11**/**11**^•−^] = −2.1 to −2.4 V)^31^ is readily reduced to give alkyl radical and Pd(I) complex **I**. After the addition of alkyl radical to styrene **10** (**I** → **J**) and the following bromo atom transfer from Pd(I) to the benzyl radical (**J** → **K**), Pd(0) complex **F** is regenerated, and β-alkylated styrene **12** is provided by elimination of HBr, which is supported by the KIE experiment (Supplementary Information, Section 1–15)^25,26^.

## Discussion

A visible-light–activated secondary phosphine oxide ligand was developed for Pd-catalyzed radical cross-couplings. The ligand-centered π → π* transition of DPAphos promoted allyl radical-mediated cross-coupling via LMCT in the allyl Pd(II) complex, and alkyl and aryl radical-mediated cross-couplings via MLCT in the Pd(0) complex. The efficient SET was observed by spectroscopic studies as a strong quenching of the fluorescence emission of DPAphos. This was achieved by the visible-light–sensitizing moiety (DPA) in the secondary phosphine oxide ligand. Our strategy for designing a visible-light–activated ligand and metal complexes showed potential for tuning the electronic state of transition metals under visible-light irradiation. Further development of ligands to orient transition-metal-catalyzed photoreactions is expected.

## Methods

### General information

NMR spectra were recorded on JEOL-JMN-ECS 400 or ECZ 400 spectrometers. Data for NMR are reported as follows: chemical shift (δ ppm), multiplicity (s singlet, br-s broad singlet, d doublet, t triplet, q quartet, and m multiplet), coupling constants (Hz), and integration. Chemical shifts are reported in the scale relative to TMS (0.0 ppm) for ^1^H NMR and the solvent signal (CHCl_3_ (77.0 ppm)) for ^13^C NMR. ^19^F and ^31^P NMR spectra are referenced to external hexafluorobenzene and 85% phosphoric acid. Infrared (IR) spectra were recorded on a Fourier transform infrared spectrophotometer equipped with ATR. High-resolution mass spectra were measured on a JEOL AccuTOF LC-plus JMS-T100LP instrument (ionization method: ESI). Melting points were measured with a SIBATA NEL-270 melting point apparatus. The absorption and emission spectra were measured by a JASCO V-730 spectrophotometer and FP-8500 spectrofluorometer. Column chromatographic purification was performed with silica gel 60 N (spherical, neutral 40-50 μm), and preparative TLC purification was performed with TLC silica gel 60 F_254_. The Pd-catalyzed reactions were carried out with standard Schlenk techniques under Ar atmosphere. Unless otherwise noted, photochemical reactions were performed with degassed solvents by freeze-pump-thaw cycles three times.

### Computational methods

All calculations were performed with the Gaussian 16 program^32^. Structure optimizations were carried out at 298.15 K, using the MN15^33^ functional with an ultrafine grid and the SDD^34^ (for Pd) and 6-31 G(d) (for the other atoms) basis sets. DMF (for Pd(II) complex) and *N*,*N*-dimethylacetamide (DMA) (for Pd(0) complex) were used as implicit solvents using PCM^35^ as a solvation model. Harmonic vibrational frequencies were computed at the same level of theory to confirm that no imaginary vibration was observed for the optimized structure. Single-point energy calculations were performed for all geometries at 298.15 K, using the ωB97X-D^36^ functional with an ultrafine grid and the SDD (for Pd) and 6-311 + G(d,p) (for the other atoms) basis sets with the same solvation model. The Gibbs free energy was calculated by the sum of total electronic energy in the single-point energy calculation and the thermal correction energy in the frequency calculation. All molecular orbitals were computed at an isovalue of 0.02.

### Procedure for the synthesis of DPAsphox (1)

A 100 mL Schlenk tube containing a magnetic stirring bar was charged with 2-bromo-9,10-diphenylanthracene **5** (1.23 g, 3.0 mmol, 1.0 equiv.), anilinium hypophosphite (716 mg, 4.5 mmol, 1.5 equiv.), (3-aminopropyl)triethoxysilane (1.06 mL, 4.5 mmol, 1.5 equiv.), Pd(OAc)_2_ (20.2 mg, 0.090 mmol, 3.0 mol %), dppp (40.8 mg, 0.099 mmol, 3.3 mol %), and MeCN (18.0 mL, 0.17 M), and purged with argon. After stirring for 20 h at 90 °C, the reaction mixture was evaporated and EtOAc (20 mL) was added. The organic layer was washed with water (3.0 mL), 1 N HCl aq. (3.0 mL), sat. NaHCO_3_ aq. (3.0 mL), and brine (3.0 mL), dried over Na_2_SO_4_, and concentrated. The crude product was purified by flash column chromatography (*n*-hexane/EtOAc = 2/1 to 1/1) to afford ethyl phosphinate **6** (998.2 mg, 2.36 mmol) in 79% yield as yellow amorphous. Next, an oven-dried 100 mL Schlenk tube containing a magnetic stirring bar was charged with **6** (998.2 mg, 2.36 mmol, 1.0 equiv.) and THF (11.8 mL, 0.20 M) under argon. To the cooling solution at –78 °C, 1 M PhMgBr in THF (7.1 mL, 3.0 equiv.) was added dropwise. After stirring for 2.5 h at the same temperature, the reaction was quenched with 6 N HCl aq. (5.9 mL, 0.40 M), and stirred for 19 h at room temperature. Then, the organic layer was extracted with EtOAc (10 mL × 3), washed with brine (3 mL), dried over Na_2_SO_4_, and concentrated. The crude product was purified by flash column chromatography (*n*-hexane/EtOAc = 1/1 to 1/4) to afford DPAsphox (**1**) (920.9 mg, 2.03 mmol) in 86% yield as yellow amorphous.

### General procedure for α-allylation of amines

A 20 mL Schlenk tube containing a magnetic stirring bar was charged with amine **7** (0.20 mmol, 1.0 equiv.), [PdCl(allyl)]_2_ (1.8 mg, 2.5 mol %), P(*p*-tolyl)_3_ (3.0 mg, 5.0 mol %), DPAsphox (4.5 mg, 5.0 mol %), allyl methyl carbonate **8** (0.30 mmol, 1.5 equiv.), and DMF (2.0 mL, 0.10 M). After the reaction mixture was degassed by freeze-pump-thaw cycles three times, it was stirred for 19 h at room temperature under irradiation with 5 W blue LED lights. Then, water (3.0 mL) was added to the reaction, and the aqueous layer was extracted with EtOAc (3.0 mL × 3). The combined organic layer was washed with water (3.0 mL × 3) and brine (3.0 mL), dried over Na_2_SO_4_, and concentrated. The crude product was purified by flash column chromatography or preparative TLC to afford the corresponding product **9**.

### General procedure for Heck reaction of unactivated alkyl bromide

A 20 mL Schlenk tube containing a magnetic stirring bar was charged with PdCl_2_(PPh_3_)_2_ (7.0 mg, 5.0 mol %), PPh_3_ (2.6 mg, 5.0 mol %), DPAsphox (4.5 mg, 5.0 mol %), and K_2_CO_3_ (33.2 mg, 1.2 equiv.). After the tube was evacuated and filled with argon, styrene **10** (0.20 mmol, 1.0 equiv.), alkyl bromide **11** (0.40 mmol, 2.0 equiv.), distilled water (3.6 mL, 1.0 equiv.), and degassed DMA (1.0 mL, 0.20 M) were added under a stream of argon. The reaction mixture was stirred for 36 h at room temperature under irradiation with 5 W blue LED lights. Then, water (3.0 mL) was added to the reaction, and the aqueous layer was extracted with EtOAc (3.0 mL × 3). The combined organic layer was washed with water (3.0 mL × 3) and brine (3.0 mL), dried over Na_2_SO_4_, and concentrated. The crude product was purified by flash column chromatography to afford the corresponding product **12**.

### General procedure for biaryl synthesis

A 20 mL Schlenk tube containing a magnetic stirring bar was charged with PdCl_2_(PPh_3_)_2_ (7.0 mg, 5.0 mol %), PPh_3_ (2.6 mg, 5.0 mol %), DPAsphox (4.5 mg, 5.0 mol %), K_3_PO_4_ (50.9 mg, 1.2 equiv.), aryl halide **13** (0.20 mmol, 1.0 equiv.), pyrrole **14** (2.0 mmol, 10.0 equiv.), and DMA (1.0 mL, 0.20 M). After the reaction mixture was degassed by freeze-pump-thaw cycles three times, it was stirred at room temperature under irradiation with 5 W blue LED lights. Then, water (3.0 mL) was added to the reaction, and the aqueous layer was extracted with EtOAc (3.0 mL × 3). The combined organic layer was washed with water (3.0 mL × 3) and brine (3.0 mL), dried over Na_2_SO_4_, and concentrated. The crude product was purified by flash column chromatography to afford the corresponding product **15**.

### General procedure for dehalogenative hydrogenation

A 20 mL Schlenk tube containing a magnetic stirring bar was charged with PdCl_2_(PPh_3_)_2_ (7.0 mg, 5.0 mol %), PPh_3_ (2.6 mg, 5.0 mol %), DPAsphox (4.5 mg, 5.0 mol %), K_3_PO_4_ (50.9 mg, 1.2 equiv.), aryl halide **16** (0.20 mmol, 1.0 equiv.), and solvent as specified. After the reaction mixture was degassed by freeze-pump-thaw cycles three times, it was stirred at room temperature under irradiation with 5 W blue LED lights. Then, water (3.0 mL) was added to the reaction, and the aqueous layer was extracted with EtOAc (3.0 mL × 3). The combined organic layer was washed with water (3.0 mL × 3) and brine (3.0 mL), dried over Na_2_SO_4_, and concentrated. The crude product was purified by flash column chromatography to afford the corresponding product **17**.

## Supplementary information

Supplementary Information file

## Data Availability

All data generated in this study are provided in the Supplementary Information.
