# Supplementary material for: A visible-light activated secondary phosphine oxide ligand enabling Pd-catalyzed radical cross-couplings
Source: Nat Commun. 2022 Jul 13;13:4052. doi: 10.1038/s41467-022-31613-9 (PMC9279477; doi:10.1038/s41467-022-31613-9)
Supplement: Supplementary file 1 — Supplementary Information file [file 41467_2022_31613_MOESM1_ESM.pdf]

Supplementary Information

A Visible-Light–Activated Secondary Phosphine Oxide Ligand Enabling Pd-Catalyzed Radical Cross-Couplings

Takahito Kuribara, Masaya Nakajima,* and Tetsuhiro Nemoto*

Graduate School of Pharmaceutical Sciences, Chiba University, 1-8-1, Inohana, Chuo-ku, Chiba 260-8675, Japan.

*E-mail: m.nakajima@chiba-u.jp (M.N.)

*E-mail: tnemoto@faculty.chiba-u.jp (T.N.)

Table of Contents

1. Supplementary Methods S3
1-1. General information S3
1-2. Computational methods S4
1-3. Benchmark studies for computational methods S5
1-4. Computational analysis S14
1-5. Synthesis of DPAsphox S33
1-6. Voltammetric study S35
1-7. Calculation of the redox potential of excited state S37
1-8. Stability test of DPAsphox S38
1-9. ^{31}P NMR study S40
1-10. Optimization of the reaction conditions S42
1-11. Preparation of substrates S46
1-12. General procedure S48
1-13. Characterization of reaction products S50
1-14. UV-Vis spectra S55
1-15. Supplemental experiments S64
1-16. Cartesian coordinates and energies S68
2. Supplementary Figures S93
2-1. NMR spectra S93
3. Supplementary References S111

1. Supplementary Methods

1-1. General information

NMR spectra were recorded on JEOL-JMN-ECS400 or ECZ400 spectrometers. Data for NMR are reported as follows: chemical shift (δ ppm), multiplicity (s = singlet, br-s = broad singlet, d = doublet, t = triplet, q = quartet, and m = multiplet), coupling constants (Hz), and integration. Chemical shifts are reported in the scale relative to TMS (0.0 ppm) for ^{1}H NMR and the solvent signal ($CHCl_3$ (77.0 ppm)) for ^{13}C NMR. ^{19}F and ^{31}P NMR spectra are referenced to external hexafluorobenzene and 85% phosphoric acid. Infrared (IR) spectra were recorded on a Fourier transform infrared spectrophotometer equipped with ATR. High-resolution mass spectra were measured on a JEOL AccuTOF LC-plus JMS-T100LP instrument (ionization method: ESI). Melting points were measured with a SIBATA NEL-270 melting point apparatus. The absorption and emission spectra were measured by a JASCO V-730 spectrophotometer and FP-8500 spectrofluorometer. Column chromatographic purification was performed with silica gel 60 N (spherical, neutral 40-50 μm), and preparative TLC purification was performed with TLC silica gel 60 F_{254}. The Pd-catalyzed reactions were carried out with standard Shlenk techniques under Ar atmosphere. Unless otherwise noted, photochemical reactions were performed with solvents degassed by freeze-pump-thaw cycles three times.

1-2. Computational methods

All calculations were performed with the Gaussian 16 program.[1] Structure optimizations were carried out at 298.15 K, using the MN15[2] functional with an ultrafine grid and the SDD[3] (for Pd) and 6-31G(d)[4] (for the other atoms) basis sets. *N*,*N*-dimetylformamide (DMF) (for ligand and Pd(II) complex) and *N*,*N*-dimethylacetamide (DMA) (for Pd(0) complex) were used as implicit solvents using PCM[5] as a solvation model. Harmonic vibrational frequencies were computed at the same level of theory to confirm that no imaginary vibration was observed for the optimized structure. Single-point energy calculations were performed for all geometries at 298.15 K, using the MN15, ωB97XD[6] and CAM-B3LYP[7] functionals with an ultrafine grid and the SDD (for Pd) and 6-311+G(d,p)[8] (for the other atoms) basis sets with the same solvation model. The Gibbs free energy was calculated by the sum of total electronic energy in the single-point energy calculation and the thermal correction energy in the frequency calculation. All molecular orbitals were computed at an isovalue of 0.02.

1-3. Benchmark studies for computational methods

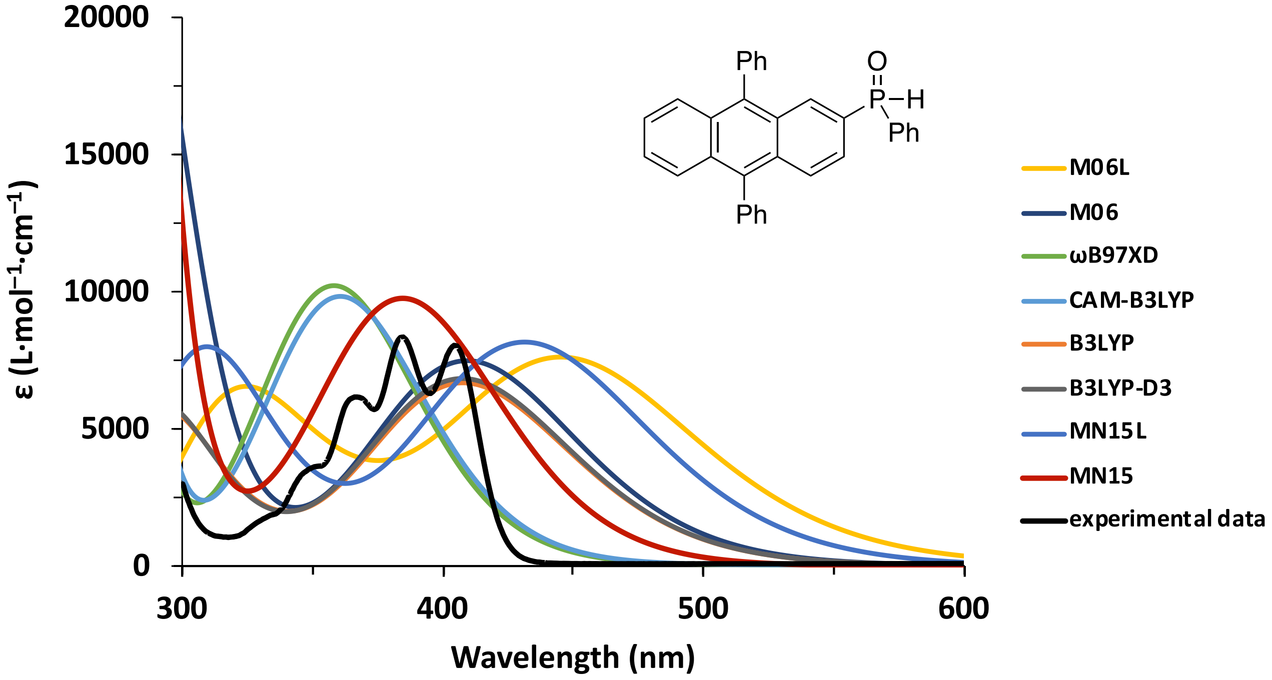

Supplementary Figure 1. UV-Vis absorption spectra of DPAsphox using functionals/6-31+G(d) in DMF (PCM).

A benchmark study of functionals for structural optimization of DPAsphox (**1**) was performed by comparing calculated and experimental UV-Vis absorption spectra as shown in Supplementary Figure 1. The absorption spectrum with MN15 functional showed good agreement with the experimental result.

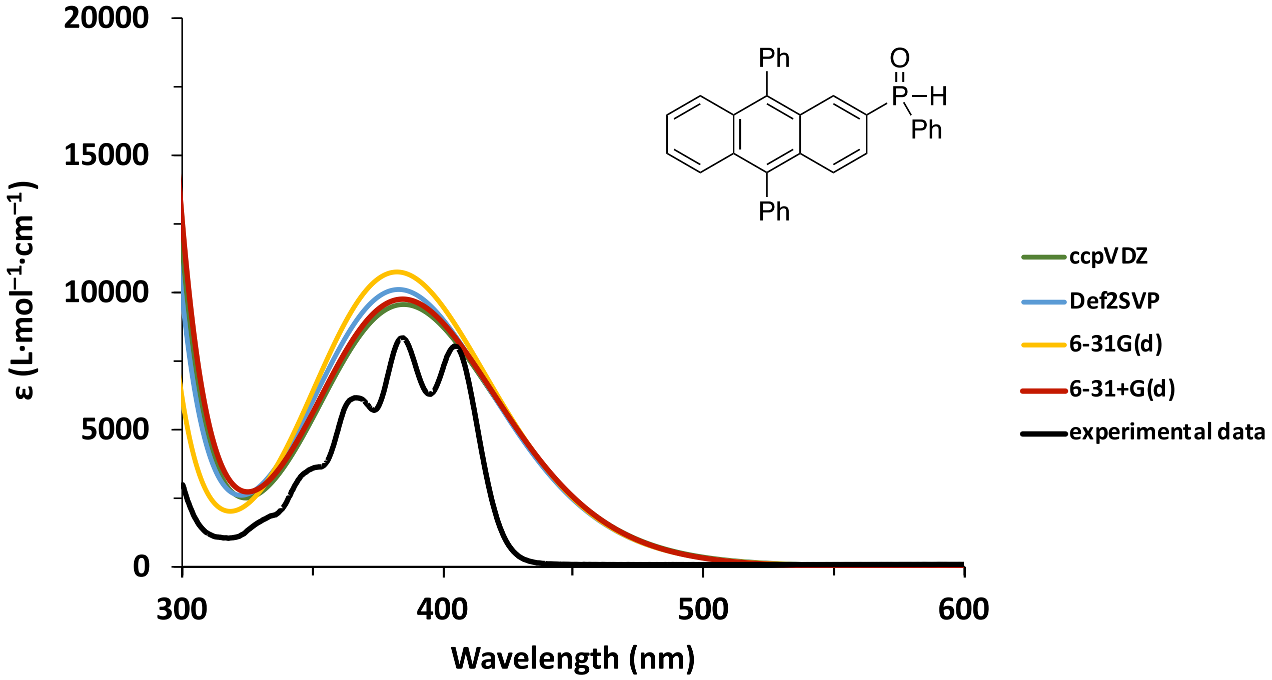

Supplementary Figure 2. UV-Vis absorption spectra of DPAsphox using MN15/basis sets in DMF (PCM).

A benchmark study of basis sets for structural optimization of DPAsphox was performed by comparing calculated and experimental UV-Vis absorption spectra as shown in Supplementary Figure 2. There was almost no change among the basis sets.

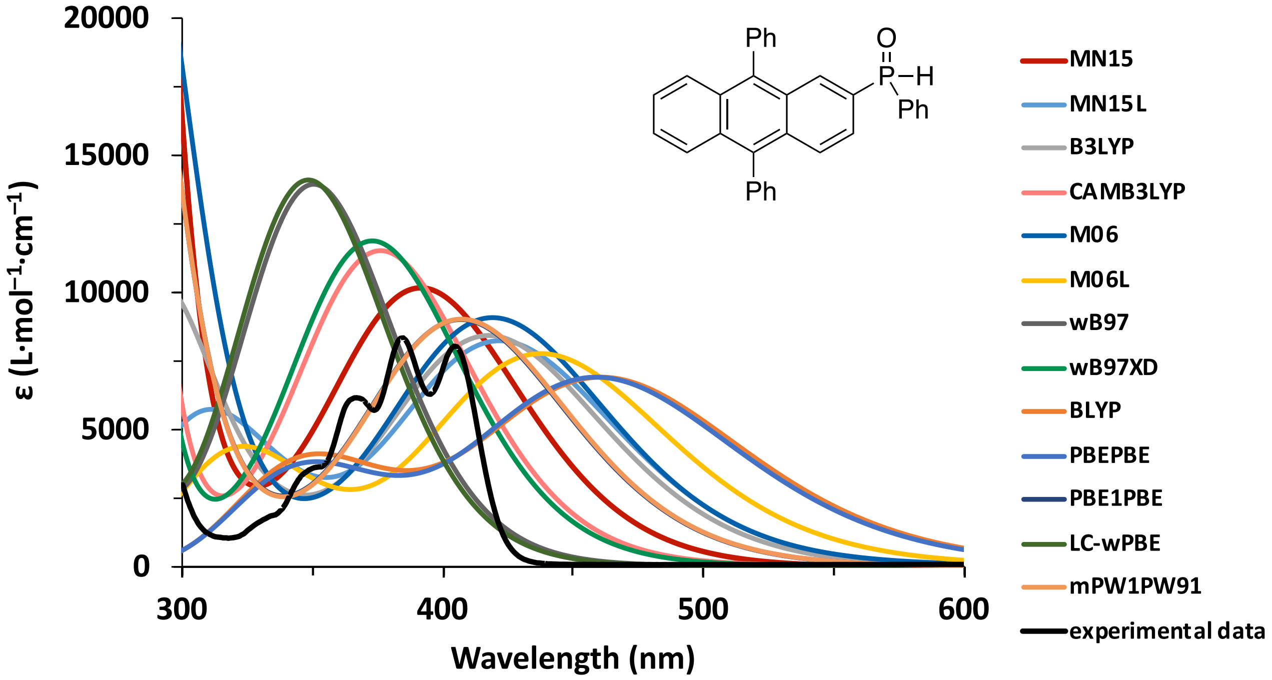

Supplementary Figure 3. UV-Vis spectra of DPAsphox using functionals/6-311+G(d,p)//MN15/6-31G(d) in DMF (PCM).

A benchmark study of functionals for single point energy calculation of DPAsphox was performed by comparing calculated and experimental UV-Vis absorption spectra as shown in Supplementary Figure 3. The UV-Vis spectrum of MN15 functional showed good agreement with the experimental result.

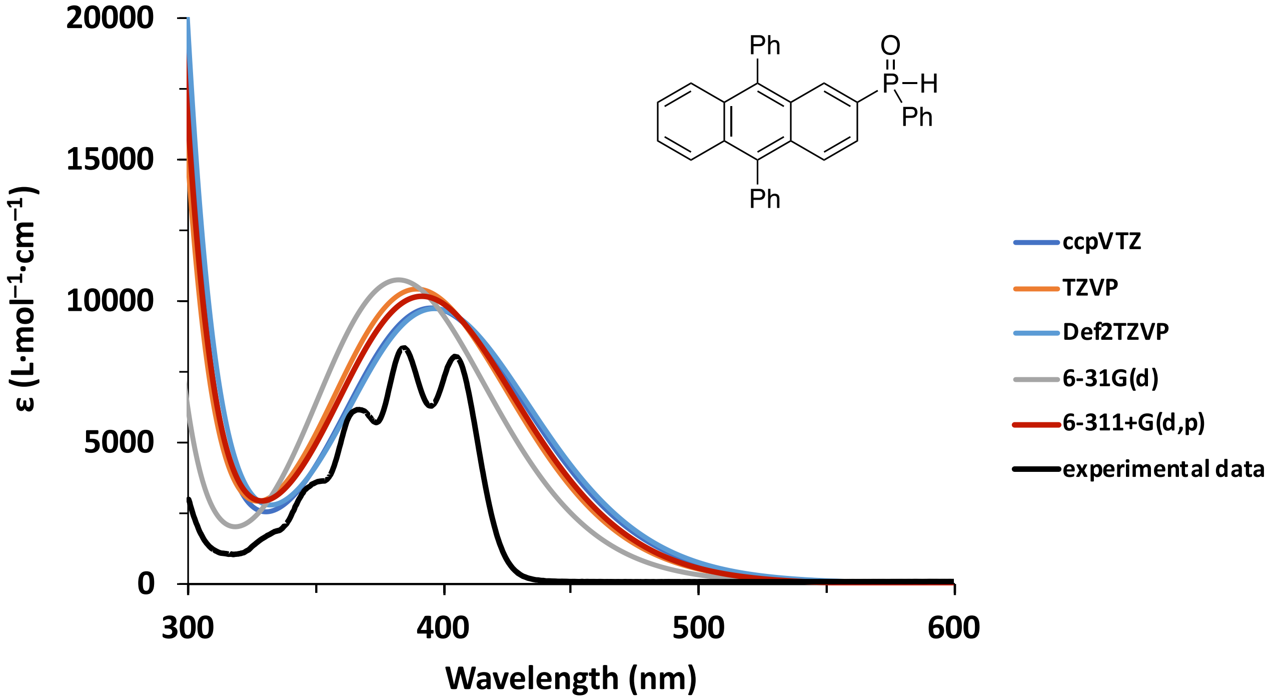

Supplementary Figure 4. UV-Vis spectra of DPAsphox using MN15/basis sets//MN15/6-31G(d) in DMF (PCM).

A benchmark study of basis sets for single point energy calculation of DPAsphox was performed by comparing calculated and experimental UV-Vis absorption spectra as shown in Supplementary Figure 4. There was almost no change among the basis sets.

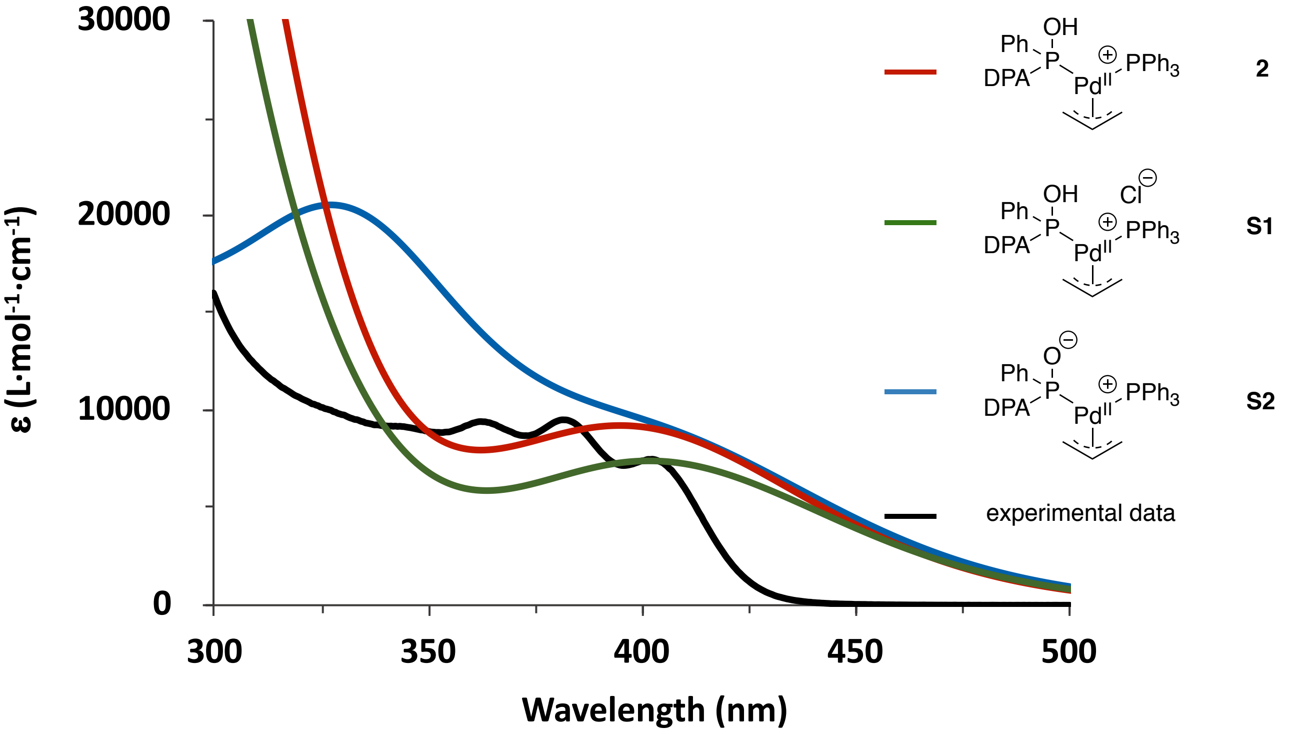

Supplementary Figure 5. UV-Vis spectra of π-allyl Pd(II) complex using MN15/SDD, 6-311+G(d,p)//MN15/SDD, 6-31G(d) in DMF (PCM).

A benchmark study of the structures of π-allyl Pd(II) complex was performed by comparing calculated and experimental UV-Vis spectra as shown in Supplementary Figure 5. The UV-Vis spectra of no counter anion **2** (red line) and chloride anion **S1** (green line) showed the similar shape as the experimental spectrum. To minimize the computational costs and simplify the structure, complex **2** was selected for further calculations.

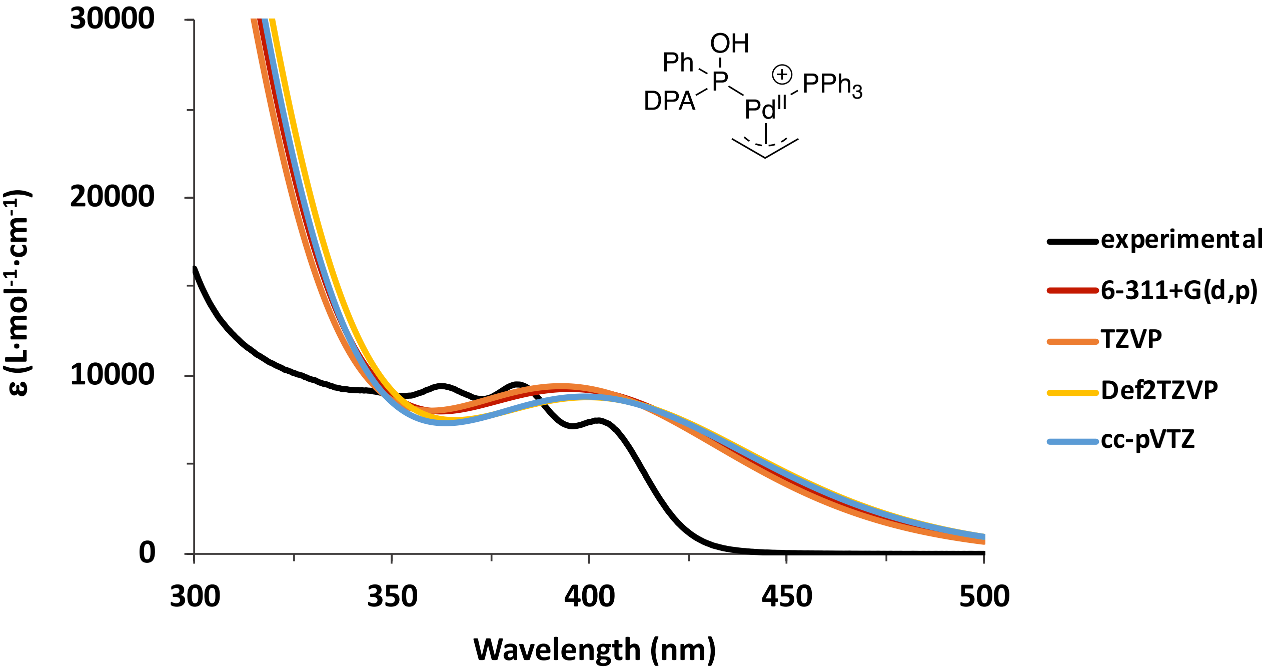

Supplementary Figure 6. UV-Vis spectra of Pd(II) complex **2** using MN15/SDD, basis sets//MN15/SDD, 6-31G(d) in DMF (PCM).

A benchmark study of basis sets for single point energy calculation of Pd(II) complex **2** was performed by comparing calculated and experimental UV-Vis spectra as shown in Supplementary Figure 6. There was almost no change among the basis sets.

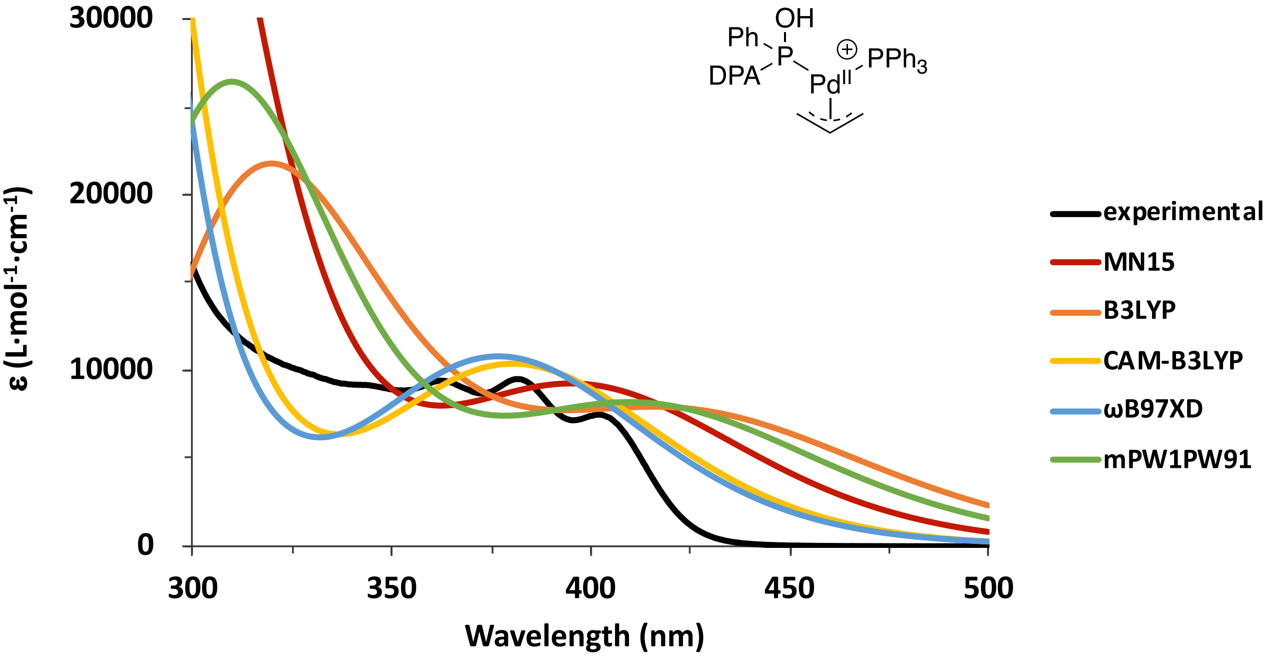

Supplementary Figure 7. UV-Vis spectra of Pd(II) complex **2** using functionals/SDD, 6-311+G(d,p)//MN15/SDD, 6-31G(d) in DMF (PCM).

A benchmark study of functionals for single point energy calculation of π-allyl Pd(II) complex (**2**) was performed by comparing calculated and experimental UV-Vis absorption spectra as shown in Supplementary Figure 7. The absorption spectra with ωB97XD and CAM-B3LYP functionals showed good agreement with the experimental result.

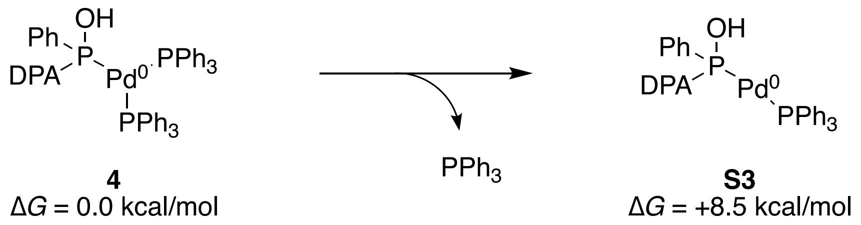

The three-coordinate Pd(0) complex (**4**) was 8.5 kcal/mol lower in energy than the two-coordinate Pd(0) complex (**S3**). Thus, complex **4** was chosen for further calculation.

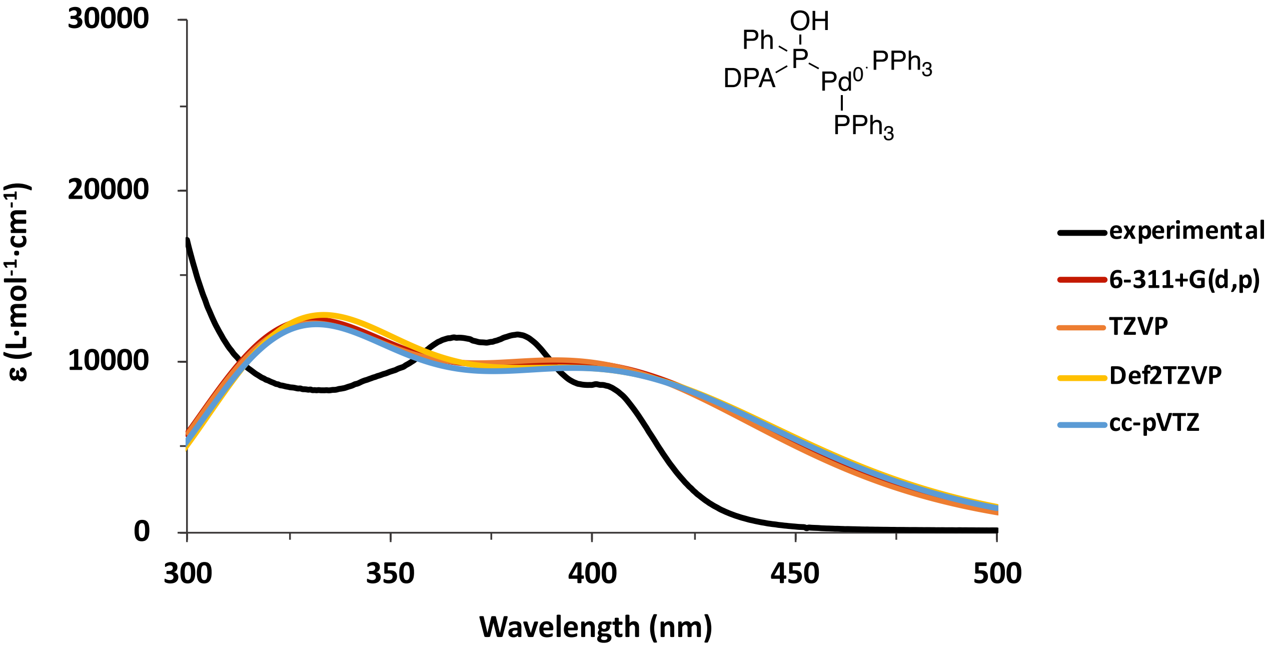

Supplementary Figure 8. UV-Vis spectra of Pd(0) complex **4** using MN15/SDD, basis sets//MN15/SDD, 6-31G(d) in DMA (PCM).

A benchmark study of basis sets for single point energy calculation of Pd(0) complex **4** was performed by comparing calculated and experimental UV-Vis absorption spectra as shown in Supplementary Figure 8. There was almost no change among the basis sets.

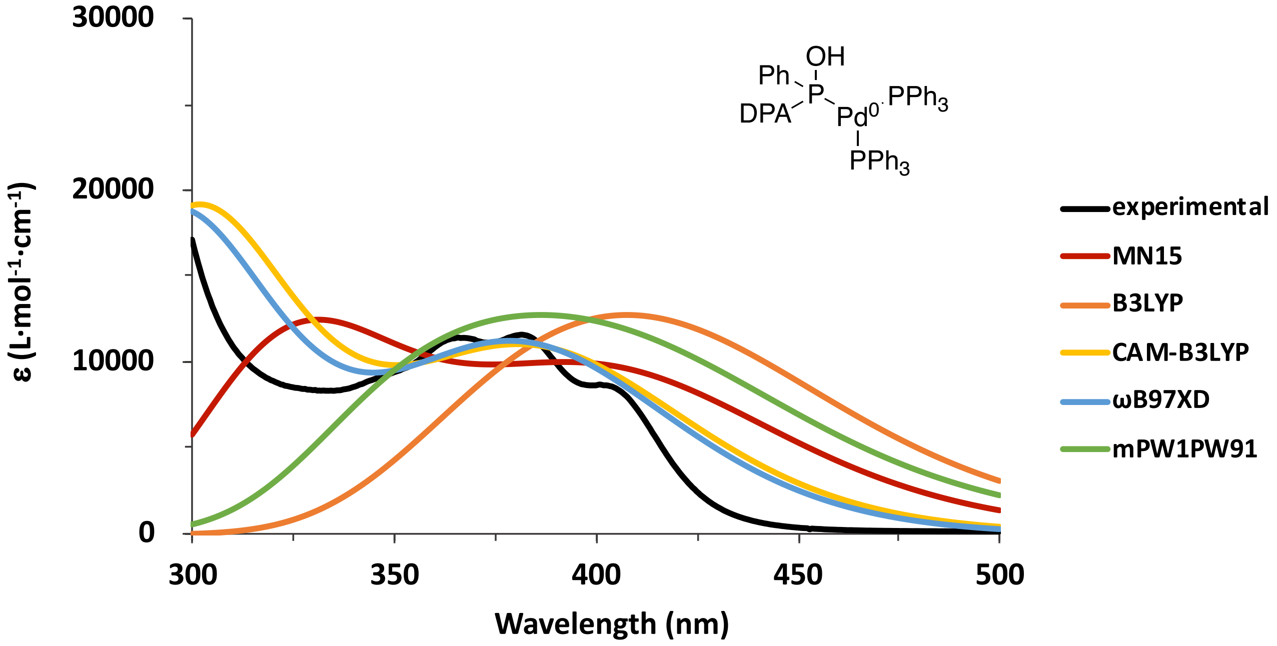

Supplementary Figure 9. UV-Vis spectra of Pd(0) complex **4** using functionals/SDD, 6-311+G(d,p)//MN15/SDD, 6-31G(d) in DMA (PCM).

A benchmark study of functionals for single point energy calculation of Pd(0) complex **4** was performed by comparing calculated and experimental UV-Vis absorption spectra as shown in Supplementary Figure 9. The absorption spectra with ωB97XD and CAM-B3LYP functionals showed good agreement with the experimental result.

1-4. Computational analysis

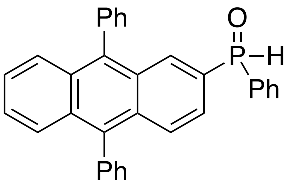

To estimate the reduction potential of excited state of DPAsphox (**1**), the following equation was used.[9]

$$E_{calc}(\text{DPAsphox}^{\bullet+}/\text{DPAsphox}^*) = E_{calc}(\text{DPAsphox}/\text{DPAsphox}^{\bullet+}) - E_{0,0}$$

$$= +1.26 \text{ (V vs. SCE)} - 2.86 \text{ (eV)} = -1.60 \text{ (V vs. SCE)}$$

E_{calc}(DPAsphox/DPAsphox$^{\bullet+}$) is the half-wave potential of DPAsphox calculated by the reported method.[10] $E_{0,0}$ is the difference in Gibbs free energy between the ground state and the first singlet excited state. The calculated reduction potential of DPAsphox* showed good agreement with the experimental value ($E_{1/2}$ = –1.52 V vs. SCE).

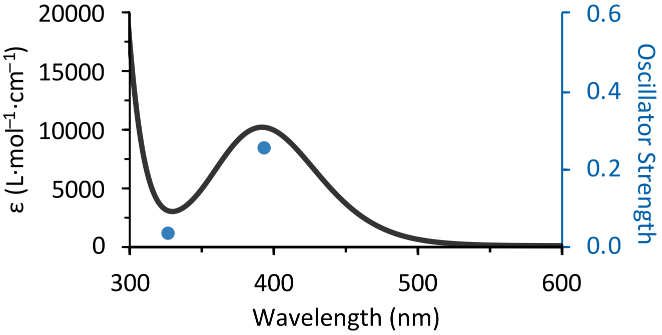

Supplementary Figure 10. Calculated UV-Vis absorption spectra and oscillator strength of DPAsphox using MN15/6-311+G(d,p)//MN15/6-31G(d) in DMF (PCM).

Supplementary Table 1. Output of TD-DFT calculation of DPAsphox using MN15/6-311+G(d,p)//MN15/6-31G(d) in DMF (PCM).

excited state	wavelength (nm)	oscillator strength	major contribution of molecular orbitals
1	392.42	0.2489	HOMO→LUMO (99%)
2	327.04	0.0321	HOMO–1→LUMO (34%)<br>HOMO→LUMO+1 (54%)
3	269.97	0.3890	HOMO–1→LUMO (10%)<br>HOMO→LUMO+3 (47%)<br>HOMO→LUMO+5 (11%)
4	268.28	0.0262	HOMO–2→LUMO (74%)<br>HOMO→LUMO+3 (10%)
5	265.74	1.3508	HOMO–3→LUMO (14%)<br>HOMO–1→LUMO (41%)<br>HOMO→LUMO+1 (31%)
6	264.78	0.0848	HOMO–3→LUMO (24%)<br>HOMO→LUMO+2 (10%)<br>HOMO→LUMO+4 (44%)
7	262.87	0.1215	HOMO–4→LUMO (36%)<br>HOMO–3→LUMO (12%)<br>HOMO→LUMO+4 (29%)
8	262.21	0.1640	HOMO→LUMO+2 (68%)<br>HOMO→LUMO+4 (17%)
9	259.78	0.0893	HOMO–4→LUMO (45%)<br>HOMO–3→LUMO (28%)
10	251.63	0.0532	HOMO–5→LUMO (72%)<br>HOMO→LUMO+7 (11%)

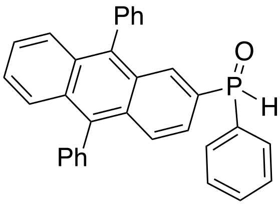

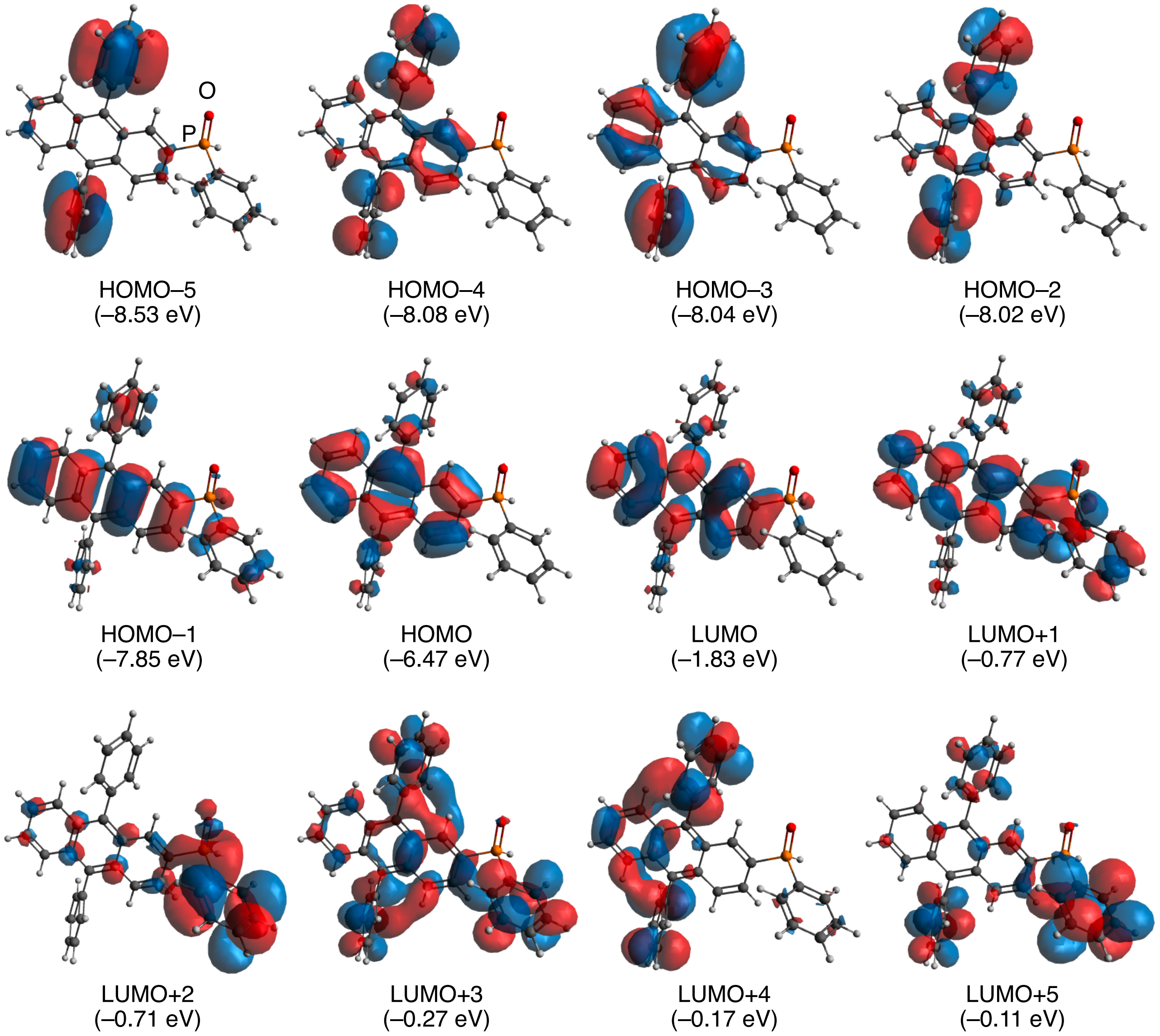

Supplementary Figure 11. Molecular orbitals of DPAsphox using MN15/6-311+G(d,p)//MN15/6-31G(d) in DMF (PCM).

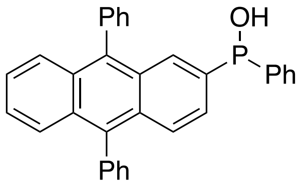

The reduction potential of the excited state of DPAphos (**1'**) was estimated as follows:

$$E_{calc}(\text{DPAphos}^{\bullet+}/\text{DPAphos}^{*}) = E_{calc}(\text{DPAphos}/\text{DPAphos}^{\bullet+}) - E_{0,0}$$

$$= +1.13 \text{ (V vs. SCE)} - 2.88 \text{ (eV)} = -1.75 \text{ (V vs. SCE)}$$

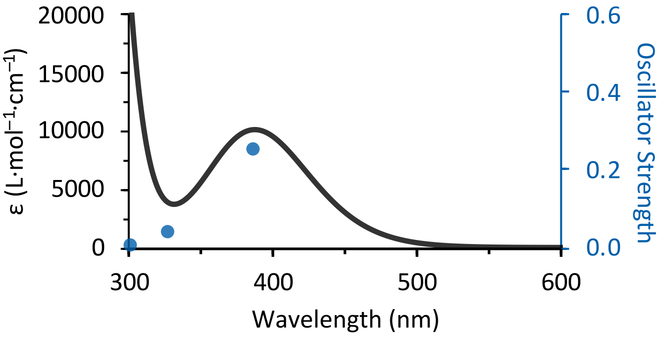

Supplementary Figure 12. Calculated UV-Vis absorption spectra and oscillator strength of DPAphos using MN15/6-311+G(d,p)//MN15/6-31G(d) in DMF (PCM).

Supplementary Table 2. Output of TD-DFT calculation of DPAphos using MN15/6-311+G(d,p)//MN15/6-31G(d) in DMF (PCM).

excited state	wavelength (nm)	oscillator strength	major contribution of molecular orbitals
1	387.66	0.2459	HOMO→LUMO (99%)
2	327.11	0.0330	HOMO–2→LUMO (35%)<br>HOMO→LUMO+1 (60%)
3	302.00	0.0027	HOMO–1→LUMO (86%)
4	269.38	1.5612	HOMO–2→LUMO (42%)<br>HOMO→LUMO+1 (26%)<br>HOMO→LUMO+2 (13%)
5	267.40	0.5408	HOMO–2→LUMO (14%)<br>HOMO→LUMO+2 (44%)<br>HOMO→LUMO+3 (19%)
6	264.57	0.0935	HOMO–3→LUMO (14%)<br>HOMO→LUMO+4 (73%)
7	263.19	0.0048	HOMO–5→LUMO (25%)<br>HOMO–4→LUMO (11%)<br>HOMO–3→LUMO (42%)<br>HOMO→LUMO+4 (17%)
8	260.04	0.0348	HOMO–4→LUMO (54%)<br>HOMO–3→LUMO (30%)
9	257.75	0.0008	HOMO–8→LUMO (13%)<br>HOMO–5→LUMO (51%)<br>HOMO–4→LUMO (20%)
10	255.36	0.0401	HOMO→LUMO+5 (80%)

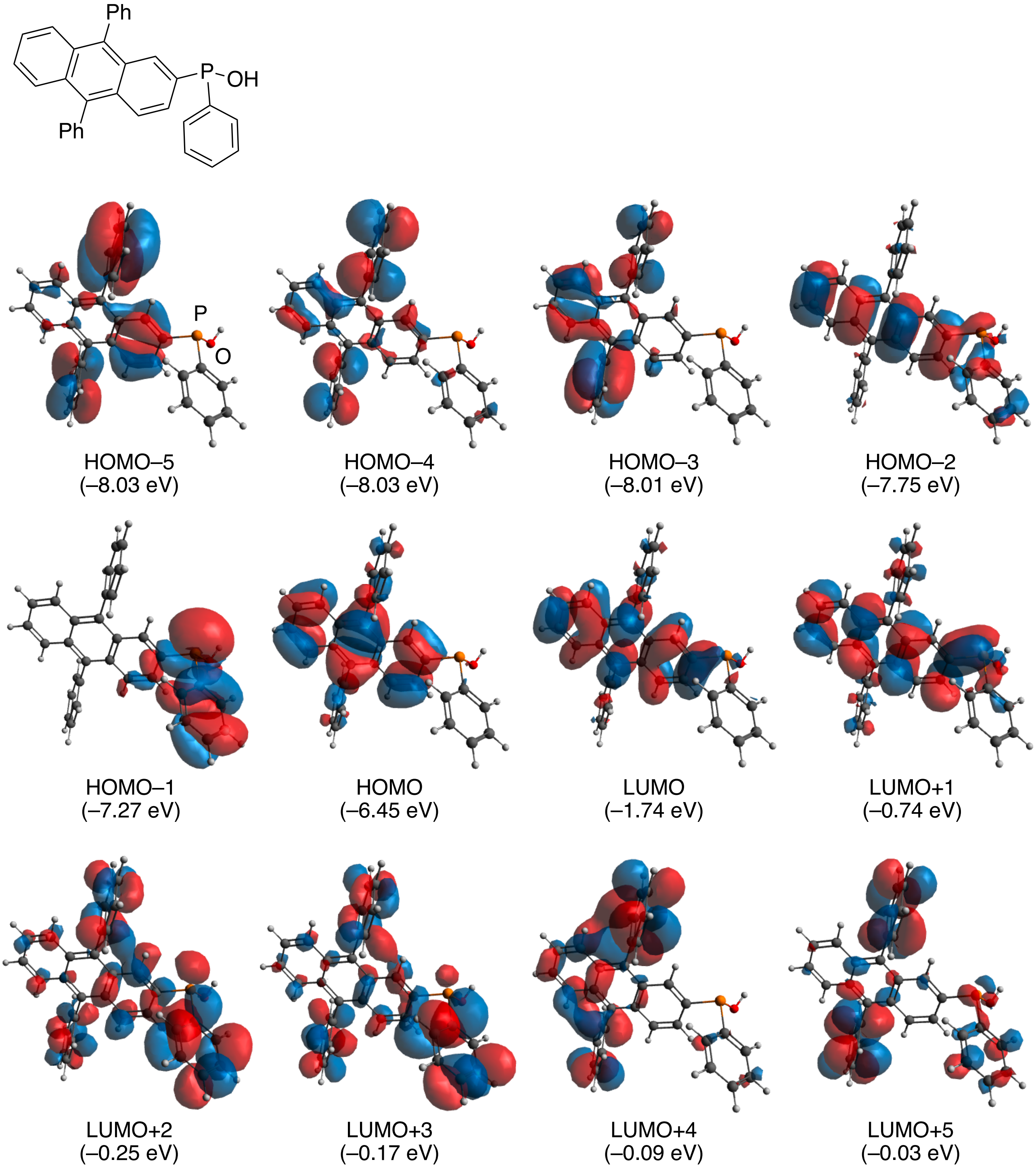

Supplementary Figure 13. Molecular orbitals of DPAphos using MN15/6-311+G(d,p)//MN15/6-31G(d) in DMF (PCM).

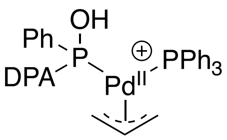

Single-point energy calculation of Pd(II) complex **2** was performed with ωB97XD and CAM-B3LYP functionals. The computational results using ωB97XD functional were in accordance with CAM-B3LYP functional.

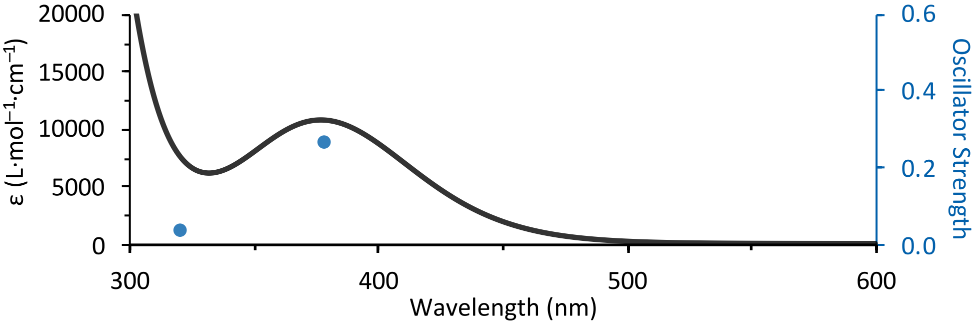

Supplementary Figure 14. Calculated UV-Vis absorption spectra and oscillator strength of Pd(II) complex **2** using ωB97XD/SDD, 6-311+G(d,p)//MN15/SDD, 6-31G(d) in DMF (PCM).

Supplementary Table 3. Output of TD-DFT calculation of Pd(II) complex **2** using ωB97XD/SDD, 6-311+G(d,p)//MN15/SDD, 6-31G(d) in DMF (PCM).

excited state	wavelength (nm)	oscillator strength	major contribution of molecular orbital
1	378.33	0.2603	HOMO→LUMO (96%)
2	320.77	0.0338	HOMO–2→LUMO (22%)<br>HOMO→LUMO+2 (24%)<br>HOMO→LUMO+4 (19%)
3	299.57	0.0985	HOMO–15→LUMO+1 (20%)<br>HOMO–12→LUMO+1 (10%)<br>HOMO–9→LUMO+1 (10%)<br>HOMO–2→LUMO+1 (15%)
4	268.82	1.8372	HOMO–2→LUMO (29%)<br>HOMO–1→LUMO (18%)<br>HOMO→LUMO+1 (10%)<br>HOMO→LUMO+2 (18%)
5	259.95	0.0469	HOMO–17→LUMO+1 (22%)<br>HOMO–15→LUMO+1 (17%)
6	256.46	0.2086	HOMO→LUMO+2 (9%)<br>HOMO→LUMO+6 (10%)<br>HOMO→LUMO+7 (11%)
7	255.00	0.0217	HOMO–18→LUMO+1 (62%)
8	253.10	0.2360	HOMO–19→LUMO+1 (14%)<br>HOMO–1→LUMO+1 (27%)<br>HOMO→LUMO+1 (10%)
9	246.90	0.1771	HOMO–9→LUMO+1 (17%)<br>HOMO–6→LUMO (14%)<br>HOMO–4→LUMO (9%)<br>HOMO→LUMO+9 (9%)<br>HOMO→LUMO+10 (9%)
10	245.58	0.1532	HOMO–9→LUMO+1 (18%)<br>HOMO–6→LUMO (19%)

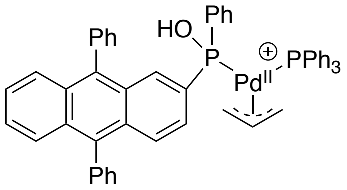

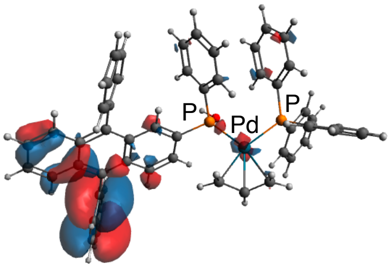

HOMO–5
(–9.22 eV)

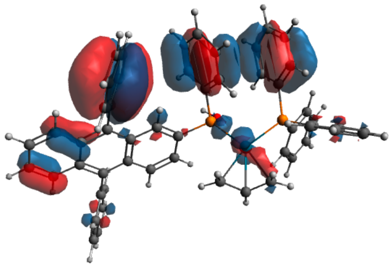

HOMO–4
(–9.19 eV)

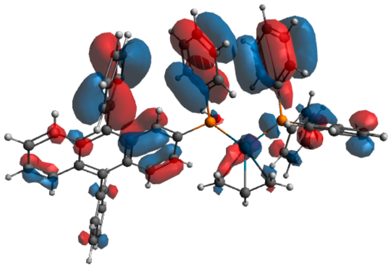

HOMO–3
(–9.17 eV)

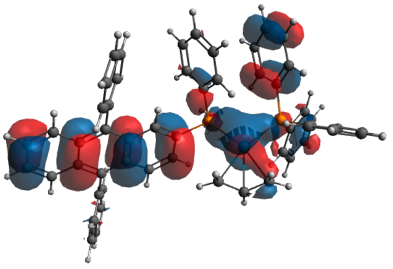

HOMO–2
(–8.91 eV)

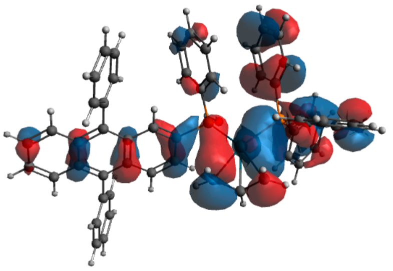

HOMO–1
(–8.61 eV)

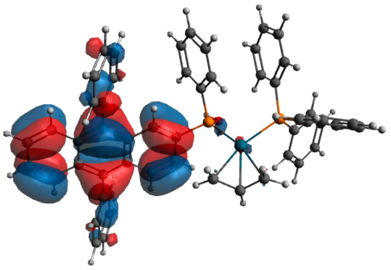

HOMO
(–7.51 eV)

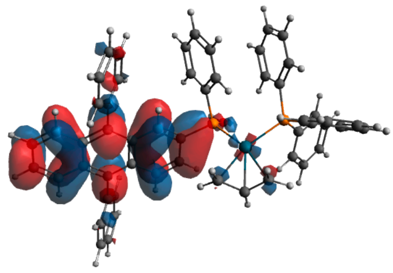

LUMO
(–0.84 eV)

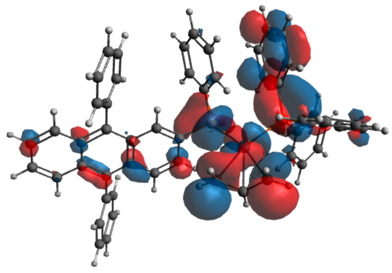

LUMO+1
(–0.11 eV)

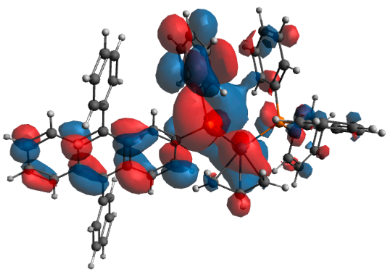

LUMO+2
(0.06 eV)

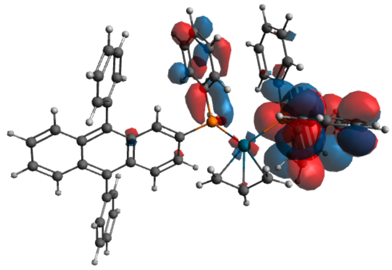

LUMO+3
(0.36 eV)

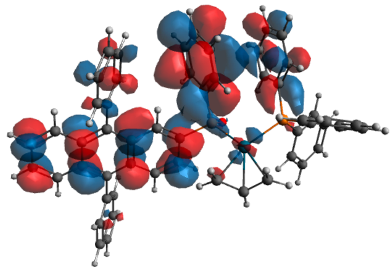

LUMO+4
(0.47 eV)

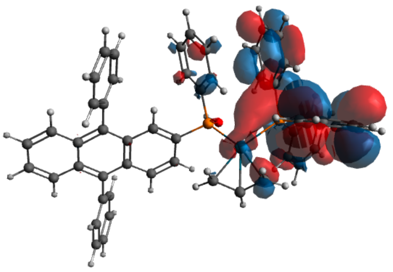

LUMO+5
(0.59 eV)

Supplementary Figure 15. Molecular orbitals of Pd(II) complex **2** using ωB97XD/SDD, 6-311+G(d,p)//MN15/SDD, 6-31G(d) in DMF (PCM).

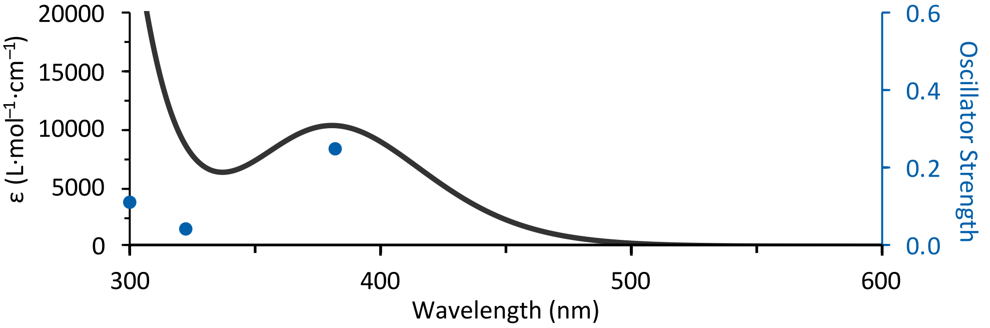

Supplementary Figure 16. Calculated UV-Vis absorption spectra and oscillator strength of Pd(II) complex **2** using CAM-B3LYP/SDD, 6-311+G(d,p)//MN15/SDD, 6-31G(d) in DMF (PCM).

Supplementary Table 4. Output of TD-DFT calculation of Pd(II) complex **2** using CAM-B3LYP/SDD, 6-311+G(d,p)//MN15/SDD, 6-31G(d) in DMF (PCM).

excited state	wavelength (nm)	oscillator strength	major contribution of molecular orbitals
1	382.15	0.2509	HOMO→LUMO (97%)
2	323.12	0.0388	HOMO–2→LUMO (22%)<br>HOMO→LUMO+2 (25%)<br>HOMO→LUMO+4 (19%)
3	300.79	0.1094	HOMO–15→LUMO+1 (19%)<br>HOMO–9→LUMO+1 (12%)<br>HOMO–2→LUMO+1 (14%)
4	272.61	1.6382	HOMO–2→LUMO (27%)<br>HOMO–1→LUMO (20%)<br>HOMO→LUMO+1 (13%)<br>HOMO→LUMO+2 (20%)
5	262.87	0.1128	HOMO→LUMO+1 (26%)<br>HOMO→LUMO+2 (25%)
6	259.79	0.1058	HOMO–17→LUMO+1 (22%)<br>HOMO–15→LUMO+1 (18%)
7	258.87	0.0950	HOMO–1→LUMO+1 (12%)<br>HOMO→LUMO+1 (15%)<br>HOMO→LUMO+7 (20%)
8	254.90	0.0072	HOMO–18→LUMO+1 (70%)
9	251.57	0.2887	HOMO–6→LUMO (24%)<br>HOMO–4→LUMO(16%)<br>HOMO→LUMO+9 (15%)<br>HOMO→LUMO+10 (17%)
10	249.70	0.2047	HOMO–9→LUMO+1 (9%)<br>HOMO–6→LUMO (19%)<br>HOMO→LUMO+4 (14%)

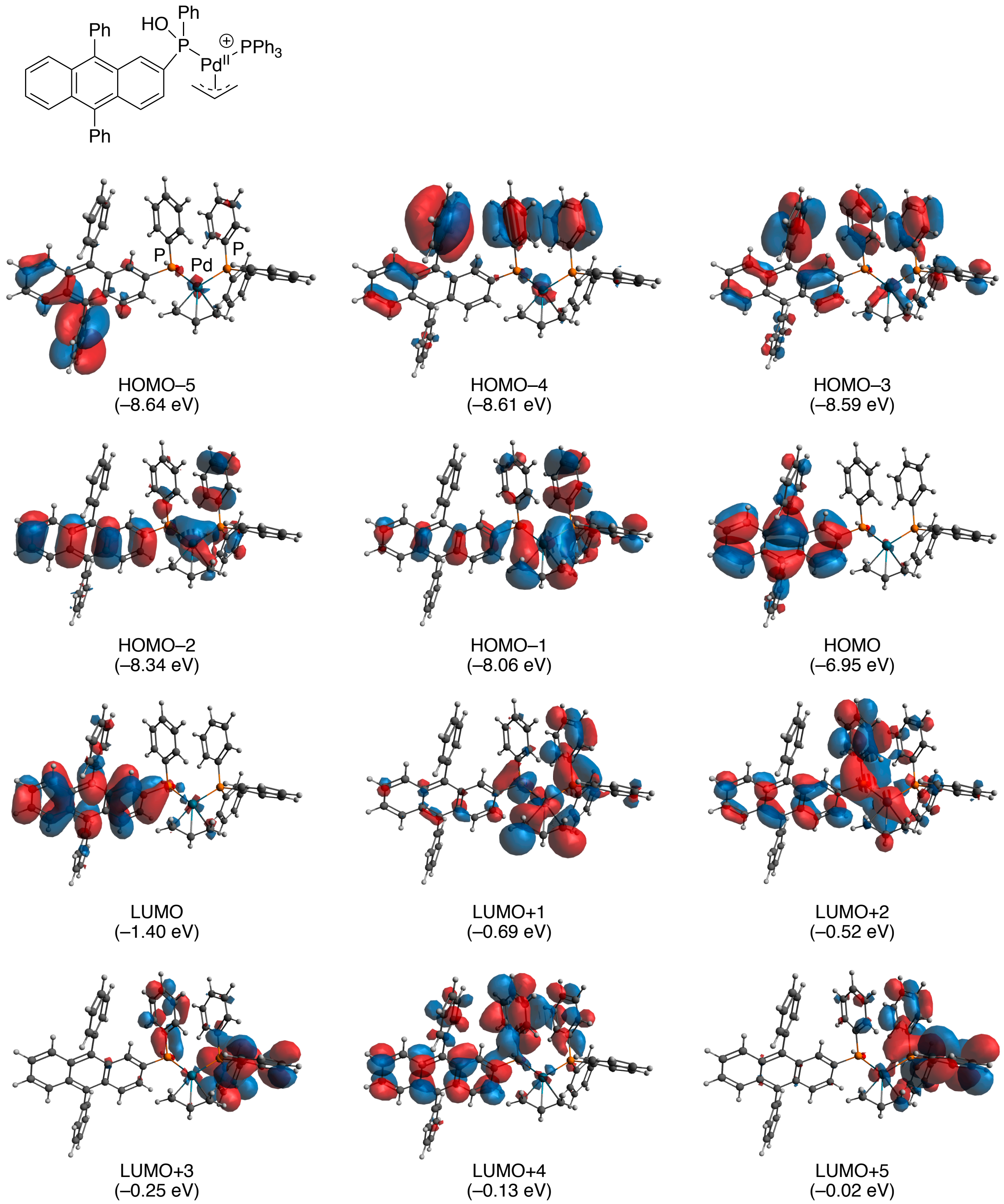

Supplementary Figure 17. Molecular orbitals of Pd(II) complex **2** using CAM-B3LYP/SDD, 6-311+G(d,p)//MN15/SDD, 6-31G(d) in DMF (PCM).

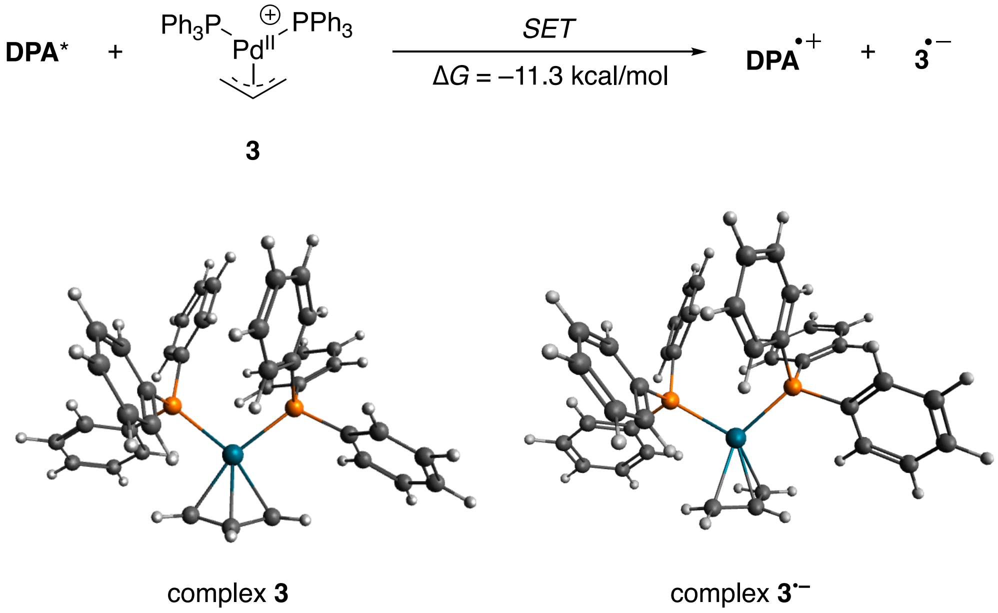

Supplementary Figure 18. The difference in energies and structures through the SET from DPA* to Pd(II) complex **3**.

The intermolecular SET from DPA* in the singlet excited state to Pd(II) complex **3** was thermodynamically favored (ΔG = –11.3 kcal/mol). The 3D structures of **3** and **3**$^{\bullet-}$ were shown in Supplementary Figure 18. TD-DFT calculations were performed with ωB97XD/SDD, 6-311+G(d,p)//MN15/SDD, 6-31G(d) in DMF (PCM).

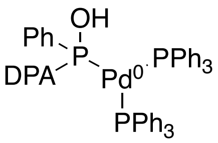

Single-point energy calculation of Pd(0) complex **4** was performed with ωB97XD and CAM-B3LYP functionals. The reduction potentials of Pd(0) complex **4(S$_1$)** in the singlet excited state was estimated as follows:

$$E_{calc}(\mathbf{4}^{\bullet+}/\mathbf{4(S_1)}) = E_{calc}(\mathbf{4}/\mathbf{4}^{\bullet+}) - E_{0,0} = -0.24\ (V) - 2.56\ (eV) = -2.80\ (V)\ (\omega B97XD)$$

$$E_{calc}(\mathbf{4}^{\bullet+}/\mathbf{4(S_1)}) = E_{calc}(\mathbf{4}/\mathbf{4}^{\bullet+}) - E_{0,0} = -0.25\ (V) - 2.60\ (eV) = -2.85\ (V)\ (CAM\text{-}B3LYP)$$

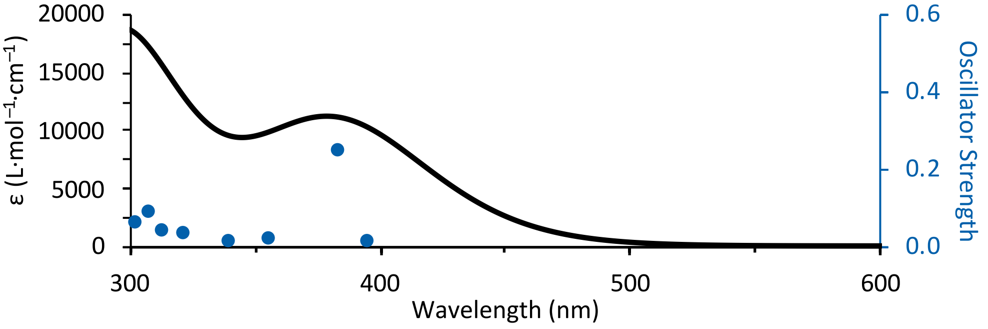

Supplementary Figure 19. Calculated UV-Vis absorption spectra and oscillator strength of Pd(0) complex **4** using ωB97XD/SDD, 6-311+G(d,p)//MN15/SDD, 6-31G(d) in DMA (PCM).

Supplementary Table 5. Output of TD-DFT calculation of Pd(0) complex **4** using ωB97XD/SDD, 6-311+G(d,p)//MN15/SDD, 6-31G(d) in DMA (PCM).

excited state	wavelength (nm)	oscillator strength	major contribution of molecular orbitals
1	395.64	0.0077	HOMO→LUMO (18%)<br>HOMO→LUMO+1 (47%)<br>HOMO→LUMO+2 (9%)
2	383.72	0.2455	HOMO–2→LUMO (58%)<br>HOMO–1→LUMO (37%)
3	356.61	0.0162	HOMO–2→LUMO (12%)<br>HOMO–2→LUMO+1 (6%)<br>HOMO–1→LUMO (7%)<br>HOMO–1→LUMO+1 (38%)<br>HOMO–1→LUMO+2 (7%)
4	340.07	0.0114	HOMO→LUMO (68%)<br>HOMO→LUMO+2 (9%)
5	321.69	0.0318	HOMO–6→LUMO (22%)<br>HOMO–2→LUMO+1 (16%)<br>HOMO–2→LUMO+2 (21%)<br>HOMO–1→LUMO+1 (9%)
6	313.69	0.0393	HOMO–3→LUMO (18%)<br>HOMO–3→LUMO+1 (23%)<br>HOMO–1→LUMO+1 (12%)
7	308.31	0.0877	HOMO–3→LUMO+1 (23%)<br>HOMO–2→LUMO (13%)<br>HOMO–1→LUMO (27%)
8	303.42	0.0575	HOMO→LUMO+3 (71%)
9	293.02	0.1553	HOMO→LUMO+4 (28%)<br>HOMO→LUMO+5 (17%)<br>HOMO→LUMO+6 (30%)
10	284.58	0.1660	HOMO→LUMO+4 (36%)<br>HOMO→LUMO+5 (34%)

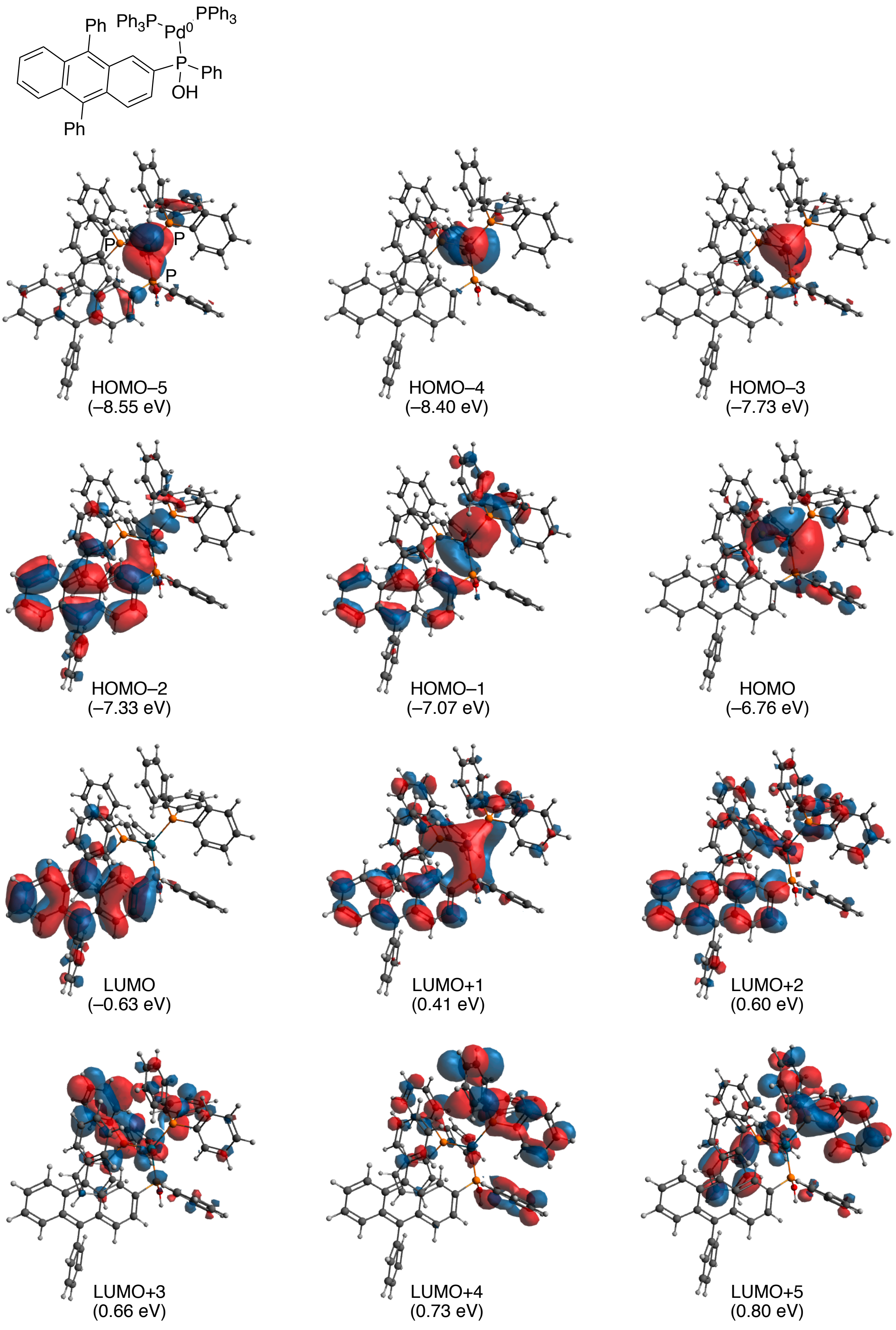

Supplementary Figure 20. Molecular orbitals of Pd(0) complex **4** using ωB97XD/SDD, 6-311+G(d,p)//MN15/SDD, 6-31G(d) in DMA (PCM).

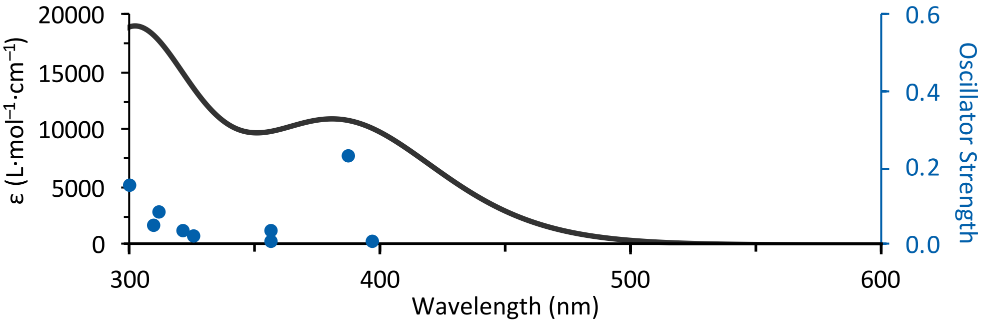

Supplementary Figure 21. Calculated UV-Vis absorption spectra and oscillator strength of Pd(0) complex **4** using CAM-B3LYP/SDD, 6-311+G(d,p)//MN15/SDD, 6-31G(d) in DMA (PCM).

Supplementary Table 6. Output of TD-DFT calculation of Pd(0) complex **4** using CAM-B3LYP/SDD, 6-311+G(d,p)//MN15/SDD, 6-31G(d) in DMA (PCM).

excited state	wavelength (nm)	oscillator strength	major contribution of molecular orbitals
1	397.13	0.0079	HOMO→LUMO (37%)<br>HOMO→LUMO+1 (37%)
2	387.83	0.2312	HOMO–2→LUMO (53%)<br>HOMO–1→LUMO (43%)
3	357.18	0.0034	HOMO–2→LUMO (9%)<br>HOMO–1→LUMO+2 (17%)<br>HOMO→LUMO (28%)
4	356.45	0.0319	HOMO–2→LUMO (12%)<br>HOMO–1→LUMO+1 (13%)<br>HOMO→LUMO (29%)<br>HOMO→LUMO+1 (9%)
5	325.97	0.0168	HOMO–6→LUMO (10%)<br>HOMO–2→LUMO (12%)<br>HOMO–2→LUMO+2 (14%)<br>HOMO–1→LUMO (20%)<br>HOMO–1→LUMO+1 (21%)
6	321.44	0.0375	HOMO–6→LUMO (13%)<br>HOMO–2→LUMO+1 (19%)<br>HOMO–1→LUMO (12%)<br>HOMO–1→LUMO+2 (21%)
7	311.37	0.0848	HOMO–3→LUMO (26%)<br>HOMO–3→LUMO+1 (38%)
8	310.15	0.0511	HOMO→LUMO+3 (76%)
9	300.02	0.1505	HOMO→LUMO+2 (9%)<br>HOMO→LUMO+4 (32%)<br>HOMO→LUMO+5 (10%)<br>HOMO→LUMO+6 (33%)
10	291.66	0.1815	HOMO→LUMO+4 (39%)<br>HOMO→LUMO+5 (38%)

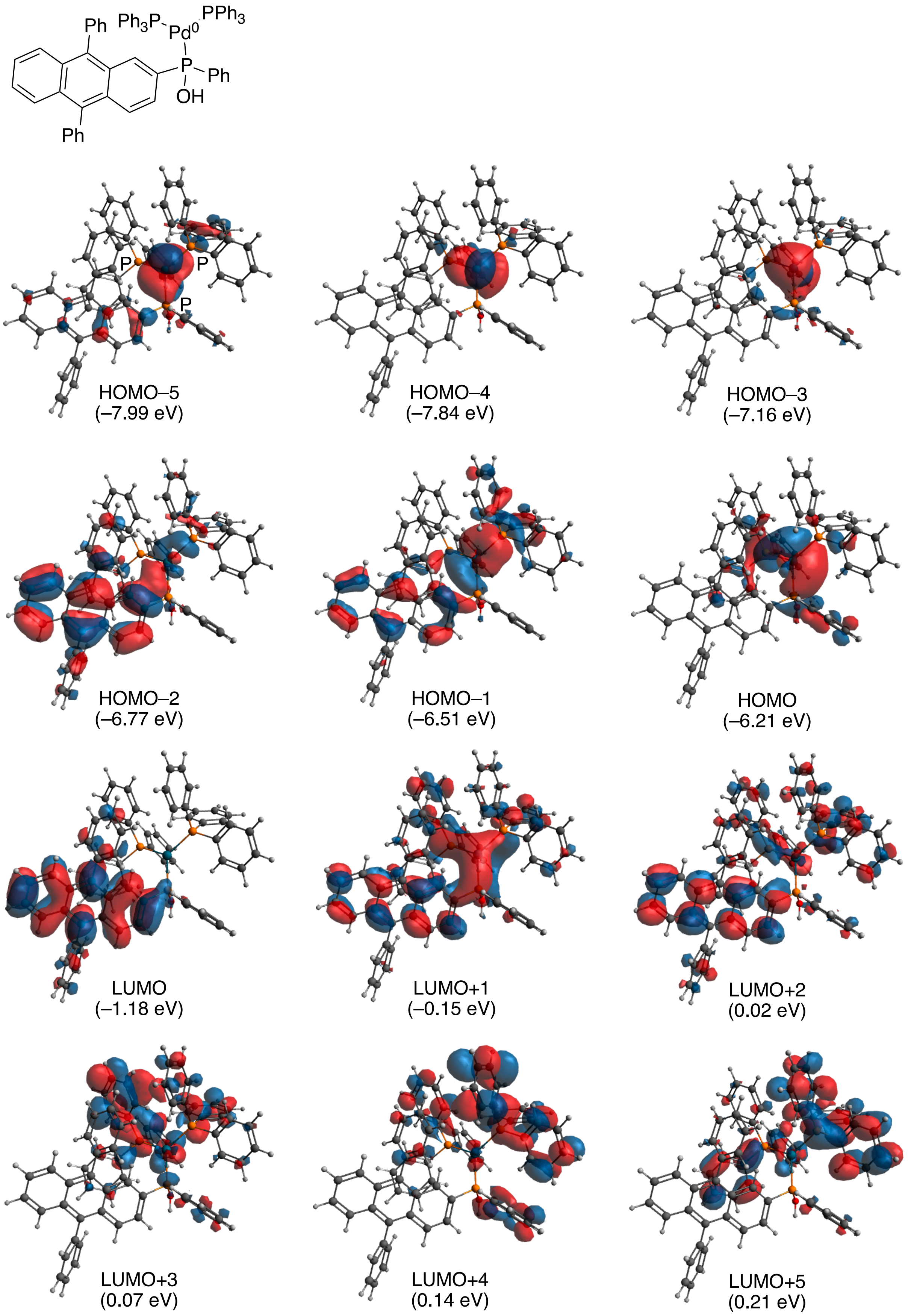

Supplementary Figure 22. Molecular orbitals of Pd(0) complex **4** using CAM-B3LYP/SDD, 6-311+G(d,p)//MN15/SDD, 6-31G(d) in DMA (PCM).

1-5. Synthesis of DPAsphox

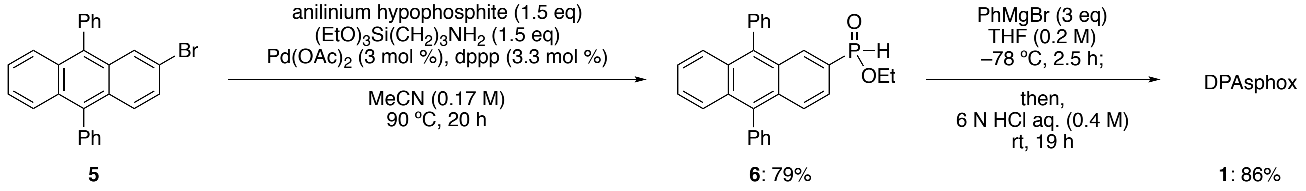

Ethyl (9,10-Diphenylanthracen-2-yl)phosphinate (6)

An 100 mL Shlenk tube containing a magnetic stirring bar was charged with 2-bromo-9,10-diphenylanthracene **5** (1.23 g, 3.0 mmol, 1.0 equiv), anilinium hypophosphite (716 mg, 4.5 mmol, 1.5 equiv), (3-aminopropyl)triethoxysilane (1.06 mL, 4.5 mmol, 1.5 equiv), $Pd(OAc)_2$ (20.2 mg, 0.090 mmol, 3 mol %), dppp (40.8 mg, 0.099 mmol, 3.3 mol %), and MeCN (18.0 mL, 0.17 M), and purged with argon. After stirring for 20 h at 90 ºC, the reaction mixture was evaporated and EtOAc (20 mL) was added. The organic layer was washed with water (3 mL), 1 N HCl aq. (3 mL), sat. $NaHCO_3$ aq. (3 mL), and brine (3 mL), dried over Na_2SO_4, and concentrated. The crude product was purified by flash column chromatography (*n*-hexane/EtOAc = 2/1 to 1/1) to afford **6** (998.2 mg, 2.36 mmol) in 79% yield as yellow amorphous.

^{1}H NMR ($CDCl_3$, 399.8 MHz): δ 8.25 (d, J = 17.4 Hz, 1H), 7.82 (dd, J = 9.2, 3.2 Hz, 1H), 7.75-7.71 (m, 2H), 7.64-7.52 (m, 7H), 7.58 (d, J = 563.7 Hz, 1H), 7.48-7.44 (m, 4H), 7.41-7.34 (m, 2H), 4.18-4.05 (m, 2H), 1.31 (t, J = 6.9 Hz, 3H); ^{13}C{^{1}H} NMR ($CDCl_3$, 100.5 MHz): δ 139.2, 138.2, 137.7, 137.4, 133.2 (d, J = 13.4 Hz), 131.3, 131.14, 131.07, 130.6, 130.4, 128.6, 128.5, 128.4 (d, J = 16.3 Hz), 128.0, 128.0 (d, J = 12.5 Hz), 127.7, 127.3, 126.9, 126.3, 126.1 (d, J = 132.3 Hz), 125.7, 123.0 (d, J = 11.5 Hz), 61.9 (d, J = 5.8 Hz), 16.2 (d, J = 6.7 Hz); ^{31}P NMR ($CDCl_3$, 161.8 MHz): 26.3 (d, J = 575.5 Hz); IR (ν, cm^{-1}): 1389, 1217, 1040, 979, 940, 744, 700, 665, 633, 613; HRMS (ESI) m/z: $[M + Na]^+$ calcd for $C_{28}H_{23}NaO_2P$, 445.1333; found, 445.1331.

(9,10-Diphenylanthracen-2-yl)(phenyl)phosphine Oxide (DPAsphox (1))

An oven-dried 100 mL Shlenk tube containing a magnetic stirring bar was charged with **6** (998.2 mg, 2.36 mmol, 1.0 eq) and THF (11.8 mL, 0.20 M) under argon. To the cooling solution at –78 ºC, 1 M PhMgBr in THF (7.1 mL, 3.0 equiv) was added dropwise. After sttiring for 2.5 h at the same temperature, the reaction was quenched with 6 N HCl aq. (5.9 mL, 0.40 M), and stirred for 19 h at room temperature. Then, the organic layer was extracted with EtOAc (10 mL × 3), washed with brine (3 mL), dried over Na_2SO_4, and concentrated. The crude product was purified by flash column chromatography (*n*-hexane/EtOAc = 1/1 to 1/4) to afford **1** (920.9 mg, 2.03 mmol) in 86% yield as yellow amorphous.

^{1}H NMR ($CDCl_3$, 399.8 MHz): δ 8.63 (one-half of doublet, 0.5H), 8.17 (d, J = 17.9 Hz, 1H), 7.78 (dd,

J = 9.2, 2.8 Hz, 1H), 7.75-7.70 (m, 2H), 7.66-7.61 (m, 2H), 7.59-7.32 (m, 16.5H); $^{13}C\{^{1}H\}$ NMR ($CDCl_3$, 100.5 MHz): δ 139.0, 138.1, 137.6, 137.4, 132.9 (d, *J* = 12.5 Hz), 132.3 (d, *J* = 2.9 Hz), 131.3, 131.13, 131.06, 131.0 (d, *J* = 2.9 Hz), 130.9 (d, *J* = 103.8 Hz), 130.6 (d, *J* = 10.5 Hz), 130.43, 130.38, 128.7 (d, *J* = 12.5 Hz), 128.53, 128.47, 128.43, 128.40 (d, *J* = 13.4 Hz), 128.1 (d, *J* = 11.5 Hz), 127.9, 127.72 (d, *J* = 102.6 Hz), 127.70, 127.2, 126.9, 126.2, 125.7, 123.0 (d, *J* = 11.5 Hz); ^{31}P NMR ($CDCl_3$, 161.8 MHz): 23.2 (d, *J* = 476.8 Hz); IR (ν, cm^{-1}): 1438, 1389, 1194, 1113, 939, 907, 763, 747, 728, 701, 672; HRMS (ESI) m/z: $[M + Na]^+$ calcd for $C_{32}H_{23}NaOP$, 477.1384; found, 477.1357.

1-6. Voltammetric study

Tetrabutylammonium perchlorate (341.9 mg, 1.0 mmol) and DPAsphox or DPA (0.020 mmol) were dissolved in dry MeCN (10 mL), and the solution was degassed by argon bubbling for 5 min. The cyclic voltammograms were measured at 0.5 V/s scan rate using platinum for working and counter electrode and Ag/Ag^{+} for reference electrode. Ferrocene (+0.38 V[11] vs. SCE) was used as an internal standard. The corrected cyclic voltammograms (vs. SCE) were shown.

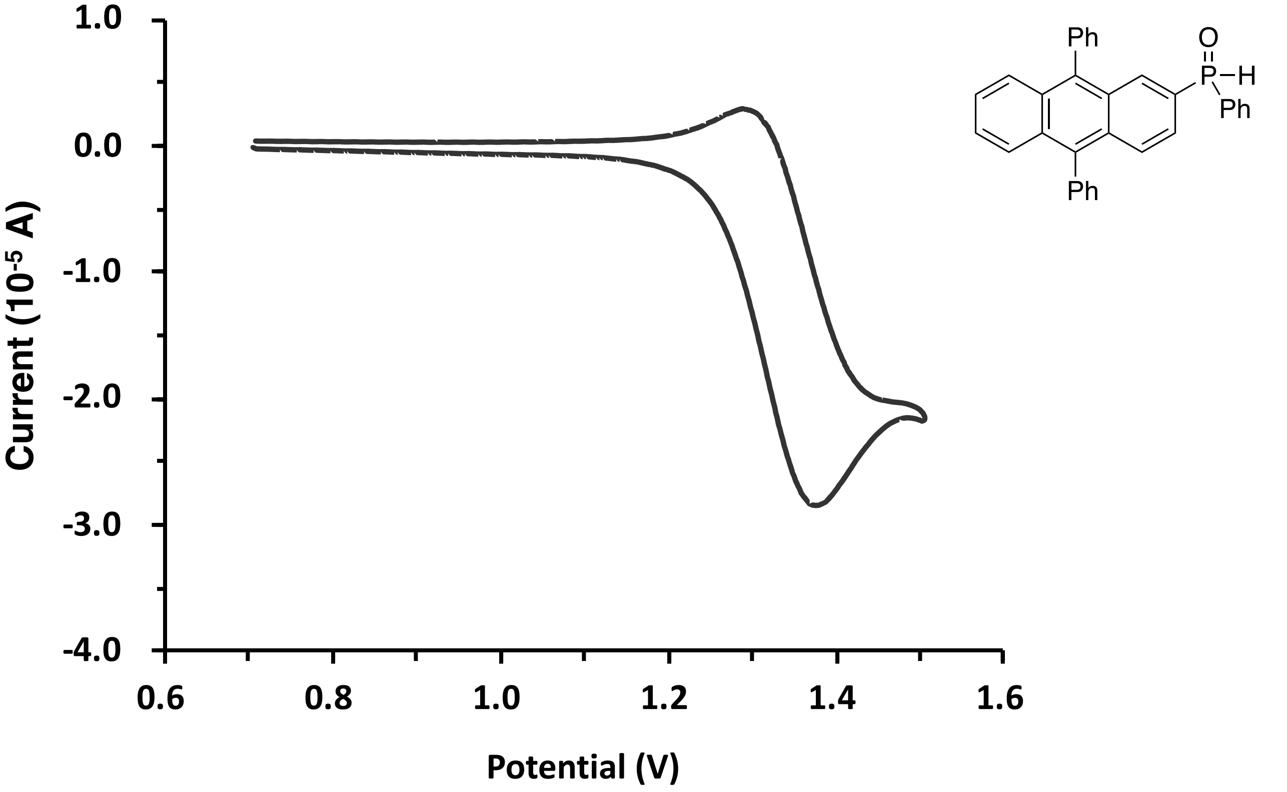

Supplementary Figure 23. The cyclic voltammogram of DPAsphox. The half-wave potential ($E_{1/2}$) is +1.33 V (vs. SCE).

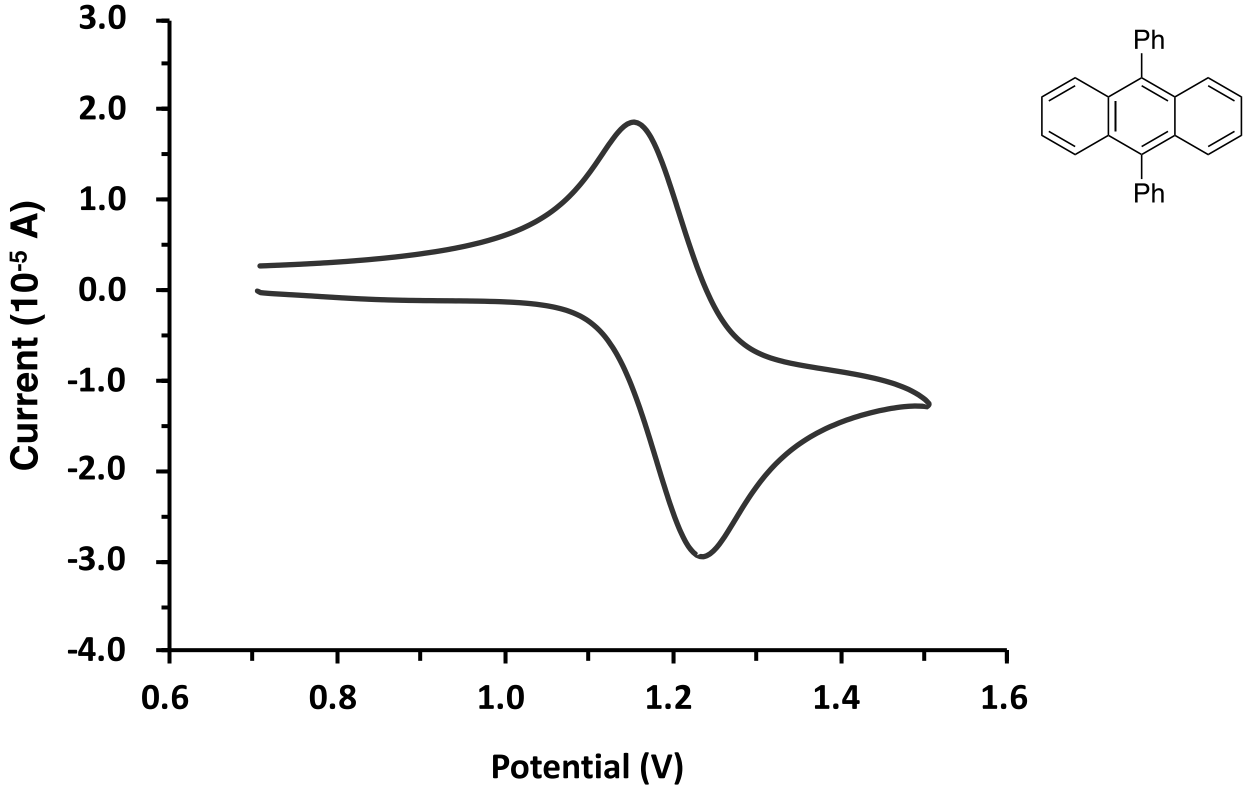

Supplementary Figure 24. The cyclic voltammogram of DPA. The half-wave potential ($E_{1/2}$) is +1.19 V (vs. SCE).

1-7. Calculation of the redox potential of excited state

To estimate the reduction potential of photocatalyst (PC) in the excited state, the Rehm-Weller formalism was used.[9]

$$E_{1/2}(\mathrm{PC}^{\bullet+}/\mathrm{PC}^{*}) = E_{1/2}(\mathrm{PC}/\mathrm{PC}^{\bullet+}) - E_{0,0}$$

$E_{1/2}(\mathrm{PC}/\mathrm{PC}^{\bullet+})$ is the half-wave potential of PC determined by the cyclic voltammogram. $E_{0,0}$ is the first singlet excited state energy estimated from the maximum fluorescence.[12]

$$E_{1/2}(\mathrm{DPAsphox}^{\bullet+}/\mathrm{DPAsphox}^{*}) = E_{1/2}(\mathrm{DPAsphox}/\mathrm{DPAsphox}^{\bullet+}) - E_{0,0} = +1.33 - 2.85 = -1.52\ (\mathrm{V})$$

$$E_{1/2}(\mathrm{DPA}^{\bullet+}/\mathrm{DPA}^{*}) = E_{1/2}(\mathrm{DPA}/\mathrm{DPA}^{\bullet+}) - E_{0,0} = +1.19 - 2.87 = -1.68\ (\mathrm{V})$$

The oxidation potential of DPA in the excited state was also estimated as follows:

$$E_{1/2}(\mathrm{DPA}^{\bullet-}/\mathrm{DPA}^{*}) = E_{1/2}(\mathrm{DPA}/\mathrm{DPA}^{\bullet-}) + E_{0,0} = -1.94^{13} + 2.87 = +0.93\ (\mathrm{V})$$

1-8. Stability test of DPAsphox

Supplementary Table 7. Stability test of DPAsphox after a week.

entry	temp.	atmosphere	light	purity (%)
1	−15 ºC	Ar	shielding condition	99
2	4 ºC	Ar	shielding condition	99
3	rt	Ar	shielding condition	96
4	rt	air	shielding condition	93
5	rt	Ar	on the bench	83
6	rt	air	on the bench	71

The purity of DPAsphox after a week under various conditions was determined by ^{1}H NMR analysis using 1,3,5-trimethoxybenzene as an internal standard. The initial purity of DPAsphox was 99% determined by the same method.

To demonstrate the relative stability of DPAsphox, tertiary phosphine **S4** was synthesized according to the following modified procedure.[14]

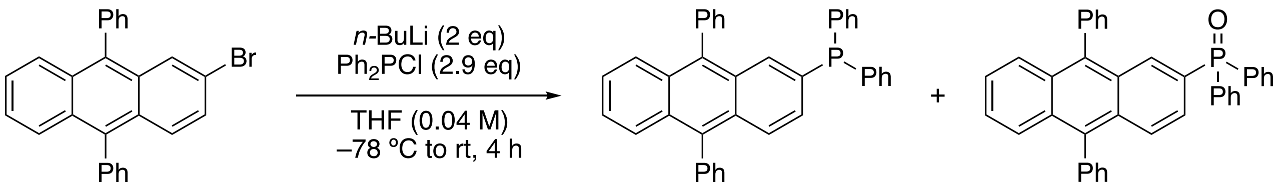

5 **S4** **S5**

(9,10-diphenylanthracen-2-yl)diphenylphosphane (S4)

An oven-dried 20 mL Shlenk tube containing a magnetic stirring bar was charged with **5** (204.7 mg, 0.50 mmol, 1.0 eq) and THF (12.5 mL, 0.040 M) under argon. To the cooling solution at –78 ºC, *n*-BuLi in cyclohexane (2 M, 0.50 mL, 2.0 equiv) was added dropwise. After stirring the resulting red solution for 1 h at the same temperature, diphenylphosphine chloride (266.6. μL, 2.9 equiv) was added dropwise, and the reacton was stirred for 3 h at room temperature. Then, the reaction was quenched with water (3.0 mL), and the organic layer was extracted with DCM (3 mL × 3), washed with brine (3 mL), dried over Na_2SO_4, and concentrated. The crude product was roughly purified by flash column chromatography (*n*-hexane/EtOAc = 1/0 to 1/1). Phoshine oxide **S5**, which is oxidized under air, was obtained in 26% yield determined by ^{31}P NMR using PPh_3 as an internal standard.[14] Tertiary phosphine **S4** was partially purified by preparative TLC (*n*-hexane/EtOAc = 100/1).

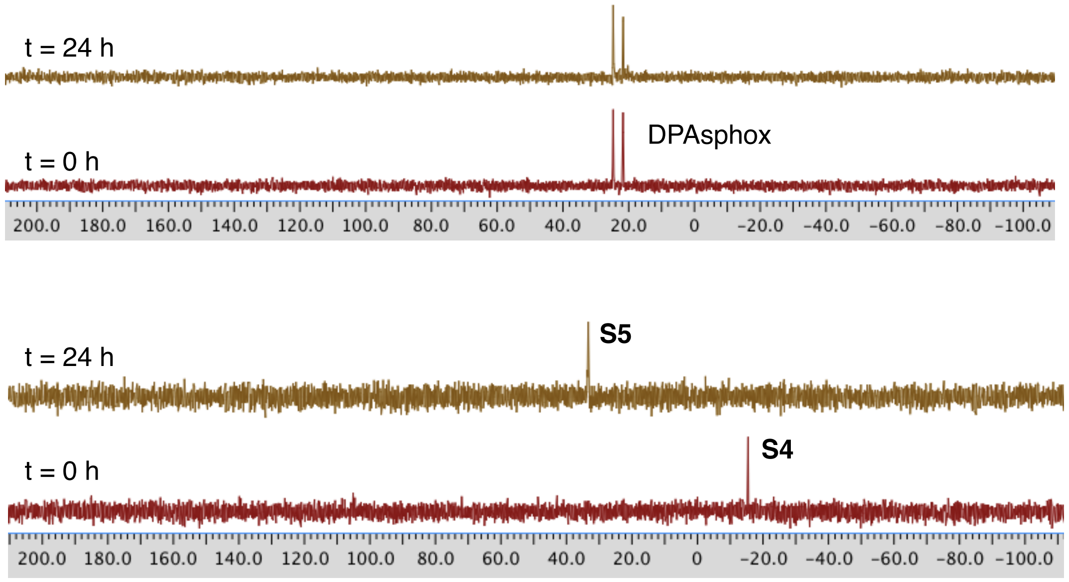

Supplementary Figure 25. ^{31}P NMR spectra of DPAsphox and **S4** in $CDCl_3$.

The ^{31}P NMR spectra of DPAsphox and **S4** were measured at 0 and 24 h in $CDCl_3$. The NMR tubes were kept on the bench.

1-9. ^{31}P NMR study

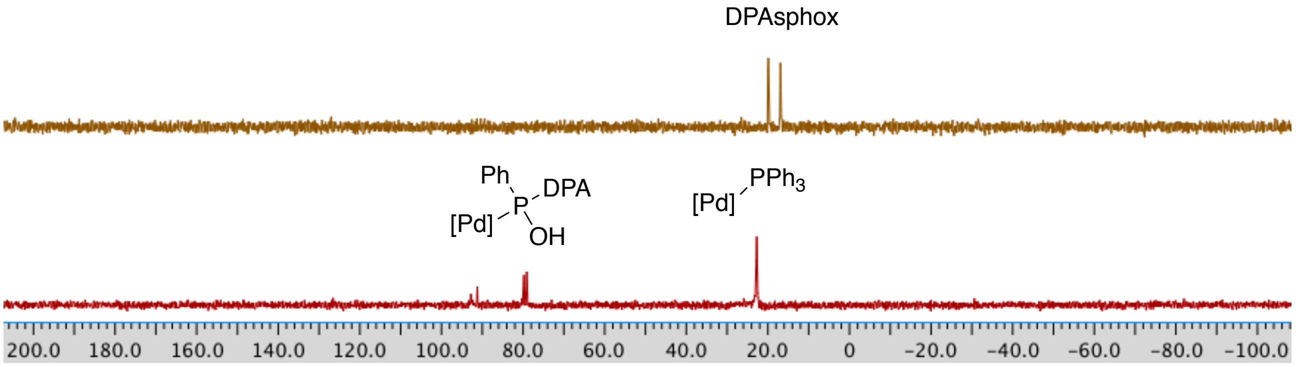

Supplementary Figure 26. ^{31}P NMR spectra of DPAsphox and the mixture of [PdCl(allyl)]$_2$, PPh_3, and DPAsphox in DMF-d_7.

An 20 mL Shlenk tube containing a magnetic stirring bar was charged with [PdCl(allyl)]$_2$ (0.91 mg, 0.0025 mmol, 0.50 equiv), PPh_3 (1.3 mg, 0.0050 mmol, 1.0 equiv), DPAsphox (2.3 mg, 0.0050 mmol, 1.0 equiv), and DMF-d_7 (0.60 mL). After the tube was evacuated and filled with argon, the reaction was stirred for 1 h under light-shielding condition. The mixture was transfered to NMR tube to measure the ^{31}P NMR spectrum. The doublet peak of DPAsphox was disappeared and new peaks between 79.1 and 92.8 ppm were emerged.[15]

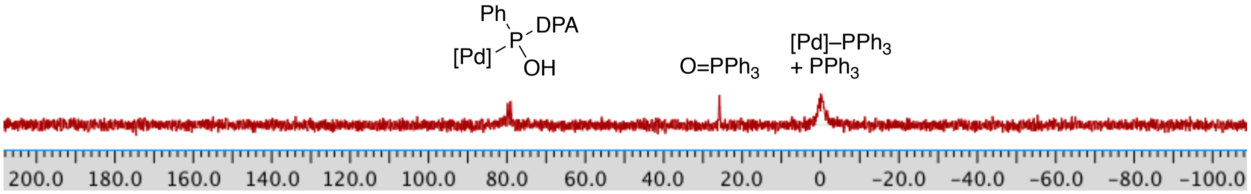

Supplementary Figure 27. A ^{31}P NMR spectrum of the mixture of $Pd(PPh_3)_4$ and DPAsphox.

A NMR tube was charged with $Pd(PPh_3)_4$ (5.8 mg, 0.0050 mmol) and DPAsphox (2.3 mg, 0.0050 mmol), evacuated, filled with argon, and added degassed DMF-d_7 (0.60 mL). A ^{31}P NMR spectrum was measured after 2 h. The doublet peak of DPAsphox was disappeared and new peaks between 79.1 and 79.8 ppm were emerged.[15]

1-10. Optimization of the reaction conditions

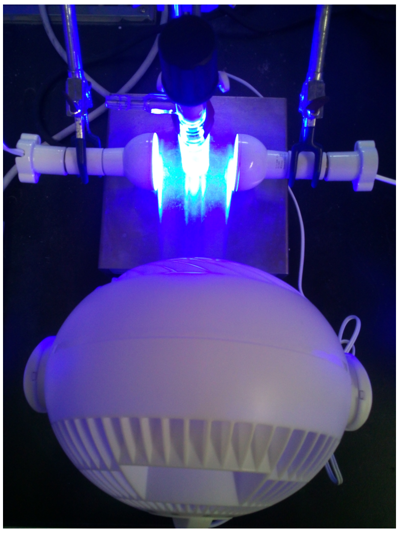

Supplementary Figure 28. Reaction setup.

The reactions were performed with external irradiation lights and air circulator (Supplementary Figure 28). The 5 W blue LED lights were purchased from Hikari Shoji Co., Ltd. (the item stock number: HS0511BD2).

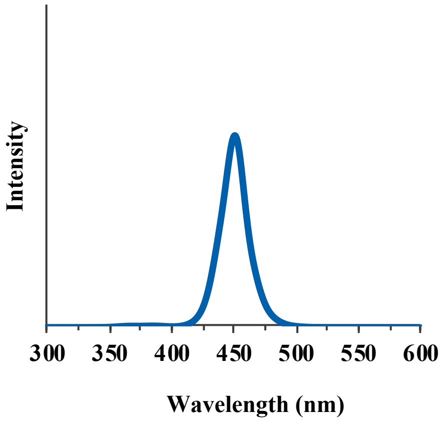

Supplementary Figure 29. The wavelength range of blue LED light.

The wavelength range of the 5 W blue LED light was comfirmed by a JASCO FP-8500 spectrofluorometer (Supplementary Figure 29).

Supplementary Table 8. Optimization of α-allylation of amines.

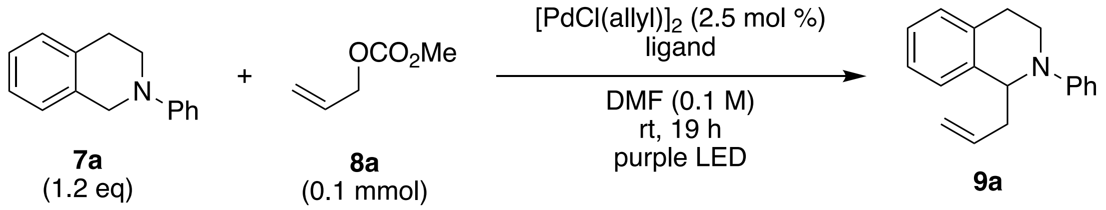

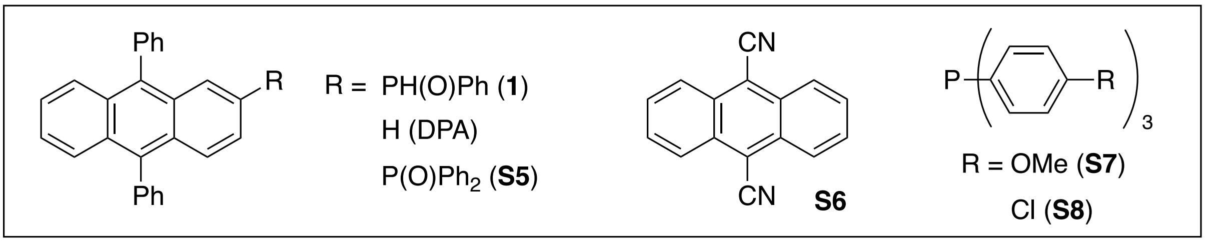

entry	ligand (mol %)	variation from the above conditions	yield (%)[a]
1	PPh_3 (5), **1** (5)	-	75
2	**1** (5)	-	8
3	**1** (10)	-	8
4	PPh_3 (10)	-	trace
5	PPh_3 (10)	addition of DPA (5 mol %)	25
6	PPh_3 (10)	addition of **S5** (5 mol %)	20
7	PPh_3 (10)	addition of **S6** (5 mol %)	8
8	DPEphos (5)	-	n.d.
9	Xantphos (5)	-	n.d.
10	PPh_3 (5), **1** (5)	DMA as solvent	61
11	PPh_3 (5), **1** (5)	MeCN as solvent	44
12	$Ph_2P(O)H$ (5), **1** (5)	-	10
13	**S7** (5), **1** (5)	-	15
14	**S8** (5), **1** (5)	-	73
15	P(*o*-tolyl)$_3$ (5), **1** (5)	-	12
16	P(*p*-tolyl)$_3$ (5), **1** (5)	-	82
17	P(*p*-tolyl)$_3$ (5), **1** (5)	at 4 ºC	77
18	P(*p*-tolyl)$_3$ (5), **1** (5)	without $[PdCl(allyl)]_2$	trace
19	P(*p*-tolyl)$_3$ (5), **1** (5)	under air	n.d.
20	P(*p*-tolyl)$_3$ (5), **1** (5)	under light-shielding condition	n.d.
21	P(*p*-tolyl)$_3$ (5), **1** (5)	using 5 W blue LED × 2	83
22	P(*p*-tolyl)$_3$ (5), **1** (5)	**4a** (1.5 eq), using 5 W blue LED × 2	76
23	P(*p*-tolyl)$_3$ (5), **1** (5)	**4a** (1.0 eq), **5a** (1.5 eq), using 5 W blue LED × 2	97[b]

[a]Yields were determined by ^{1}H NMR using triphenylmethane as an internal standard. [b]Isolated yield. DPA = 9,10-diphenylanthracene; DPEphos = bis[(2-diphenylphosphino)phenyl] ether; Xantphos = 4,5-bis(diphenylphosphino)-9,9-dimethylxanthene.

Supplementary Table 9. Optimization of Heck reaction of unactivated alkyl bromide.

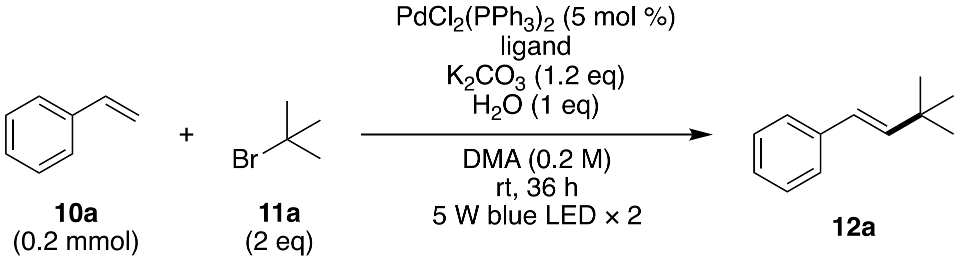

entry	ligand (mol %)	variation from the above conditions	yield (%)[a]
1	PPh_3 (5), **1** (5)	-	93[b]
2	PPh_3 (5), **1** (5)	**11a** (1.5 eq)	82
3	PPh_3 (5), **1** (5)	**11a** (1.5 eq), DMA (0.1 M)	80
4	PPh_3 (5)	-	19
5	PPh_3 (5)	addition of DPA (5 mol %)	31
6	PPh_3 (5)	addition of **S5** (5 mol %)	50
7	**1** (5)	-	12
8	PPh_3 (5), **1** (5)	DMF as solvent	91
9	Xantphos (5)	-	57
10	PPh_3 (5), **1** (5)	without $PdCl_2(PPh_3)_2$	trace
11	PPh_3 (5), **1** (5)	without H_2O	82
12	-	$Pd(PPh_3)_4$ instead of $PdCl_2(PPh_3)_4$	32
13	**1** (5)	$Pd(PPh_3)_4$ instead of $PdCl_2(PPh_3)_4$	79

[a]Yields were determined by ^{1}H NMR yield using dimethyl terephthalate as an internal standard. [b]Isolated yield.

Supplementary Table 10. Optimization of biaryl synthesis.

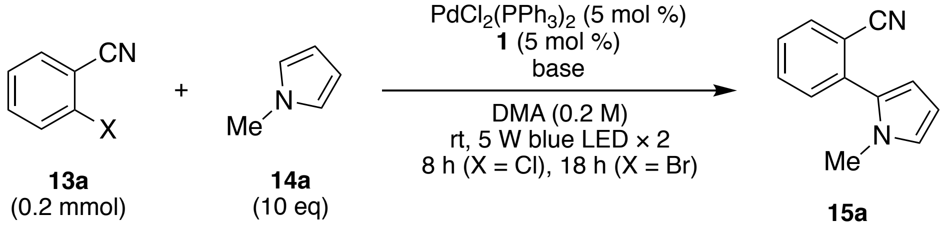

entry	X	base (eq)	variation from the above conditions	yield (%)[a]
1	Cl	K_2CO_3 (1.2)	-	70
2	Cl	K_2CO_3 (1.2)	DMA (0.3 M)	65
3	Cl	K_2CO_3 (1.2)	NMP as solvent	57
4	Cl	K_2CO_3 (1.2)	MeCN as solvent	30
5	Cl	K_2CO_3 (1.2)	DMF as solvent	41
6	Cl	Cs_2CO_3 (1.2)	-	46
7	Cl	Na_2CO_3 (1.2)	-	69
8	Cl	$KHCO_3$ (1.2)	-	67
9	Cl	K_2HPO_4 (1.2)	-	76
10	Cl	K_3PO_4 (1.2)	-	77
11	Cl	K_3PO_4 (2)	-	76
12	Cl	K_3PO_4 (1.2)	without $PdCl_2(PPh_3)_2$	7
13	Cl	K_3PO_4 (1.2)	without **1**	14
14	Cl	K_3PO_4 (1.2)	$Ph_2P(O)H$ instead of **1**	28
15	Cl	K_3PO_4 (1.2)	DPA instead of **1**	7
16	Cl	K_3PO_4 (1.2)	Xantphos instead of **1**	43
17	Cl	-	-	8
18	Cl	K_3PO_4 (1.2)	under light-shielding condition	n.d.
19	Cl	K_3PO_4 (1.2)	using purple LED	16
20	Cl	K_3PO_4 (1.2)	using green LED	trace
21	Cl	K_3PO_4 (1.2)	under air	trace
22	Cl	K_3PO_4 (1.2)	$PdCl_2$ instead of $PdCl_2(PPh_3)_2$	trace
23	Br	K_3PO_4 (1.2)	-	15
24	Br	K_3PO_4 (1.2)	PPh_3 instead of **1**	15
25	Br	K_3PO_4 (1.2)	addition of PPh_3 (5 mol %)	84[b]
26	Cl	K_3PO_4 (1.2)	addition of PPh_3 (5 mol %)	80[b]

[a]Yields were determined by ^{1}H NMR yield using dimethyl terephthalate as an internal standard. [b]Isolated yield.

1-11. Preparation of substrates.

Amine **7**,[16] allyl methyl carbonate **8**,[17] styrene **10e**,[18] styrenes **10f** and **10h**,[19] and 1,3-dimethylindole **14l**[20] were prepared according to the reported procedure.

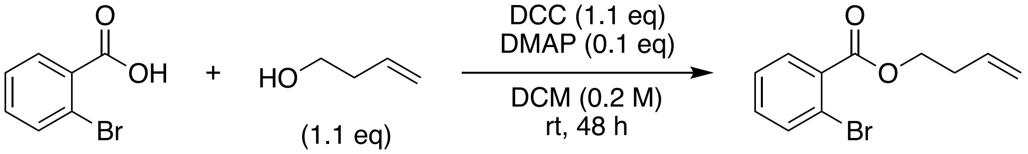

but-3-en-1-yl 2-bromobenzoate (16d)

To a stirred solution of 2-bromobenzoic acid (603.0 mg, 3.0 mmol, 1.0 equiv), 3-buten-1-ol (0.28 mL, 3.3 mmol, 1.1 equiv), and DMAP (36.7 mg, 0.30 mmol, 0.10 equiv) in DCM (15.0 mL, 0.20 M) was added DCC (680.9 mg, 3.3 mmol, 1.1 equiv) at room temperature. After stirring for 48 h, the reaction mixture was filtered through a short pad of celite, and the filtrate was concentrated *in vacuo*. The crude product was purified by flash column chromatography (*n*-hexane/EtOAc = 50/1) to afford **16d** (760.0 mg) in quantitative yield as colorless oil.

^{1}H NMR ($CDCl_3$, 399.8 MHz): δ 7.78 (dd, J = 7.3, 2.3 Hz, 1H), 7.65 (dd, J = 7.3, 1.4 Hz, 1H), 7.38-7.29 (m, 2H), 5.88 (ddt, J = 17.4, 10.5, 6.9 Hz, 2H), 5.18 (ddd, J = 17.4, 1.8, 1.8 Hz, 1H), 5.12 (ddd, J = 10.5, 1.8, 1.4 Hz, 1H), 4.40 (t, J = 6.9 Hz, 2H), 2.56-2.51 (m, 2H); ^{13}C{^{1}H} NMR ($CDCl_3$, 100.5 MHz): δ 165.8, 134.1, 133.7, 132.3, 132.1, 131.1, 126.9, 121.4, 117.3, 64.4, 32.8; IR (ν, cm^{-1}): 1729, 1289, 1246, 1131, 1106, 1043, 1028, 916, 741; HRMS (ESI) m/z: $[M + Na]^+$ calcd for $C_{11}H_{11}BrNaO$, 276.9840; found, 276.9837.

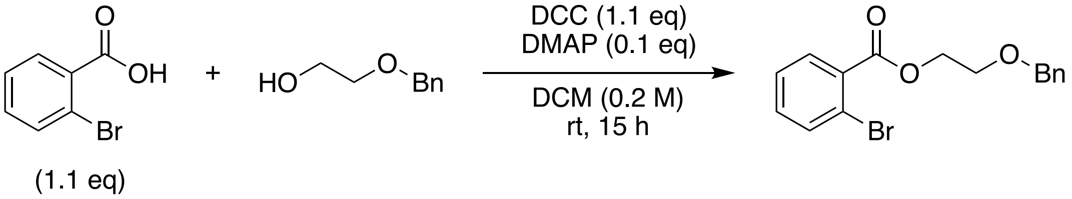

2-(benzyloxy)ethyl 2-bromobenzoate (16e)

To a stirred solution of 2-bromobenzoic acid (639.0 mg, 3.2 mmol, 1.1 equiv), 2-(benzyloxy)ethanol (439.4 mg, 2.89 mmol, 1.0 equiv), DMAP (35.3 mg, 0.29 mmol, 0.10 equiv) in DCM (14.5 mL, 0.20 M) was added DCC (655.9 mg, 3.2 mmol, 1.1 equiv) at room temperature. After stirring for 15 h, the reaction mixture was filtered through a short pad of celite, and the filtrate was concentrated *in vacuo*. The crude product was purified by flash column chromatography (*n*-hexane/EtOAc = 10/1) to afford **16e** (954.2 mg) in quantitative yield as colorless oil.

^{1}H NMR ($CDCl_3$, 399.8 MHz): δ 7.81 (dd, J = 6.9, 2.3 Hz, 1H), 7.66 (dd, J = 7.8, 1.4 Hz, 1H), 7.39-7.28 (m, 7H), 4.61 (s, 2H), 4.52 (t, J = 5.0 Hz, 2H), 3.82 (t, J = 5.0 Hz, 2H); ^{13}C{^{1}H} NMR ($CDCl_3$, 100.5 MHz): δ 166.0, 137.8, 134.2, 132.5, 131.9, 131.4, 128.3, 127.6, 127.0, 121.7, 73.1, 67.7, 64.5;

IR (ν, cm^{-1}): 1729, 1289, 1248, 1098, 1043, 1027, 908, 729, 696, 644; HRMS (ESI) m/z: $[M + Na]^+$ calcd for $C_{16}H_{15}BrNaO_3$, 357.0102; found, 357.0089.

1-12. General procedure

General procedure for α-allylation of amines

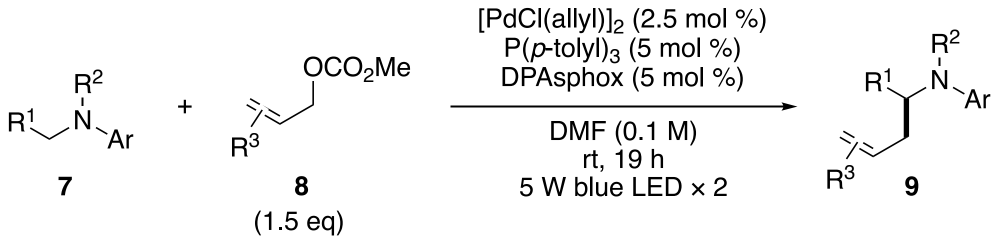

An 20 mL Shlenk tube containing a magnetic stirring bar was charged with amine **7** (0.20 mmol, 1.0 equiv), $[PdCl(allyl)]_2$ (1.8 mg, 2.5 mol %), P(*p*-tolyl)$_3$ (3.0 mg, 5.0 mol %), DPAsphox (4.5 mg, 5.0 mol %), allyl carbonate **8** (0.30 mmol, 1.5 equiv), and DMF (2.0 mL, 0.10 M). After the reaction mixture was degassed by freeze-pump-thaw cycles three times, it was sitrred for 19 h at room temperature under irradiation with 5 W blue LED lights. Then, water (3 mL) was added to the reaction, and the aqueous layer was extracted with EtOAc (3 mL × 3). The combined organic layer was washed with water (3 mL × 3) and brine (3 mL), dried over Na_2SO_4, and concentrated. The crude product was purified by flash column chromatography to afford the corresponding product **9**.

General procedure for Heck reaction of unactivated alkyl bromide

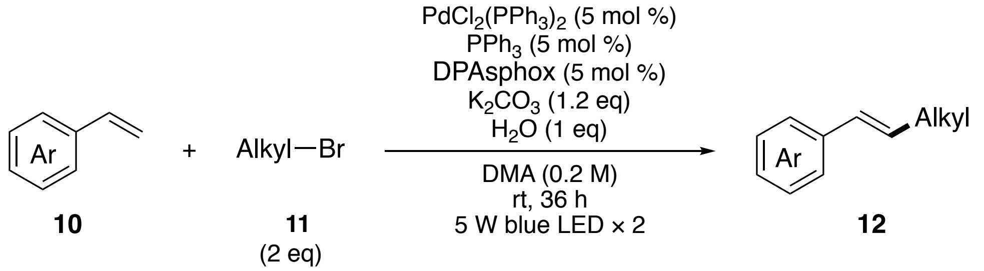

An 20 mL Shlenk tube containing a magnetic stirring bar was charged with $PdCl_2(PPh_3)_2$ (7.0 mg, 5.0 mol %), PPh_3 (2.6 mg, 5.0 mol %), DPAsphox (4.5 mg, 5.0 mol %), and K_2CO_3 (33.2 mg, 1.2 equiv). After the tube was evacuated and filled with argon, styrene **10** (0.20 mmol, 1.0 equiv), alkyl bromide **11** (0.40 mmol, 2.0 equiv), distilled water (3.6 μL, 1.0 equiv), and degassed DMA (1.0 mL, 0.20 M) were added under a stream of argon. The reaction mixture was sitrred for 36 h at room temperature under irradiation with 5 W blue LED lights. Then, water (3 mL) was added to the reaction, and the aqueous layer was extracted with EtOAc (3 mL × 3). The combined organic layer was washed with water (3 mL × 3) and brine (3 mL), dried over Na_2SO_4, and concentrated. The crude product was purified by flash column chromatography to afford the corresponding product **12**.

General procedure for biaryl synthesis

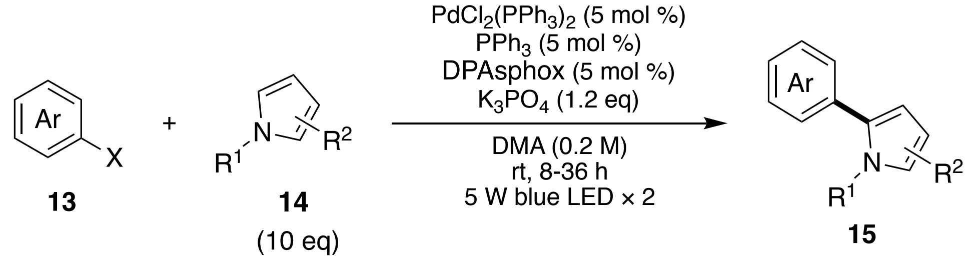

An 20 mL Shlenk tube containing a magnetic stirring bar was charged with $PdCl_2(PPh_3)_2$ (7.0 mg, 5.0 mol %), PPh_3 (2.6 mg, 5.0 mol %), DPAsphox (4.5 mg, 5.0 mol %), K_3PO_4 (50.9 mg, 1.2 equiv), aryl halide **13** (0.20 mmol, 1.0 equiv), pyrrole **14** (2.0 mmol, 10.0 equiv), and DMA (1.0 mL, 0.20 M). After the reaction mixture was degassed by freeze-pump-thaw cycles three times, it was sitrred at room temperature under irradiation with 5 W blue LED lights. Then, water (3 mL) was added to the reaction, and the aqueous layer was extracted with EtOAc (3 mL × 3). The combined organic layer was washed with water (3 mL × 3) and brine (3 mL), dried over Na_2SO_4, and concentrated. The crude product was purified by flash column chromatography to afford the corresponding product **15**.

General procedure for dehalogative hydrogenation

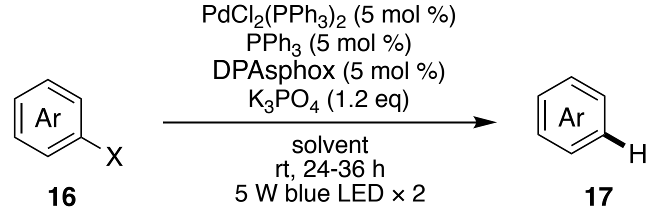

An 20 mL Shlenk tube containing a magnetic stirring bar was charged with $PdCl_2(PPh_3)_2$ (7.0 mg, 5.0 mol %), PPh_3 (2.6 mg, 5.0 mol %), DPAsphox (4.5 mg, 5.0 mol %), K_3PO_4 (50.9 mg, 1.2 equiv), aryl halide **16** (0.20 mmol, 1.0 equiv), and solvent as specified. After the reaction mixture was degassed by freeze-pump-thaw cycles three times, it was sitrred at room temperature under irradiation with 5 W blue LED lights. Then, water (3 mL) was added to the reaction, and the aqueous layer was extracted with EtOAc (3 mL × 3). The combined organic layer was washed with water (3 mL × 3) and brine (3 mL), dried over Na_2SO_4, and concentrated. The crude product was purified by flash column chromatography to afford the corresponding product **17**.

1-13. Characterization of reaction products

Spectral data of the reported compounds were matched with the literature data.

References: (**9a**, **q**, **r**)[21];(**9c**, **d**, **f-h**)[22]; **9m**[23]; (**12a**, **f**)[24]; (**12b**, **n**)[25]; (**12c**, **d**, **i**, **q**)[26]; **12g**[27]; **12h**[28]; **12j**[29]; (**12k**, **o**, **p**)[30]; (**12l**, **m**)[31]; **12r**[32]; (**15a**, **b**, **d**, **h-j**)[33]; **15f**[34]; **15g**[35]; **15k**[36]; **17d**[37]; **17e**[38].

New substrates were characterized as follows:

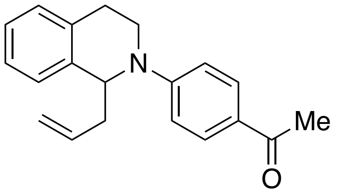

1-(4-(1-allyl-3,4-dihydroisoquinolin-2(1H)-yl)phenyl)ethan-1-one (9b)

The crude product was purified by preparative TLC (DCM/Et_3N = 100/1) to afford **9b** (44.7 mg) in 77% yield as light yellow oil.

^{1}H NMR ($CDCl_3$, 399.8 MHz): δ 7.89 (d, *J* = 9.2 Hz, 2H), 7.20-7.11 (m, 4H), 6.83 (d, *J* = 9.2 Hz, 2H), 5.81 (ddt, *J* = 16.9, 10.1, 7.3 Hz, 1H), 5.12-5.07 (m, 2H), 4.85 (t, *J* = 7.3 Hz, 1H), 3.75-3.60 (m, 2H), 3.05-3.01 (m, 2H), 2.75 (ddd, *J* = 14.0, 6.9, 6.9 Hz, 1H), 2.55-2.48 (m, 4H); ^{13}C{^{1}H} NMR ($CDCl_3$, 100.5 MHz): δ 196.3, 152.2, 137.2, 134.59, 134.57, 130.7, 128.3, 127.3, 127.0, 126.1, 125.8, 117.8, 111.2, 58.8, 42.2, 40.7, 27.7, 26.0; IR (ν, cm^{-1}): 1659, 1591, 1517, 1390, 1355, 1278, 1224, 1187, 810; HRMS (ESI) m/z: $[M + H]^+$ calcd for $C_{20}H_{22}NO$, 292.1701; found, 292.1678.

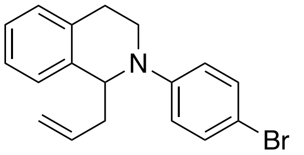

1-allyl-2-(4-bromophenyl)-1,2,3,4-tetrahydroisoquinoline (9e)

The crude product was purified by preparative TLC (*n*-hexane/EtOAc/Et_3N = 100/3/1) to afford **9e** **(**39.9 mg) in 61% yield as light yellow oil.

^{1}H NMR ($CDCl_3$, 399.8 MHz): δ 7.31 (d, *J* = 9.2 Hz, 2H), 7.20-7.10 (m, 4H), 6.75 (d, *J* = 9.2 Hz, 2H), 5.83 (ddt, *J* = 17.4, 10.1, 7.3 Hz, 1H), 5.09-5.05 (m, 2H), 4.69 (t, *J* = 6.9 Hz, 1H), 3.65-3.51 (m, 2H), 3.05-2.98 (m, 1H), 2.93-2.86 (m, 1H), 2.74-2.67 (m, 1H), 2.52-2.45 (m, 1H); ^{13}C{^{1}H} NMR ($CDCl_3$, 100.5 MHz): δ 148.5, 137.9, 135.4, 134.8, 132.0, 128.6, 127.4, 126.9, 126.0, 117.4, 115.5, 109.1, 59.4, 42.1, 40.9, 27.4; IR (ν, cm^{-1}): 1587, 1490, 1389, 1332, 1223, 914, 799, 755; HRMS (ESI) m/z: $[M + H]^+$ calcd for $C_{18}H_{19}BrN$, 328.0701; found, 328.0698.

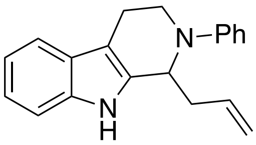

1-allyl-2-phenyl-2,3,4,9-tetrahydro-1H-pyrido[3,4-b]indole (9i)

The crude product was purified by flash column chromatography (*n*-hexane/EtOAc = 10/1) to afford **9i** (48.3 mg) in 84% yield as yellow oil.

^{1}H NMR ($CDCl_3$, 399.8 MHz): δ 7.71 (br-s, 1H), 7.46 (d, *J* = 7.3 Hz, 1H), 7.28-7.21 (m, 3H), 7.14 (dd, *J* = 7.3, 7.3 Hz, 1H), 7.08 (dd, *J* = 7.3, 7.3 Hz, 1H), 6.96 (d, *J* = 8.7 Hz, 2H), 6.78 (t, *J* = 7.3 Hz, 1H), 6.06-5.95 (m, 1H), 5.20-5.16 (m, 2H), 4.78 (t, *J* = 6.4 Hz, 1H), 3.91 (dd, *J* = 13.7, 4.1 Hz, 1H), 3.50 (ddd, *J* = 13.7, 11.4, 4.1 Hz, 1H), 2.92 (ddd, *J* = 16.5, 11.4, 4.1 Hz, 1H), 2.70-2.53 (m, 3H); ^{13}C{^{1}H} NMR ($CDCl_3$, 100.5 MHz): δ 150.3, 135.9, 135.7, 135.3, 129.4, 127.1, 121.8, 119.5, 118.8, 118.3, 116.40, 116.40, 110.9, 109.4, 55.9, 42.4, 38.4, 20.5; IR (ν, cm^{-1}): 3407, 1597, 1499, 1459, 1393, 1227, 744, 693; IR (ν, cm^{-1}): 1597, 1502, 1390, 1330, 1211, 889, 745, 691; HRMS (ESI) m/z: $[M + H]^+$ calcd for $C_{20}H_{21}N_2$, 289.1705; found, 289.1697.

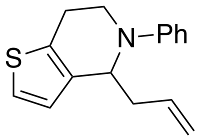

4-allyl-5-phenyl-4,5,6,7-tetrahydrothieno[3,2-c]pyridine (9j)

The crude product was purified by preparative TLC (*n*-hexane/Et_3N = 200/1) to afford **9j** (35.3 mg) in 69 % yield as orange oil.

^{1}H NMR ($CDCl_3$, 399.8 MHz): δ 7.24-7.20 (m, 2H), 7.05 (d, *J* = 5.5 Hz, 1H), 6.92 (d, *J* = 8.2 Hz, 2H), 6.83 (d, *J* = 5.5 Hz, 1H), 6.75 (t, *J* = 7.3 Hz, 1H), 5.97-5.87 (m, 1H), 5.11-5.04 (m, 2H), 4.79 (t, *J* = 6.4 Hz, 1H), 3.90 (dd, *J* = 13.7, 5.0 Hz, 1H), 3.52 (ddd, *J* = 13.7, 11.9, 4.1 Hz, 1H), 3.01 (ddd, *J* = 15.6, 11.9, 5.0 Hz, 1H), 2.74-2.50 (m, 3H); ^{13}C{^{1}H} NMR ($CDCl_3$, 100.5 MHz): δ 150.2, 137.0, 135.9, 134.3, 129.4, 125.8, 122.1, 118.4, 117.2, 115.9, 58.1, 41.5, 39.3, 23.7; IR (ν, cm^{-1}): 1596, 1499, 1396, 1215, 914, 747, 710, 691, 656; HRMS (ESI) m/z: $[M + H]^+$ calcd for $C_{16}H_{18}NS$, 256.1160; found, 256.1152.

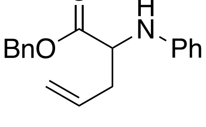

benzyl 2-(phenylamino)pent-4-enoate (9l)

The crude product was purified by preparative TLC (*n*-hexane/Et_3N = 100/1) to afford **9l** (14.3 mg) in 25% yield as yellow oil.

^{1}H NMR ($CDCl_3$, 399.8 MHz): δ 7.37-7.27 (m, 5H), 7.16 (dd, J = 8.2, 8.2 Hz, 2H), 6.74 (t, J = 7.3 Hz, 1H), 6.61 (d, J = 7.3 Hz), 5.75 (ddd, J = 17.4, 9.6, 7.3 Hz, 1H), 5.15-5.10 (m, 4H), 4.23-4.15 (m, 2H), 2.64-2.55 (m, 2H); $^{13}C\{^{1}H\}$ NMR ($CDCl_3$, 100.5 MHz): δ 173.3, 146.6, 135.6, 132.7, 129.4, 128.7, 128.5, 128.4, 119.2, 118.6, 113.7, 67.0, 56.2, 37.1; IR (ν, cm^{-1}): 1737, 1604, 1505, 1315, 1262, 1180, 1147, 749, 693; HRMS (ESI) m/z: $[M + Na]^+$ calcd for $C_{18}H_{19}NNaO_2$, 304.1314; found, 304.1302.

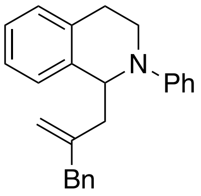

1-(2-benzylallyl)-2-phenyl-1,2,3,4-tetrahydroisoquinoline (9n)

The crude product was purified by flash column chromatography (*n*-hexane/DCM/Et_3N = 100/2/1), and then, preparative TLC (chromatorex NH-TLC plate, *n*-hexane) to afford **9n** (42.1 mg) in 62% yield as light yellow oil.

^{1}H NMR ($CDCl_3$, 399.8 MHz): δ 7.27-7.03 (m, 11H), 6.87 (d, J = 8.2 Hz, 2H), 6.73 (t, J = 7.3 Hz, 1H), 4.86-4.83 (m, 3H), 3.60-3.56 (m, 2H), 3.31 (d, J = 7.3 Hz, 2H), 3.03-2.95 (m, 1H), 2.78 (dt, J = 16.0, 5.0 Hz, 1H), 2.65 (dd, J = 13.7, 7.3 Hz, 1H), 2.33 (dd, J = 13.7, 7.3 Hz, 1H); $^{13}C\{^{1}H\}$ NMR ($CDCl_3$, 100.5 MHz): δ 149.5, 146.2, 139.4, 138.4, 134.8, 129.2, 129.1, 128.5, 128.3, 127.4, 126.5, 126.1, 125.6, 117.4, 114.8, 114.3, 58.8, 43.2, 41.71, 41.68, 26.8; IR (ν, cm^{-1}): 1596, 1502, 1494, 1390, 1264, 1029, 894, 733, 695; HRMS (ESI) m/z: $[M + H]^+$ calcd for $C_{25}H_{26}N$, 340.2065; found, 340.2063.

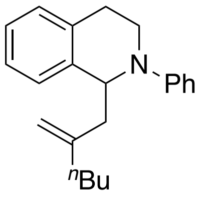

1-(2-methylenehexyl)-2-phenyl-1,2,3,4-tetrahydroisoquinoline (9o)

The crude product was purified by preparative TLC (*n*-hexane/DCM/Et_3N = 100/2/1) to afford **9o** (33.3 mg) in 55% yield as light yellow oil.

^{1}H NMR ($CDCl_3$, 399.8 MHz): δ 7.24-7.21 (m, 2H), 7.16-7.06 (m, 4H), 6.90 (d, J = 8.2 Hz, 2H), 6.72 (t, J = 7.3 Hz, 1H), 4.85-4.80 (m, 2H), 4.71 (s, 1H), 3.64-3.60 (m, 2H), 3.06-2.98 (m, 1H), 2.87-2.81 (m, 1H), 2.70 (dd, J = 13.7, 7.3 Hz, 1H), 2.36 (dd, J = 13.7, 7.3 Hz, 1H), 2.03 (t, J = 6.9 Hz, 1H), 1.42-1.24 (m, 2H), 0.87 (t, J = 7.3 Hz, 3H); $^{13}C\{^{1}H\}$ NMR ($CDCl_3$, 100.5 MHz): δ 149.6, 147.1, 138.7, 134.8, 129.3, 128.6, 127.7, 126.6, 125.6, 117.4, 114.3, 112.4, 58.7, 42.8, 41.8, 36.0, 30.1, 27.1, 22.6, 14.1; IR (ν, cm^{-1}): 1597, 1502, 1390, 1330, 1211, 889, 745, 691; HRMS (ESI) m/z: $[M + H]^+$ calcd for $C_{22}H_{28}N$, 306.2222; found, 306.2224.

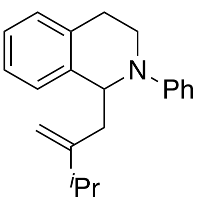

1-(3-methyl-2-methylenebutyl)-2-phenyl-1,2,3,4-tetrahydroisoquinoline (9p)

The crude product was purified by preparative TLC (*n*-hexane/DCM/Et_3N = 100/2/1) to afford **9p** (40.6 mg) in 70% yield as colorless oil.

^{1}H NMR ($CDCl_3$, 399.8 MHz): δ 7.24-7.20 (m, 2H), 7.14-7.06 (m, 4H), 6.89 (d, *J* = 8.2 Hz, 2H), 6.72 (t, 7.3 Hz, 1H), 4.86 (s, 1H), 4.83 (t, *J* = 7.3 Hz, 1H), 4.77 (s, 1H), 3.67-3.56 (m, 2H), 3.06-2.98 (m, 1H), 2.89-2.82 (m, 1H), 2.75 (dd, *J* = 14.2, 7.3 Hz, 1H), 2.37 (dd, *J* = 14.2, 7.3 Hz, 1H), 2.20-2.10 (m, 1H), 0.98 (t, *J* = 6.9 Hz, 6H); ^{13}C{^{1}H} NMR ($CDCl_3$, 100.5 MHz): δ 153.0, 149.6, 138.7, 134.9, 129.3, 128.6, 127.7, 126.6, 125.6, 117.4, 114.2, 109.9, 59.3, 41.9, 41.5, 33.7, 27.2, 21.83, 21.76; IR (ν, cm^{-1}): 1597, 1502, 1389, 1330, 1210, 888, 744, 690; HRMS (ESI) m/z: $[M + H]^+$ calcd for $C_{21}H_{26}N$, 292.2065; found, 292.2055.

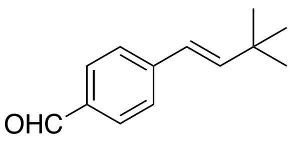

(E)-4-(3,3-dimethylbut-1-en-1-yl)benzaldehyde (12e)

The crude product was purified by flash column chromatography (*n*-hexane) to afford **12e** (24.0 mg) in 64% yield as colorless oil

^{1}H NMR ($CDCl_3$, 399.8 MHz): δ 9.94 (s, 1H), 7.79 (d, *J* = 8.2 Hz, 2H), 7.48 (d, *J* = 8.2 Hz, 2H), 6.43 (d, *J* = 16.0 Hz, 1H), 6.33 (d, *J* = 16.0 Hz, 1H), 1.13 (s, 9H); ^{13}C{^{1}H} NMR ($CDCl_3$, 100.5 MHz): δ 191.7, 145.7, 144.3, 134.8, 130.1, 126.4, 123.9, 33.7, 29.3; IR (ν, cm^{-1}): 2958, 1694, 1600, 1213, 1166, 971, 807; HRMS (ESI) m/z: $[M + H]^+$ calcd for $C_{13}H_{17}O$, 189.1279; found, 189.1286.

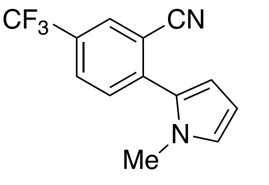

2-(1-methyl-1H-pyrrol-2-yl)-5-(trifluoromethyl)benzonitrile (15c)

The crude product was purified by flash column chromatography (*n*-hexane/EtOAc = 10/1) to afford **15c** (36.2 mg) in 72% yield as light yellow oil.

^{1}H NMR ($CDCl_3$, 399.8 MHz): δ 7.98 (d, *J* = 1.4 Hz, 1H), 7.83 (dd, *J* = 8.7, 1.4 Hz, 1H), 7.56 (d, *J* =

8.2 Hz, 1H), 6.84 (dd, J = 2.8, 1.8 Hz, 1H), 6.51 (dd, J = 3.7, 1.8 Hz, 1H), 6.27 (dd, J = 3.7, 2.8 Hz, 1H), 3.64 (s, 3H); $^{13}C\{^1H\}$ NMR ($CDCl_3$, 100.5 MHz): δ 140.2, 131.1, 130.7 (q, J = 3.8 Hz), 129.6 (q, J = 33.6 Hz), 129.1 (q, J = 3.8 Hz), 128.7, 126.4, 123.1 (q, J = 273.2 Hz), 117.6, 113.05, 113.02, 109.0, 35.1; ^{19}F NMR ($CDCl_3$, 376.2 MHz): δ -62.8; IR (ν, cm^{-1}): 1329, 1169, 1125, 1080, 751, 730, 722; HRMS (ESI) m/z: $[M + H]^+$ calcd for $C_{13}H_{10}F_3N_2$, 251.0796; found, 251.0787.

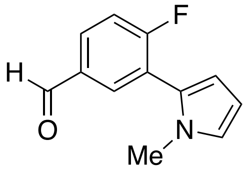

4-fluoro-3-(1-methyl-1H-pyrrol-2-yl)benzaldehyde (15e)

The crude product was purified by flash column chromatography (*n*-hexane/EtOAc = 10/1) to afford **15e** (30.2 mg) in 74% yield as colorless oil.

^1H NMR ($CDCl_3$, 399.8 MHz): δ 9.99 (s, 1H), 7.91-7.86 (m, 2H), 7.32-7.27 (m, 1H), 6.80 (dd, J = 1.8, 2.8 Hz, 1H), 6.30 (dd, J = 3.7, 1.8 Hz, 1H), 6.25 (dd, J = 3.7, 2.8 Hz, 1H); $^{13}C\{^1H\}$ NMR ($CDCl_3$, 100.5 MHz): δ 190.5, 163.5 (d, J = 256.9 Hz), 134.0 (d, J = 5.8 Hz), 132.9 (d, J = 2.9 Hz), 130.5 (d, J = 9.6 Hz), 126.5, 124.3, 122.4 (d, J = 16.3 Hz), 116.8 (d, J = 24.0 Hz), 110.9, 108.2, 34.7 (d, J = 4.8 Hz); ^{19}F NMR ($CDCl_3$, 376.2 MHz): δ -103.2; IR (ν, cm^{-1}): 1699, 1582, 1497, 1249, 1225, 1167, 825, 718, 614; HRMS (ESI) m/z: $[M + H]^+$ calcd for $C_{12}H_{11}FNO$, 204.0825; found, 204.0818.

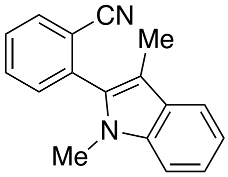

2-(1,3-dimethyl-1H-indol-2-yl)benzonitrile (15l)

The crude product was purified by flash column chromatography (*n*-hexane/EtOAc = 20/1 to 10/1) to afford **15l** (15.4 mg) in 31% yield as white solid.

Mp 126-128 ºC; ^1H NMR ($CDCl_3$, 399.8 MHz): δ 7.83 (d, J = 7.8 Hz, 1H), 7.70 (dd, J = 7.8, 7.8 Hz, 1H), 7.63 (d, J = 7.8 Hz, 1H), 7.53 (dd, J = 7.8, 7.8 Hz, 1H), 7.47 (d, J = 7.8 Hz, 1H), 7.36-7.28 (m, 2H), 7.17 (dd, J = 7.8 Hz, 1H), 3.59 (s, 3H), 2.26 (s, 3H); $^{13}C\{^1H\}$ NMR ($CDCl_3$, 100.5 MHz): δ 137.6, 136.0, 133.3, 133.2, 132.4, 132.3, 128.5, 128.1, 122.6, 119.4, 119.3, 117.9, 114.5, 111.4, 109.4, 30.9, 9.5; IR (ν, cm^{-1}): 1467, 1428, 1384, 1362, 1330, 1243, 1233, 765, 740; HRMS (ESI) m/z: $[M + H]^+$ calcd for $C_{17}H_{15}N_2$, 247.1235; found, 247.1227.

1-14. UV-Vis spectra

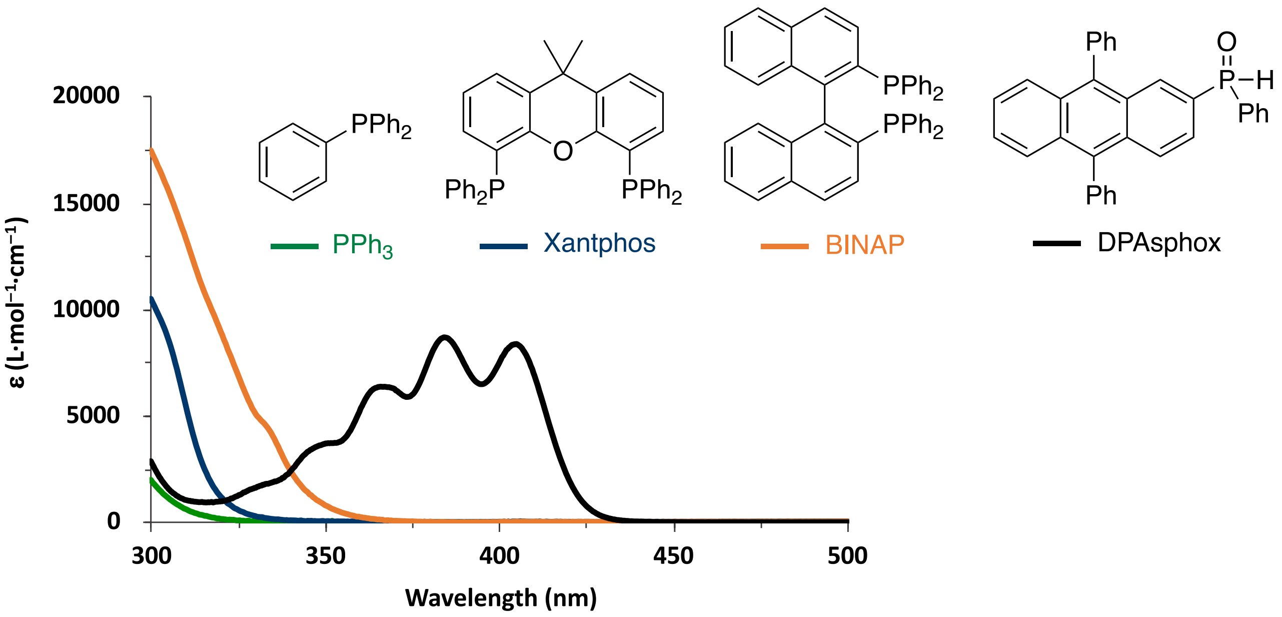

Supplementary Figure 30. UV-Vis spectra of phosphine ligands in DMF.

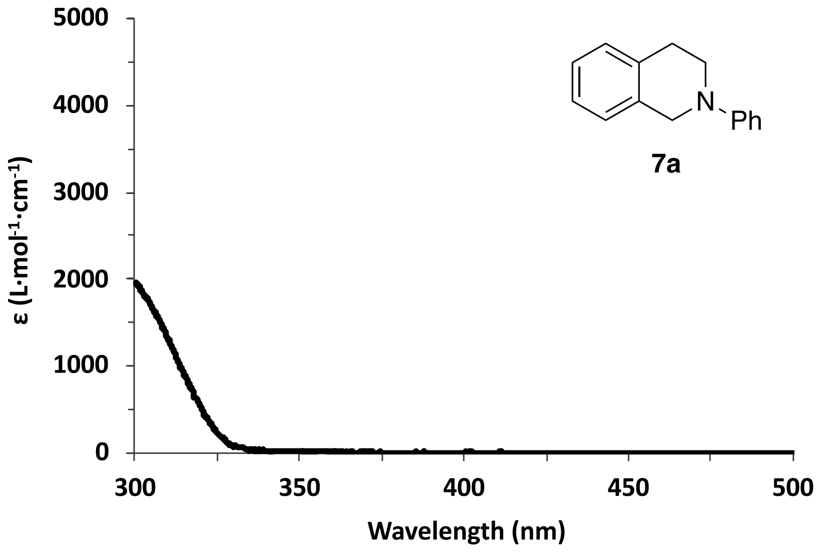

Supplementary Figure 31. UV-Vis spectrum of **7a** in DMF (0.05 mM).

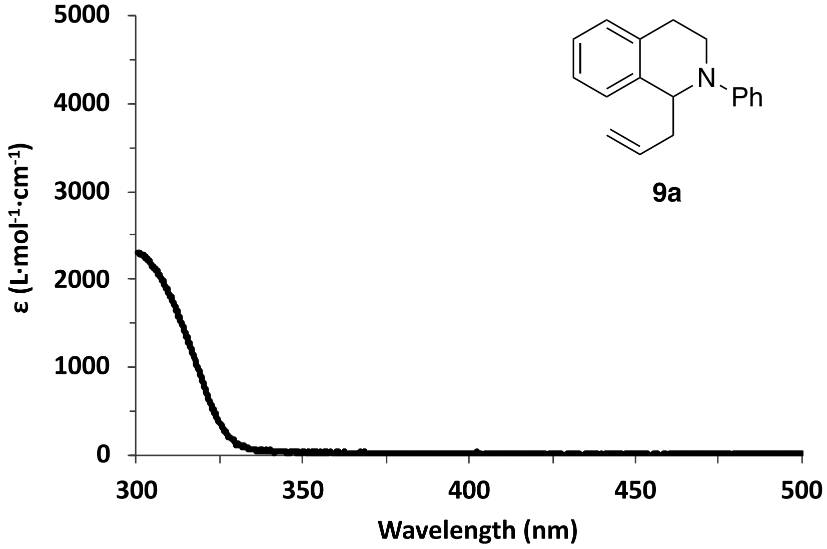

Supplementary Figure 32. UV-Vis spectrum of **9a** in DMF (0.05 mM).

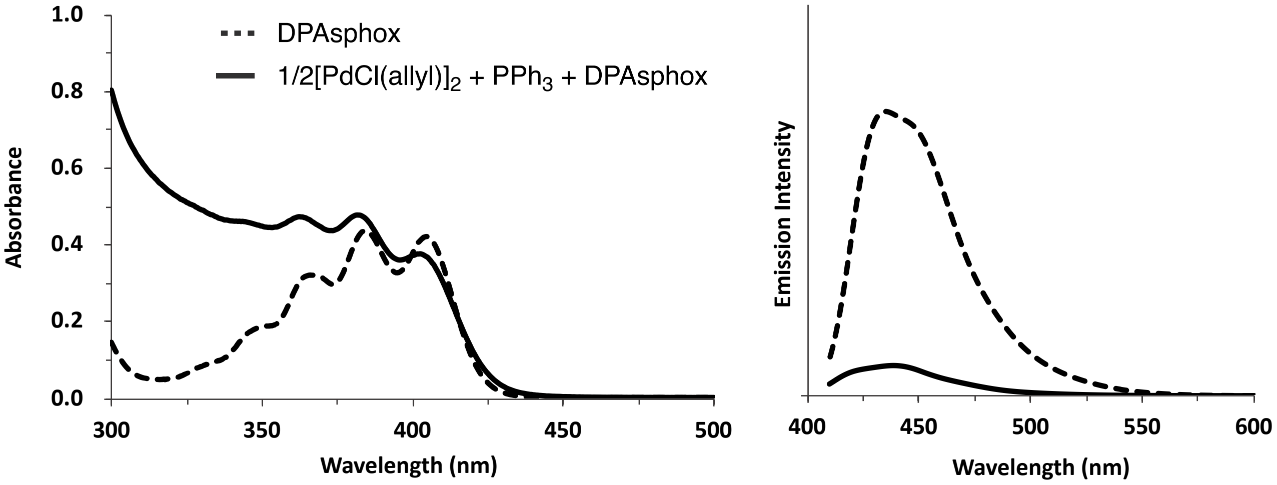

Supplementary Figure 33. Absorption and emission spectra of the mixture of $[PdCl(allyl)]_2$ (0.5 eq), PPh_3, and DPAsphox in DMF (0.05 mM).

Absorption and emission (irradiation at 400 nm) spectra of DPAsphox (dotted line) and the mixture of $[PdCl(allyl)]_2$ (0.5 eq), PPh_3, and DPAsphox (solid line) in DMF (0.05 mM) were shown in Supplementary Figure 33. The UV-Vis spectrum of the mixture were measured after stirring for 1 h at room temperature under light-shielding condition. The emission of DPAsphox was suppressed by the additon of $1/2[PdCl(allyl)]_2$ and PPh_3.

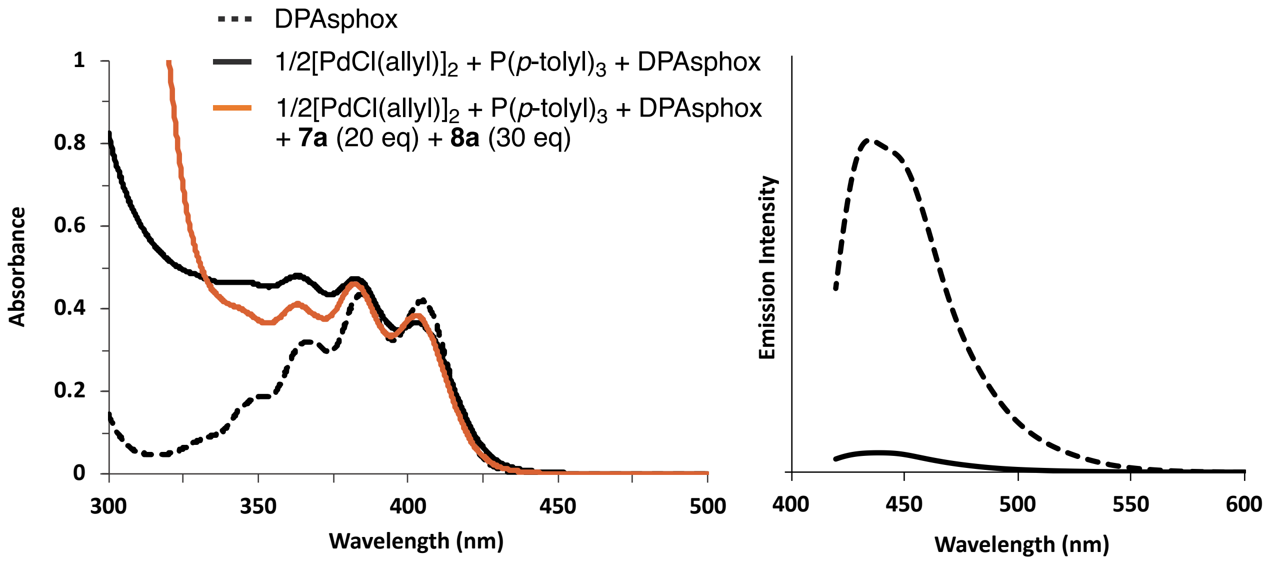

Supplementary Figure 34. UV-Vis spectra of the mixture of $[PdCl(allyl)]_2$ (0.5 eq), P(*p*-tolyl)$_3$, and DPAsphox, and the reaction mixture of α-allylation of amine.

Absorption and emission (irradiation at 400 nm) spectra of DPAsphox (dotted line), the mixture of $[PdCl(allyl)]_2$ (0.5 eq), P(*p*-tolyl)$_3$, and DPAsphox (dotted black line), and the reaction mixture of $[PdCl(allyl)]_2$ (0.5 eq), P(*p*-tolyl)$_3$, DPAsphox, **7a** (20 eq), and **8a** (30 eq) (orange line) in DMF (0.05 mM) were shown in Supplementary Figure 34. The UV-Vis spectra of the mixtures were measured after stirring for 1 h at room temperature under light-shielding condition. The absorption in the addition of **7a** and **8a** showed almost no change in the visible region. The emission of DPAsphox was suppressed by the additon of 1/2$[PdCl(allyl)]_2$ and P(*p*-tolyl)$_3$.

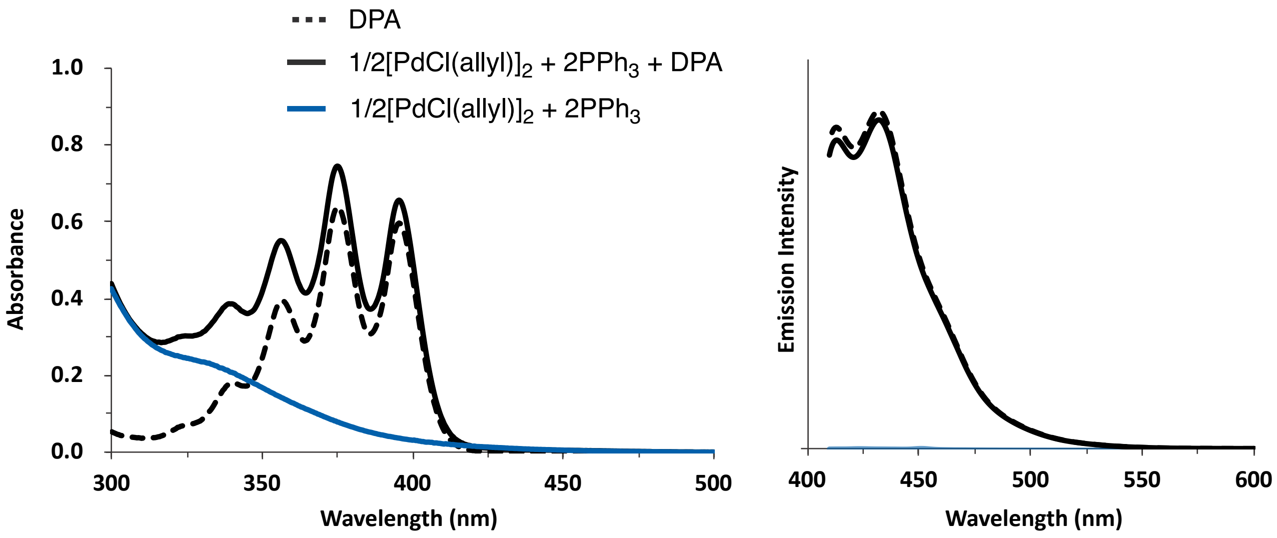

Supplementary Figure 35. UV-Vis spectra of the mixture of $[PdCl(allyl)]_2$, PPh_3, and DPA in DMF (0.05 mM).

Absorption and emission (irradiation at 400 nm) spectra of 9,10-diphenylanthracene (DPA) (dotted black line), the mixture of $[PdCl(allyl)]_2$ (0.5 eq), PPh_3 (2 eq) and DPA (solid black line), and the mixture of $[PdCl(allyl)]_2$ (0.5 eq) and PPh_3 (2 eq) (blue line) in DMF (0.05 mM) were shown in Supplementary Figure 35. The UV-Vis spectra of the mixtures were measured after stirring for 1 h at room temperature under light-shielding condition.

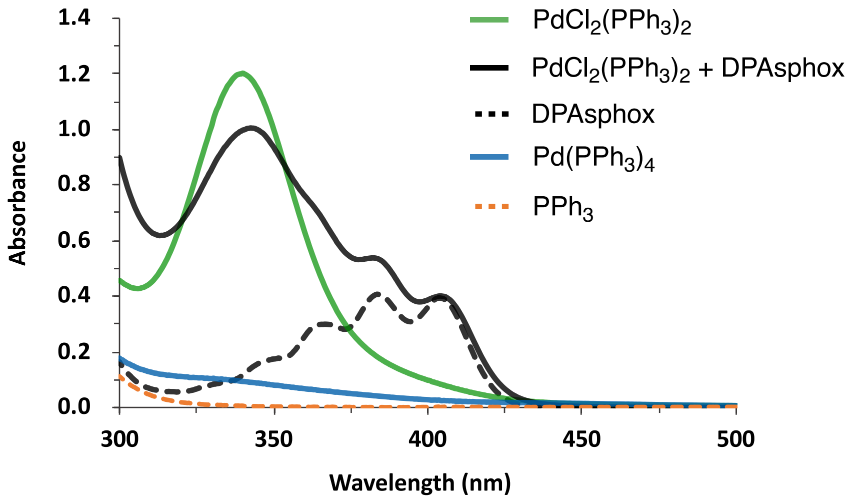

Supplementary Figure 36. Absorption spectra of the mixture of $PdCl_2(PPh_3)_2$ and DPAsphox in DMA (0.05 mM).

Initial observatons of the absorption spectra using $PdCl_2(PPh_3)_2$ in DMA (0.05 mM) were shown in Supplementary Figure 36. The mixture of $PdCl_2(PPh_3)_2$ and DPAsphox (solid black line) still showed the peak at 340 nm as same as $PdCl_2(PPh_3)_2$ even after stirring for 1 h at room temperature under light-shielding condition.

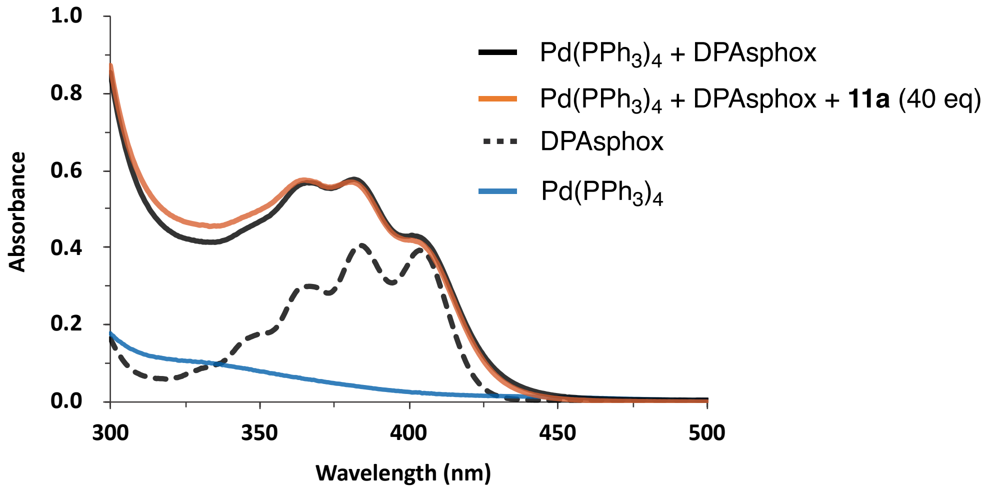

Supplementary Figure 37. Absorption spectra of the mixture of Pd(PPh_3)$_4$, DPAsphox, and **11a** (40 eq) in DMA (0.05 mM).

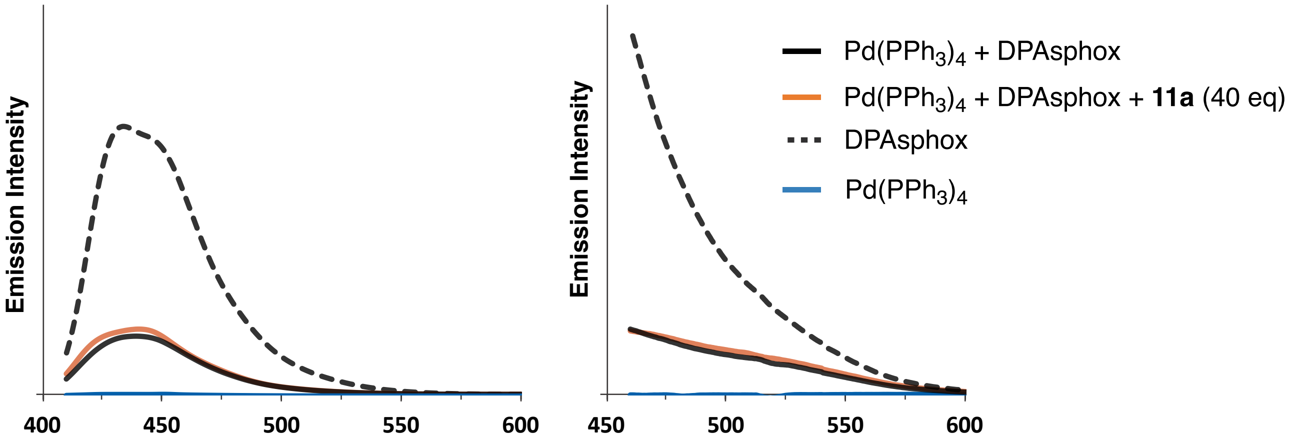

Supplementary Figure 38. Emission (irraditation at 400 nm (left) and 450 nm (right)) spectra of the mixture of Pd(PPh_3)$_4$, DPAsphox, and **11a** (40 eq) in DMA (0.05 mM).

Absorption (Supplementary Figure 37) and emission spectra (Supplementary Figure 38) of the mixture of Pd(PPh_3)$_4$ and DPAsphox (solid black line), the mixture of Pd(PPh_3)$_4$, DPAsphox, and **11a** (40 eq) (orange line), DPAsphox (broken black line), and Pd(PPh_3)$_4$ (blue line) in DMA (0.05 mM) were shown. The emission spectra (irradiation at 450 nm) were corrected by subtracting background emission. The UV-Vis spectra of the mixture were measured after stirring for 1 h at room temperature under light-shielding condition. The emission of DPAsphox was suppressed by the additon of Pd(PPh_3)$_4$.

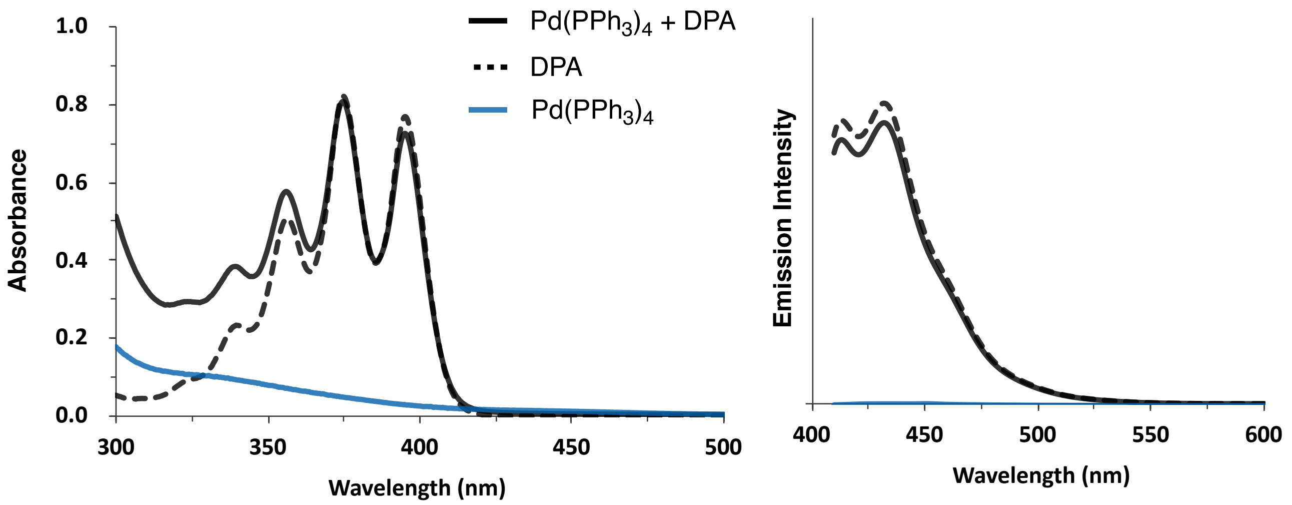

Supplementary Figure 39. UV-Vis spectra of the mixture of $Pd(PPh_3)_4$ and DPA in DMA (0.05 mM).

Absorption and emission (irradiation at 400 nm) spectra of the mixture of $Pd(PPh_3)_4$ and DPA (solid black line), DPA (dotted black line), and $Pd(PPh_3)_4$ (blue line) in DMA (0.05 mM) were shown in Supplementary Figure 39. The UV-Vis spectrum of the mixture was measured after stirring for 1 h at room temperature under light-shielding condition.

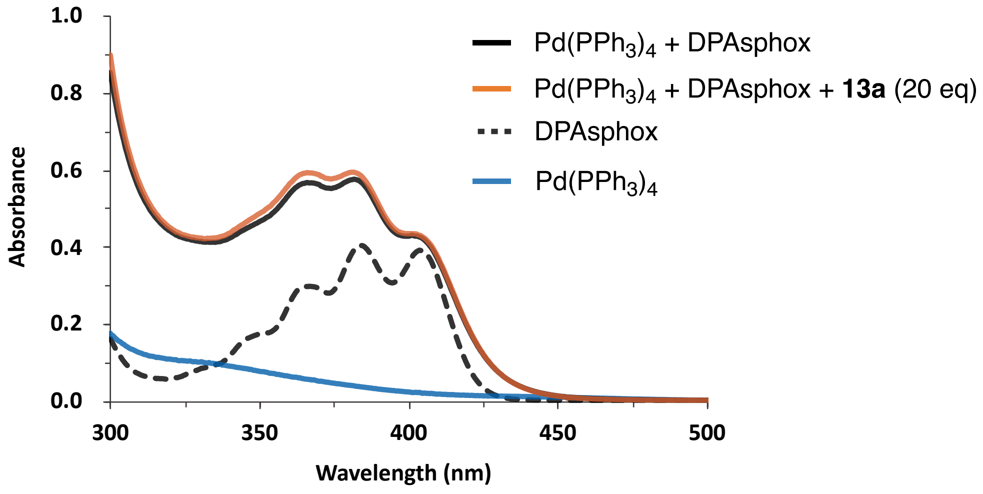

Supplementary Figure 40. Absorption spectra of the mixture of $Pd(PPh_3)_4$, DPAsphox, and 2-chlorobenzonitrile (**13a**) in DMA (0.05 mM).

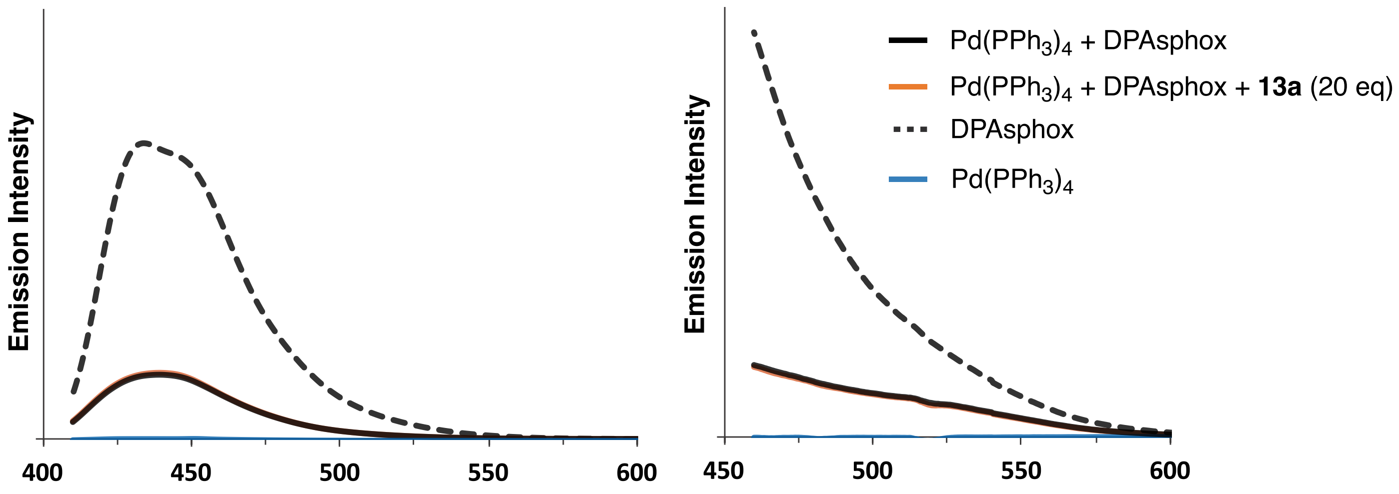

Supplementary Figure 41. Emission (irraditation at 400 nm (left) and 450 nm (right)) spectra of the mixture of $Pd(PPh_3)_4$, DPAsphox, and **13a** in DMA (0.05 mM).

Absorption (Supplementary Figure 40) and emission spectra (Supplementary Figure 41) of the mixture of $Pd(PPh_3)_4$ and DPAsphox (solid black line), the mixture of $Pd(PPh_3)_4$, DPAsphox, and 2-chlorobenzonitrile (**13a**) (20 eq) (orange line), DPAsphox (dotted black line), and $Pd(PPh_3)_4$ (blue line) in DMA (0.05 mM) were shown. The emission spectra (irradiation at 450 nm) were corrected by subtracting background emission. The UV-Vis spectra of the mixtures were measured after stirring for 1 h at room temperature under light-shielding condition. The addition of **13a** showed no change in the absorption and emission spectra.

1-15. Supplemental experiments

Addition of TEMPO as a radical oxidant

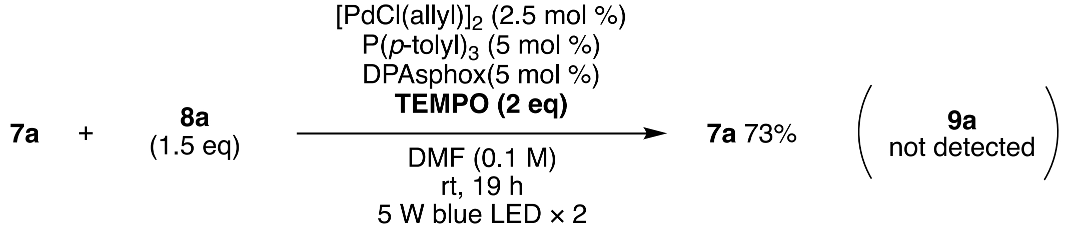

An 20 mL Shlenk tube containing a magnetic stirring bar was charged with **7a** (41.9 mg, 0.20 mmol, 1.0 equiv), $[PdCl(allyl)]_2$ (1.8 mg, 2.5 mol %), P(*p*-tolyl)$_3$ (3.0 mg, 5.0 mol %), DPAsphox (4.5 mg, 5.0 mol %), TEMPO (62.5 mg, 0.40 mmol, 2.0 equiv), **8a** (33.8 μL, 0.30 mmol, 1.5 equiv), and DMF (2.0 mL, 0.10 M). After the reaction mixture was degassed by freeze-pump-thaw cycles three times, it was sitrred for 19 h at room temperature under irradiation with 5 W blue LED lights. Then, water (3 mL) was added to the reaction, and the aqueous layer was extracted with EtOAc (3 mL × 3). The combined organic layer was washed with water (3 mL × 3) and brine (3 mL), dried over Na_2SO_4, and concentrated. The crude product was purified by flash column chromatography (*n*-hexane to *n*-hexane/EtOAc = 30/1) to recover **7a** (30.5 mg) as colorless oil.

Addition of nitrobenzene as a SET inhibitor

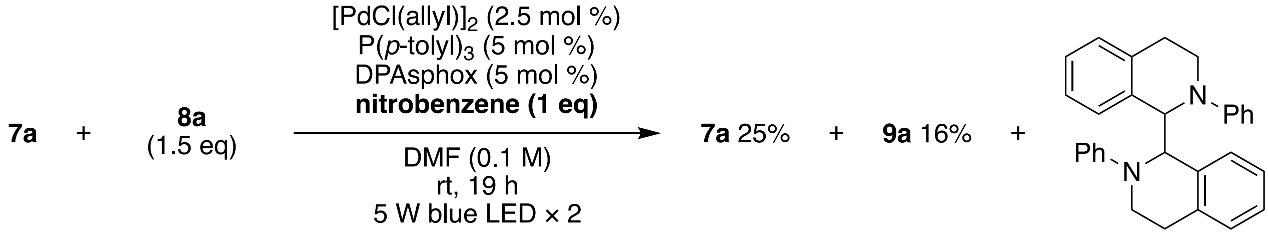

S9 18% (dr = 1.27:1)

An 20 mL Shlenk tube containing a magnetic stirring bar was charged with **7a** (41.9 mg, 0.20 mmol, 1.0 equiv), $[PdCl(allyl)]_2$ (1.8 mg, 2.5 mol %), P(*p*-tolyl)$_3$ (3.0 mg, 5.0 mol %), DPAsphox (4.5 mg, 5.0 mol %), nitrobenzene (20.5 μL, 0.20 mmol, 1.0 equiv), **8a** (33.8 μL, 0.30 mmol, 1.5 equiv), and DMF (2.0 mL, 0.10 M). After the reaction mixture was degassed by freeze-pump-thaw cycles three times, it was sitrred for 19 h at room temperature under irradiation with 5 W blue LED lights. Then, water (3 mL) was added to the reaction, and the aqueous layer was extracted with EtOAc (3 mL × 3). The combined organic layer was washed with water (3 mL × 3) and brine (3 mL), dried over Na_2SO_4, and concentrated. The yield of **7a**, **9a**, and **S9**[39] was determined by ^{1}H NMR using triphenylmethane as an internal standard.

Addition of methyl vinyl ketone as a radical scavenger

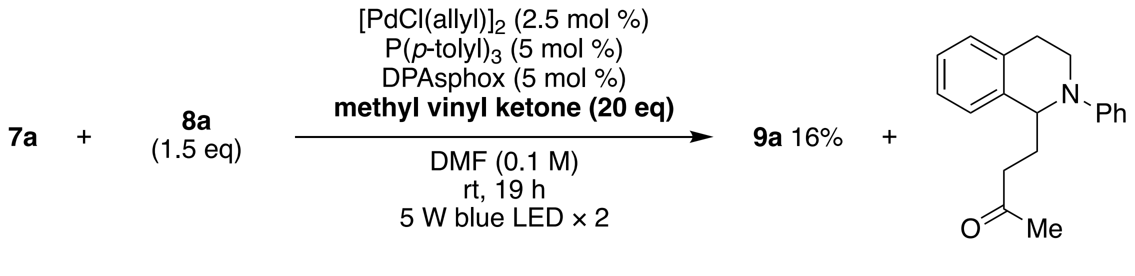

S10 6%

An 20 mL Shlenk tube containing a magnetic stirring bar was charged with **7a** (41.9 mg, 0.20 mmol, 1.0 equiv), $[PdCl(allyl)]_2$ (1.8 mg, 2.5 mol %), P(*p*-tolyl)$_3$ (3.0 mg, 5.0 mol %), DPAsphox (4.5 mg, 5.0 mol %), methyl vinyl ketone (326 μL, 4.0 mmol, 20.0 equiv), **8a** (33.8 μL, 0.30 mmol, 1.5 equiv), and DMF (2.0 mL, 0.10 M). After the reaction mixture was degassed by freeze-pump-thaw cycles three times, it was sitrred for 19 h at room temperature under irradiation with 5 W blue LED lights. Then, water (3 mL) was added to the reaction, and the aqueous layer was extracted with EtOAc (3 mL × 3). The combined organic layer was washed with water (3 mL × 3) and brine (3 mL), dried over Na_2SO_4, and concentrated. The crude product was purified by flash column chromatography (hexane to hexane/EtOAc = 100/1) to afford **9a** (5.0 mg) as light yellow oil and **S10**[39] (3.6 mg) as light yellow oil.

Addition of P(OPh)$_3$ in the Heck reaction using Xantphos

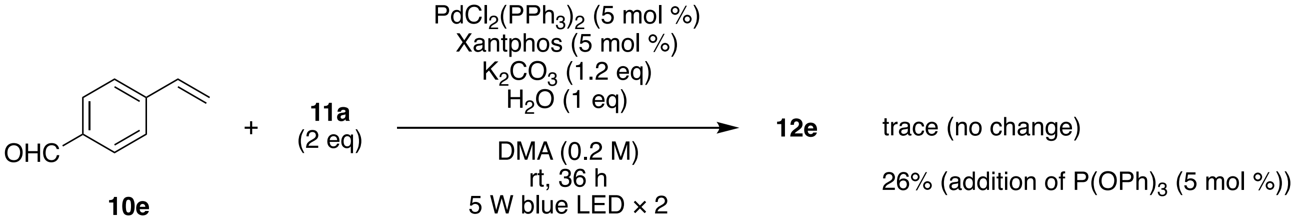

10e

An 20 mL Shlenk tube containing a magnetic stirring bar was charged with $PdCl_2(PPh_3)_2$ (7.0 mg, 5.0 mol %), Xantphos (4.5 mg, 5.0 mol %), and K_2CO_3 (33.2 mg, 1.2 equiv). After the tube was evacuated and filled with argon, styrene **10e** (0.20 mmol, 1.0 equiv), alkyl bromide **11a** (44.9 μL, 2.0 equiv), distilled water (3.6 μL, 1.0 equiv), P(OPh)$_3$ (2.6 μL, 5.0 mol %) and degassed DMA (1.0 mL, 0.20 M) were added under a stream of argon. The reaction mixture was sitrred for 36 h at room temperature under irradiation with 5 W blue LED lights. Then, water (3 mL) was added to the reaction, and the aqueous layer was extracted with EtOAc (3 mL × 3). The combined organic layer was washed with water (3 mL × 3) and brine (3 mL), dried over Na_2SO_4, and concentrated. The yield of **12e** was determined as trace (without P(OPh)$_3$) and 26% (with 5 mol % of P(OPh)$_3$) by ^{1}H NMR analysis using dimethyl terephthalate as an internal standard.

KIE experiment for the Heck reaction[26]

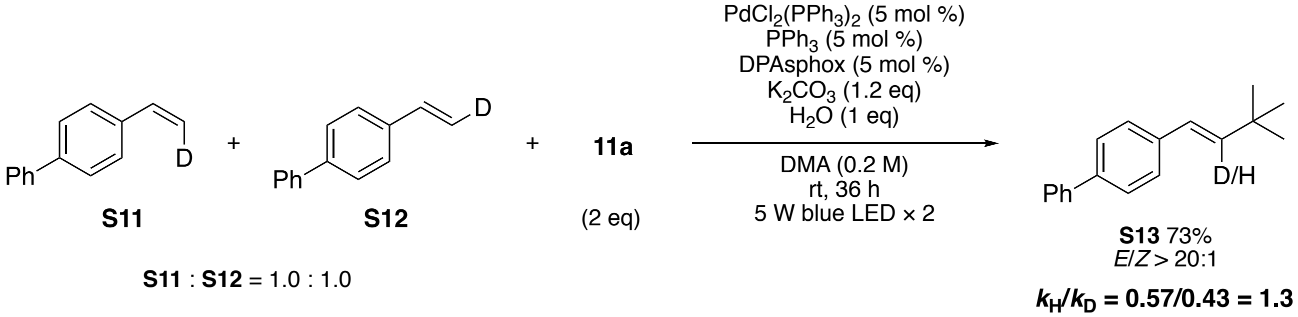

An 20 mL Shlenk tube containing a magnetic stirring bar was charged with the 1:1 mixture of **S11** and **S12** (36.2 mg, 0.2 mmol, 1.0 equiv), $PdCl_2(PPh_3)_2$ (7.0 mg, 5.0 mol %), PPh_3 (2.6 mg, 5.0 mol %), DPAsphox (4.5 mg, 5.0 mol %), and K_2CO_3 (33.2 mg, 1.2 equiv). After the tube was evacuated and filled with argon, alkyl bromide **11a** (44.9 µL, 2.0 equiv), distilled water (3.6 µL, 1.0 equiv) and degassed DMA (1.0 mL, 0.20 M) were added under a stream of argon. The reaction mixture was sitrred for 36 h at room temperature under irradiation with 5 W blue LED lights. Then, water (3 mL) was added to the reaction, and the aqueous layer was extracted with EtOAc (3 mL × 3). The combined organic layer was washed with water (3 mL × 3) and brine (3 mL), dried over Na_2SO_4, and concentrated. The crude product was purified by flash column chromatography (*n*-hexane/EtOAc = 10/1 to 5/1) to afford **S13** (34.4 mg) as white solid. The ratio of k_H/k_D was 1.3 determined by ^{1}H NMR analysis.

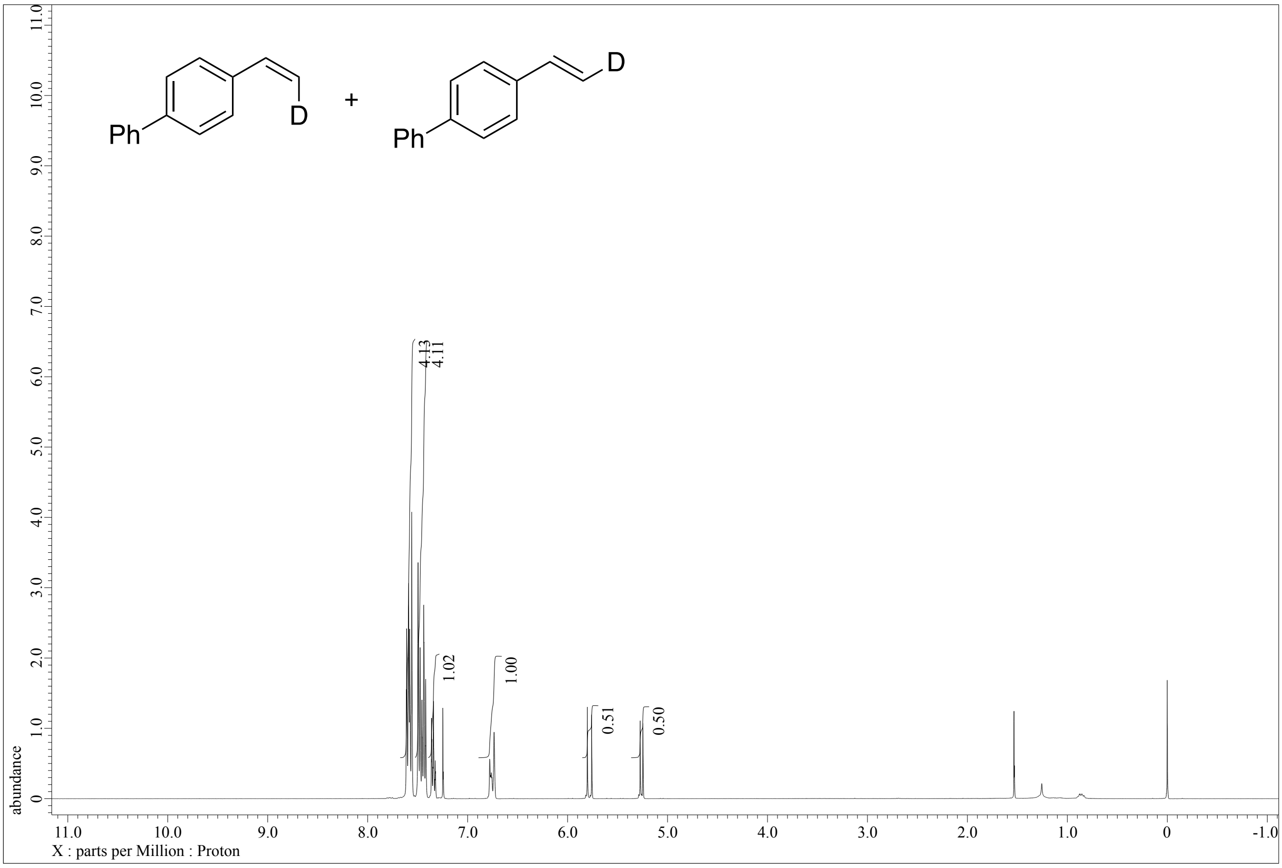

Supplementary Figure 42. ^{1}H NMR spectrum of the mixture of **S11** and **S12** ($CDCl_3$, 399.8 MHz, 20.3 ^oC).

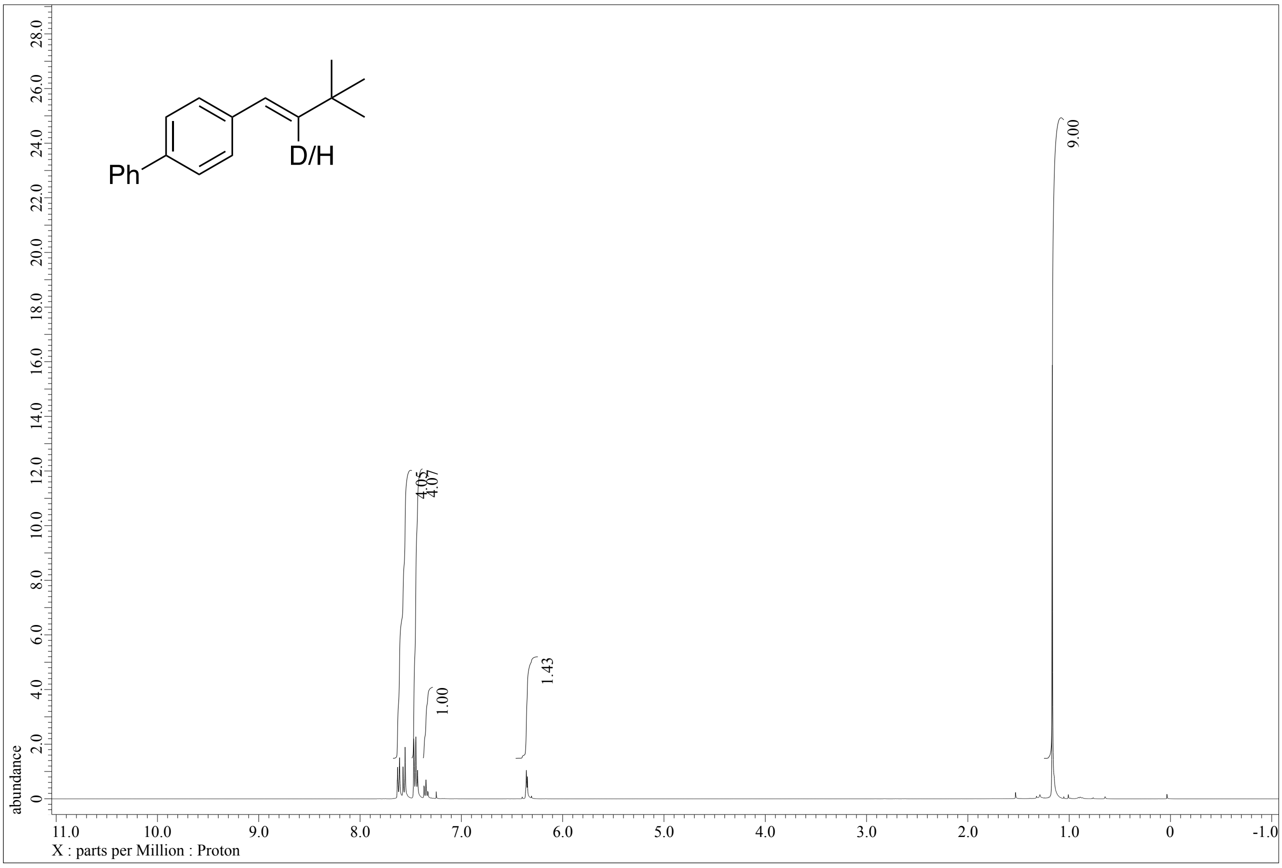

Supplementary Figure 43. ^{1}H NMR spectrum of **S13** ($CDCl_3$, 399.8 MHz, 21.1 ºC).

1-16. Cartesian coordinates and energies.

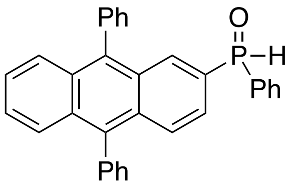

DPAsphox (1)

Energy (E) =

-1648.49118549 Hartree (MN15)

Gibbs free energy (G) =

-1648.09765649 Hartree (MN15)

Charge = 0, Spin = 1

C	-2.780801	1.115784	0.217227
C	-3.094109	-0.289894	0.190619
C	-2.087279	-1.236791	-0.102979
C	-0.770033	-0.802525	-0.350294
C	-0.449216	0.600258	-0.281690
C	-1.455902	1.549028	-0.009260
C	0.277004	-1.727346	-0.689043
C	1.560266	-1.311929	-0.893082
C	1.889039	0.074961	-0.768769
C	0.915743	0.993178	-0.477788
P	3.570712	0.669270	-0.989253
H	3.925753	0.298512	-2.300853
O	3.715830	2.135823	-0.714745
C	-3.844489	2.046634	0.460518
C	-5.119995	1.621517	0.709248
C	-5.418566	0.229183	0.736596
C	-4.439543	-0.691247	0.482835
C	4.597445	-0.447747	-0.025924
C	5.780916	-0.957581	-0.573822
C	6.602809	-1.786655	0.189554
C	6.242210	-2.105992	1.499627
C	5.060286	-1.602451	2.050376
C	4.236748	-0.776583	1.289063
C	-1.121845	3.002405	0.038176
C	-1.199079	3.712952	1.244311
C	-0.884734	5.070937	1.288636
C	-0.490298	5.736577	0.126523
C	-0.411035	5.038346	-1.079616
C	-0.724448	3.679960	-1.122962
C	-2.418081	-2.691520	-0.150005
C	-1.908157	-3.569460	0.816639
C	-2.216068	-4.929202	0.773507
C	-3.038202	-5.428748	-0.238121
C	-3.551052	-4.562840	-1.205729
C	-3.243125	-3.203209	-1.161316
H	0.028797	-2.780117	-0.781418
H	2.337472	-2.032511	-1.140359
H	1.189818	2.041011	-0.386944
H	-3.619789	3.108558	0.440249
H	-5.910078	2.345483	0.889512
H	-6.430544	-0.099979	0.956979
H	-4.672235	-1.751457	0.503329
H	6.056828	-0.709272	-1.597138
H	7.519338	-2.184624	-0.236959
H	6.881566	-2.753671	2.093594
H	4.780630	-1.859440	3.068273
H	3.308061	-0.392509	1.707978
H	-1.508123	3.191581	2.148055
H	-0.946036	5.608438	2.231351
H	-0.245498	6.794918	0.160913
H	-0.106318	5.551049	-1.988247
H	-0.662738	3.132742	-2.061548
H	-1.266406	-3.177157	1.603071
H	-1.815113	-5.597650	1.531032
H	-3.278229	-6.488214	-0.272300
H	-4.190166	-4.945872	-1.997091

H	-3.642759	-2.525465	-1.913044

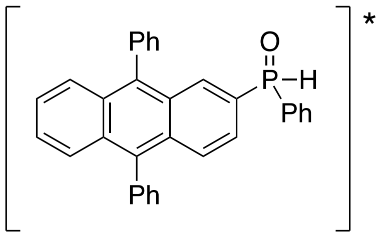

DPAsphox* in the singlet excited state

Energy (E) =

-1648.38472358 Hartree (MN15)

Gibbs free energy (G) =

-1647.99263758 Hartree (MN15)

Charge = 0, Spin = 1

C	-2.991566	0.830516	0.112193
C	-3.113336	-0.604051	0.150355
C	-1.950816	-1.422988	-0.070723
C	-0.666810	-0.821117	-0.252668
C	-0.524268	0.613305	-0.162119
C	-1.696202	1.434419	-0.018387
C	0.485023	-1.584925	-0.548805
C	1.757623	-1.009616	-0.626290
C	1.916024	0.360144	-0.417127
C	0.771764	1.154299	-0.182530
P	3.499247	1.190931	-0.516439
H	3.805174	1.320203	-1.886533
O	3.507569	2.509161	0.201533
C	-4.174811	1.593786	0.167557
C	-5.430169	1.006593	0.388688
C	-5.528791	-0.368587	0.546480
C	-4.374941	-1.160302	0.427972
C	4.730998	-0.018031	-0.014920
C	5.874731	-0.212213	-0.798488
C	6.862508	-1.106417	-0.384932
C	6.708555	-1.804897	0.813411
C	5.568446	-1.615664	1.600024
C	4.580309	-0.725156	1.187361
C	-1.561698	2.900713	-0.001687
C	-2.093571	3.669169	1.054367
C	-1.961757	5.054888	1.068692
C	-1.307514	5.710170	0.022612
C	-0.778126	4.965411	-1.035111
C	-0.895534	3.579081	-1.043614
C	-2.090283	-2.890974	-0.118876
C	-1.331471	-3.721074	0.729930
C	-1.471149	-5.105522	0.687537
C	-2.361723	-5.695209	-0.213308
C	-3.117492	-4.887494	-1.066643
C	-2.989548	-3.502145	-1.015436
H	0.383918	-2.651433	-0.719608
H	2.616888	-1.641738	-0.838897
H	0.916088	2.215600	-0.002599
H	-4.117277	2.669200	0.034294
H	-6.313387	1.636855	0.441095
H	-6.486167	-0.839670	0.749036
H	-4.461739	-2.234809	0.553437
H	5.990493	0.333610	-1.733243
H	7.747609	-1.259086	-0.996219
H	7.477398	-2.502173	1.135624
H	5.450555	-2.165460	2.529698
H	3.683792	-0.584315	1.788635
H	-2.592051	3.160850	1.876701
H	-2.366320	5.624762	1.901162
H	-1.209455	6.792397	0.031873
H	-0.275341	5.467519	-1.857605
H	-0.490523	3.002969	-1.872609
H	-0.644000	-3.263551	1.437916
H	-0.886546	-5.725917	1.361882
H	-2.465959	-6.776291	-0.249896
H	-3.806114	-5.338262	-1.776567
H	-3.572710	-2.875182	-1.686351

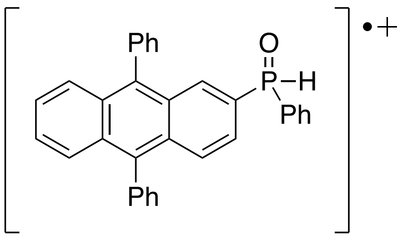

DPAsphox•+

Energy (E) =

-1648.28304846 Hartree (MN15)

Gibbs free energy (G) =

-1647.88874046 Hartree (MN15)

Charge = 1, Spin = 2

C	-2.835778	1.028928	0.186410
C	-3.085376	-0.385796	0.201755
C	-2.038332	-1.303425	-0.103203
C	-0.723183	-0.806453	-0.367606
C	-0.453502	0.599012	-0.275488
C	-1.513952	1.521823	-0.023470
C	0.328976	-1.679347	-0.737139
C	1.618557	-1.209967	-0.916870
C	1.902286	0.157253	-0.736911
C	0.882997	1.042407	-0.423277
P	3.577003	0.831833	-0.904062
H	3.928869	0.558996	-2.237549
O	3.624417	2.278919	-0.526247
C	-3.927306	1.911580	0.367074
C	-5.198751	1.432310	0.647102
C	-5.421677	0.052497	0.758951
C	-4.383591	-0.840573	0.538709
C	4.622737	-0.311824	-0.003975
C	5.775476	-0.827667	-0.608985
C	6.608873	-1.688312	0.105310
C	6.290639	-2.030684	1.420230
C	5.139518	-1.519435	2.027089
C	4.303387	-0.662520	1.316766
C	-1.241541	2.972908	0.022203
C	-1.543315	3.714841	1.177460
C	-1.274306	5.080106	1.221949
C	-0.724646	5.721999	0.110170
C	-0.432554	4.994268	-1.045490
C	-0.677729	3.624564	-1.088361
C	-2.309267	-2.755071	-0.151543
C	-1.607456	-3.637482	0.687869
C	-1.875399	-5.003055	0.647508
C	-2.826633	-5.504075	-0.244044
C	-3.520210	-4.635411	-1.089267
C	-3.273310	-3.266118	-1.037669
H	0.120796	-2.733132	-0.883902
H	2.411839	-1.904602	-1.183258
H	1.132806	2.089706	-0.280178
H	-3.767258	2.980230	0.277591
H	-6.017795	2.130405	0.787695
H	-6.409052	-0.321811	1.009987
H	-4.565754	-1.905267	0.633213
H	6.019098	-0.559572	-1.635158
H	7.502048	-2.091401	-0.363318
H	6.939879	-2.702212	1.975477
H	4.894312	-1.793837	3.049098
H	3.400640	-0.270170	1.782032
H	-1.965352	3.210080	2.043162
H	-1.494640	5.642578	2.124532
H	-0.523597	6.789021	0.144402
H	-0.012183	5.492878	-1.913936
H	-0.457026	3.055569	-1.988303
H	-0.871524	-3.242862	1.384357
H	-1.340809	-5.675968	1.311585
H	-3.027212	-6.571136	-0.279957
H	-4.254992	-5.023481	-1.788600
H	-3.807263	-2.586541	-1.697600

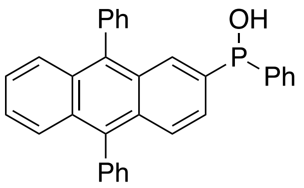

DPAphos (1')

Energy (E) =

-1648.48573154 Hartree (MN15)

Gibbs free energy (G) =

-1648.09338854 Hartree (MN15)

Charge = 0, Spin = 1

P	3.593516	0.683979	-1.212927
C	1.907820	0.097271	-0.865067
O	3.992773	-0.230288	-2.540141
C	0.932902	1.017023	-0.580496
C	-0.414723	0.624425	-0.276096
C	-0.737072	-0.779173	-0.308314
C	0.308359	-1.712846	-0.632217
C	1.581780	-1.297502	-0.886918
C	-1.413887	1.576872	0.007627
C	-2.736764	1.150811	0.261487
C	-3.060274	-0.251951	0.227465
C	-2.055994	-1.204752	-0.057276
C	-3.776277	2.082297	0.590326
C	-5.051577	1.663998	0.852696
C	-5.372162	0.277048	0.816796
C	-4.407309	-0.646105	0.521075
C	4.489376	-0.281606	0.051863
C	5.599995	-1.065286	-0.281831
C	6.307693	-1.743924	0.712946
C	5.917863	-1.639373	2.048309
C	4.812902	-0.854443	2.389152
C	4.103901	-0.180097	1.397206
C	-1.075453	3.030206	0.040244
C	-1.576267	3.900524	-0.938019
C	-1.259309	5.258469	-0.909115
C	-0.437216	5.764094	0.099644
C	0.066503	4.905851	1.078650
C	-0.250730	3.547858	1.048650
C	-2.393367	-2.658781	-0.089649
C	-3.199132	-3.181336	-1.110737
C	-3.514281	-4.539796	-1.141289
C	-3.027270	-5.393894	-0.150157
C	-2.224088	-4.883449	0.871221
C	-1.909722	-3.524747	0.900816
H	4.183535	0.341327	-3.300154
H	1.176386	2.076986	-0.595216
H	0.062997	-2.769879	-0.673798
H	2.363316	-2.016828	-1.124472
H	-3.530798	3.138796	0.637994
H	-5.822027	2.388705	1.102095
H	-6.385545	-0.047136	1.038315
H	-4.651914	-1.703874	0.514388
H	5.898154	-1.150512	-1.323360
H	7.164403	-2.356337	0.442333
H	6.470449	-2.167270	2.821282
H	4.501602	-0.772321	3.427504
H	3.236046	0.421848	1.665567
H	-2.218054	3.503646	-1.722085
H	-1.652985	5.920932	-1.675675
H	-0.190060	6.822222	0.122505
H	0.705784	5.293380	1.867683
H	0.141912	2.875960	1.809379
H	-3.578696	-2.512819	-1.880948
H	-4.138652	-4.931167	-1.940352
H	-3.272571	-6.452458	-0.173637
H	-1.843017	-5.542611	1.646975
H	-1.282515	-3.124111	1.694757

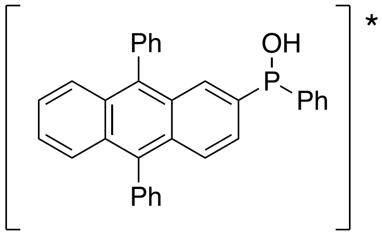

DPAphos* in the singlet excited state

Energy (E) =

-1648.37769345 Hartree (MN15)

Gibbs free energy (G) =

-1647.98741645 Hartree (MN15)

Charge = 0, Spin = 1

P	-3.522073	1.182052	0.879291
C	-1.931792	0.363286	0.569083
O	-3.880117	0.692158	2.428973
C	-0.790950	1.168140	0.360389
C	0.488597	0.634549	0.123551
C	0.647499	-0.798861	0.168643
C	-0.497138	-1.585388	0.435324
C	-1.766008	-1.022389	0.599206
C	1.645060	1.471487	-0.061658
C	2.950341	0.888567	-0.199659
C	3.106820	-0.542928	-0.148451
C	1.949053	-1.379336	0.036649
C	4.089866	1.668099	-0.482937
C	5.362670	1.100497	-0.651715
C	5.512999	-0.276919	-0.597200
C	4.384787	-1.084616	-0.376477
C	-4.636337	0.060413	-0.031403
C	-5.758018	-0.512649	0.578788
C	-6.634760	-1.309467	-0.161664
C	-6.404505	-1.533602	-1.518922
C	-5.289278	-0.960481	-2.136910
C	-4.412206	-0.169696	-1.397969
C	1.486849	2.935148	-0.109859
C	2.205850	3.773426	0.766474
C	2.052090	5.156305	0.722494
C	1.185883	5.738310	-0.206648
C	0.469131	4.923584	-1.087331
C	0.611619	3.540109	-1.035177
C	2.110067	-2.844777	0.095971
C	2.961098	-3.440372	1.047807
C	3.109130	-4.823456	1.108763
C	2.421069	-5.644047	0.211422
C	1.578448	-5.069763	-0.743777
C	1.419791	-3.687552	-0.797192
H	-4.007874	1.465313	3.000622
H	-0.914482	2.247187	0.402506
H	-0.387655	-2.660245	0.534818
H	-2.623361	-1.670430	0.771332
H	3.980751	2.741575	-0.596920
H	6.214260	1.746177	-0.847204
H	6.484278	-0.739382	-0.747250
H	4.503458	-2.162858	-0.404523
H	-5.933040	-0.340445	1.637416
H	-7.498383	-1.756252	0.325027
H	-7.088678	-2.152085	-2.094187
H	-5.101972	-1.134122	-3.193630
H	-3.538210	0.265657	-1.881523
H	2.872975	3.321908	1.497587
H	2.605920	5.781530	1.418115
H	1.069812	6.818174	-0.243997
H	-0.199691	5.368381	-1.819673
H	0.059397	2.908491	-1.727707
H	3.491579	-2.802857	1.751893
H	3.759899	-5.262559	1.860507
H	2.540693	-6.723248	0.256072
H	1.046562	-5.700276	-1.451586
H	0.769437	-3.241414	-1.546542

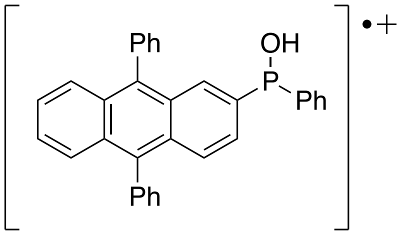

DPAphos•+

Energy (E) =

-1648.28489375 Hartree (MN15)

Gibbs free energy (G) =

-1647.88945175 Hartree (MN15)

Charge = 1, Spin = 2

P	2.729162	-2.766361	-1.157014
C	1.117317	-1.997404	-0.788798
O	2.476994	-4.189232	-0.357046
C	1.030741	-0.609539	-0.692376
C	-0.175770	0.043273	-0.369775
C	-1.370782	-0.743939	-0.243047
C	-1.273194	-2.147486	-0.426419
C	-0.056986	-2.763995	-0.663282
C	-0.218885	1.462383	-0.213260
C	-1.470195	2.095674	0.051541
C	-2.671358	1.311957	0.139302
C	-2.619511	-0.109160	-0.006967
C	-1.548752	3.486822	0.309170
C	-2.759941	4.099665	0.584243
C	-3.935077	3.335987	0.654134
C	-3.885971	1.965220	0.457259
C	3.730939	-1.800281	0.017598
C	3.449951	-1.797303	1.393466
C	4.199576	-1.003941	2.257800
C	5.226426	-0.197940	1.751294
C	5.507249	-0.192643	0.385479
C	4.763654	-0.999270	-0.480843
C	1.021831	2.258903	-0.319638
C	1.112221	3.299393	-1.260944
C	2.282048	4.045595	-1.374069
C	3.367244	3.776485	-0.537461
C	3.286123	2.748122	0.403786
C	2.126555	1.984145	0.507764
C	-3.859521	-0.912915	0.092799
C	-4.903933	-0.716989	-0.825213
C	-6.064358	-1.482503	-0.739080
C	-6.201639	-2.434768	0.273013
C	-5.171775	-2.627115	1.195997
C	-4.001354	-1.877332	1.103346
H	2.836655	-4.939656	-0.856568
H	1.922626	-0.012587	-0.857477
H	-2.173243	-2.750990	-0.380922
H	-0.003682	-3.844315	-0.758858
H	-0.640289	4.078507	0.311925
H	-2.792455	5.168400	0.771312
H	-4.881088	3.811977	0.892558
H	-4.790210	1.377593	0.570579
H	2.643285	-2.419776	1.776985
H	3.985455	-1.006794	3.323265
H	5.807413	0.425228	2.426489
H	6.305028	0.432989	-0.005637
H	4.984610	-1.006269	-1.546595
H	0.268029	3.501731	-1.915811
H	2.345799	4.836933	-2.115324
H	4.276134	4.365768	-0.620662
H	4.127410	2.534467	1.056582
H	2.068688	1.180308	1.239743
H	-4.792316	0.024930	-1.612536
H	-6.861553	-1.334059	-1.461792
H	-7.110533	-3.025824	0.342657
H	-5.278378	-3.362045	1.988720
H	-3.198007	-2.021847	1.822024

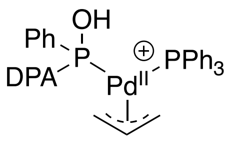

2

Energy (E) =

-2928.77672983 Hartree (MN15)

-2931.16954425 Hartree (ωB97XD)

-2930.58349180 Hartree (CAM-B3LYP)

Gibbs free energy (G) =

-2928.06134983 Hartree (MN15)

-2930.45416425 Hartree (ωB97XD)

-2929.86811180 Hartree (CAM-B3LYP)

Charge = 1, Spin = 1

C	1.352672	-2.536448	2.413913
C	2.624601	-2.014249	2.726292
C	0.266178	-1.646807	2.289285
Pd	1.865806	-0.970900	0.966370
P	3.986077	-0.485473	0.105488
P	0.501305	-0.081709	-0.655039
C	4.203152	1.015816	-0.899843
C	5.217294	-0.315229	1.430529
C	4.542665	-1.842877	-0.957306
C	4.867829	0.463776	2.545988
C	5.781325	0.665301	3.578692
C	7.046098	0.072943	3.512887
C	7.395173	-0.709709	2.411878
C	6.486682	-0.900913	1.367903
C	5.575477	-1.665075	-1.891252
C	6.007476	-2.739886	-2.666883
C	5.408986	-3.994587	-2.520049
C	4.377862	-4.177091	-1.597111
C	3.943708	-3.103263	-0.818410
C	4.869549	2.147151	-0.412737
C	4.924833	3.312293	-1.180690
C	4.315813	3.356340	-2.434726
C	3.659891	2.226222	-2.930923
C	3.606314	1.060566	-2.170168
C	-1.271892	-0.388437	-0.514222
O	0.879480	-0.837901	-2.057640
C	-2.149712	0.615288	-0.194467
C	-3.556024	0.374947	-0.065173
C	-4.037805	-0.970693	-0.240922
C	-3.081286	-2.003091	-0.536299
C	-1.751351	-1.729928	-0.678432
C	-4.448923	1.423303	0.242209
C	-5.827553	1.143760	0.377999
C	-6.309661	-0.204770	0.214592
C	-5.409749	-1.251595	-0.091535
C	-6.782441	2.181407	0.640547
C	-8.117957	1.908555	0.755915
C	-8.593331	0.574656	0.602483
C	-7.718831	-0.442618	0.333811
C	-3.932000	2.810580	0.429670
C	-5.912047	-2.647185	-0.259399
C	-5.906552	-3.261106	-1.519924
C	-6.374741	-4.566485	-1.675092
C	-6.855138	-5.273836	-0.571039
C	-6.865195	-4.670625	0.688583
C	-6.396462	-3.365625	0.843067
C	-4.026887	3.438763	1.680510
C	-3.525460	4.726808	1.869073
C	-2.918405	5.404359	0.809262
C	-2.816911	4.788156	-0.439639
C	-3.322434	3.501442	-0.628717
C	0.616042	1.690415	-0.956269
C	1.332227	2.499557	-0.066635
C	1.441952	3.870631	-0.303886
C	0.845244	4.429380	-1.435010
C	0.126390	3.625794	-2.326502
C	0.004360	2.260136	-2.084296

H	1.272755	-3.536455	1.988681
H	3.509987	-2.641962	2.653261
H	2.706944	-1.147970	3.384574
H	0.222107	-0.750315	2.911123
H	-0.679491	-1.994498	1.881692
H	3.874437	0.913433	2.597046
H	5.505391	1.274094	4.435479
H	8.376944	-1.172981	2.361307
H	6.767949	-1.507609	0.510348
H	6.036750	-0.686202	-2.009671
H	6.808079	-2.598404	-3.387856
H	3.907564	-5.150483	-1.487097
H	3.132282	-3.236936	-0.102717
H	5.345575	2.126941	0.564166
H	5.444710	4.185184	-0.794359
H	3.184290	2.254490	-3.907980
H	3.085253	0.185292	-2.554280
H	0.265441	-0.653753	-2.793748
H	-1.783336	1.624797	-0.035726
H	-3.436922	-3.023090	-0.647123
H	-1.045984	-2.527332	-0.908304
H	-6.426773	3.202774	0.736401
H	-8.822838	2.712384	0.951690
H	-9.657560	0.369622	0.684966
H	-8.087860	-1.454439	0.195652
H	-5.531263	-2.707077	-2.378426
H	-6.366322	-5.029609	-2.658643
H	-7.220267	-6.290640	-0.691617
H	-7.236274	-5.216706	1.552318
H	-6.404442	-2.892006	1.823035
H	-4.495726	2.906858	2.506332
H	-3.604531	5.198859	2.845166
H	-2.525335	6.407113	0.956425
H	-2.342019	5.306794	-1.268879
H	-3.235857	3.018597	-1.601220
H	1.816091	2.049765	0.801604
H	2.004857	4.496364	0.383399
H	0.938494	5.495657	-1.625024
H	-0.342645	4.066037	-3.202674
H	-0.574025	1.640223	-2.769026
H	7.756466	0.219974	4.322148
H	5.744929	-4.829205	-3.129792
H	4.352291	4.267456	-3.026472

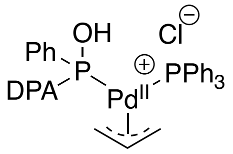

S1

Energy (E) =

-3389.10746879 Hartree (MN15)

Gibbs free energy (G) =

-3388.39282879 Hartree (MN15)

Charge = 0, Spin = 1

C	-1.347320	2.390741	-2.480601
C	-2.541004	2.677013	-1.790455
C	-0.160671	2.201758	-1.741147
Pd	-1.686585	0.831887	-0.978753
P	-3.778539	0.003056	-0.351222
P	-0.436722	-0.910342	-0.139718
C	-3.923799	-1.367312	0.839859
C	-4.729242	1.364466	0.394117
C	-4.714627	-0.577943	-1.792299
C	-4.060611	2.185484	1.318290
C	-4.728276	3.239298	1.939542
C	-6.065779	3.496813	1.625409
C	-6.730411	2.695719	0.696543
C	-6.068130	1.629142	0.083589
C	-5.919386	-1.283886	-1.633315
C	-6.639479	-1.702333	-2.750057
C	-6.157657	-1.432251	-4.034731

C	-4.954791	-0.746075	-4.201718	C	1.342532	4.154041	1.601057
C	-4.233981	-0.320950	-3.083605	C	2.233998	3.088757	1.466789
C	-4.164396	-1.134740	2.200679	C	-0.679234	-1.025833	1.653299
C	-4.236237	-2.205550	3.092366	C	-0.771591	0.176958	2.371561
C	-4.063014	-3.512952	2.636091	C	-0.923030	0.156220	3.757231
C	-3.811736	-3.750244	1.282875	C	-0.985266	-1.067957	4.429213
C	-3.741966	-2.683930	0.387728	C	-0.891328	-2.264218	3.716033
H	-4.118275	-4.344945	3.333653	C	-0.737575	-2.249270	2.327950
H	-6.718064	-1.765221	-4.904400	H	-1.397449	2.033402	-3.509058
H	-6.585609	4.324626	2.100490	H	-3.495918	2.662405	-2.311694
C	1.368414	-0.855981	-0.267691	H	-2.509897	3.256908	-0.865986
O	-0.905457	-2.278427	-0.827995	H	0.006612	2.787775	-0.832530
C	1.959727	0.360688	-0.057305	H	0.731291	1.817725	-2.229832
C	3.376804	0.528214	-0.031551	H	-3.011774	1.991165	1.548860
C	4.198593	-0.632675	-0.248322	H	-4.204267	3.863586	2.658385
C	3.543949	-1.891534	-0.478757	H	-7.768083	2.898571	0.444975
C	2.180487	-2.012742	-0.482765	H	-6.596014	1.013419	-0.640145
C	3.953855	1.802246	0.154524	H	-6.286987	-1.508803	-0.633418
C	5.357099	1.935559	0.139360	H	-7.572218	-2.244290	-2.619500
C	6.187645	0.774013	-0.067260	H	-4.571970	-0.546833	-5.198983
C	5.602920	-0.498092	-0.263808	H	-3.286188	0.200136	-3.212421
C	5.992170	3.203150	0.358162	H	-4.301267	-0.120638	2.568573
C	7.354138	3.324587	0.364890	H	-4.423960	-2.014225	4.145879
C	8.176614	2.177553	0.165495	H	-3.664459	-4.764974	0.921714
C	7.611740	0.948215	-0.036539	H	-3.548168	-2.874048	-0.665266
C	3.059730	2.983804	0.336335	H	-0.579942	-3.175238	-0.490225
C	6.465604	-1.694542	-0.490196	H	1.332819	1.232732	0.098724
C	6.533458	-2.717004	0.467012	H	4.159628	-2.767903	-0.659756
C	7.337908	-3.836949	0.253187	H	1.713119	-2.982204	-0.647190
C	8.085218	-3.948480	-0.921224	H	5.365147	4.073186	0.531214
C	8.024475	-2.935542	-1.880793	H	7.814597	4.294185	0.536090
C	7.219771	-1.816297	-1.666240	H	9.258399	2.281546	0.187404
C	2.983057	3.977623	-0.650277	H	8.244145	0.075420	-0.168696
C	2.097353	5.048622	-0.513824	H	5.949131	-2.627682	1.381010
C	1.271944	5.137295	0.609542	H	7.382074	-4.621133	1.004909

H	8.711931	-4.820964	-1.088008
H	8.602016	-3.017406	-2.798264
H	7.170847	-1.024615	-2.411648
H	3.618908	3.901172	-1.530381
H	2.048627	5.810076	-1.288175
H	0.580892	5.969793	0.714828
H	0.709745	4.220116	2.482934
H	2.283472	2.317880	2.234494
H	-0.723531	1.130704	1.839738
H	-0.996643	1.089485	4.309968
H	-1.110133	-1.087801	5.509024
H	-0.948903	-3.215552	4.239495
H	-0.672230	-3.185295	1.774386
Cl	-0.103998	-5.054265	-0.086822

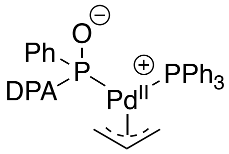

S2

Energy (E) =

-2928.32264869 Hartree (MN15)

Gibbs free energy (G) =

-2927.61883569 Hartree (MN15)

Charge = 0, Spin = 1

C	1.043207	-2.039706	2.716859
C	2.233842	-1.402415	3.089634
C	-0.035396	-1.282097	2.189210
Pd	1.698954	-0.872221	0.977199
P	3.865585	-0.499268	0.209760
P	0.548960	-0.505031	-0.982857
C	4.192670	0.738052	-1.085247
C	4.972135	0.003617	1.563843
C	4.551017	-2.041505	-0.457928
C	4.509267	1.002010	2.436697
C	5.320154	1.469582	3.468910
C	6.596811	0.928459	3.649509
C	7.059655	-0.069273	2.791192
C	6.253879	-0.527620	1.745444
C	5.655942	-2.045794	-1.322970
C	6.173838	-3.253215	-1.790324
C	5.589475	-4.462307	-1.402746
C	4.486971	-4.465687	-0.546379
C	3.967061	-3.258296	-0.077089
C	4.900628	1.919708	-0.833663
C	5.038158	2.881767	-1.837144
C	4.473307	2.669277	-3.094499
C	3.777012	1.485564	-3.353986
C	3.637386	0.523111	-2.356789
C	-1.260239	-0.622136	-0.742803
O	0.934807	-1.466582	-2.107397
C	-1.997419	0.447246	-0.305226
C	-3.410805	0.366481	-0.091656
C	-4.058530	-0.903840	-0.295426
C	-3.251212	-2.014751	-0.717991
C	-1.909973	-1.878790	-0.949728
C	-4.157613	1.492196	0.321022
C	-5.546054	1.364270	0.546503
C	-6.191650	0.087827	0.362722
C	-5.442494	-1.033617	-0.058020
C	-6.357149	2.487552	0.921254
C	-7.703529	2.360621	1.126176
C	-8.339355	1.096825	0.955326
C	-7.607899	0.004122	0.578803
C	-3.469955	2.802235	0.516826
C	-6.115524	-2.350933	-0.259210
C	-6.270927	-2.882843	-1.547280
C	-6.900519	-4.113672	-1.736431
C	-7.384032	-4.828883	-0.638704
C	-7.234418	-4.307925	0.648401
C	-6.603919	-3.077356	0.836248

C	-3.416590	3.396918	1.786296
C	-2.752194	4.609034	1.978498
C	-2.127133	5.243426	0.902901
C	-2.170923	4.659882	-0.365518
C	-2.838744	3.450212	-0.556984
C	0.666493	1.235718	-1.528062
C	1.235782	2.230770	-0.727158
C	1.327130	3.545819	-1.190155
C	0.845119	3.870809	-2.459561
C	0.260559	2.884860	-3.261645
C	0.172219	1.573617	-2.796013
H	1.051743	-3.118257	2.556231
H	3.122633	-1.983715	3.323645
H	2.212442	-0.379306	3.467403
H	-0.224340	-0.273665	2.565077
H	-0.897064	-1.793080	1.767169
H	3.506912	1.411437	2.297897
H	4.955394	2.246317	4.135893
H	8.051065	-0.492315	2.931176
H	6.625072	-1.299570	1.075097
H	6.106395	-1.103295	-1.629448
H	7.030129	-3.251125	-2.459637
H	4.026931	-5.404896	-0.250675
H	3.098235	-3.250831	0.581977
H	5.343304	2.098345	0.142993
H	5.587872	3.797024	-1.631911
H	3.326676	1.318875	-4.329473
H	3.057258	-0.380058	-2.546857
H	-1.501535	1.396166	-0.118485
H	-3.730814	-2.979096	-0.860204
H	-1.318018	-2.725351	-1.292506
H	-5.881432	3.457509	1.031070
H	-8.295532	3.228022	1.407069
H	-9.411606	1.007656	1.110443
H	-8.099589	-0.952277	0.427038
H	-5.892075	-2.323468	-2.400749
H	-7.015205	-4.512613	-2.741324
H	-7.874988	-5.787674	-0.785574
H	-7.606769	-4.860528	1.507544
H	-6.487470	-2.667796	1.838027
H	-3.899590	2.898457	2.624908
H	-2.718825	5.054601	2.969725
H	-1.606913	6.186236	1.052621
H	-1.679754	5.141637	-1.207298
H	-2.859077	2.989769	-1.543881
H	1.629565	1.965254	0.256682
H	1.781444	4.312190	-0.566303
H	0.922049	4.892629	-2.824532
H	-0.118900	3.140162	-4.248424
H	-0.270765	0.797233	-3.419234
H	5.991773	-5.401650	-1.773351
H	7.227342	1.283648	4.460493
H	4.574357	3.423128	-3.871495

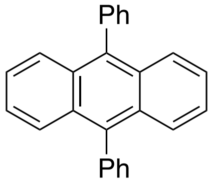

DPA

Energy (E) =

-1000.60875601 Hartree (MN15)

-1001.52080353 Hartree (ωB97XD)

-1001.28581793 Hartree (CAM-B3LYP)

Gibbs free energy (G) =

-1000.29992101 Hartree (MN15)

-1001.21196853 Hartree (ωB97XD)

-1000.97698293 Hartree (CAM-B3LYP)

Charge = 0, Spin = 1

C	0.712660	3.658316	0.237993
C	1.400828	2.479844	0.159261

C	0.720470	1.219973	0.061645
C	-0.720414	1.220025	0.061644
C	-1.400619	2.479967	0.159404
C	-0.712337	3.658389	0.238093
C	1.426982	0.000012	0.000011
C	0.720387	-1.220012	-0.061663
C	-0.720467	-1.219977	-0.061659
C	-1.427009	0.000089	0.000012
C	1.400640	-2.479860	-0.159388
C	0.712383	-3.658340	-0.238171
C	-0.712558	-3.658305	-0.238227
C	-1.400765	-2.479795	-0.159463
C	2.919916	-0.000044	0.000016
C	3.633299	-0.491555	1.102098
C	5.028204	-0.491971	1.102790
C	5.728926	-0.000114	0.000058
C	5.028261	0.491791	-1.102685
C	3.633354	0.491461	-1.102027
C	-2.919943	0.000068	0.000029
C	-3.633336	-0.491345	1.102151
C	-5.028239	-0.491757	1.102844
C	-5.728961	-0.000019	0.000058
C	-5.028296	0.491758	-1.102740
C	-3.633389	0.491430	-1.102078
H	2.486432	2.482783	0.179320
H	-2.486219	2.483039	0.179593
H	-1.250659	4.599494	0.313887
H	2.486242	-2.482959	-0.179452
H	1.250796	-4.599401	-0.313910
H	-1.251008	-4.599340	-0.314024
H	-2.486365	-2.482846	-0.179624
H	3.084409	-0.875932	1.959552
H	5.567416	-0.874655	1.965564
H	6.815814	-0.000145	0.000073
H	5.567515	0.874456	-1.965441
H	3.084506	0.875878	-1.959491
H	-3.084447	-0.875621	1.959652
H	-5.567455	-0.874343	1.965659
H	-6.815849	-0.000055	0.000072
H	-5.567550	0.874310	-1.965546
H	-3.084543	0.875731	-1.959596
H	1.251099	4.599438	0.313681

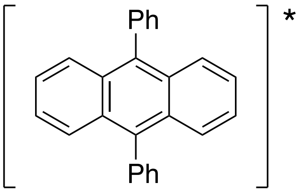

DPA* in the singlet excited state

Energy (E) =

-1000.50110385 Hartree (MN15)

-1001.40584289 Hartree (ωB97XD)

-1001.17175839 Hartree (CAM-B3LYP)

Gibbs free energy (G) =

-1000.19481885 Hartree (MN15)

-1001.09955789 Hartree (ωB97XD)

-1000.86547339 Hartree (CAM-B3LYP)

Charge = 0, Spin = 1

C	0.693299	3.698852	0.301810
C	1.384948	2.479893	0.208477
C	0.721277	1.246801	0.055867
C	-0.721264	1.246810	0.055870
C	-1.384917	2.479911	0.208492
C	-0.693251	3.698861	0.301819
C	1.435029	-0.000014	-0.000007
C	0.721264	-1.246823	-0.055892
C	-0.721277	-1.246815	-0.055891
C	-1.435030	0.000001	-0.000004
C	1.384918	-2.479923	-0.208523
C	0.693254	-3.698872	-0.301865
C	-0.693296	-3.698864	-0.301863

C	-1.384946	-2.479906	-0.208520
C	2.909127	-0.000013	0.000002
C	3.635937	-0.717598	0.971624
C	5.028104	-0.712639	0.974793
C	5.731307	0.000005	0.000032
C	5.028115	0.712644	-0.974741
C	3.635947	0.717588	-0.971599
C	-2.909128	0.000008	0.000006
C	-3.635939	-0.717563	0.971638
C	-5.028106	-0.712604	0.974806
C	-5.731309	0.000022	0.000031
C	-5.028116	0.712641	-0.974756
C	-3.635948	0.717587	-0.971612
H	2.467718	2.490880	0.278459
H	-2.467686	2.490913	0.278488
H	-1.251485	4.626059	0.397107
H	2.467687	-2.490924	-0.278517
H	1.251489	-4.626069	-0.397162
H	-1.251542	-4.626054	-0.397160
H	-2.467715	-2.490895	-0.278512
H	3.091044	-1.265622	1.737387
H	5.566296	-1.262303	1.742867
H	6.818076	0.000012	0.000043
H	5.566316	1.262318	-1.742802
H	3.091064	1.265612	-1.737368
H	-3.091046	-1.265573	1.737411
H	-5.566298	-1.262255	1.742889
H	-6.818078	0.000027	0.000040
H	-5.566316	1.262298	-1.742829
H	-3.091065	1.265592	-1.737394
H	1.251547	4.626043	0.397091

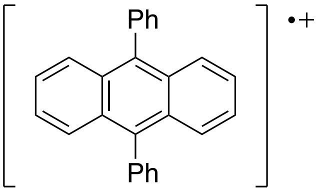

DPA$^{•+}$

Energy (E) =

-1000.40616326 Hartree (MN15)

-1001.31753485 Hartree (ωB97XD)

-1001.08289217 Hartree (CAM-B3LYP)

Gibbs free energy (G) =

-1000.19481885 Hartree (MN15)

-1001.00841785 Hartree (ωB97XD)

-1000.77377517 Hartree (CAM-B3LYP)

Charge = 1, Spin = 2

C	-0.701307	-3.662743	0.287527
C	-1.396602	-2.466960	0.196508
C	-0.718136	-1.231968	0.058561
C	0.718096	-1.231989	0.058564
C	1.396532	-2.467000	0.196491
C	0.701206	-3.662763	0.287514
C	-1.436227	0.000025	-0.000012
C	-0.718101	1.232001	-0.058599
C	0.718133	1.231974	-0.058588
C	1.436218	-0.000017	-0.000004
C	-1.396518	2.467018	-0.196584
C	-0.701177	3.662775	-0.287607
C	0.701335	3.662746	-0.287565
C	1.396616	2.466958	-0.196513
C	-2.915584	0.000031	0.000001
C	-3.624221	0.647731	1.025591
C	-5.016843	0.637037	1.028500
C	-5.714344	0.000016	0.000044
C	-5.016867	-0.637005	-1.028428
C	-3.624245	-0.647689	-1.025559
C	2.915574	-0.000028	0.000010

C	3.624209	0.647865	1.025478
C	5.016832	0.637174	1.028383
C	5.714331	-0.000033	0.000042
C	5.016853	-0.637234	-1.028317
C	3.624230	-0.647917	-1.025445
H	-2.479323	-2.474941	0.253135
H	2.479255	-2.475008	0.253094
H	1.246535	-4.596000	0.386918
H	-2.479236	2.475042	-0.253238
H	-1.246495	4.596013	-0.387052
H	1.246700	4.595961	-0.386972
H	2.479341	2.474926	-0.253093
H	-3.077187	1.140172	1.826147
H	-5.556853	1.127326	1.833325
H	-6.800725	0.000010	0.000060
H	-5.556896	-1.127303	-1.833234
H	-3.077231	-1.140136	-1.826123
H	3.077175	1.140465	1.825934
H	5.556843	1.127616	1.833114
H	6.800712	-0.000036	0.000054
H	5.556880	-1.127676	-1.833038
H	3.077212	-1.140509	-1.825918
H	-1.246661	-4.595963	0.386944

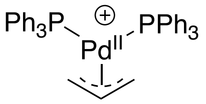

3

Energy (E) =

-2315.79302238 Hartree (MN15)

-2317.65822952 Hartree (ωB97XD)

-2317.18733081 Hartree (CAM-B3LYP)

Gibbs free energy (G) =

-2315.23882738 Hartree (MN15)

-2317.10403452 Hartree (ωB97XD)

-2316.63313581 Hartree (CAM-B3LYP)

Charge = 1, Spin = 1

C	0.021289	-2.175918	-2.997225
C	-1.180166	-1.443034	-3.051739
C	1.244377	-1.474206	-3.036930
Pd	0.044562	-0.656403	-1.416380
P	-1.813888	0.000614	-0.127558
P	1.874238	-0.030840	-0.105080
C	-1.635381	1.381916	1.047454
C	-3.209557	0.505466	-1.179050
C	-2.409591	-1.402453	0.860853
C	-2.939953	1.290334	-2.311811
C	-3.981935	1.742137	-3.120336
C	-5.300741	1.397593	-2.811744
C	-5.574497	0.612343	-1.690766
C	-4.533859	0.170835	-0.871149
C	-3.115749	-1.220222	2.059174
C	-3.545730	-2.324628	2.795468
C	-3.282029	-3.617804	2.338714
C	-2.594732	-3.808432	1.137554
C	-2.159102	-2.705559	0.403578
C	-2.051743	2.677354	0.707981
C	-1.772663	3.751147	1.554689
C	-1.075513	3.543752	2.745948
C	-0.677059	2.253014	3.100535
C	-0.960207	1.176503	2.260892
C	3.483056	-0.678223	-0.658640
C	4.097075	-0.080464	-1.771622
C	5.292355	-0.591042	-2.272018
C	5.883782	-1.705818	-1.668780
C	5.276512	-2.304231	-0.565187
C	4.078309	-1.793103	-0.058384
C	1.653896	-0.682087	1.576288
C	2.096751	-0.016679	2.726842
C	1.779715	-0.521044	3.988885

C	1.017706	-1.686730	4.111247
C	0.593270	-2.369163	2.968226
C	0.918847	-1.870550	1.706656
C	2.162550	1.753093	0.024367
C	1.177269	2.641274	-0.423057
C	1.364079	4.019899	-0.304193
C	2.546543	4.515603	0.244940
C	3.548200	3.635832	0.668462
C	3.361437	2.260155	0.554679
H	0.008376	-3.203696	-2.635353
H	-2.132415	-1.928157	-2.848676
H	-1.229483	-0.527878	-3.643467
H	1.331872	-0.561415	-3.630224
H	2.174182	-1.990841	-2.814456
H	-1.908089	1.548537	-2.556233
H	-3.765021	2.353073	-3.992449
H	-6.599575	0.342499	-1.451016
H	-4.754075	-0.435945	0.004408
H	-3.321167	-0.215480	2.422193
H	-4.085777	-2.173774	3.726257
H	-2.389828	-4.812880	0.777056
H	-1.604224	-2.856241	-0.522150
H	-2.585233	2.855485	-0.222667
H	-2.099629	4.750489	1.279160
H	-0.145998	2.076052	4.032954
H	-0.668785	0.172311	2.561740
H	3.635968	0.786706	-2.244423
H	5.735119	-3.167938	-0.090882
H	3.613102	-2.261942	0.805944
H	2.651689	0.914354	2.644541
H	2.112652	0.007635	4.878276
H	0.754496	-2.061148	5.097019
H	-0.007153	-3.272167	3.052574
H	0.572215	-2.388629	0.812517
H	0.264252	2.248792	-0.867844
H	0.587340	4.700417	-0.643406
H	2.695238	5.588471	0.335521
H	4.475212	4.023008	1.082634
H	4.148373	1.576308	0.869368
H	-6.113516	1.739619	-3.447044
H	-3.613055	-4.476159	2.917365
H	-0.852157	4.382401	3.400268
H	5.762914	-0.120708	-3.131404
H	6.816194	-2.104387	-2.059864

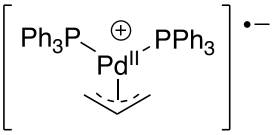

3•−

Energy (E) =

-2315.89824467 Hartree (MN15)

-2317.75981649 Hartree (ωB97XD)

-2317.29226212 Hartree (CAM-B3LYP)

Gibbs free energy (G) =

-2315.35167167 Hartree (MN15)

-2317.21324349 Hartree (ωB97XD)

-2316.74568912 Hartree (CAM-B3LYP)

Charge = 0, Spin = 2

C	-0.317660	-3.525017	-1.211462
C	0.950043	-3.089236	-1.678809
C	-0.545925	-3.871683	0.131497
Pd	0.016264	-1.448244	-0.512518
P	1.895644	-0.116045	-0.043958
P	-1.919506	-0.172274	-0.084078
C	1.738839	1.034425	1.368665
C	3.399504	-1.063138	0.388275
C	2.432898	0.939909	-1.432898
C	3.230430	-2.297088	1.034711
C	4.335864	-3.044837	1.443825
C	5.625403	-2.571933	1.191907

C	5.804571	-1.349151	0.540396
C	4.698339	-0.595549	0.144994
C	3.083094	2.171116	-1.264705
C	3.453130	2.932780	-2.374583
C	3.186930	2.468067	-3.664593
C	2.552620	1.235960	-3.843625
C	2.174786	0.480080	-2.733439
C	2.151183	0.653697	2.654917
C	1.835703	1.441018	3.764368
C	1.106219	2.621133	3.605623
C	0.704915	3.015973	2.327302
C	1.018568	2.231223	1.217964
C	-3.466005	-0.692800	-0.908660
C	-3.797836	-2.056658	-0.867829
C	-4.972469	-2.520882	-1.457884
C	-5.825739	-1.626446	-2.110395
C	-5.502595	-0.269533	-2.161077
C	-4.330683	0.197484	-1.560379
C	-1.690638	1.544226	-0.663939
C	-2.162009	2.672872	0.019411
C	-1.830233	3.954452	-0.427626
C	-1.026723	4.119925	-1.559033
C	-0.571652	2.999823	-2.261304
C	-0.908460	1.722170	-1.815139
C	-2.445009	0.032400	1.652469
C	-1.456664	-0.032275	2.645449
C	-1.783142	0.178147	3.986062
C	-3.106523	0.436515	4.348436
C	-4.100640	0.490235	3.367437
C	-3.772542	0.291973	2.025518
H	-1.160456	-3.550129	-1.904553
H	1.088696	-2.897791	-2.741449
H	1.849658	-3.376162	-1.133941
H	0.287246	-4.004865	0.818642
H	-1.531366	-4.164251	0.481102

H	2.217953	-2.662970	1.212626
H	4.190565	-3.996983	1.947936
H	6.807714	-0.979649	0.342201
H	4.846709	0.358748	-0.356589
H	3.291866	2.541049	-0.262645
H	3.950231	3.888923	-2.231095
H	2.343596	0.869321	-4.845609
H	1.654057	-0.469734	-2.861033
H	2.710560	-0.269286	2.794986
H	2.162351	1.130004	4.753972
H	0.139481	3.934784	2.186346
H	0.701179	2.553906	0.227989
H	-3.122289	-2.750795	-0.369001
H	-6.164550	0.429800	-2.666037
H	-4.090875	1.258371	-1.594876
H	-2.766180	2.553132	0.916590
H	-2.189216	4.824799	0.116595
H	-0.754522	5.119029	-1.890419
H	0.067354	3.115165	-3.134650
H	-0.525572	0.842656	-2.333143
H	-0.427366	-0.244155	2.358484
H	-1.002237	0.137420	4.742056
H	-3.365403	0.593167	5.392768
H	-5.132334	0.688377	3.647648
H	-4.548895	0.344143	1.263848
H	6.489245	-3.156050	1.499243
H	3.473158	3.064173	-4.527555
H	0.855998	3.229957	4.470871
H	-5.218207	-3.579180	-1.416643
H	-6.737755	-1.987163	-2.579366

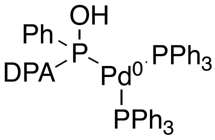

4

Energy (E) =

-3847.27545878 Hartree (MN15)

-3850.28629515 Hartree (ωB97XD)

-3849.54013148 Hartree (CAM-B3LYP)

Gibbs free energy (G) =

-3846.37124678 Hartree (MN15)

-3849.38208315 Hartree (ωB97XD)

-3848.63591948 Hartree (CAM-B3LYP)

Charge = 0, Spin = 1

P	0.684372	-1.540412	1.384541
Pd	1.464985	0.428352	0.327217
P	0.015239	2.182744	0.705939
O	-0.077003	2.579553	2.310888
H	-0.717684	3.288810	2.507003
H	0.780711	-3.231309	-3.781741
C	-0.116870	-2.760422	-3.386422
C	-0.407336	-1.429314	-3.694960
H	-0.753805	-4.516250	-2.304042
C	-0.979421	-3.482449	-2.558286
C	-1.533608	-0.812121	-3.148570
C	-2.115336	-2.869480	-2.031517
C	-2.393627	-1.519044	-2.294706
H	-4.378214	-2.842597	-3.246028
H	-2.790731	-3.429390	-1.387833
H	-1.115231	0.082896	-0.796522
C	-5.177714	-2.399775	-2.659442
C	-3.539332	-0.835635	-1.631431
C	-1.958457	0.698019	-0.481685
H	-6.663959	-3.721438	-3.427319
C	-6.451668	-2.887887	-2.762699
C	-4.864046	-1.300400	-1.792852
C	-3.291522	0.277334	-0.799449
C	-1.707542	1.817108	0.268118
C	-7.503305	-2.308489	-1.996493
C	-5.943254	-0.661154	-1.082310
C	-4.381530	0.985138	-0.181281
C	-2.801095	2.611435	0.736742
C	-4.086117	2.193261	0.541458
C	-5.696540	0.492185	-0.301187
C	-7.255662	-1.230477	-1.190951
H	-2.611549	3.547217	1.263962
H	-5.999645	0.804687	2.365310
C	-6.824235	1.249189	1.809332
C	-7.865749	1.886959	2.483298
H	-7.859715	1.930491	3.569613
H	0.243036	-0.866324	-4.361150
H	-1.747154	0.231997	-3.371581
H	-8.507000	-2.721189	-2.059730
H	-8.062888	-0.788388	-0.614688
H	-7.867464	1.718739	-1.391507
H	-4.913012	2.783059	0.926171
C	-6.814766	1.178108	0.407986
C	-7.870123	1.766842	-0.304101
C	-8.911929	2.406528	0.368308
H	-9.721432	2.860455	-0.197984
C	-8.913066	2.466552	1.763652
H	-9.725238	2.964217	2.287623
C	5.574023	-1.318989	2.469396
C	4.659484	-0.977095	1.475021
C	6.013975	0.930227	0.868719
H	3.766933	-1.584348	1.323461
C	4.867876	0.151136	0.662847
H	6.197153	1.803825	0.247045
P	3.598177	0.525632	-0.602053
C	4.192749	2.103752	-1.316214
H	5.789599	-1.500153	-1.104145
C	5.080486	-1.530746	-1.928728
C	5.166950	2.168912	-2.323945
C	3.986522	-0.656751	-1.942818
C	5.593815	3.404592	-2.812928

H	5.593731	1.250967	-2.724704
C	5.265867	-2.447102	-2.968753
C	3.084450	-0.712983	-3.017771
C	4.367688	-2.491758	-4.035989
C	3.276448	-1.616537	-4.061364
H	4.514668	-3.203639	-4.844291
H	2.573684	-1.645938	-4.891798
H	6.116917	-3.123417	-2.944609
H	7.419399	-0.785505	3.455913
H	5.389480	-2.190671	3.092998
C	6.709148	-0.528559	2.673831
H	7.809220	1.209066	2.025543
C	6.926699	0.592570	1.872330
H	2.901522	3.245819	-0.028202
C	3.664809	3.296695	-0.803650
H	3.663815	5.447519	-0.884693
C	4.094525	4.533503	-1.287002
C	5.056891	4.587566	-2.297409
H	5.387149	5.548071	-2.685605
H	6.345710	3.444440	-3.597462
H	2.217350	-0.050296	-3.017549
H	1.193577	7.027615	0.614599
C	0.860455	6.169085	0.036183
H	0.813451	4.857649	1.747369
C	0.637396	4.946143	0.677133
H	0.835748	7.237015	-1.837230
C	0.658777	6.287000	-1.339278
C	0.198866	3.834827	-0.053097
C	0.230940	5.177621	-2.077069
C	0.003341	3.961154	-1.437957
H	0.072877	5.264374	-3.149303
H	-0.328283	3.095971	-2.014796
H	2.166783	-2.234020	-0.917493
H	2.836844	-4.364149	-2.002832
C	2.229864	-4.387123	-1.099495

C	1.861990	-3.185977	-0.486821
C	1.078984	-3.176418	0.674488
C	0.652446	-4.405448	1.209982
C	1.007457	-5.605614	0.595979
H	0.032288	-4.419702	2.105462
C	1.800046	-5.598271	-0.558499
H	2.075117	-6.537704	-1.031867
H	0.669348	-6.548895	1.017646
H	2.488817	0.820533	5.313218
H	1.280089	0.621439	3.159808
C	2.380980	-0.154782	4.844317
C	1.689820	-0.269265	3.637726
C	2.948256	-1.285282	5.437226
H	3.496937	-1.195998	6.371565
C	1.533257	-1.521139	3.014120
C	2.815336	-2.528693	4.817474
C	2.113446	-2.646761	3.614916
H	3.260671	-3.413002	5.267470
H	2.035997	-3.621729	3.141173
H	-0.998618	0.145342	3.027988
H	-3.425447	0.248150	3.452741
C	-1.647768	-0.625911	2.614510
C	-3.020928	-0.556655	2.841773
C	-1.098244	-1.646107	1.818161
C	-3.879116	-1.498805	2.264196
H	-4.953365	-1.436622	2.425025
C	-1.968137	-2.585753	1.251469
C	-3.347504	-2.510171	1.467160
H	-1.574648	-3.375100	0.618803
H	-4.004746	-3.241249	0.999408

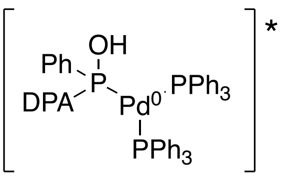

4* in the singlet excited state

Energy (E) =

-3847.19410316 Hartree (MN15)

-3850.22118408 Hartree (ωB97XD)

-3849.44501796 Hartree (CAM-B3LYP)

Gibbs free energy (G) =

-3846.28942216 Hartree (MN15)

-3849.32040008 Hartree (ωB97XD)

-3848.54033696 Hartree (CAM-B3LYP)

Charge = 0, Spin = 1

P	0.988461	-1.173569	1.459292
Pd	1.458643	0.695144	0.112336
P	-0.245774	2.103709	0.731106
O	-0.260978	2.700967	2.275717
H	-0.921250	3.403427	2.431728
H	0.628078	-3.144047	-3.744487
C	-0.285956	-2.698299	-3.357886
C	-0.647236	-1.400696	-3.728503
H	-0.810568	-4.421196	-2.168296
C	-1.093653	-3.416343	-2.472472
C	-1.802669	-0.816862	-3.203958
C	-2.249710	-2.833988	-1.953680
C	-2.610476	-1.522352	-2.301279
H	-4.547178	-2.836725	-3.330366
H	-2.876572	-3.385775	-1.253793
H	-1.378200	0.136356	-0.911093
C	-5.370818	-2.420433	-2.756934
C	-3.779159	-0.875822	-1.639869
C	-2.228474	0.638948	-0.449896
H	-6.828372	-3.710752	-3.628225
C	-6.639862	-2.905268	-2.922993
C	-5.092414	-1.356527	-1.837359
C	-3.553897	0.192190	-0.747076
C	-1.975495	1.646654	0.446119
C	-7.717851	-2.358611	-2.169922
C	-6.190148	-0.769053	-1.111937
C	-4.652509	0.803724	-0.047223
C	-3.079925	2.314217	1.068490
C	-4.361232	1.905064	0.831014
C	-5.959454	0.310352	-0.224699
C	-7.497789	-1.326401	-1.297555
H	-2.911300	3.150992	1.744605
H	-6.343991	0.215616	2.443336
C	-7.142223	0.760299	1.940768
C	-8.194234	1.309047	2.674177
H	-8.221470	1.187798	3.754282
H	-0.029343	-0.838196	-4.426007
H	-2.073176	0.202641	-3.475127
H	-8.719521	-2.763054	-2.291780
H	-8.324253	-0.914418	-0.725949
H	-8.080205	1.724745	-1.180879
H	-5.188147	2.412545	1.319399
C	-7.090259	0.903606	0.546555
C	-8.115007	1.610229	-0.098995
C	-9.168312	2.159862	0.633028
H	-9.954978	2.707013	0.119330
C	-9.210480	2.010091	2.020943
H	-10.031234	2.438146	2.591035
C	5.721401	-1.828953	2.100808
C	4.891807	-1.437634	1.052147
C	5.714713	0.826753	1.239764
H	4.241265	-2.173619	0.578672
C	4.884842	-0.106040	0.602138
H	5.715027	1.864773	0.912968
P	3.661773	0.404789	-0.652033
C	4.321718	1.982483	-1.292823
H	5.910429	-1.472928	-1.411442
C	5.145222	-1.509849	-2.183862
C	5.579074	2.031961	-1.914208
C	3.991339	-0.723844	-2.066399

C	6.082062	3.242760	-2.388920
H	6.165527	1.120104	-2.020907
C	5.316958	-2.352067	-3.285911
C	3.019307	-0.786766	-3.076800
C	4.344207	-2.409077	-4.285266
C	3.196151	-1.617271	-4.182787
H	4.478970	-3.066506	-5.140493
H	2.436595	-1.653357	-4.961401
H	6.212999	-2.963067	-3.361630
H	7.193173	-1.196800	3.547496
H	5.709052	-2.861721	2.440693
C	6.550487	-0.893327	2.724689
H	7.183881	1.168586	2.774514
C	6.542426	0.434059	2.293318
H	2.599744	3.122307	-0.672889
C	3.574700	3.159806	-1.160884
H	3.484634	5.277955	-1.533966
C	4.076203	4.372401	-1.641185
C	5.329505	4.413365	-2.254322
H	5.721771	5.355978	-2.628148
H	7.058764	3.273571	-2.865354
H	2.106790	-0.195312	-2.967093
H	1.154012	6.810616	-0.065195
C	0.765790	5.897525	-0.509928
H	0.666225	4.847696	1.372840
C	0.483461	4.795653	0.301884
H	0.786780	6.677413	-2.520842
C	0.559233	5.822050	-1.889475
C	-0.017017	3.614257	-0.261584
C	0.059205	4.646687	-2.458429
C	-0.232241	3.548828	-1.649330
H	-0.107253	4.588425	-3.531147
H	-0.626662	2.630895	-2.087193
H	2.268420	-2.044614	-0.931763
H	2.673542	-4.241581	-1.989511
C	2.065742	-4.182264	-1.087935
C	1.854581	-2.942282	-0.484013
C	1.091741	-2.822015	0.682749
C	0.482410	-3.972881	1.211514
C	0.669785	-5.212029	0.596532
H	-0.141287	-3.900178	2.100729
C	1.473038	-5.322914	-0.543571
H	1.621719	-6.293754	-1.009848
H	0.192994	-6.095649	1.013297
H	4.059027	0.781867	4.799021
H	2.571410	0.814942	2.802551
C	3.573676	-0.133907	4.470004
C	2.733714	-0.111646	3.356530
C	3.808185	-1.334603	5.142182
H	4.471669	-1.357381	6.003416
C	2.106834	-1.284194	2.910184
C	3.197662	-2.509920	4.698815
C	2.350781	-2.486097	3.590062
H	3.381420	-3.448882	5.215520
H	1.887829	-3.410389	3.253690
H	-0.045597	0.070648	3.891775
H	-2.338908	0.354460	4.782563
C	-0.888303	-0.387052	3.376664
C	-2.180440	-0.223534	3.875397
C	-0.669264	-1.127856	2.205433
C	-3.267053	-0.800467	3.212277
H	-4.273550	-0.671698	3.604596
C	-1.765537	-1.700588	1.545484
C	-3.057745	-1.542847	2.049403
H	-1.611286	-2.251220	0.619082
H	-3.900775	-1.976762	1.513820

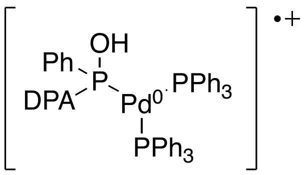

$4^{\bullet+}$

Energy (E) =

-3847.11461861 Hartree (MN15)

-3850.13359514 Hartree (ωB97XD)

-3849.38761156 Hartree (CAM-B3LYP)

Gibbs free energy (G) =

-3846.20957761 Hartree (MN15)

-3849.22855414 Hartree (ωB97XD)

-3848.48257056 Hartree (CAM-B3LYP)

Charge = 1, Spin = 2

P	1.024123	-1.149904	1.571151
Pd	1.450750	0.591445	0.044023
P	-0.242429	2.075281	0.593327
O	-0.115687	2.623937	2.124065
H	-0.898607	3.090461	2.470711
H	0.616247	-3.591336	-3.330298
C	-0.293766	-3.105266	-2.984957
C	-0.653602	-1.851687	-3.485943
H	-0.821768	-4.702627	-1.632249
C	-1.102700	-3.731236	-2.032653
C	-1.802579	-1.212871	-3.016326
C	-2.260182	-3.099504	-1.577748
C	-2.610317	-1.824536	-2.047484
H	-4.558736	-3.222823	-2.933512
H	-2.892500	-3.580828	-0.832354
H	-1.362620	-0.096938	-0.757957
C	-5.379889	-2.729539	-2.421189
C	-3.772573	-1.101444	-1.458672
C	-2.205268	0.495518	-0.401173
H	-6.853278	-4.082611	-3.159064
C	-6.656106	-3.206298	-2.547118
C	-5.092616	-1.574641	-1.622321
C	-3.537813	0.052062	-0.685604
C	-1.962176	1.615601	0.348585
C	-7.731399	-2.558676	-1.873756
C	-6.185971	-0.888041	-0.983054
C	-4.631548	0.767762	-0.084216
C	-3.056077	2.392403	0.850604
C	-4.338130	1.970936	0.649214
C	-5.946858	0.284829	-0.227658
C	-7.502659	-1.438491	-1.121290
H	-2.871373	3.321427	1.389116
H	-6.400005	0.549412	2.423816
C	-7.168588	1.041977	1.829901
C	-8.223877	1.707568	2.453961
H	-8.281537	1.731043	3.539310
H	-0.042605	-1.366818	-4.244602
H	-2.072962	-0.226118	-3.388742
H	-8.738783	-2.957220	-1.961953
H	-8.327008	-0.949580	-0.610942
H	-8.003147	1.605066	-1.417648
H	-5.165930	2.553571	1.042211
C	-7.079178	0.998366	0.431065
C	-8.068007	1.636164	-0.331610
C	-9.123569	2.302765	0.291416
H	-9.882073	2.795211	-0.311947
C	-9.203881	2.339364	1.685270
H	-10.026644	2.858377	2.170472
C	5.856476	-1.514181	2.122863
C	4.955605	-1.244559	1.096711
C	5.814603	1.013835	0.928557
H	4.264311	-2.020619	0.766224
C	4.931455	0.021329	0.487492
H	5.806157	1.997827	0.465312
P	3.666793	0.351306	-0.772755
C	4.121771	1.958894	-1.495587

H	5.938088	-1.466818	-1.507083
C	5.132191	-1.598308	-2.225674
C	4.998295	2.062600	-2.584877
C	3.955604	-0.848891	-2.104449
C	5.324861	3.316989	-3.101671
H	5.422827	1.164525	-3.029117
C	5.267863	-2.521759	-3.265144
C	2.919135	-1.029268	-3.034936
C	4.235925	-2.695051	-4.189072
C	3.061878	-1.943204	-4.077404
H	4.343798	-3.415758	-4.995315
H	2.258813	-2.077743	-4.798571
H	6.180940	-3.104625	-3.351903
H	7.429682	-0.724770	3.371031
H	5.863336	-2.495066	2.591012
C	6.733279	-0.517300	2.562498
H	7.392203	1.519472	2.303528
C	6.710260	0.742985	1.966536
H	2.903701	3.049479	-0.078787
C	3.586645	3.125928	-0.926593
H	3.490158	5.273982	-0.998396
C	3.919198	4.379032	-1.441291
C	4.784948	4.473460	-2.533368
H	5.038219	5.447872	-2.943096
H	6.001396	3.390899	-3.949110
H	1.993278	-0.461476	-2.920097
H	0.705988	6.873526	-0.319419
C	0.499032	5.893644	-0.742124
H	0.286324	4.940895	1.185965
C	0.257651	4.809768	0.106860
H	0.669981	6.563109	-2.783282
C	0.476910	5.718724	-2.126727
C	-0.016686	3.550284	-0.436540
C	0.213722	4.457115	-2.672115
C	-0.026915	3.374421	-1.830187
H	0.202582	4.319842	-3.750163
H	-0.215537	2.383022	-2.249002
H	2.275392	-2.187638	-0.786502
H	2.723468	-4.463086	-1.637637
C	2.157743	-4.335642	-0.716471
C	1.916378	-3.049043	-0.231900
C	1.199577	-2.848239	0.953526
C	0.676381	-3.962954	1.631827
C	0.901542	-5.248001	1.139837
H	0.089189	-3.824141	2.537989
C	1.651864	-5.437499	-0.026782
H	1.828852	-6.443407	-0.398568
H	0.495221	-6.105225	1.669938
H	4.053429	1.494221	4.414213
H	2.449827	1.146306	2.546927
C	3.632657	0.507365	4.239402
C	2.730402	0.312626	3.193897
C	4.005967	-0.570062	5.045684
H	4.717428	-0.422091	5.854207
C	2.190532	-0.960920	2.952408
C	3.473634	-1.839710	4.809349
C	2.569302	-2.037238	3.765816
H	3.766602	-2.679355	5.434352
H	2.169023	-3.031474	3.580864
H	-0.048014	0.360991	3.827029
H	-2.362725	0.753230	4.611324
C	-0.874720	-0.176074	3.366748
C	-2.179780	0.050273	3.802513
C	-0.628136	-1.073922	2.316980
C	-3.248192	-0.623510	3.203897
H	-4.265182	-0.440347	3.543163
C	-1.703258	-1.756687	1.730634
C	-3.006832	-1.536058	2.175798
H	-1.523920	-2.441437	0.904281
H	-3.834407	-2.055667	1.695874

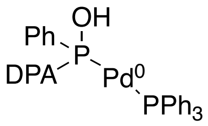

S3

Energy (E) =

-2811.72803867 Hartree (MN15)

Gibbs free energy (G) =

-2811.08423667 Hartree (MN15)

Charge = 0, Spin = 1

Pd	-1.816537	1.175550	-0.505769
P	-0.002205	2.487756	-0.825752
C	1.583087	1.634861	-0.649736
O	0.222561	3.227755	-2.282454
C	1.568749	0.300981	-0.331142
C	2.777288	-0.442852	-0.126848
C	4.032252	0.239329	-0.312417
C	4.006275	1.631237	-0.676225
C	2.832666	2.312139	-0.822601
C	2.747861	-1.809982	0.218653
C	3.963758	-2.513518	0.379942
C	5.220043	-1.835775	0.181012
C	5.243069	-0.464386	-0.162022
C	3.990419	-3.890995	0.779903
C	5.170093	-4.562969	0.948561
C	6.411199	-3.895839	0.740990
C	6.432092	-2.576827	0.378207
P	-3.642774	-0.190729	-0.195469
C	-3.693022	-0.980030	1.446567
C	-3.189928	-0.246034	2.531995
C	-3.198274	-0.788402	3.817324
C	-3.693163	-2.079294	4.024944
C	-4.186857	-2.820115	2.947929
C	-4.190867	-2.271842	1.663317
C	-3.720672	-1.583827	-1.367997
C	-4.923938	-2.144612	-1.819958
C	-4.907132	-3.224789	-2.703895
C	-3.690410	-3.754542	-3.140635
C	-2.487861	-3.203466	-2.692767
C	-2.503045	-2.118386	-1.815893
C	-5.266343	0.621501	-0.353454
C	-5.366593	1.704299	-1.240327
C	-6.589037	2.347396	-1.435302
C	-7.719368	1.921007	-0.733933
C	-7.626216	0.850103	0.158013
C	-6.404969	0.201120	0.348283
C	0.185045	3.880754	0.327031
C	0.730741	5.108348	-0.070198
C	0.881766	6.139095	0.859217
C	0.493221	5.948154	2.187270
C	-0.055905	4.727062	2.587912
C	-0.216625	3.699503	1.658488
C	1.441983	-2.503219	0.424282
C	1.049411	-3.553945	-0.419190
C	-0.161843	-4.215805	-0.213363
C	-1.003592	-3.828202	0.831519
C	-0.627187	-2.779476	1.673622
C	0.588383	-2.123562	1.471411
C	6.546239	0.238632	-0.352667
C	7.362035	-0.052912	-1.455133
C	8.579159	0.606497	-1.631051
C	8.995715	1.566710	-0.706389
C	8.190270	1.864159	0.394991
C	6.973765	1.203705	0.570386
H	-0.600272	3.224265	-2.799345
H	0.608905	-0.205784	-0.237631
H	4.951730	2.141917	-0.833950
H	2.838301	3.368386	-1.086217
H	3.047605	-4.398213	0.960618
H	5.163480	-5.605785	1.255131

H	7.343709	-4.435000	0.887086
H	7.380488	-2.065479	0.242853
H	-2.780121	0.749617	2.354460
H	-2.807442	-0.212280	4.652175
H	-3.689596	-2.508849	5.023513
H	-4.569278	-3.825175	3.107347
H	-4.572280	-2.853974	0.825986
H	-5.874493	-1.735251	-1.482746
H	-5.844195	-3.651797	-3.052195
H	-3.680293	-4.593831	-3.831552
H	-1.537797	-3.612636	-3.028028
H	-1.566711	-1.677913	-1.466888
H	-4.472989	2.039453	-1.768833
H	-6.657114	3.185673	-2.123986
H	-8.671012	2.426425	-0.877673
H	-8.504389	0.519986	0.707114
H	-6.336239	-0.633343	1.044236
H	1.022725	5.253780	-1.108035
H	1.301702	7.091978	0.546584
H	0.610554	6.753274	2.908324
H	-0.368584	4.581046	3.618787
H	-0.664912	2.749487	1.956089
H	1.706792	-3.854376	-1.233151
H	-0.452806	-5.027567	-0.875877
H	-1.954168	-4.333462	0.987852
H	-1.278197	-2.476571	2.490840
H	0.887346	-1.310021	2.130946
H	7.035177	-0.802597	-2.173398
H	9.200725	0.371733	-2.491635
H	9.943729	2.080783	-0.843664
H	8.509529	2.609342	1.119437
H	6.342878	1.434061	1.426987

PPh_3

Energy (E) =

-1035.49904475 Hartree (MN15)

Gibbs free energy (G) =

-1035.27351175 Hartree (MN15)

Charge = 0, Spin = 1

P	-0.006555	0.000854	-1.226025
C	0.772553	1.450463	-0.424025
C	0.610222	2.692039	-1.057093
C	1.151170	3.851654	-0.502804
C	1.877826	3.781087	0.687356
C	2.054455	2.549900	1.321528
C	1.503681	1.391561	0.771089
C	0.869485	-1.397708	-0.433621
C	0.435901	-2.043323	0.732456
C	1.174915	-3.096485	1.274611
C	2.358106	-3.512651	0.662359
C	2.799775	-2.876433	-0.499557
C	2.055061	-1.832312	-1.046428
C	-1.651691	-0.054693	-0.426029
C	-2.657577	-0.777994	-1.084280
C	-3.933031	-0.891213	-0.531292
C	-4.222707	-0.267302	0.683511
C	-3.232566	0.463377	1.343356
C	-1.954237	0.567814	0.793555
H	0.058263	2.745758	-1.994478
H	1.014698	4.806618	-1.003717
H	2.308453	4.681826	1.117143
H	2.620630	2.490832	2.247719
H	1.640326	0.436023	1.273516
H	-0.481735	-1.721003	1.219979
H	0.826446	-3.590307	2.178250
H	2.931399	-4.333165	1.086083
H	3.717312	-3.199536	-0.984576
H	2.391905	-1.348596	-1.962407
H	-2.437036	-1.250869	-2.040247
H	-4.701382	-1.456794	-1.052095

H	-5.218106	-0.346518	1.112864
H	-3.455171	0.951685	2.288761
H	-1.185720	1.134546	1.315434

2. Supplementary Figures

2-1. NMR spectra

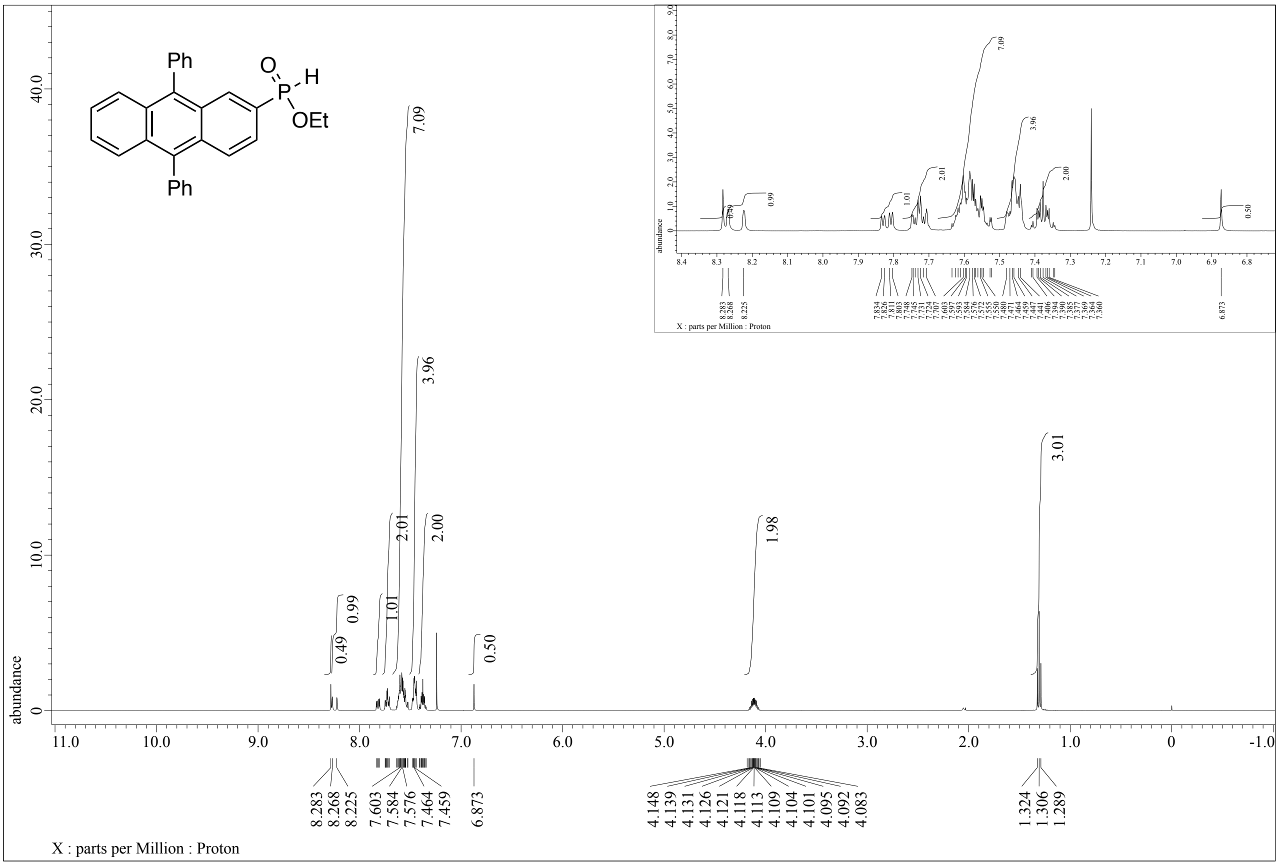

Supplementary Figure 44. ^{1}H NMR spectrum of **6** ($CDCl_3$, 399.8 MHz, 19.6 ºC).

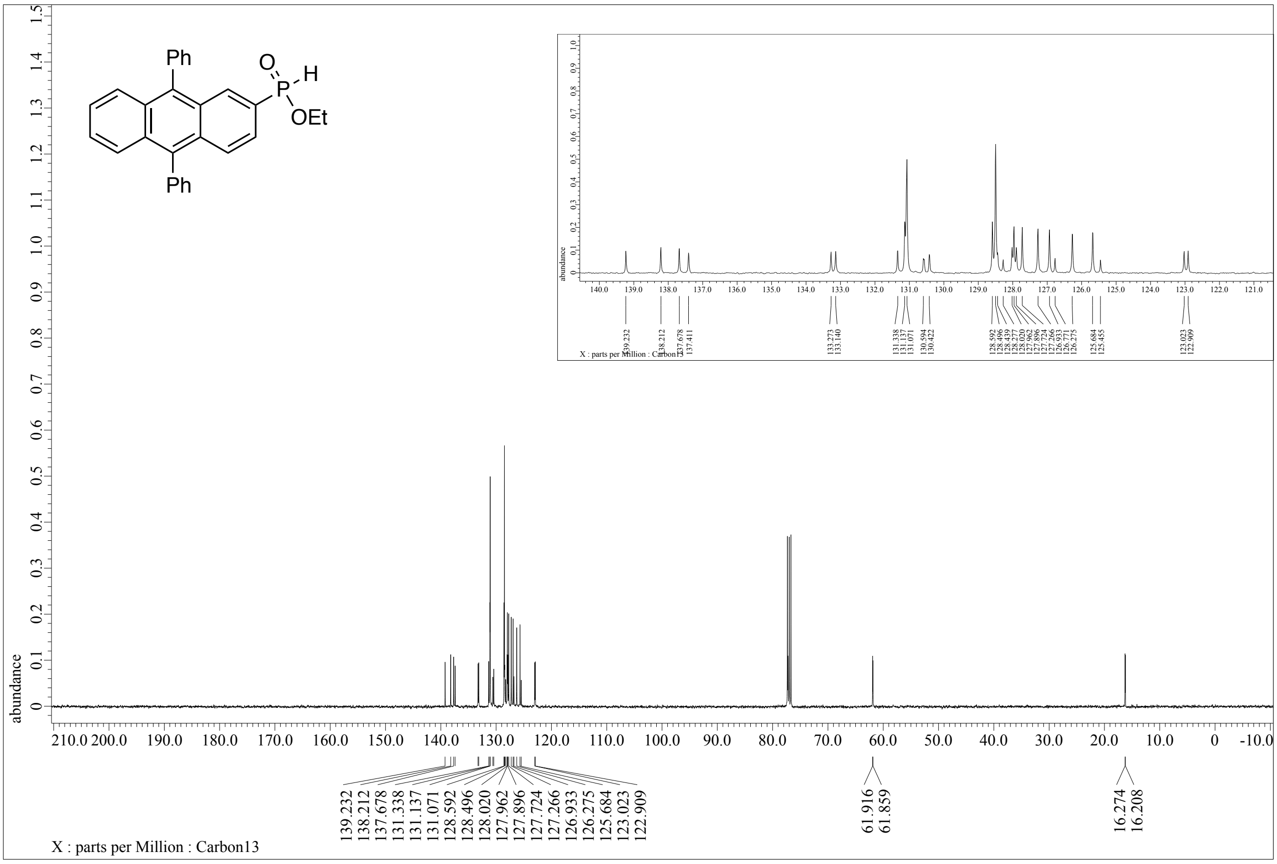

Supplementary Figure 45. ^{13}C NMR spectrum of **6** ($CDCl_3$, 100.5 MHz, 19.8 ºC).

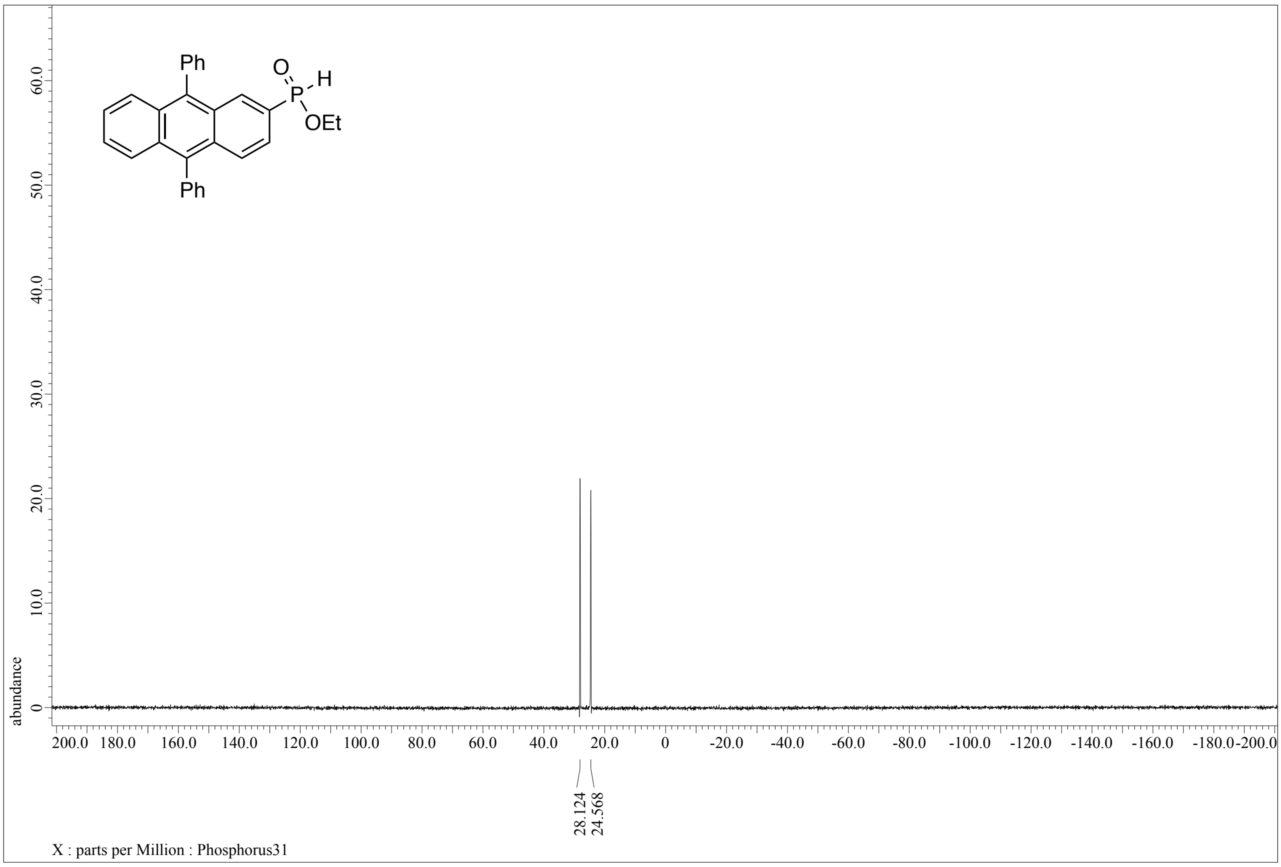

Supplementary Figure 46. ^{31}P NMR spectrum of **6** ($CDCl_3$, 161.8 MHz, 19.8 ºC).

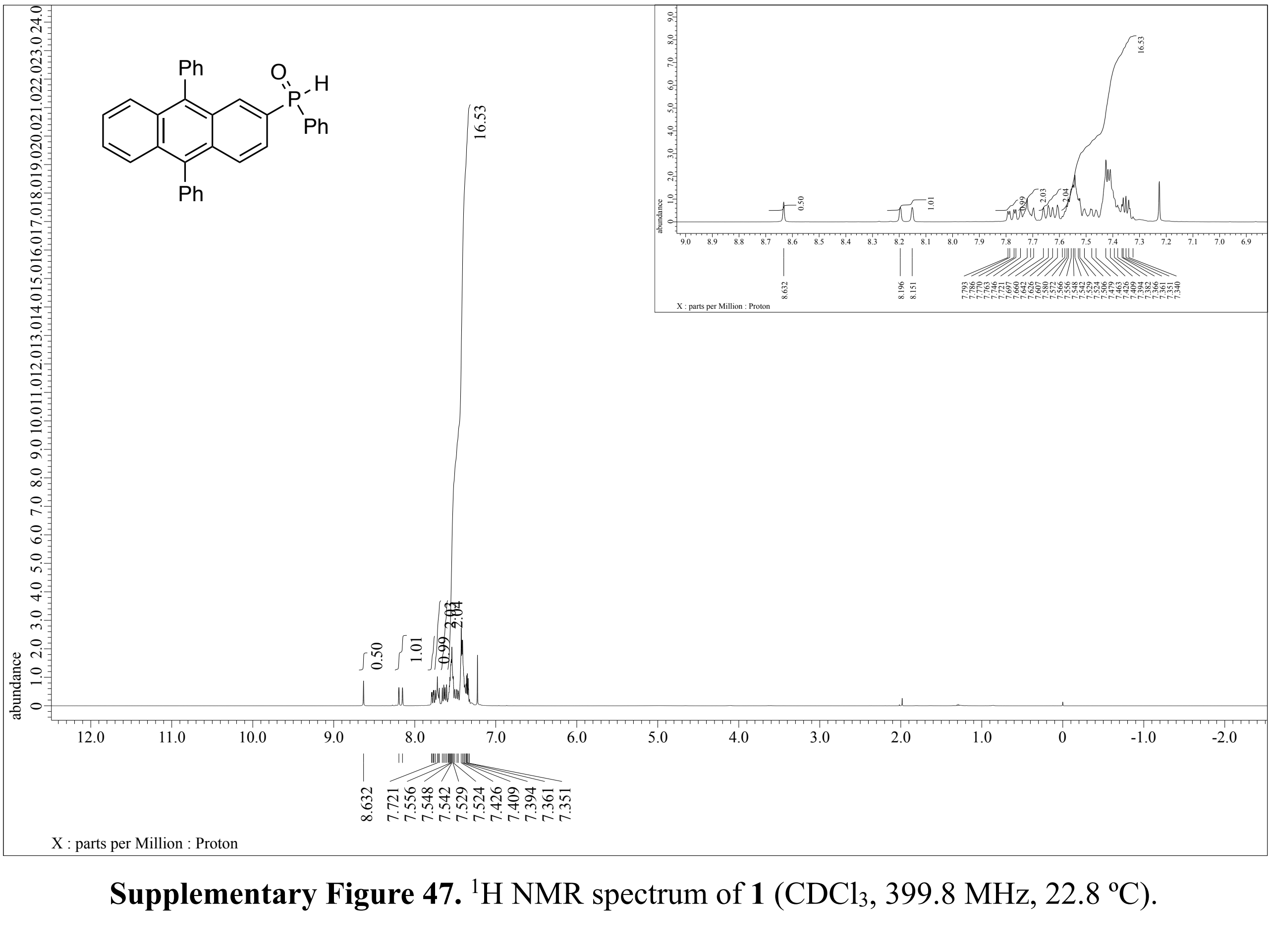

Supplementary Figure 47. ^{1}H NMR spectrum of **1** ($CDCl_3$, 399.8 MHz, 22.8 ºC).

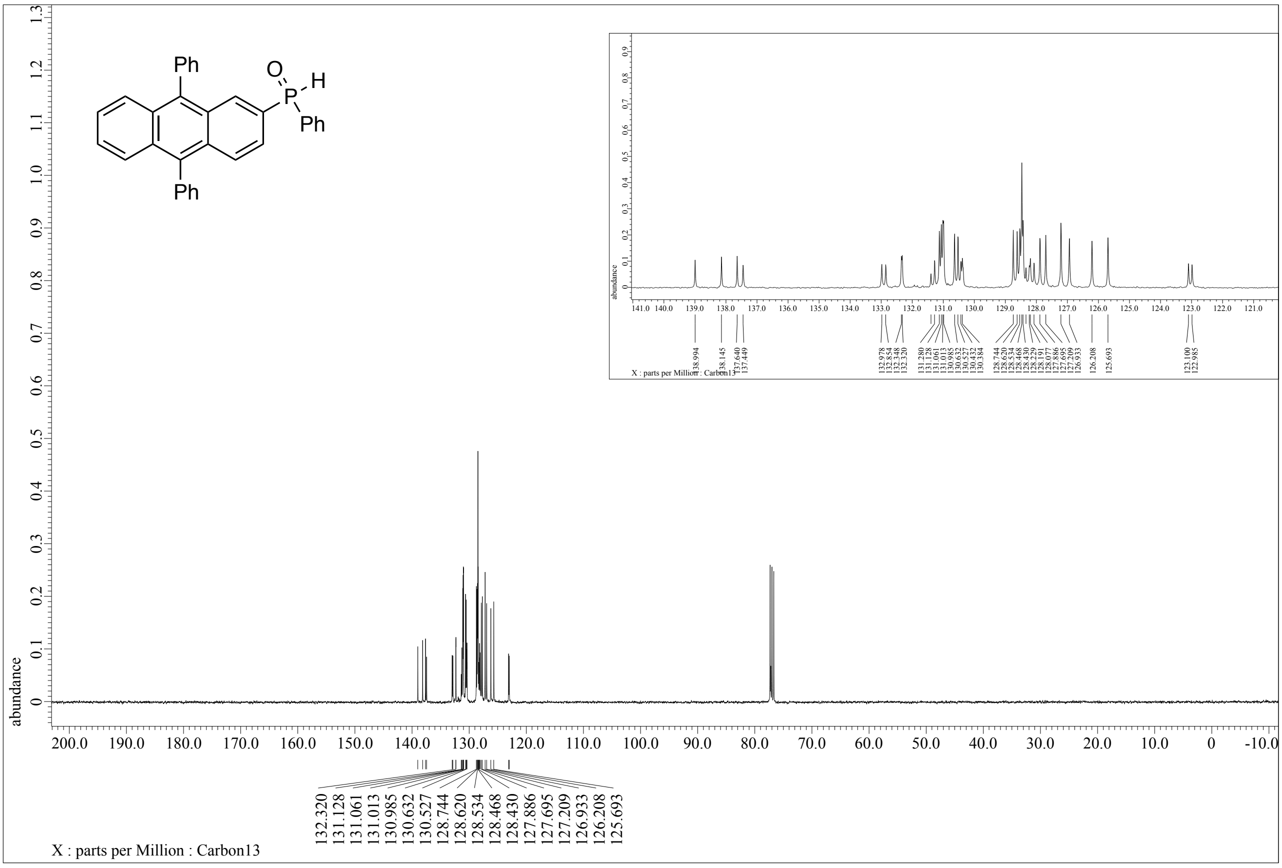

Supplementary Figure 48. ^{13}C NMR spectrum of **1** ($CDCl_3$, 100.5 MHz, 22.5 ºC).

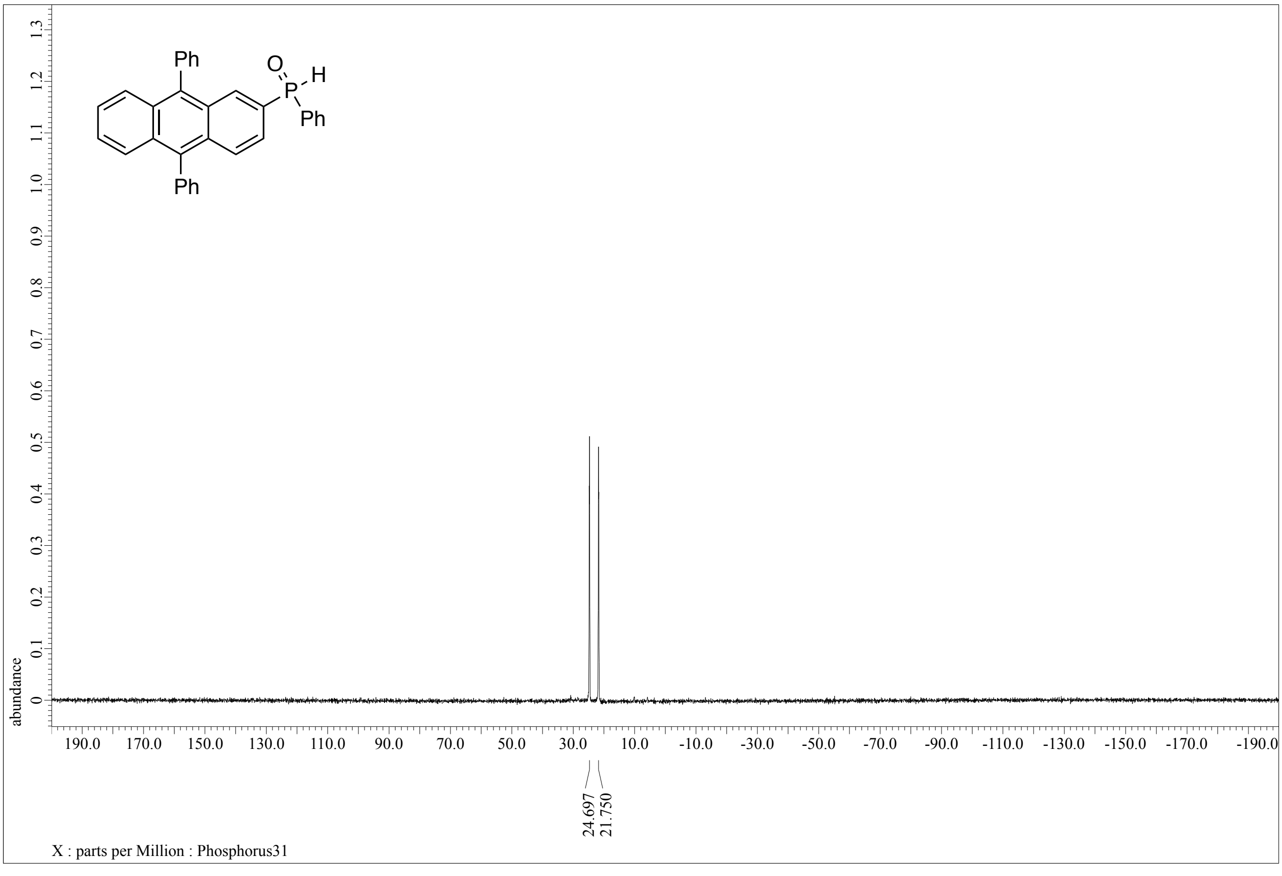

Supplementary Figure 49. ^{31}P NMR spectrum of **1** ($CDCl_3$, 161.8 MHz, 20.5 ºC).

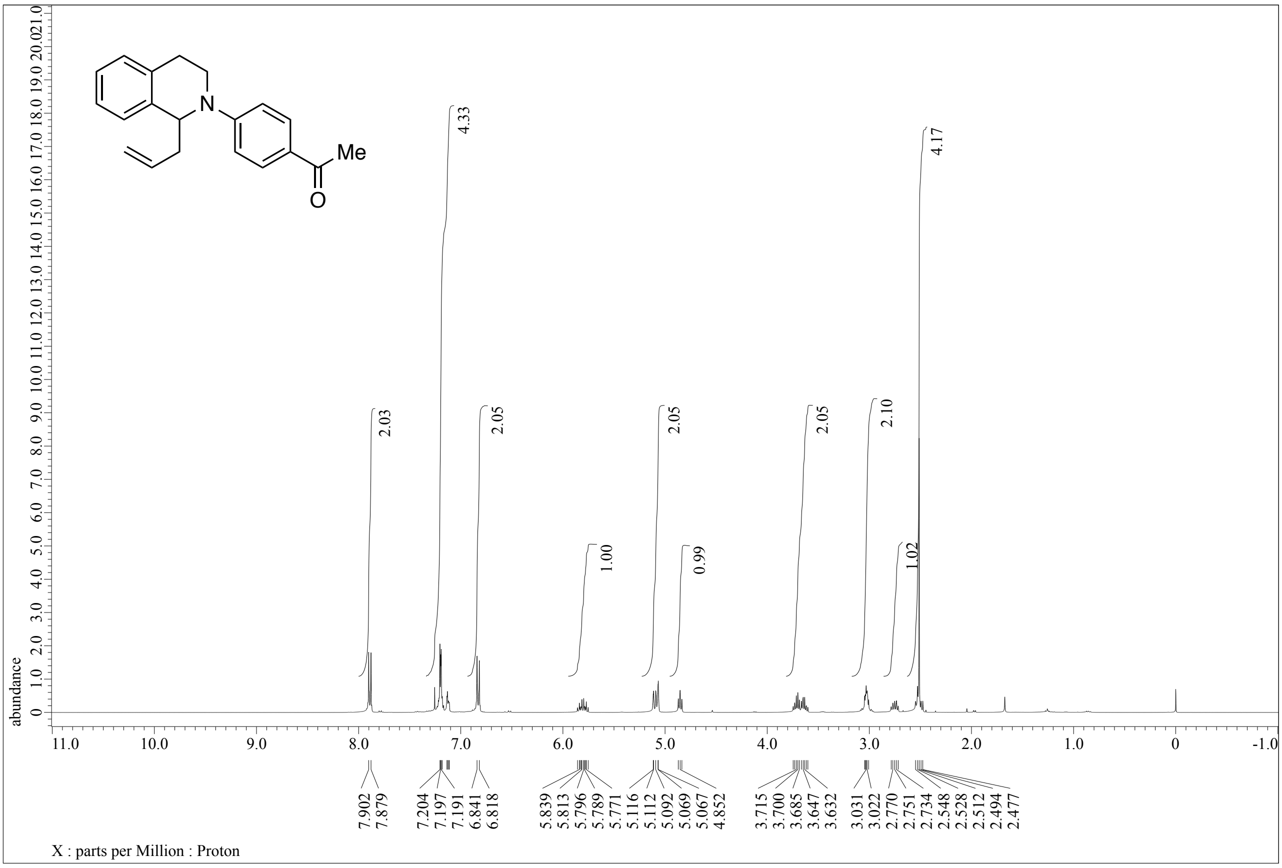

Supplementary Figure 50. ^{1}H NMR spectrum of **9b** ($CDCl_3$, 399.8 MHz, 19.6 ºC).

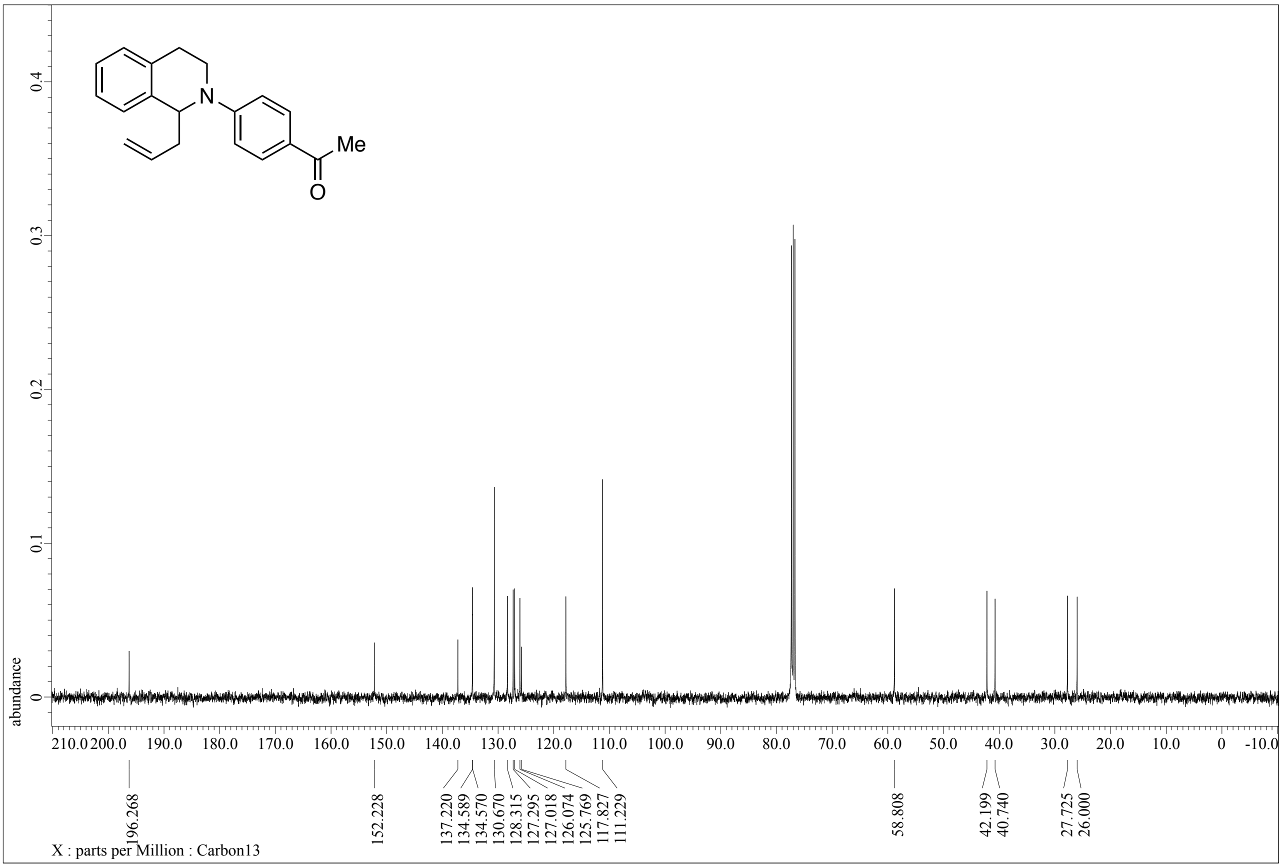

Supplementary Figure 51. ^{13}C NMR spectrum of **9b** ($CDCl_3$, 100.5 MHz, 19.8 ºC).

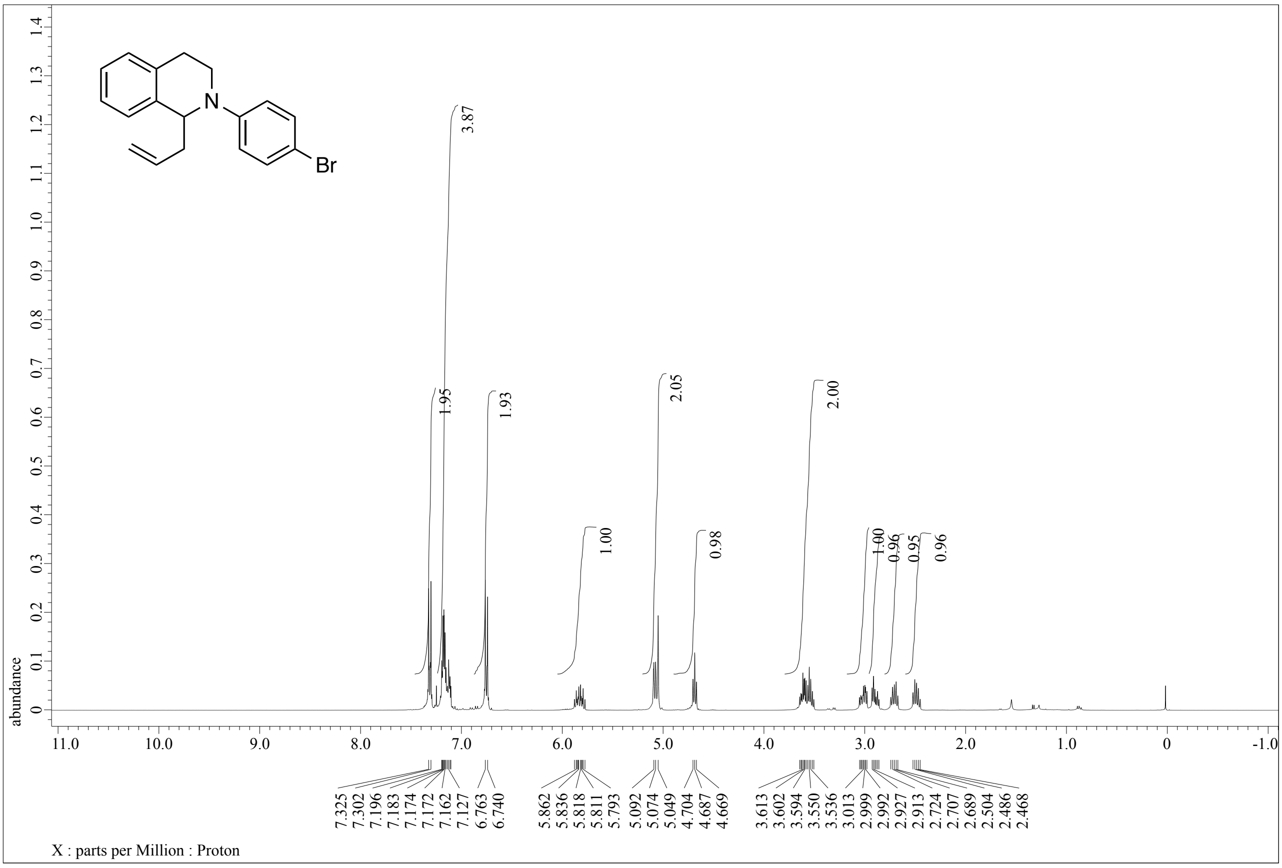

Supplementary Figure 52. ^{1}H NMR spectrum of **9e** ($CDCl_3$, 399.8 MHz, 21.4 ºC).

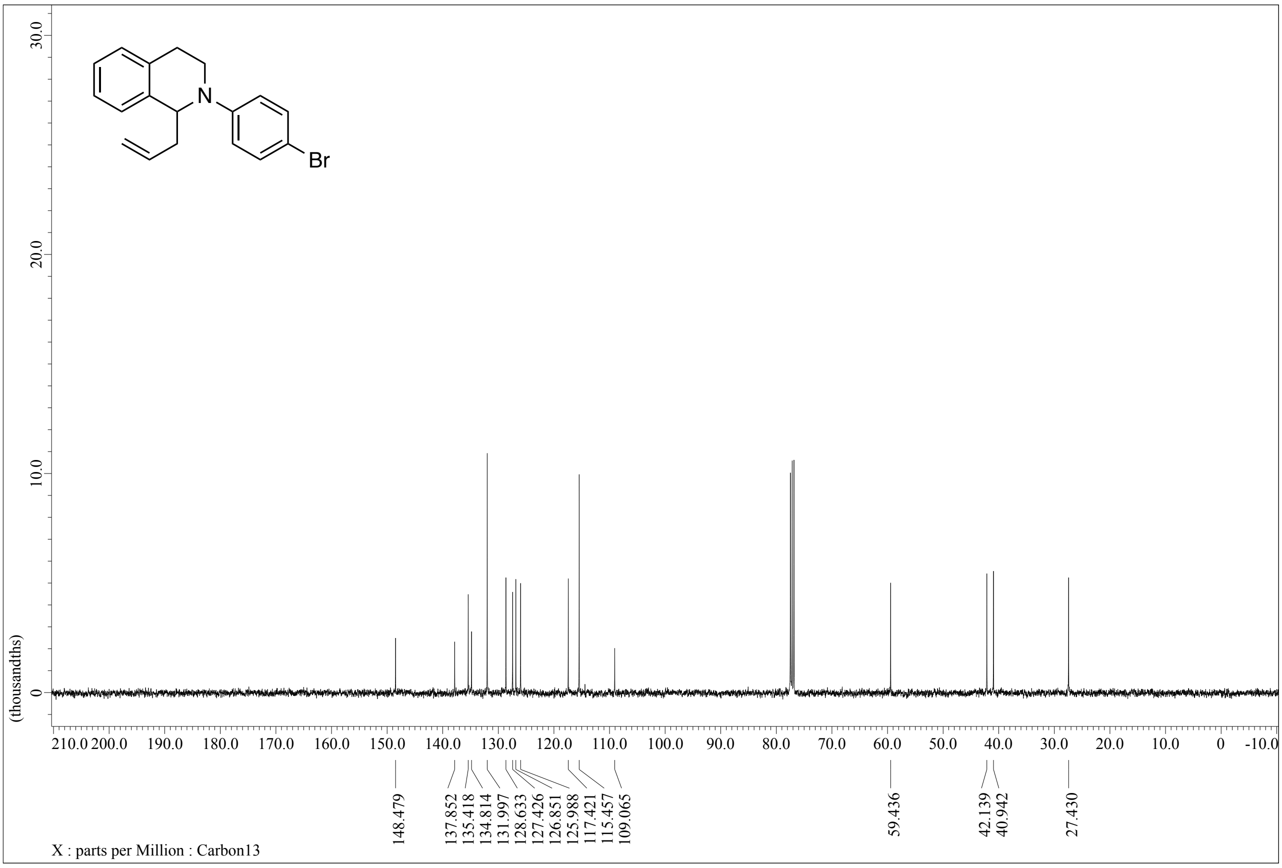

Supplementary Figure 53. ^{13}C NMR spectrum of **9e** ($CDCl_3$, 100.5 MHz, 21.5 ºC).

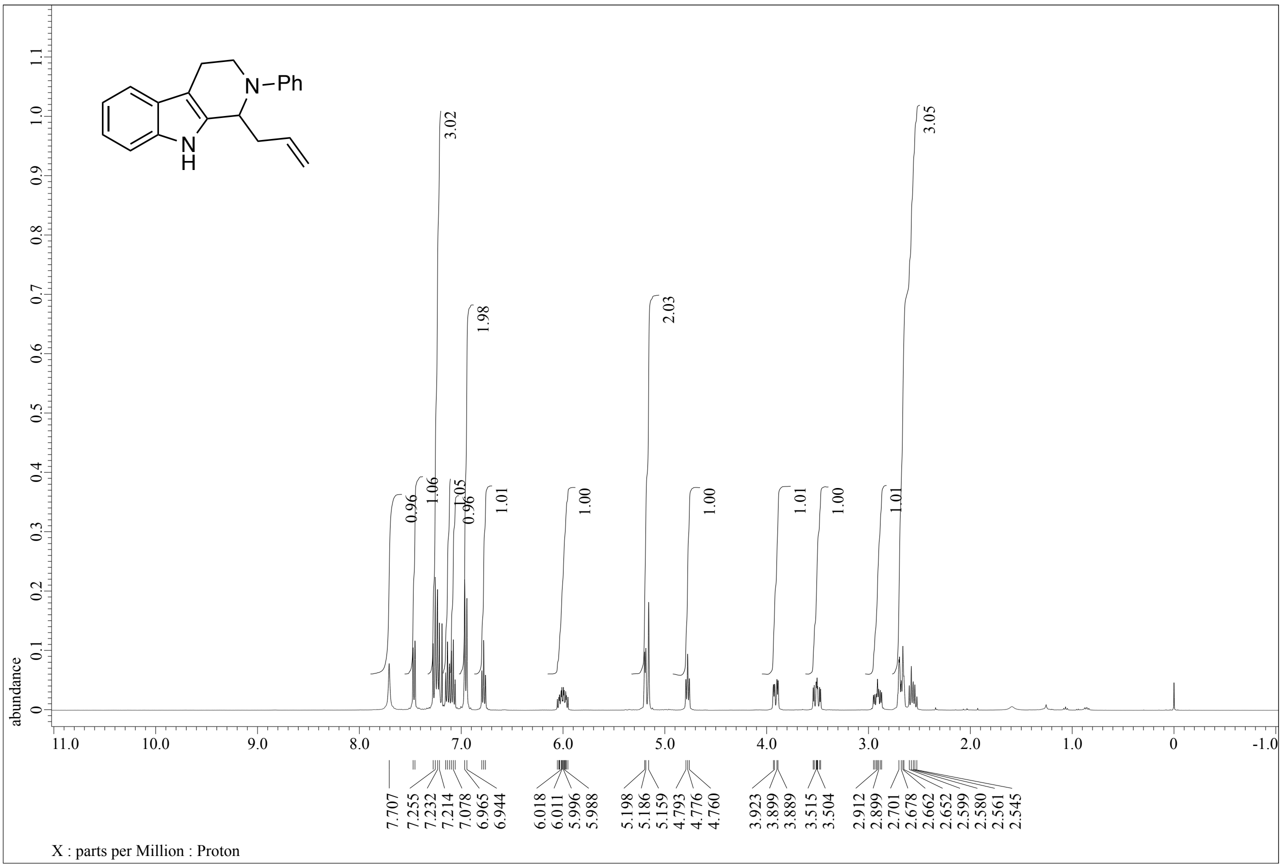

Supplementary Figure 54. ^{1}H NMR spectrum of **9i** ($CDCl_3$, 399.8 MHz, 24.3 ºC).

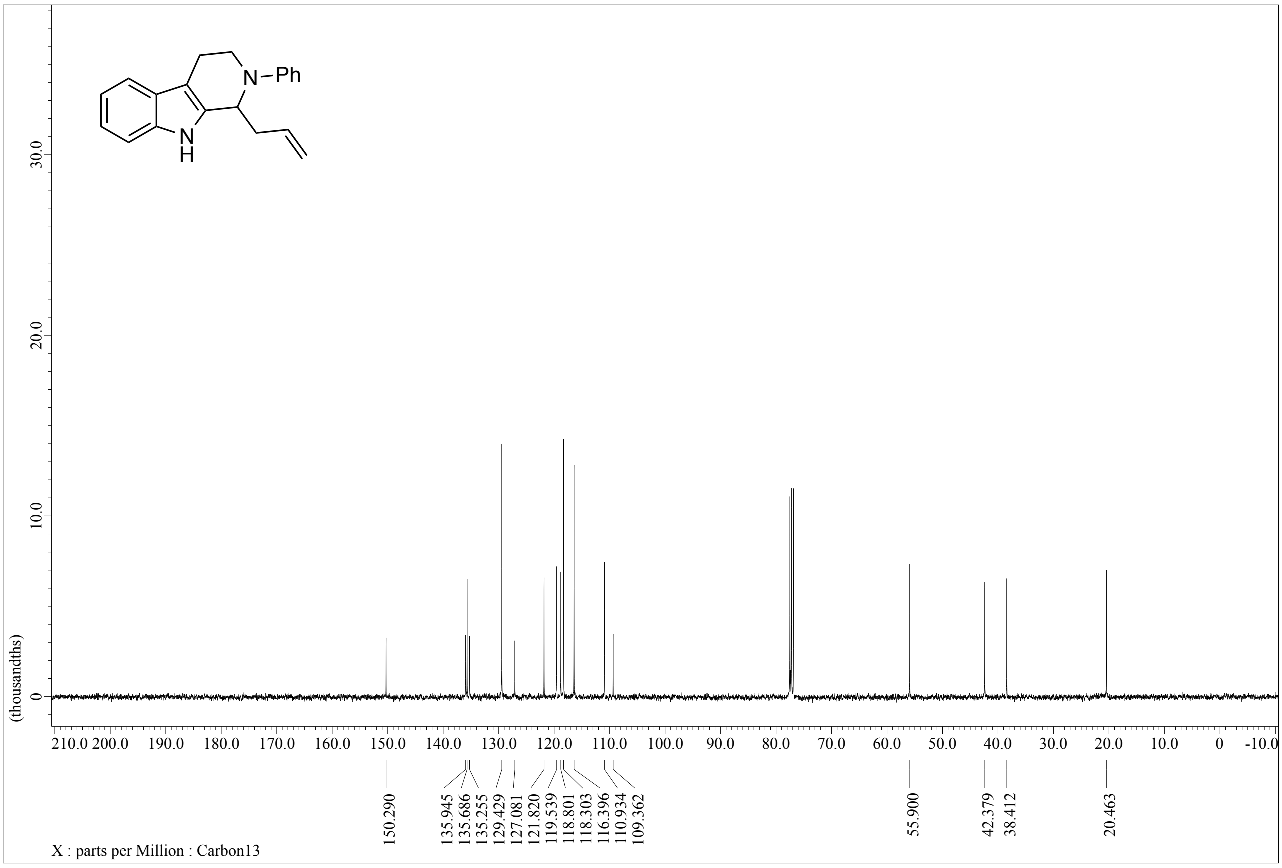

Supplementary Figure 55. ^{13}C NMR spectrum of **9i** ($CDCl_3$, 100.5 MHz, 24.3 ºC).

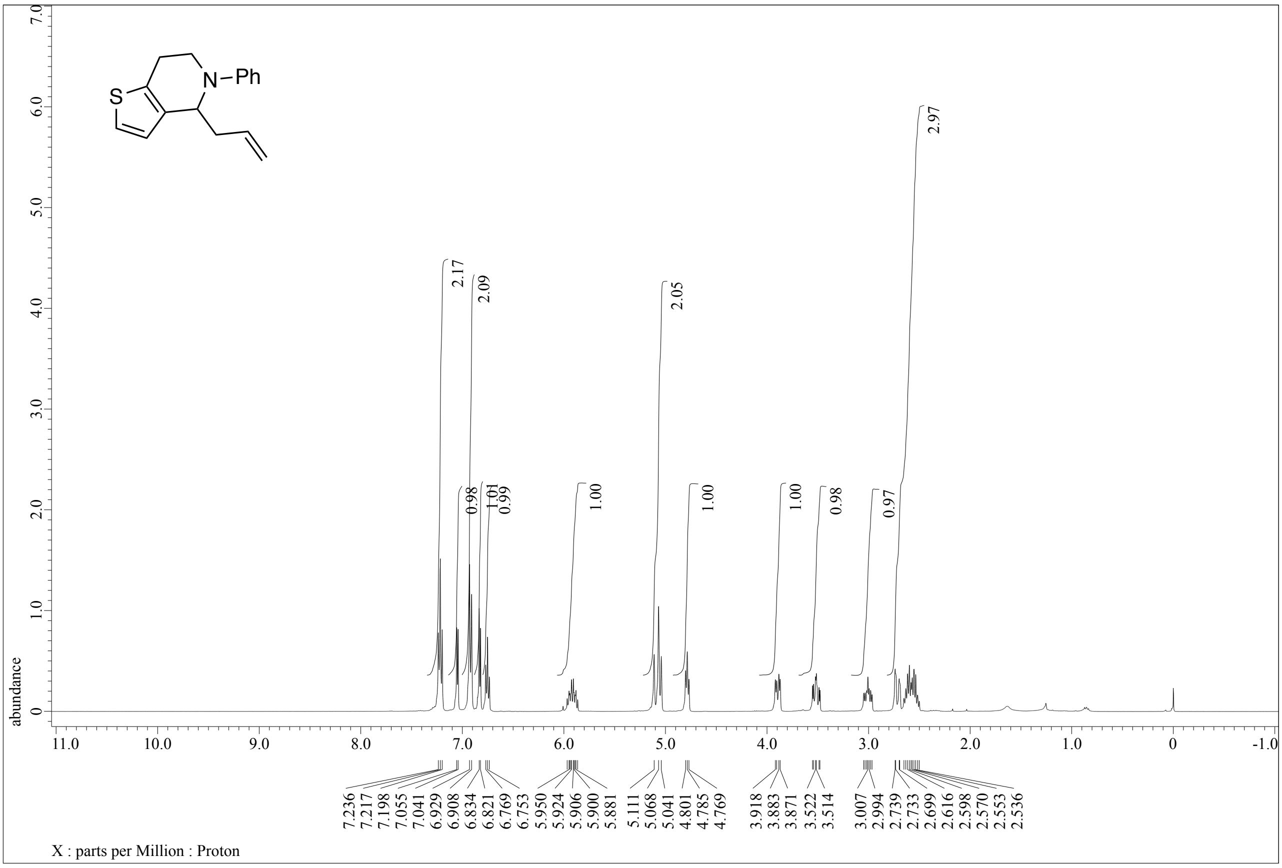

Supplementary Figure 56. ^{1}H NMR spectrum of **9j** ($CDCl_3$, 399.8 MHz, 24.0 ºC).

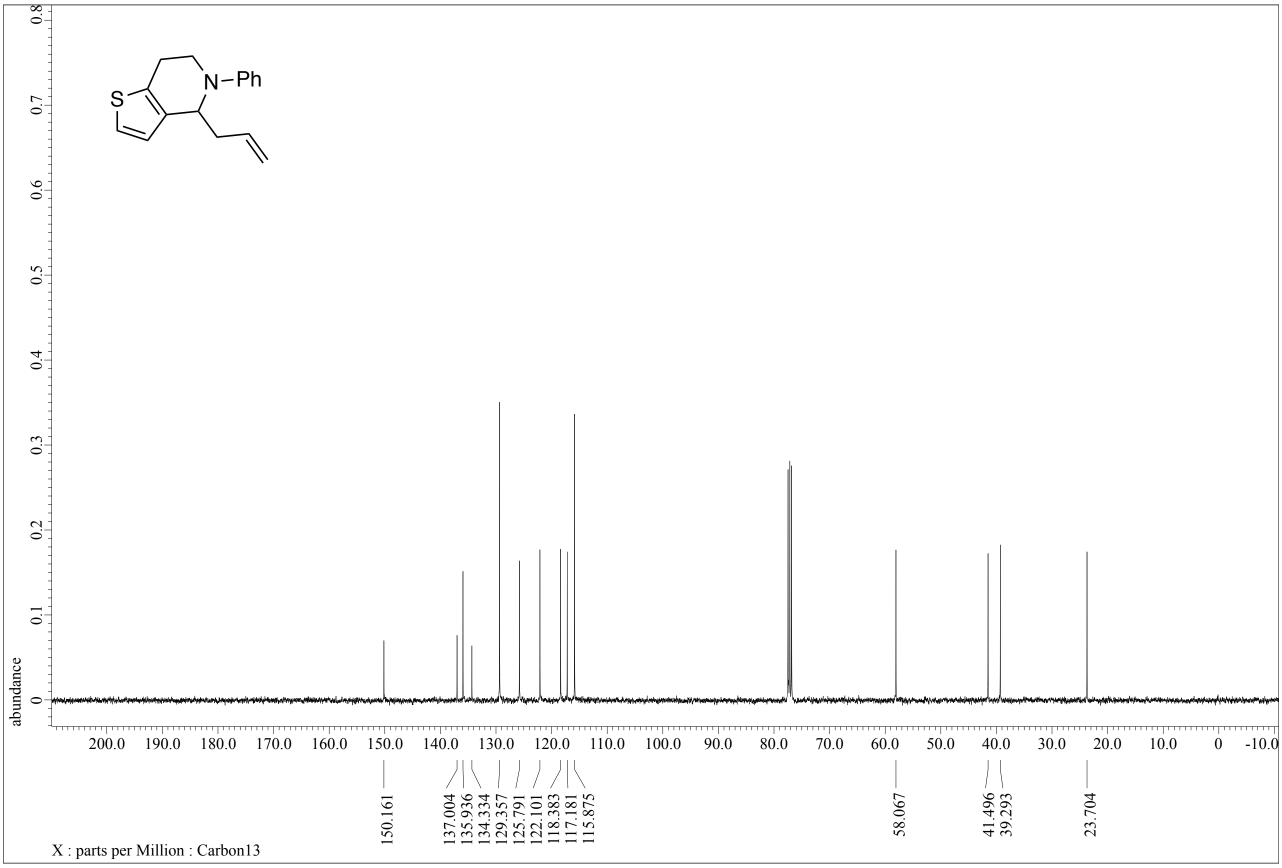

Supplementary Figure 57. ^{13}C NMR spectrum of **9j** ($CDCl_3$, 100.5 MHz, 24.0 ºC).

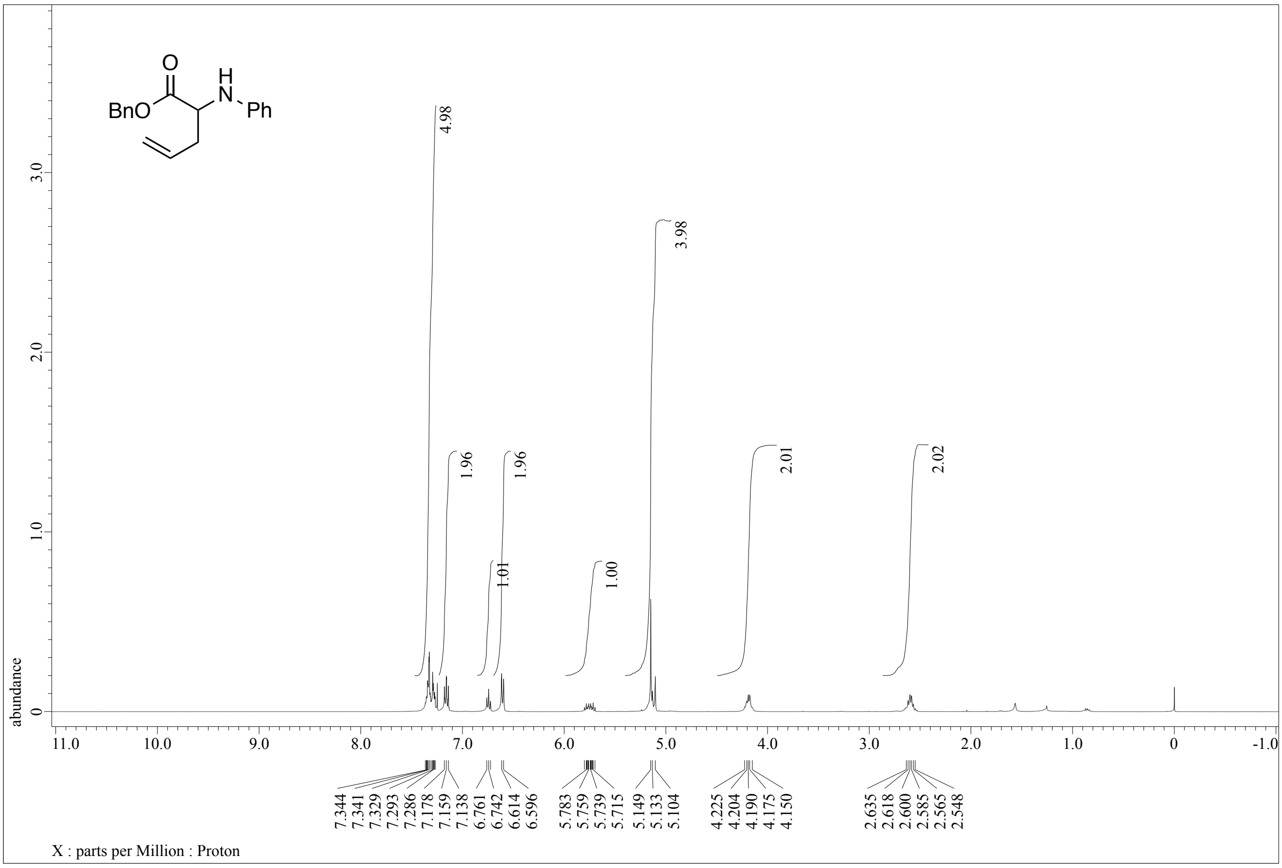

Supplementary Figure 58. ^{1}H NMR spectrum of **9l** ($CDCl_3$, 399.8 MHz, 22.1 ºC).

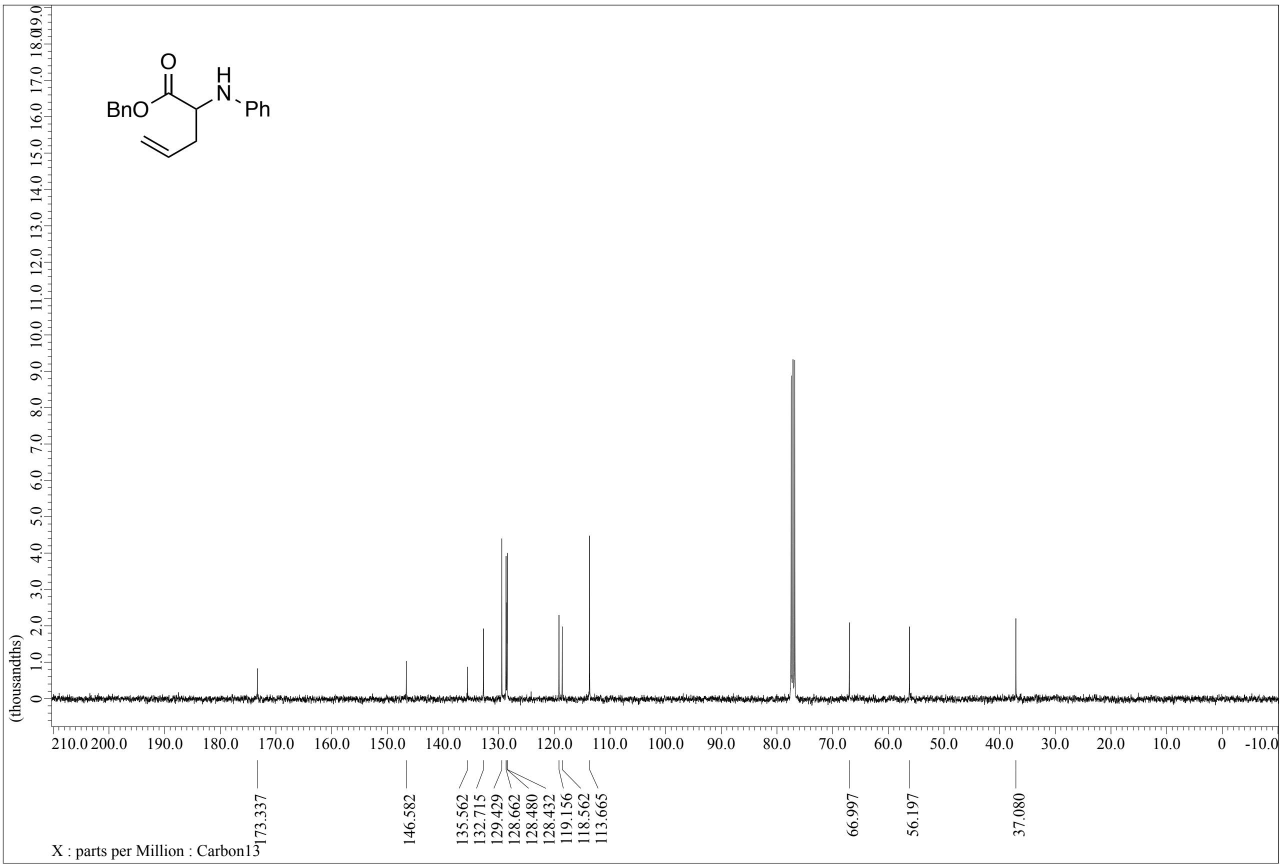

Supplementary Figure 59. ^{13}C NMR spectrum of **9l** ($CDCl_3$, 100.5 MHz, 22.4 ºC).

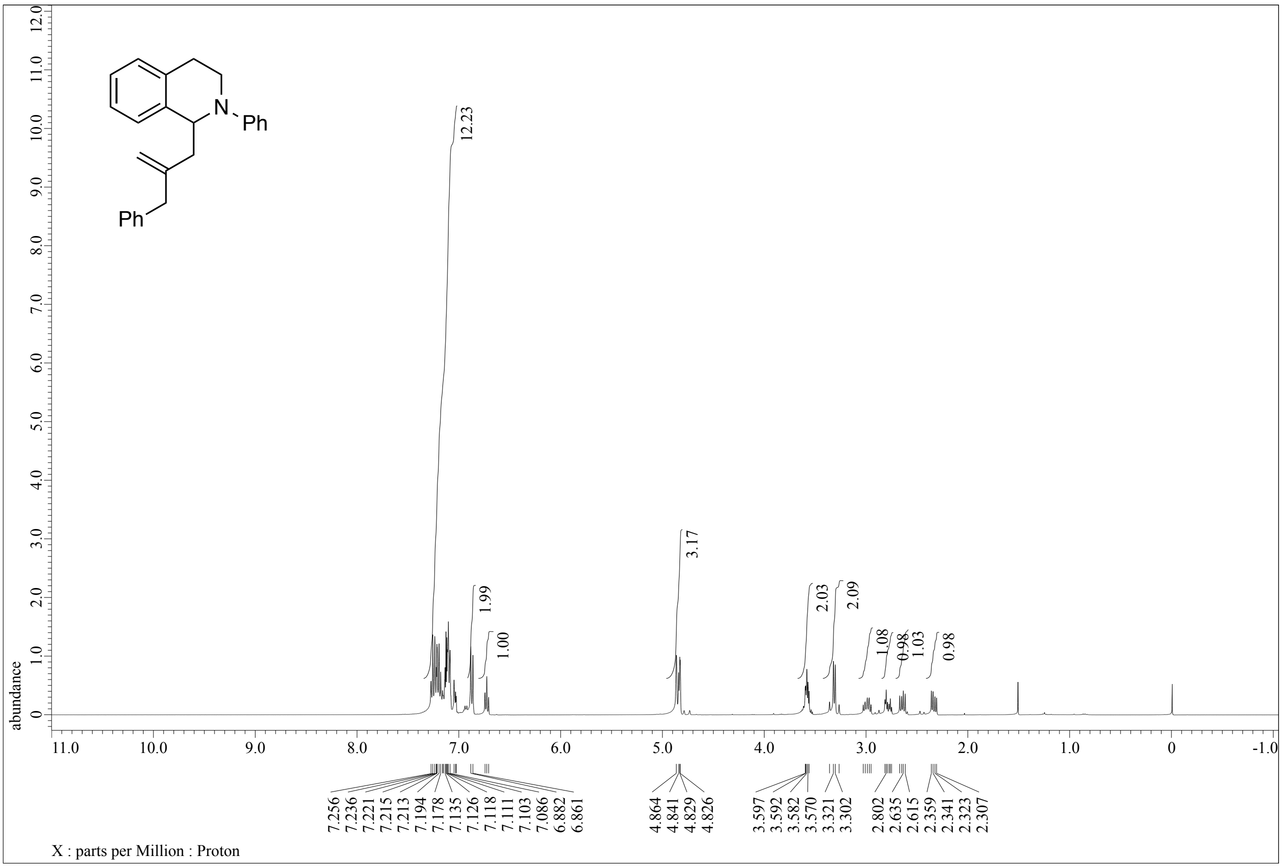

Supplementary Figure 60. ^{1}H NMR spectrum of **9n** ($CDCl_3$, 399.8 MHz, 20.3 ºC).

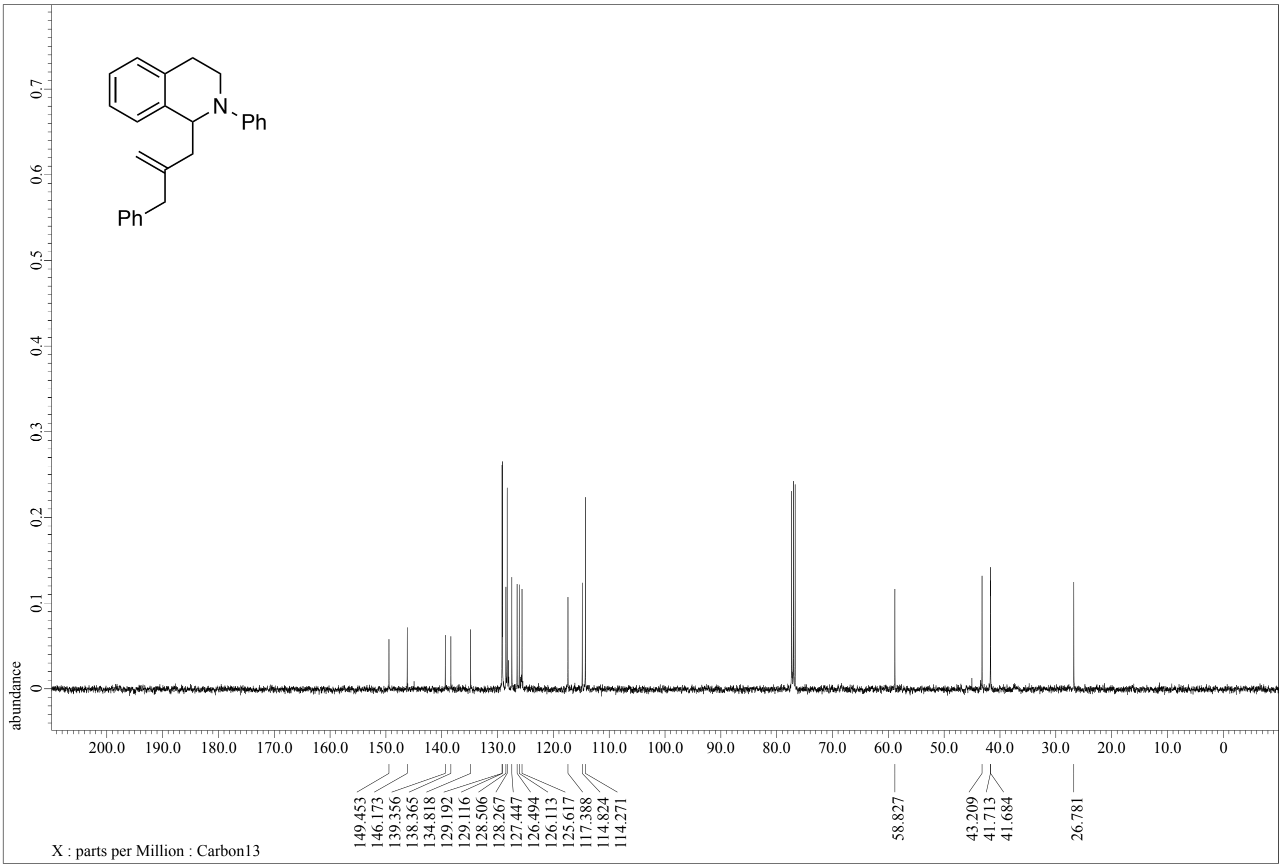

Supplementary Figure 61. ^{13}C NMR spectrum of **9n** ($CDCl_3$, 100.5 MHz, 20.1 ºC).

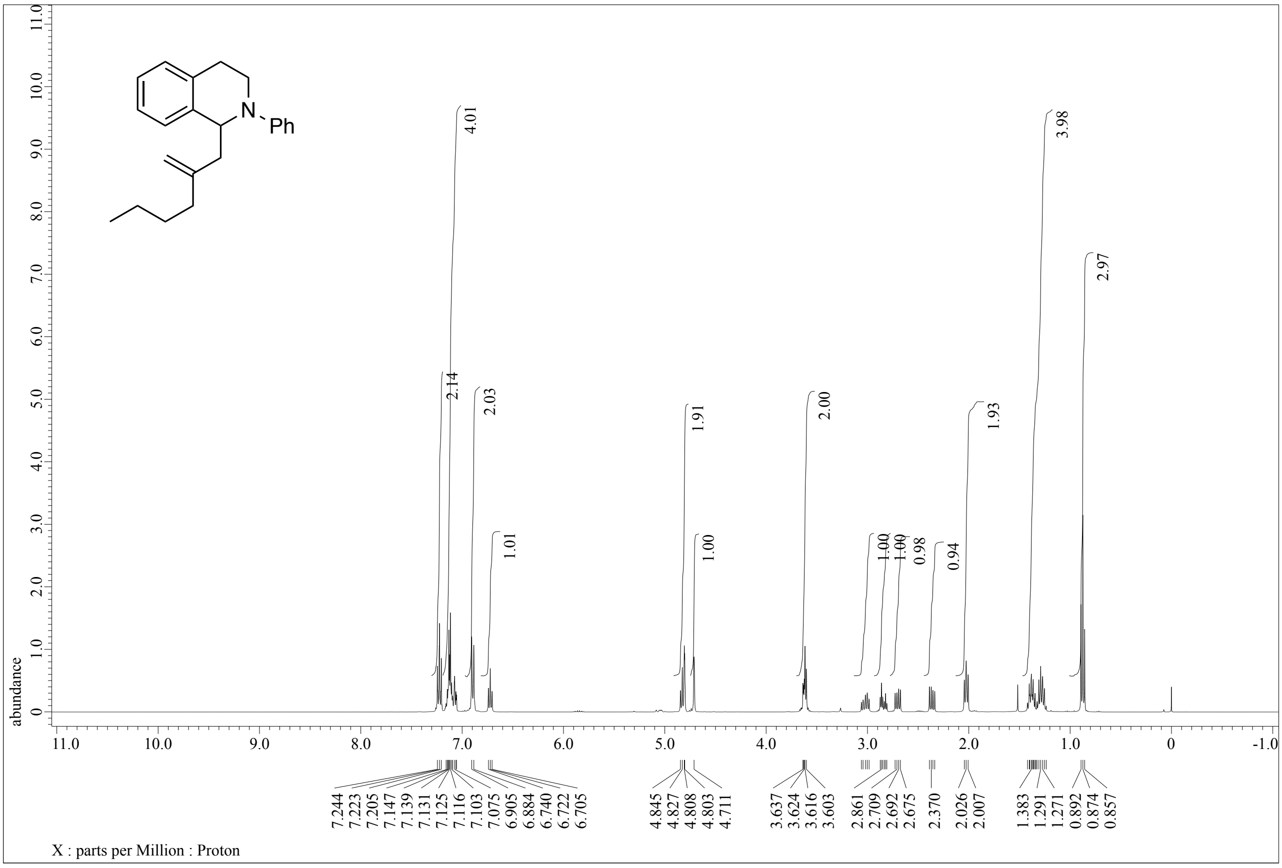

Supplementary Figure 62. ^{1}H NMR spectrum of **9o** ($CDCl_3$, 399.8 MHz, 19.8 ºC).

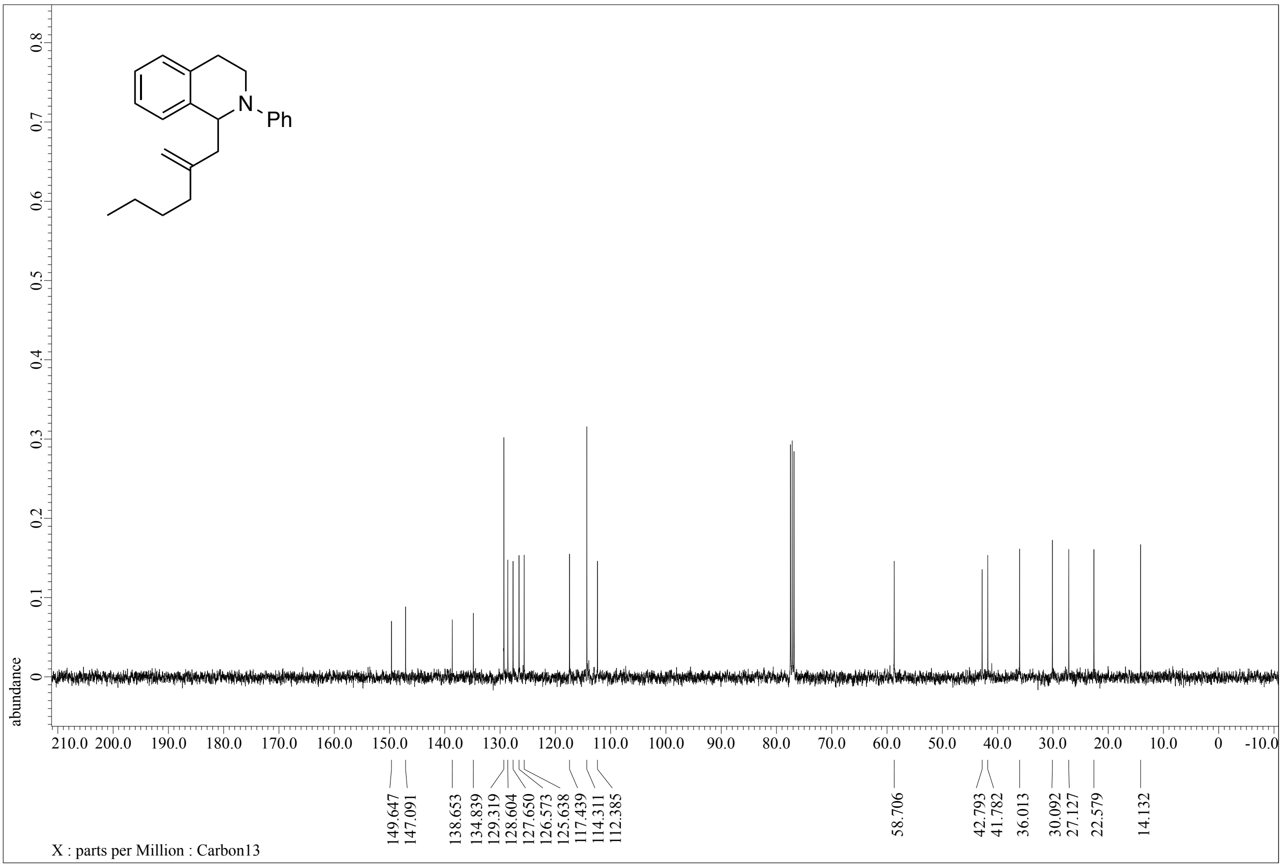

Supplementary Figure 63. ^{13}C NMR spectrum of **9o** ($CDCl_3$, 100.5 MHz, 19.9 ºC).

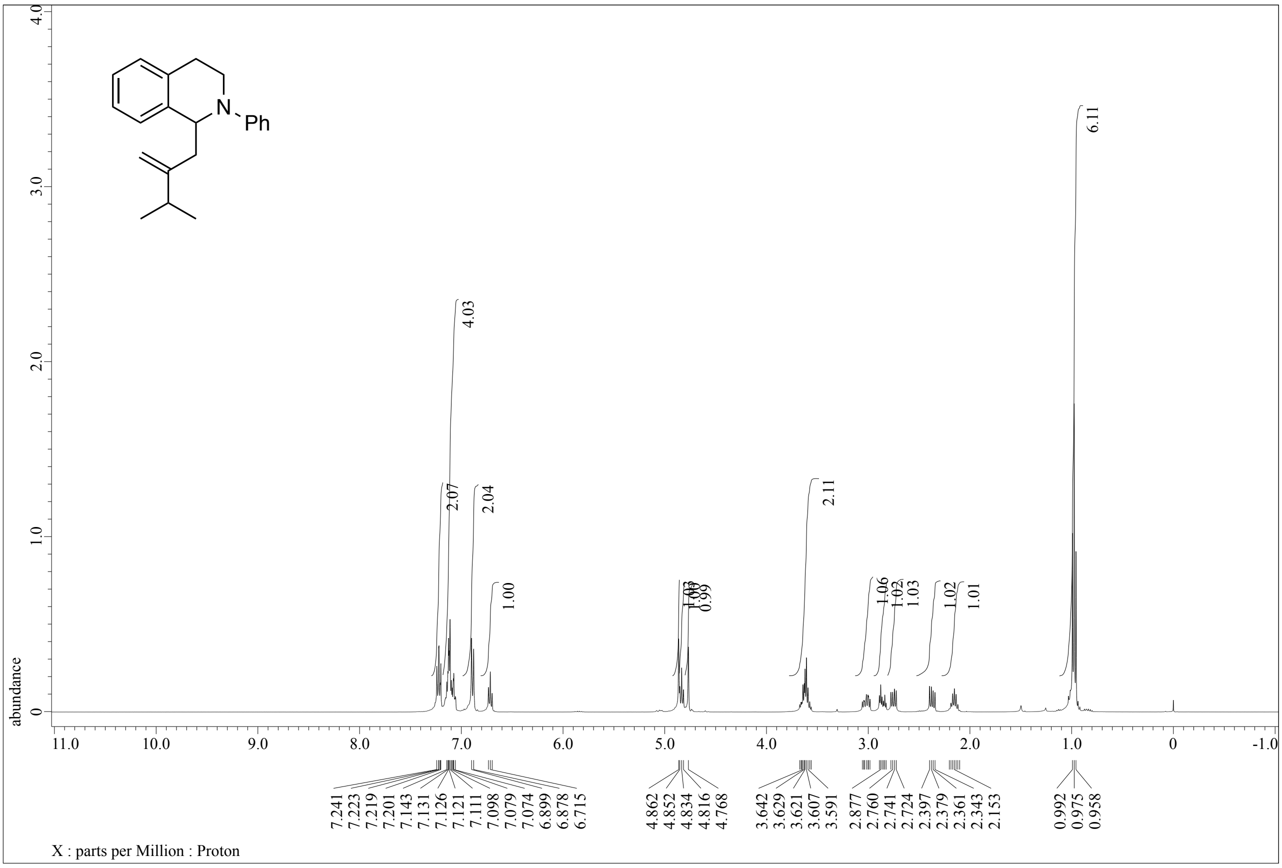

Supplementary Figure 64. ^{1}H NMR spectrum of **9p** ($CDCl_3$, 399.8 MHz, 20.9 ºC).

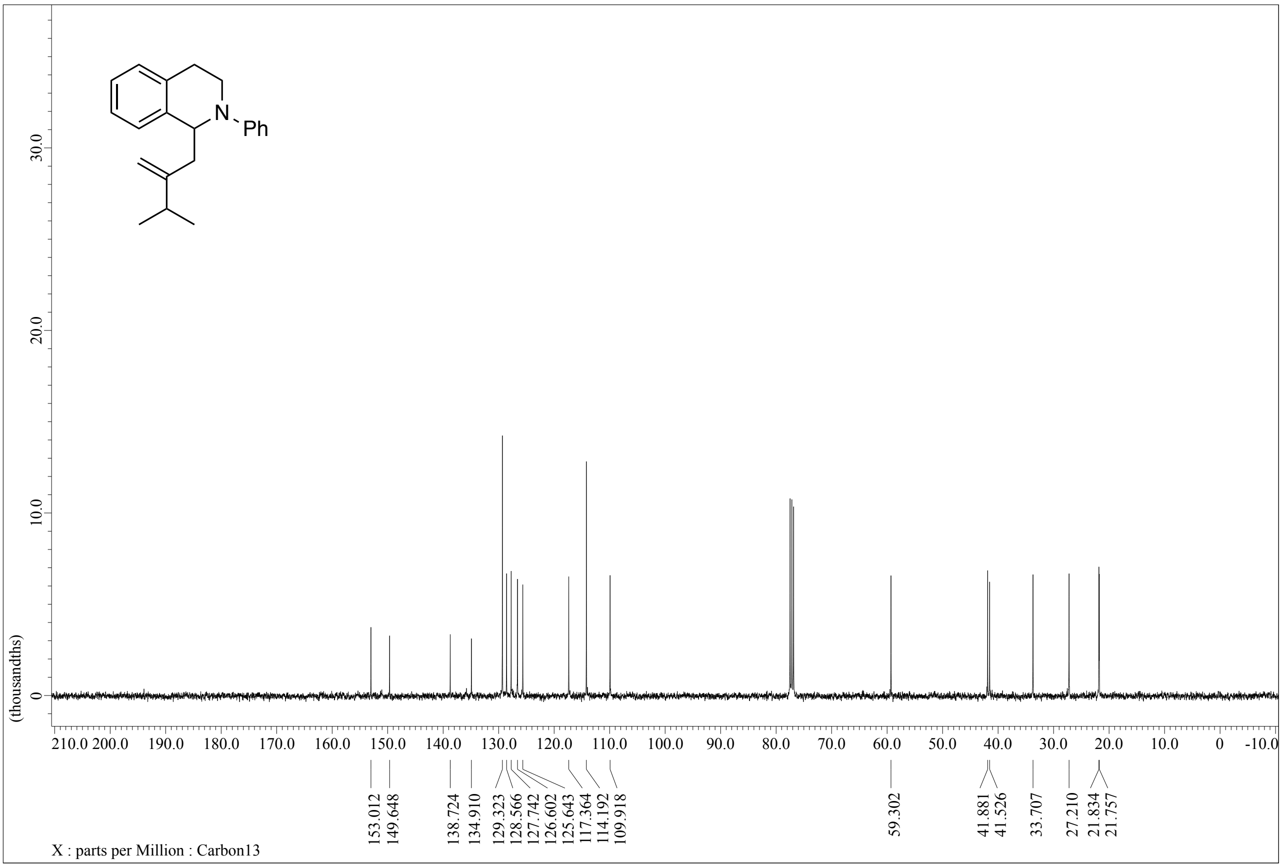

Supplementary Figure 65. ^{13}C NMR spectrum of **9p** ($CDCl_3$, 100.5 MHz, 21.0 ºC).

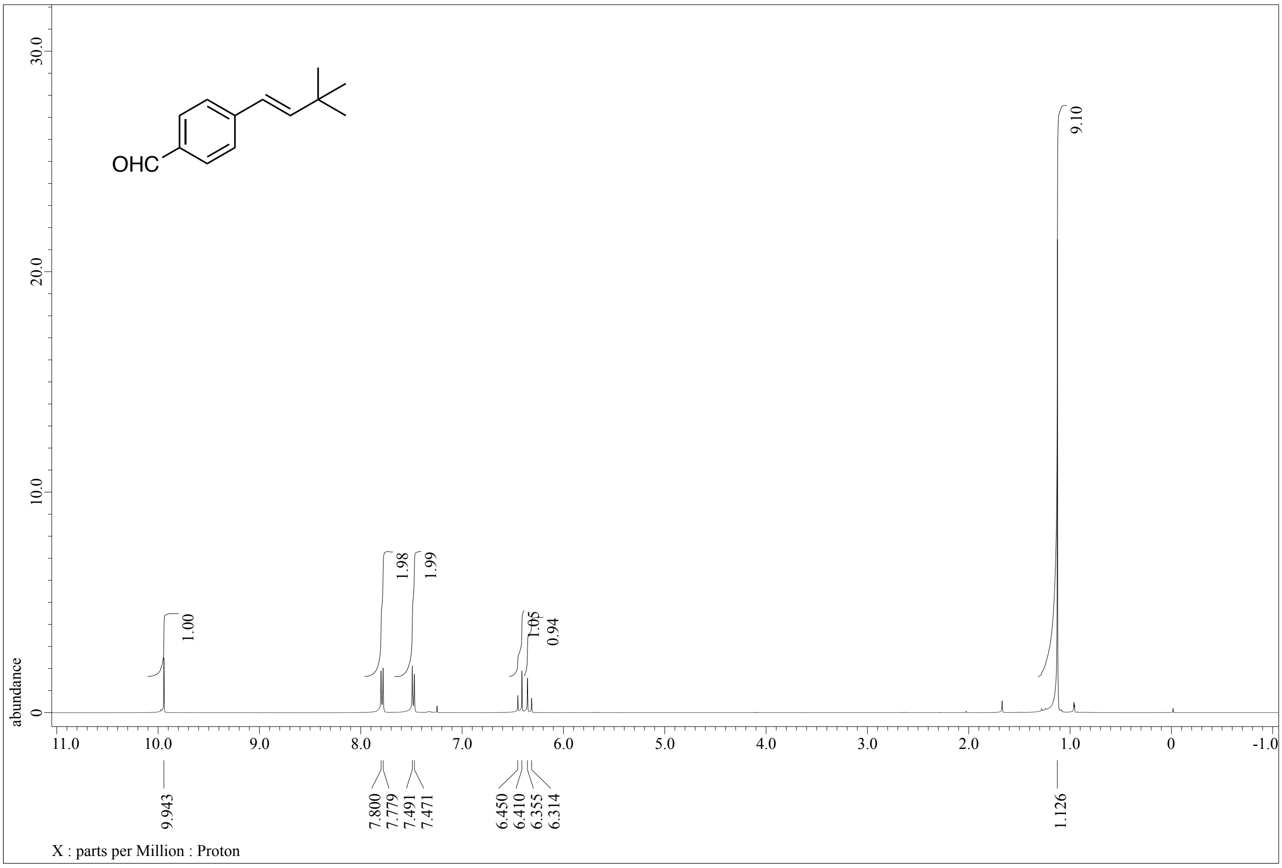

Supplementary Figure 66. ^{1}H NMR spectrum of **12e** ($CDCl_3$, 399.8 MHz, 23.2 ºC).

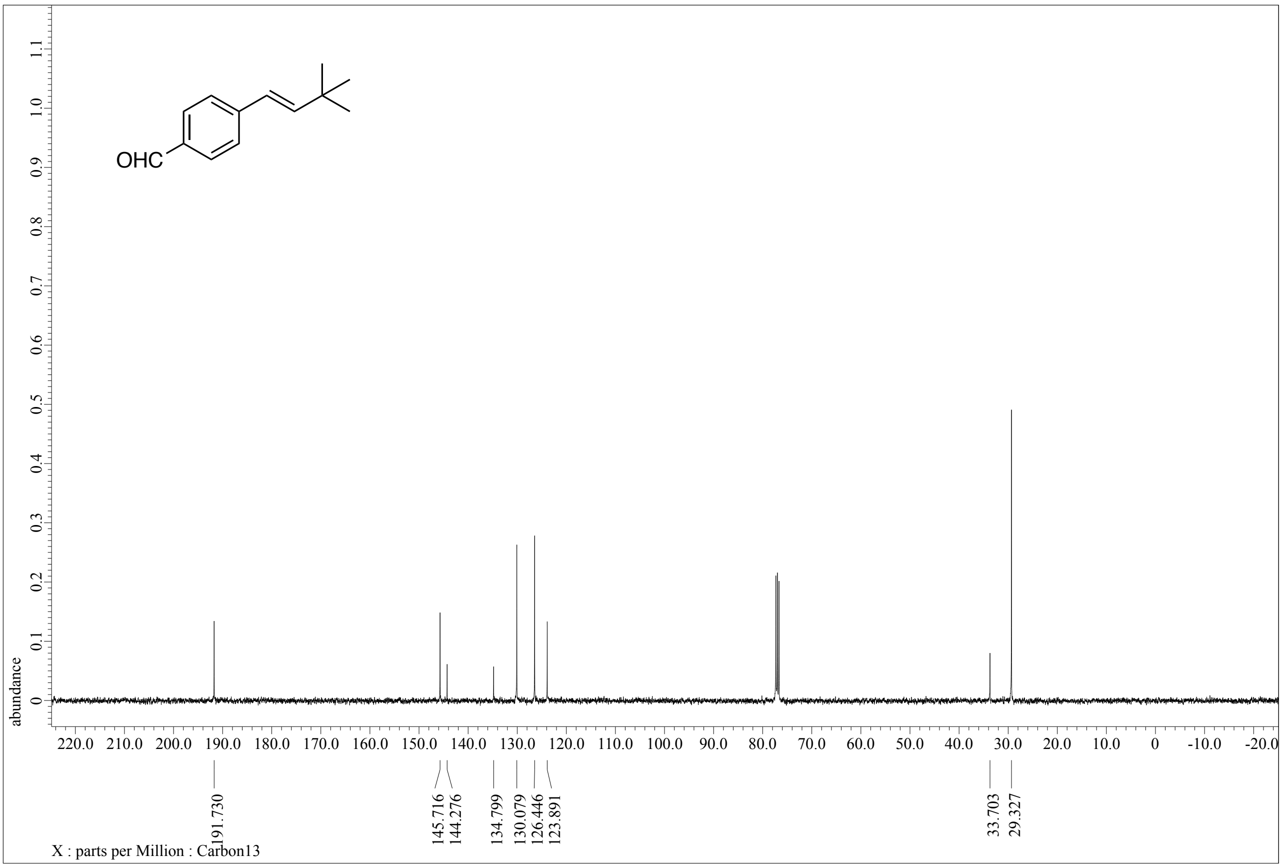

Supplementary Figure 67. ^{13}C NMR spectrum of **12e** ($CDCl_3$, 100.5 MHz, 23.3 ºC).

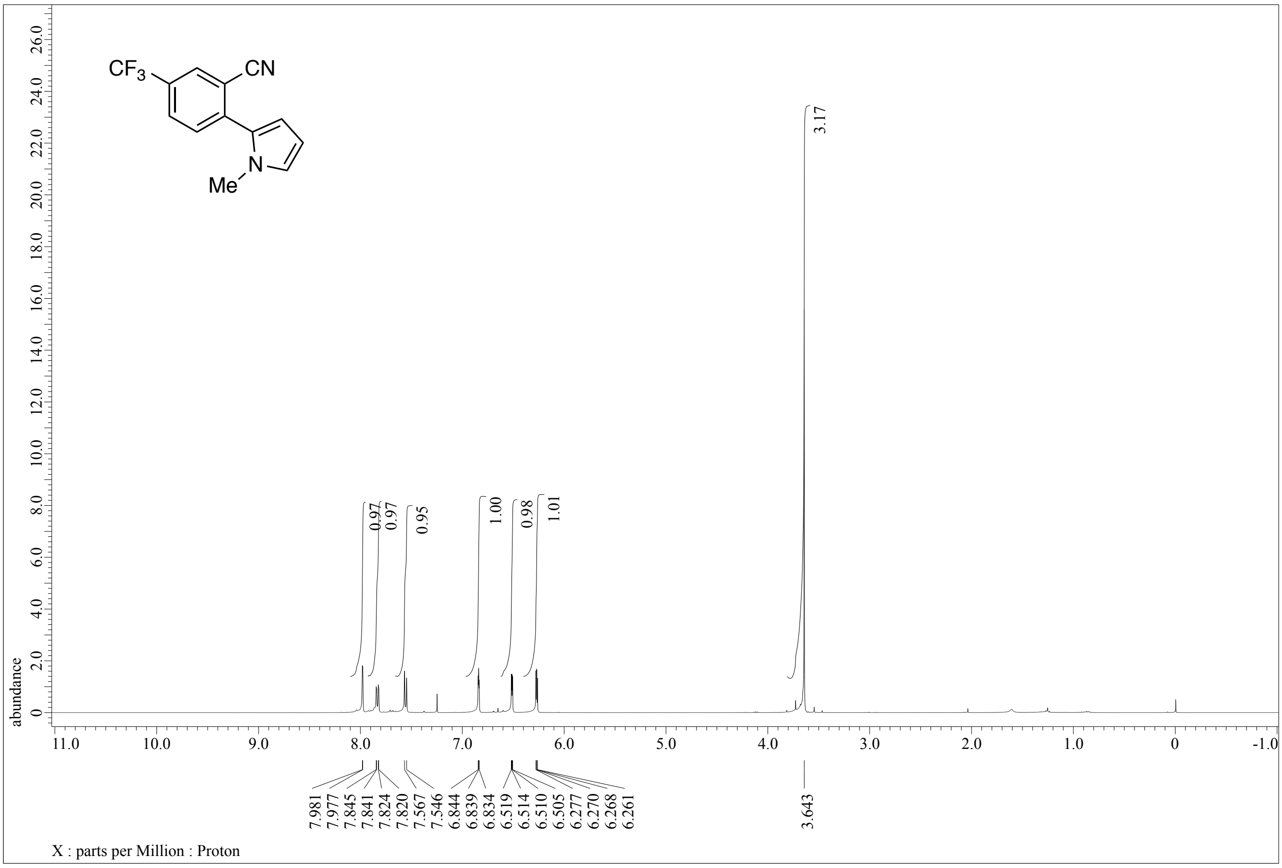

Supplementary Figure 68. ^{1}H NMR spectrum of **15c** ($CDCl_3$, 399.8 MHz, 23.9 ºC).

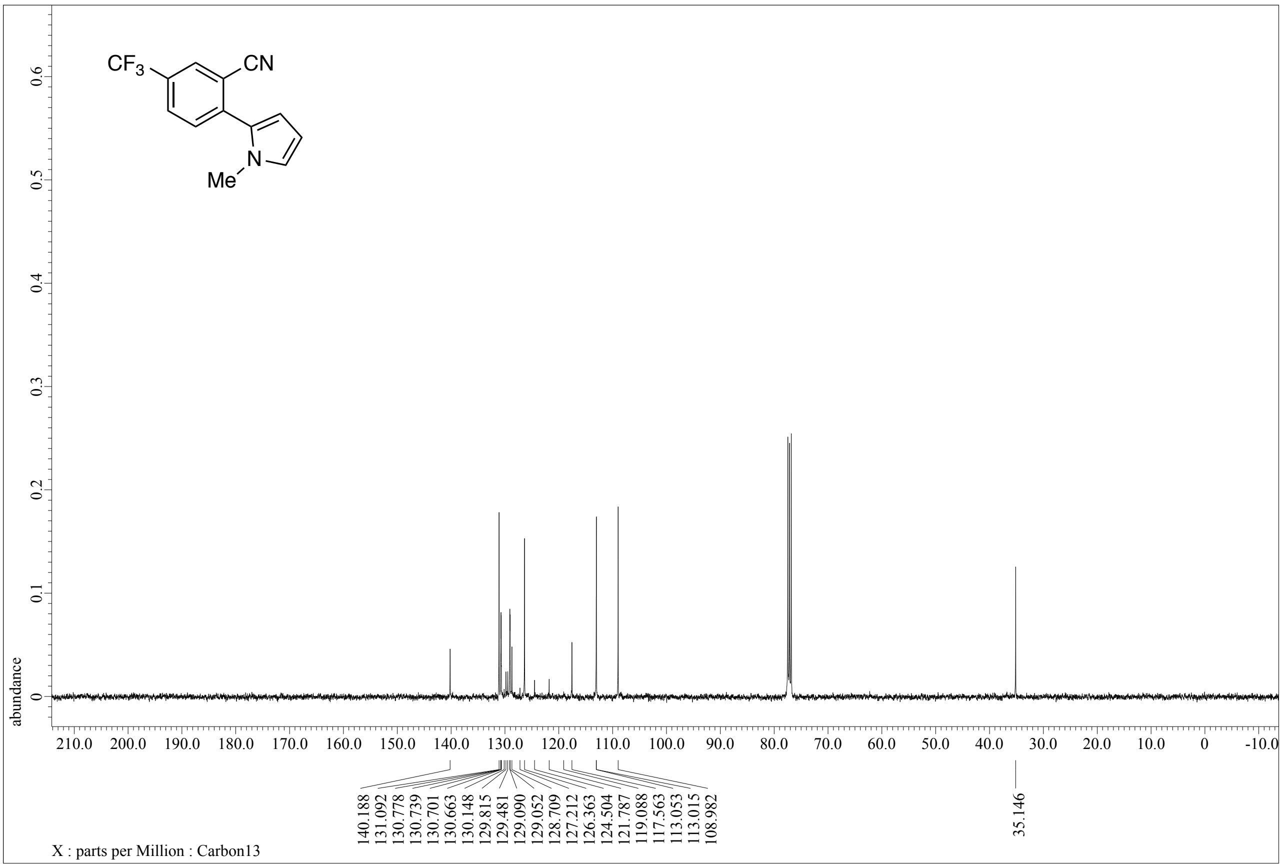

Supplementary Figure 69. ^{13}C NMR spectrum of **15c** ($CDCl_3$, 100.5 MHz, 23.7 ºC).

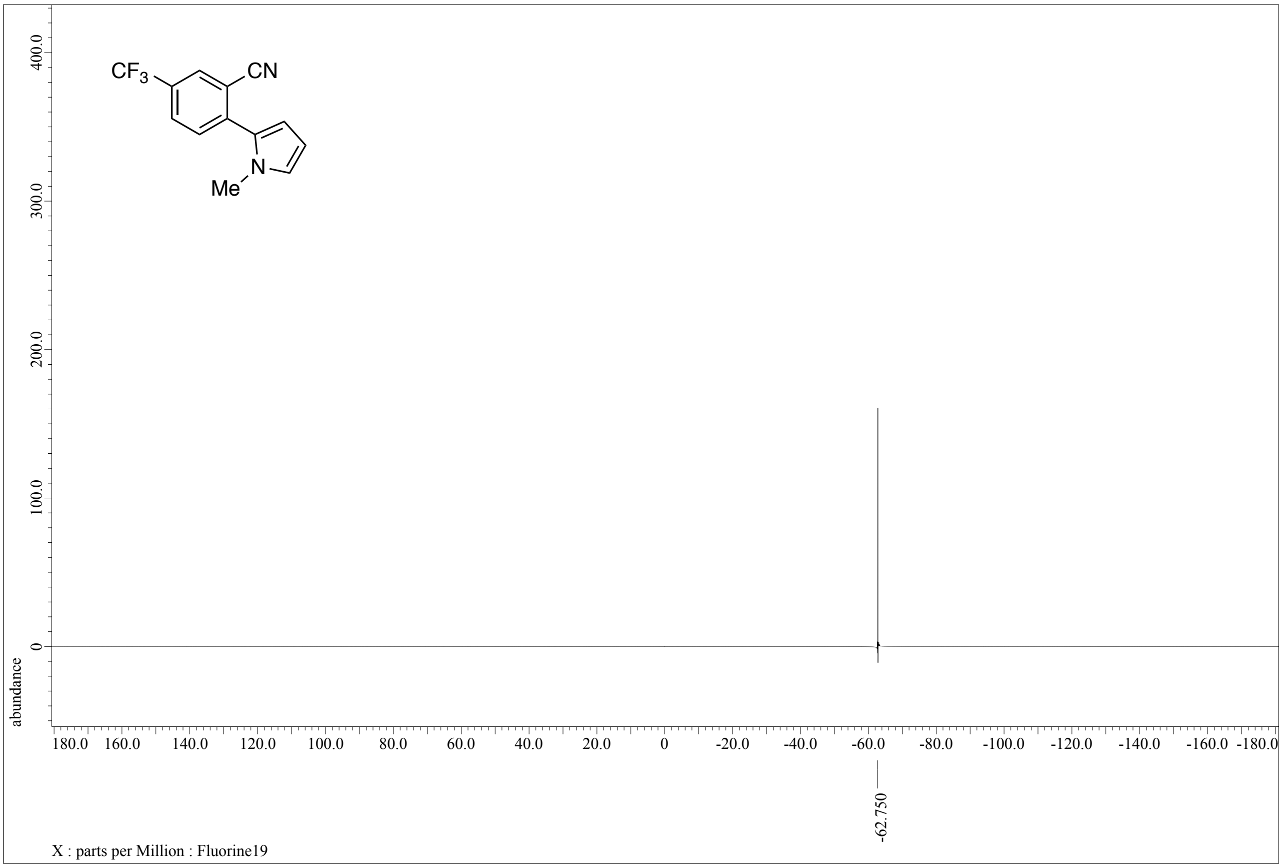

Supplementary Figure 70. ^{19}F NMR spectrum of **15c** ($CDCl_3$, 376.2 MHz, 23.8 ºC).

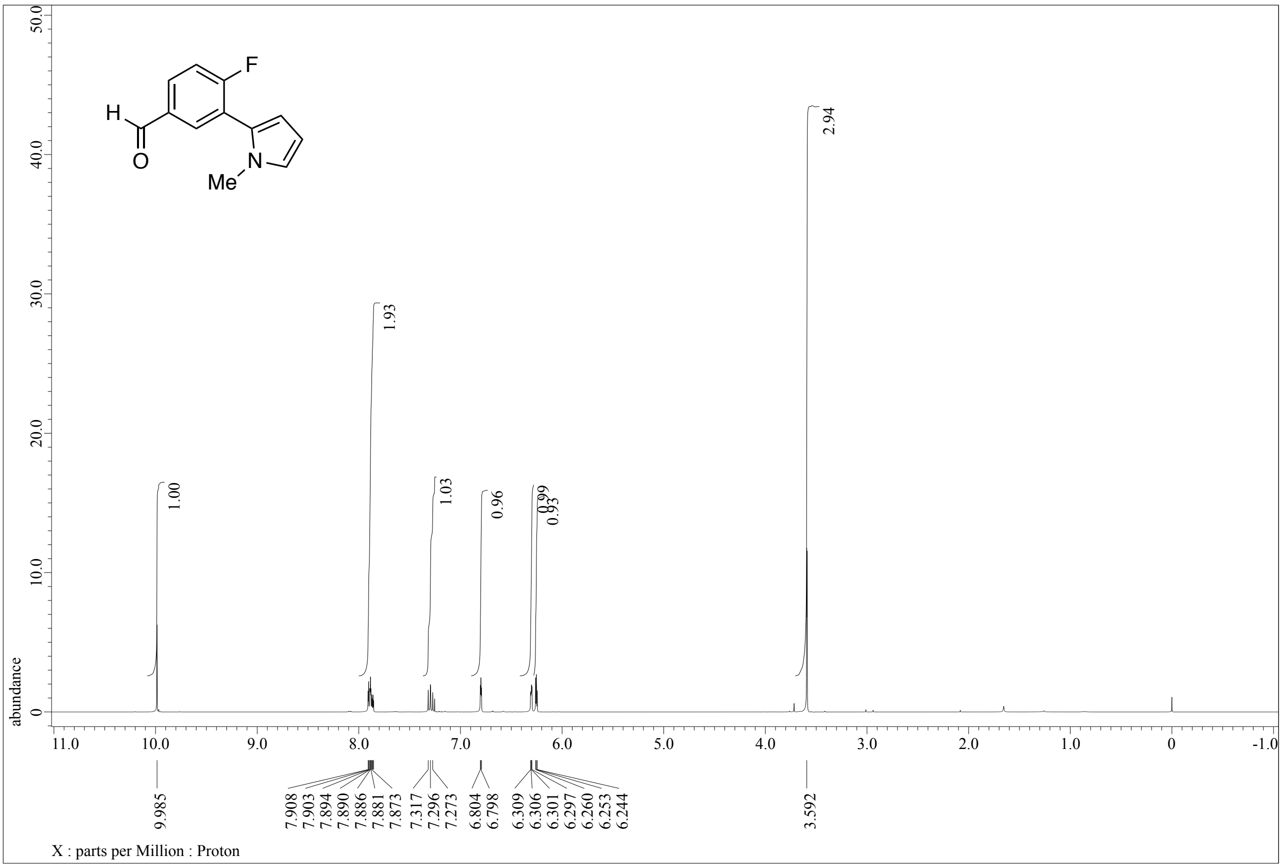

Supplementary Figure 71. ^{1}H NMR spectrum of **15e** ($CDCl_3$, 399.8 MHz, 20.2 ºC).

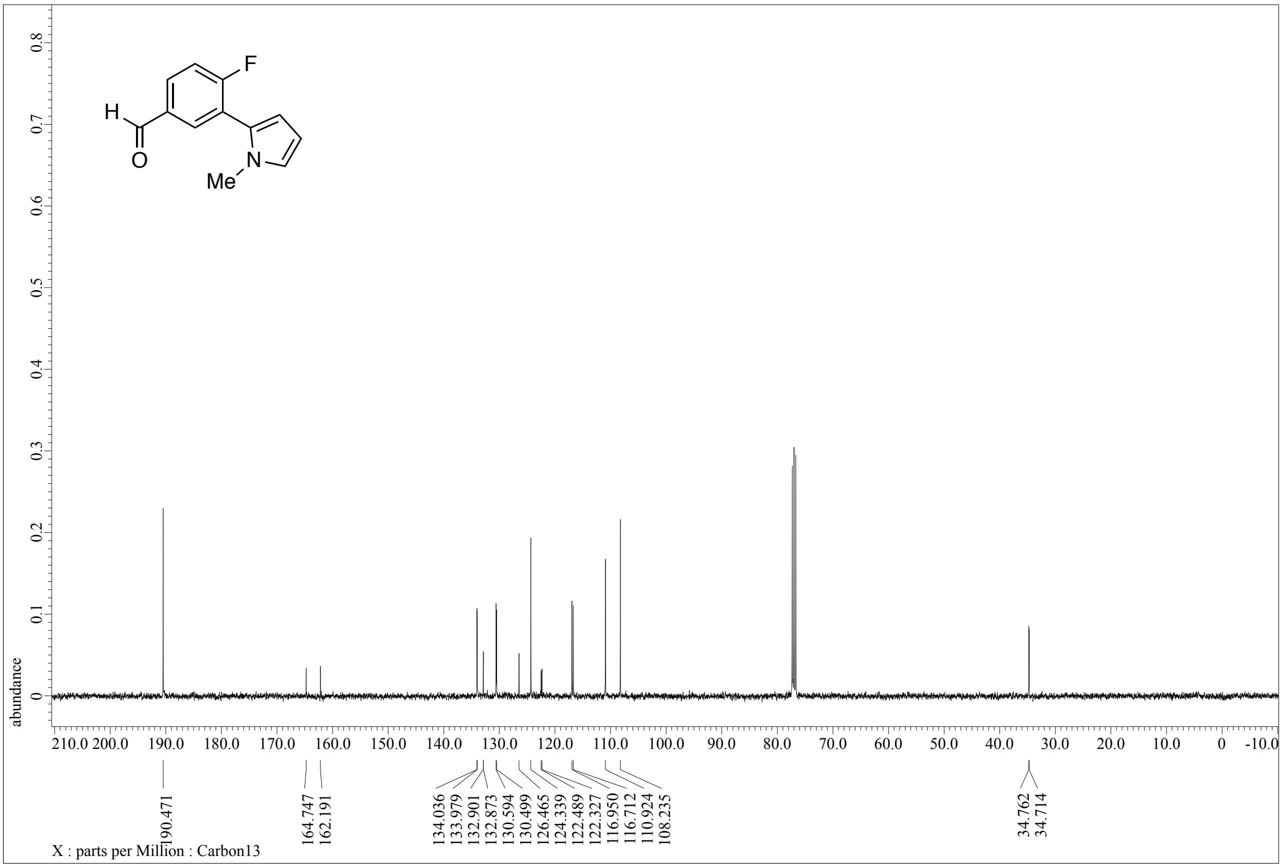

Supplementary Figure 72. ^{13}C NMR spectrum of **15e** ($CDCl_3$, 100.5 MHz, 20.4 ºC).

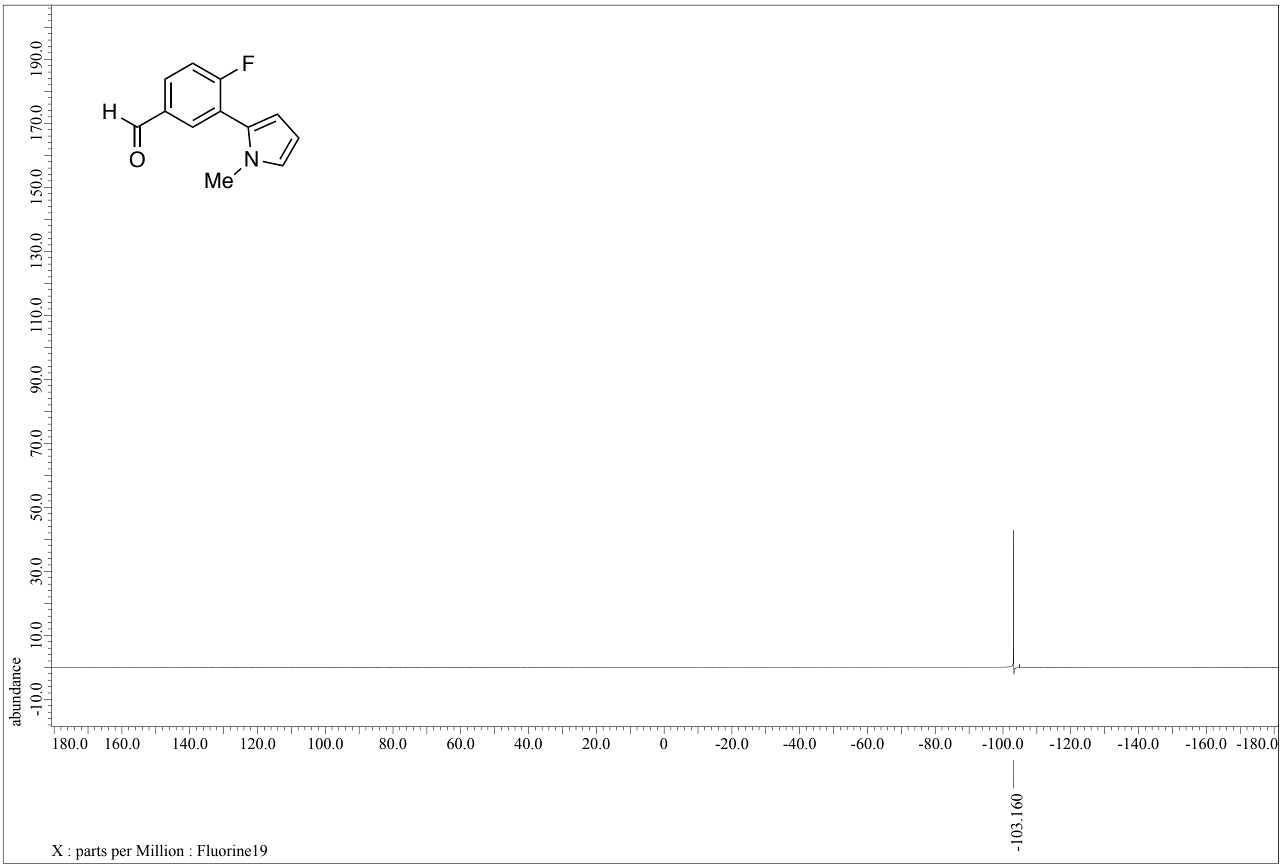

Supplementary Figure 73. ^{19}F NMR spectrum of **15e** ($CDCl_3$, 376.2 MHz, 20.3 ºC).

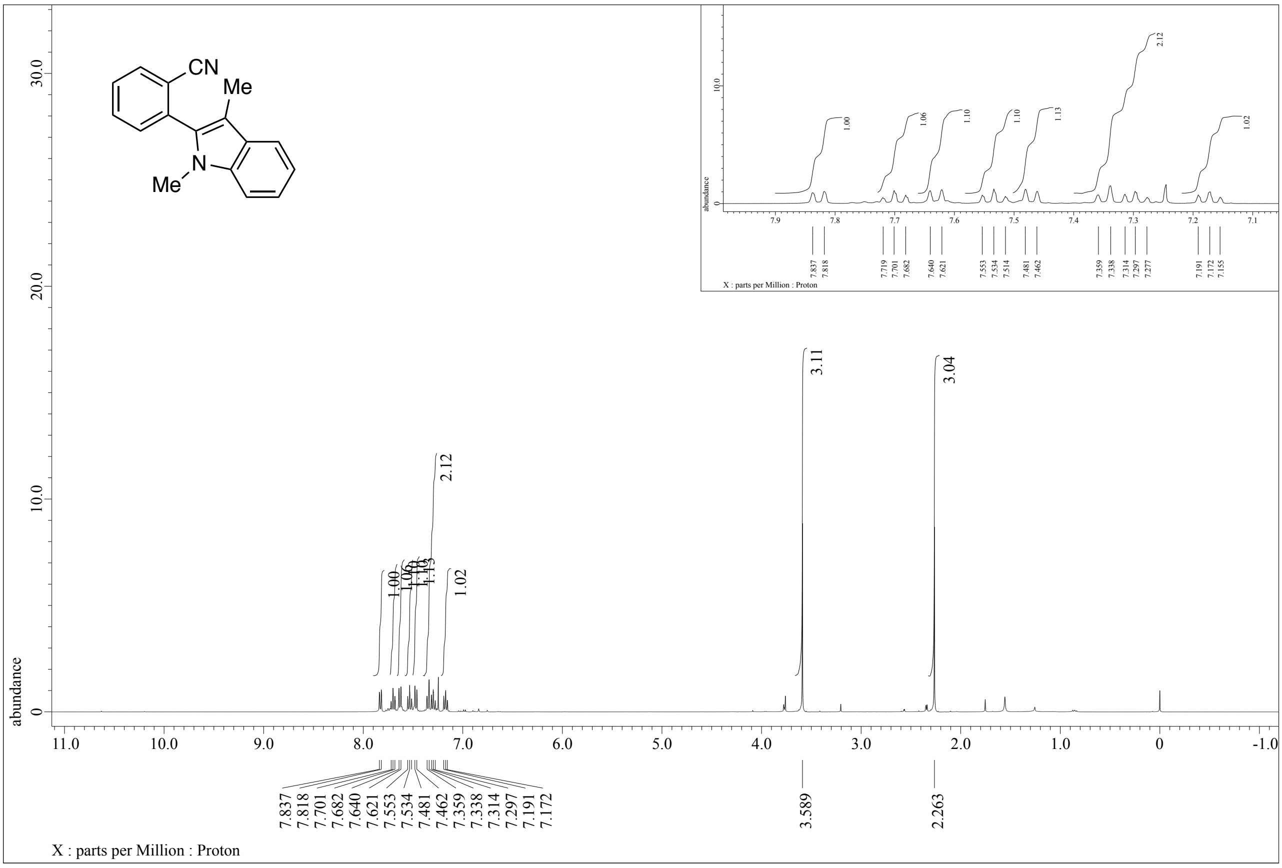

Supplementary Figure 74. ^{1}H NMR spectrum of **15l** ($CDCl_3$, 399.8 MHz, 23.8 ºC).

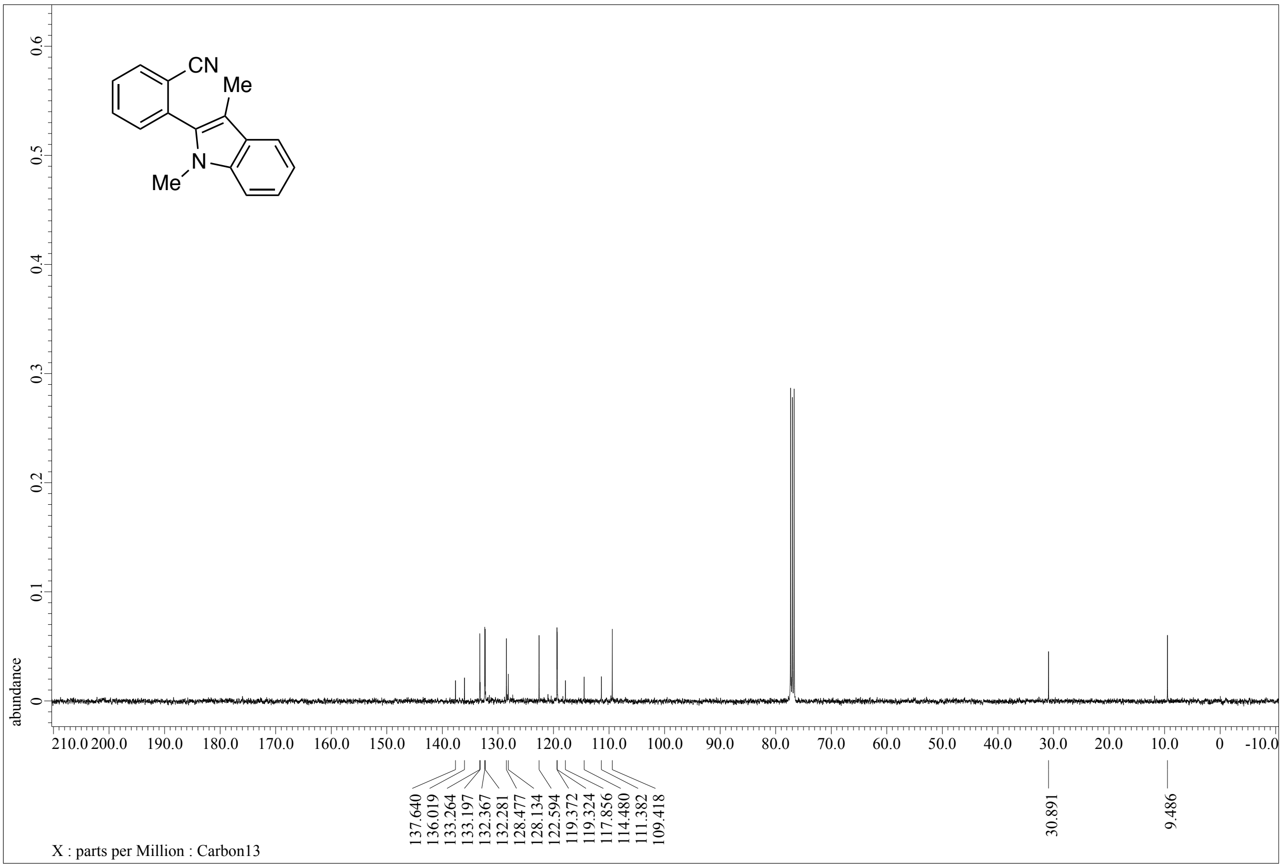

Supplementary Figure 75. ^{13}C NMR spectrum of **15l** ($CDCl_3$, 100.5 MHz, 24.1 ºC).

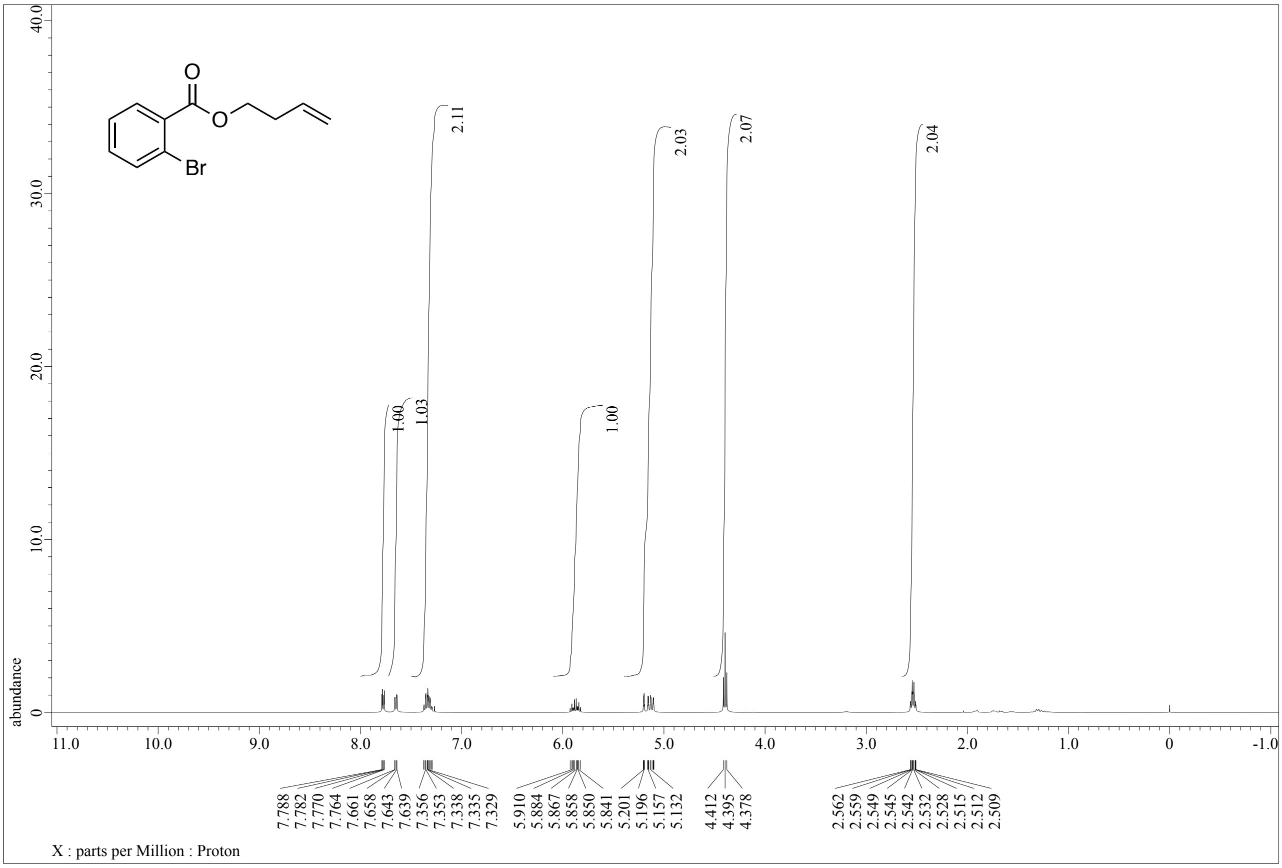

Supplementary Figure 76. ^{1}H NMR spectrum of **16p** ($CDCl_3$, 399.8 MHz, 23.4 ºC).

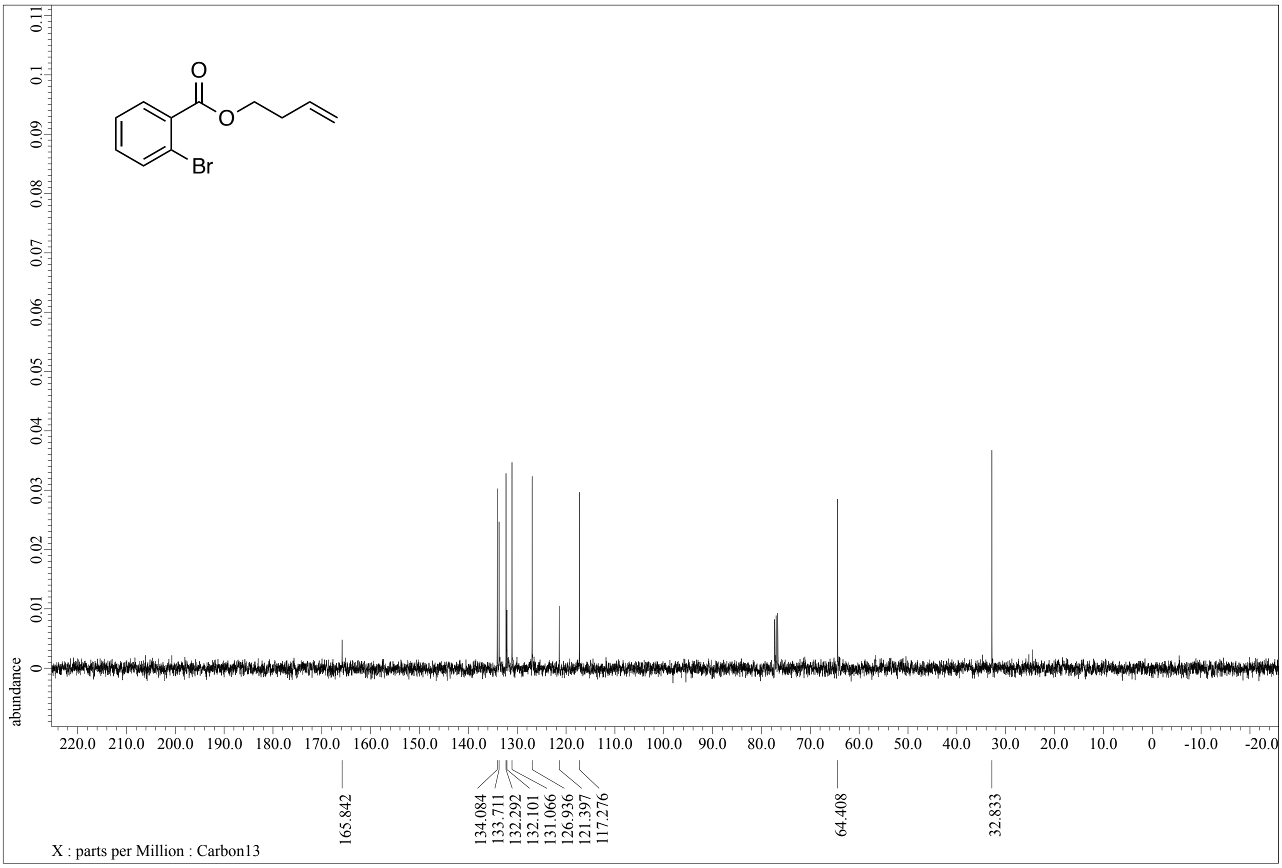

Supplementary Figure 77. ^{13}C NMR spectrum of **16p** ($CDCl_3$, 100.5 MHz, 22.4 ºC).

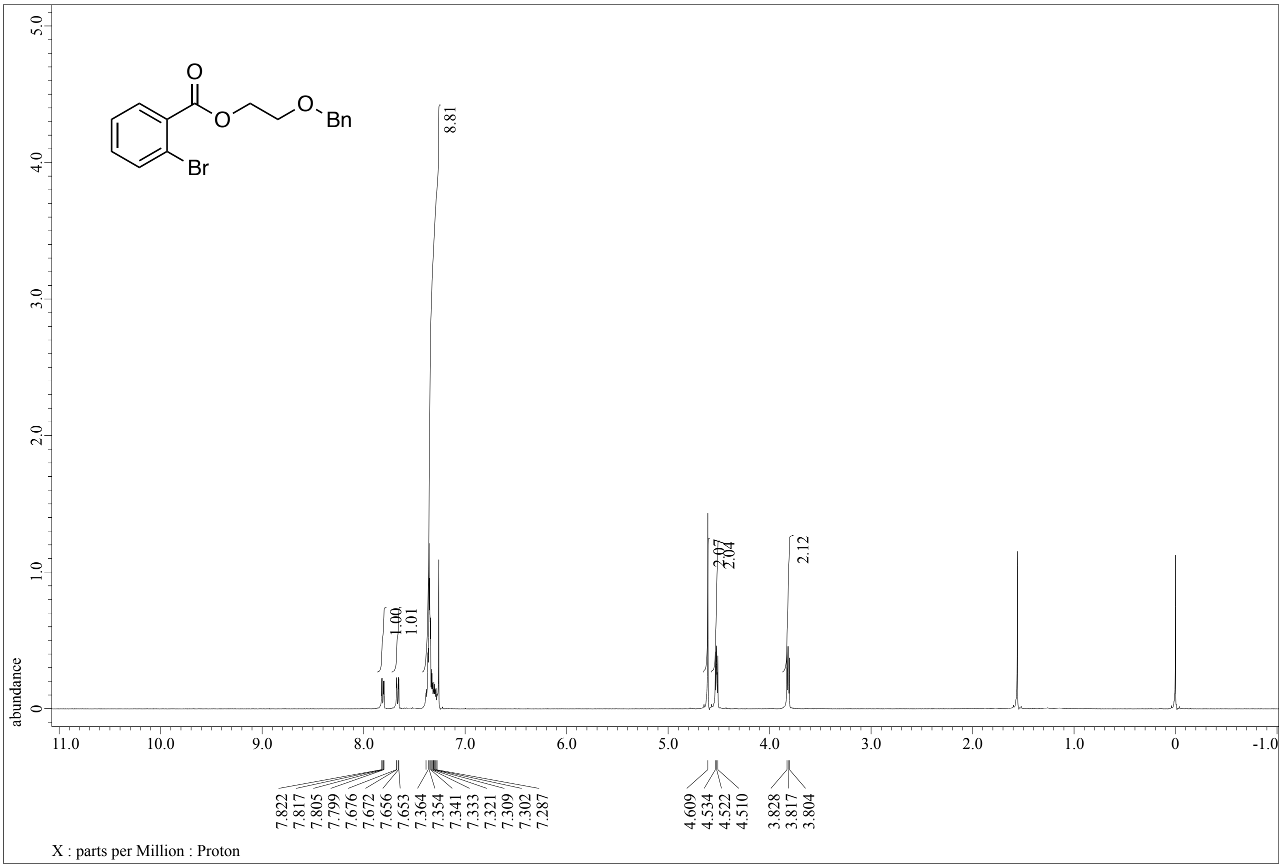

Supplementary Figure 78. ^{1}H NMR spectrum of **16q** ($CDCl_3$, 399.8 MHz, 22.5 ºC).

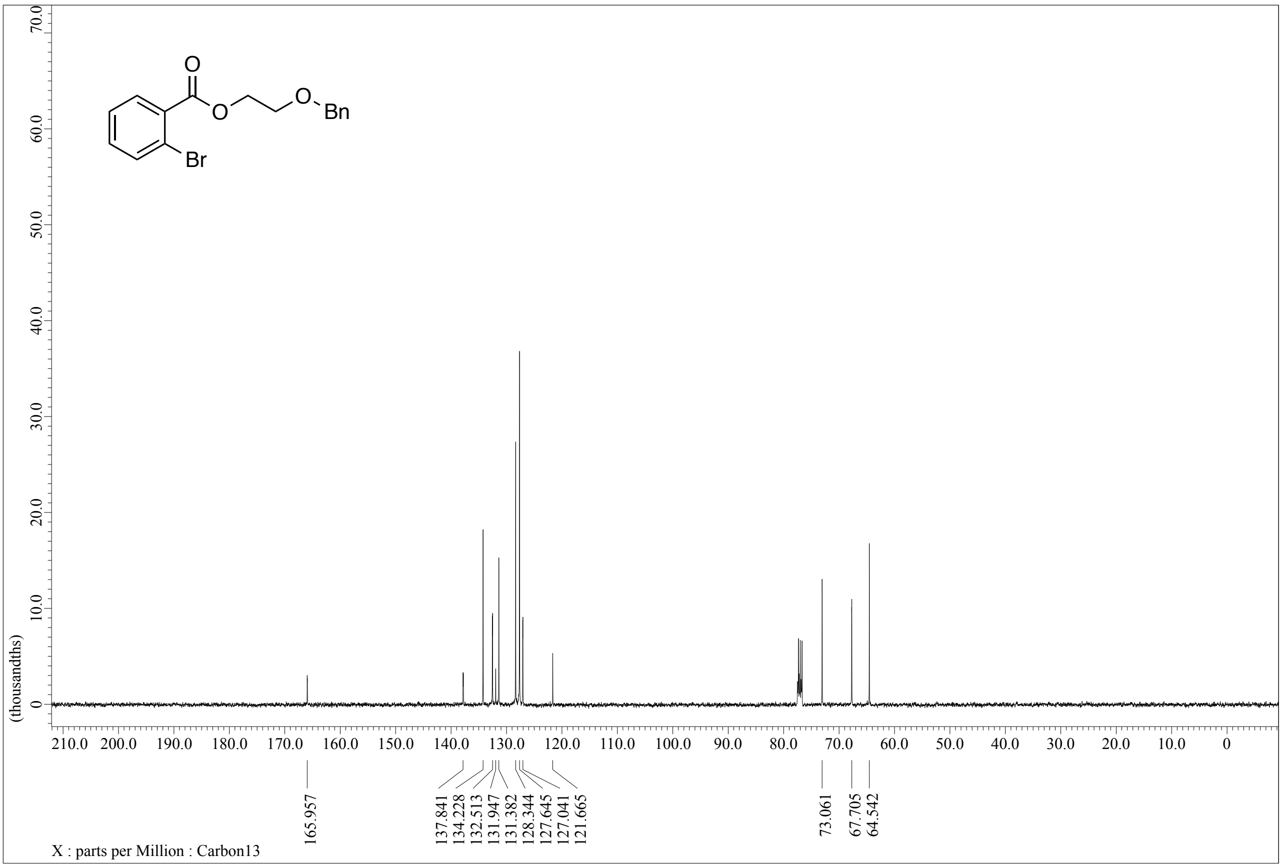

Supplementary Figure 79. ^{13}C NMR spectrum of **16q** ($CDCl_3$, 100.5 MHz, 22.5 ºC).

3. Supplementary References

(1) Frisch, M. J., Trucks, G. W., Schlegel, H. B., Scuseria, G. E., Robb, M. A., Cheeseman, J. R., Scalmani, G., Barone, V., Petersson, G. A., Nakatsuji, H., Li, X., Caricato, M., Marenich, A. V., Bloino, J., Janesko, B. G., Gomperts, R., Mennucci, B., Hratchian, H. P., Ortiz, J. V., Izmaylov, A. F., Sonnenberg, J. L., Williams-Young, D., Ding, F., Lipparini, F., Egidi, F., Goings, J., Peng, B., Petrone, A., Henderson, T., Ranasinghe, D., Zakrzewski, V. G., Gao, J., Rega, N., Zheng, G., Liang, W., Hada, M., Ehara, M., Toyota, K., Fukuda, R., Hasegawa, J., Ishida, M., Nakajima, T., Honda, Y., Kitao, O., Nakai, H., Vreven, T., Throssell, K., Montgomery, J. A., Jr., Peralta, J. E., Ogliaro, F., Bearpark, M. J., Heyd, J. J., Brothers, E. N., Kudin, K. N., Staroverov, V. N., Keith, T. A., Kobayashi, R., Normand, J., Raghavachari, K., Rendell, A. P., Burant, J. C., Iyengar, S. S., Tomasi, J., Cossi, M., Millam, J. M., Klene, M., Adamo, C., Cammi, R., Ochterski, J. W., Martin, R. L., Morokuma, K., Farkas, O., Foresman, J. B. & Fox, D. J. Gaussian 16, Rev. C.01; Gaussian, Inc.: Wallingford, CT, 2016.

(2) Yu, H. S., He, X., Li, S. L. & Truhlar, D. G. *Chem. Sci.* **7**, 5032–5051 (2016).

(3) Andrae, D., Häuβermann, U., Dolg, M., Stoll, H. & Preuβ, H. *Theor. Chim. Acta* **77**, 123−141 (1990).

(4) (a) Hehre, W. J., Ditchfield, R. & Pople, J. A. *J. Chem. Phys.* **56**, 2257−2261 (1972). (b) Hariharan, P. C. & Pople, J. A. *Theor. Chim. Acta* **28**, 213−222 (1973). (c) Francl, M. M., Pietro, W. J., Hehre, W. J., Binkley, J. S., Gordon, M. S., DeFrees, D. J. & Pople, J. *J. Chem. Phys.* **77**, 3654–3665 (1982).

(5) Scalmani, G. & Frisch, M. J. *J. Chem. Phys.* **132**, 114110 (2010).

(6) Chai, J.-D. & Head-Gordon, M. *Phys. Chem. Chem. Phys.* **10**, 6615–6620 (2008).

(7) Yanai, T., Tew, D. P. & Handy, N. C. *Chem. Phys. Lett.* **393**, 51–57 (2004).

(8) (a) McLean, A. D. & Chandler, G. S. *J. Chem. Phys.* **72**, 5639−5648 (1980). (b) Krishnan, R., Binkley, J. S., Seeger, R. & Pople, J. *J. Chem. Phys.* **72**, 650−654 (1980). (c) Clark, T., Chandrasekhar, J., Spitznagel, G. W. & Schleyer, P. v. R. *J. Comput. Chem.* **4**, 294–301 (1983).

(9) Rehm, D. & Weller, A. *Isr. J. Chem.* **8**, 259 (1970).

(10) Roth, H. G., Romero, N. A. & Nicewicz, D. A. *Synlett* **27**, 714–723 (2016).

(11) Pavlishchuk, V. V. & Addison, A. W. *Inorg. Chim. Acta.* **298**, 97–102 (2000).

(12) Hu, A., Chen, Y., Guo, J.-J., Yu, N., An, Q. & Zuo, Z. *J. Am. Chem. Soc.* **140**, 13580–13585 (2018).

(13) Montalti, M., Credi, A., Prodi, L. & Gandolfi, M. T. Handbook of Photochemistry, 3rd ed., CRC, Taylor & Francis, Boca Raton, 2006.

(14) Wu, C.-L., Chang, C.-H., Chang, Y.-T., Chen, C.-T., Chen, C.-T. & Su, C.-J. *J. Mater. Chem. C* **2**, 7188–7200 (2014).

(15) Shaikh, T. M., Weng, C.-M. & Hong, F.-E *Coord. Chem. Rev.* **256**, 771–803 (2012).

(16) (a) Tanoue, A., Yoo, W.-J. & Kobayashi, S. *Org. Lett.* **16**, 2346–2349 (2014). (b) Luo, W., Yang, J.-D. & Cheng, J.-P. *iScience*, **23**, 100851 (2020). (c) Xu, C., Zhu, Z., Wang, Y., Jing, Z., Gao, B., Zhao,

L. & Dong, W.-K. *J. Org. Chem.* **84**, 2234–2242 (2019).
(17) Chen, H., Jia, X., Yu, Y., Qian, Q. & Gong, H. *Angew. Chem. Int. Ed.* **56**, 13103–13106 (2017).
(18) Song, D., Cho, S., Han, Y.,You, Y. & Nam, W. *Org. Lett.* **15**, 3582–3585 (2013).
(19) Hussain, M. I., Feng, Y., Hu, L., Deng, Q., Zhang, X. & Xiong, Y. *J. Org. Chem.* **83**, 7852–7859 (2018).
(20) Greulich, T. W., Daniliuc, C. G. & Studer, A. *Org. Lett.* **17**, 254–257 (2015).
(21) Xuan, J., Zeng, T.-T., Feng, Z.-J., Deng, Q.-H., Chen, J.-R., Lu, L.-Q., Xiao, W.-J. & Alper, H. *Angew. Chem. Int. Ed.* **54**, 1625–1628 (2015).
(22) Feng, Z., Zeng, T., Xuan, J., Liu, Y., Lu, L. & Xiao, W.-J. *Sci. China Chem.* **59**, 171–174 (2016).
(23) Li, Z., Ma, P., Tan, Y., Liu, Y., Gao, M., Zhang, Y., Yang, B., Huang, X., Gao, Y. & Zhang, J. *Green Chem.* **22**, 646 (2020).
(24) Kurandina, D., Rivas, M., Radzhabov, M. & Gevorgyan, V. *Org. Lett.* **20**, 357–360 (2018).
(25) Koy, M., Sanfort, F., Tlahuext-Aca, A., Quach, L., Daniliuc, C. G. & Glorius, F. *Chem. Eur. J.* **24**, 4552–4555 (2018).
(26) Wang, G.-Z., Shang, R., Cheng, W.-M. & Fu, Y. *J. Am. Chem. Soc.* **139**, 18307–18312 (2017).
(27) Zong, Z., Wang, W., Bai, X., Xi, H. & Li, Z. *Asian. J. Org. Chem.* **4**, 622–625 (2015).
(28) Shi, H., Dai, W., Wang, B. & Cao, S. *Organometallics* **37**, 459–463 (2018).
(29) Mai, W.-P., Song, G., Sun, G.-C., Yang, L.-R., Yuan, J.-W., Xiao, Y.-M., Mao, P. & Qu, L.-B. *RSC Advances* **3**, 19264–19267 (2013).
(30) Lu, X.-L., Shannon, M., Peng, X.-S. & Wong, H. N. C. *Org. Lett.* **21**, 2546–2549 (2019).
(31) Zhang, Y.-L., Yang, L., Wu, J., Zhu, C. & Wang, P. *Org. Lett.* **22**, 7768–7772 (2020).
(32) Huang, H.-J., Wang, Y.-T., Wu, Y.-K. & Ryu, I. *Org. Chem. Front.* **7**, 1266–1270 (2020).
(33) Constantin, T., Juliá, F., Sheikh, N. S. & Leonori, D. *Chem. Sci.* **11**, 12822–12828 (2020).
(34) Yu, F., Mao, R., Yu, M., Gu, X. & Wang, Y. *J. Org, Chem.* **84***,* 9946–9956 (2019).
(35) Marzo, L., Ghosh, I., Esteban, F. & König, B. *ACS Catal.* **6**, 6780–6784 (2016).
(36) Ghosh, I., Shaikh, R. S. & König, B. *Angew. Chem. Int. Ed.* **56**, 8544–8549 (2017).
(37) Tokuyasu, T., Kunikawa, S., McCullough, K. J., Masuyama, A. & Nojima, M. *J. Org. Chem.* **70**, 251–260 (2005).
(38) Gathirwa, J. W. & Maki, T. *Tetrahedron* **68**, 370–375 (2012).
(39) Zhu, X., Lin, Y., Martin, J. S., Sun, Y., Zhu, D. & Yan, Y. *Nat. Commun.* **10**, 2843 (2019).
